# Steric Restraints in Redox‐Active Guanidine Ligands and Their Impact on Coordination Chemistry

**DOI:** 10.1002/chem.202502457

**Published:** 2025-10-25

**Authors:** Eliane Engels, Hanna Koepcke, Marko Lörsch, Patrick David Römgens, Anna Katharina Helm, Simone Leingang, Elisabeth Kaifer, Hans‐Jörg Himmel

**Affiliations:** ^1^ Ruprecht‐Karls‐Universität Heidelberg: Universität Heidelberg Institut für Anorganische Chemie Heidelberg 69120 Germany

**Keywords:** coordination, guanidine, macrocyclic rings, redox‐active ligands, steric restraint

## Abstract

The introduction of redox‐active ligands into coordination compounds is attractive for a number of applications; intramolecular electron transfer between a redox‐active ligand and a metal is the basis for applications in switchable devices and advanced redox catalysis for multielectron substrate activations. A fine‐tuning of the properties of redox‐active ligands focusses on the redox potential and frontier orbital energies, as well as the steric demand and coordination mode. In this work, we report the first synthesis of new *o*‐diguanidino‐benzene ligands in which the two guanidino groups are connected through an alkyl chain of different length. The introduction of an ethylene bridge between the two guanidino groups turns a strongly coordinating ligand into a noncoordinating molecule, while keeping its strong Brønsted basicity. These features qualify the ethylene‐bridged compound as a powerful alternative to Huenig's base, diisopropyl‐ethyl‐amine. The change from an ethylene to a propylene bridge switches back on the coordination ability. It is also possible to introduce a methyl group to the central C atom of the propylene bridge without loss of nucleophilic properties. The analysis, based on a variety of metal complexes, identifies three structural parameters to assay the structural restraint. Macrocyclic tetraguanidines are also formed.

## Graphical abstract

One carbon makes the difference; a diguandine with an ethylene bridge does not coordinate metals, while a diguanidine with a propylene bridge coordinates to a variety of metals. In this work, we report the synthesis and coordination chemistry of the first cyclic diguanidino‐benzene ligands and compare their coordination chemistry with that of open diguanidino‐benzene ligands.

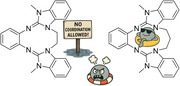

## 1 Introduction

The design of ligands with confined coordination space is a very important issue in coordination chemistry, since it allows to drive chemical reactions highly selectively in a rational approach. Such ligands bind selectively to metals and control the approach of two reactants. A confined coordination space also could favor special coordination modes with distinct physical and chemical properties. A number of strategies have been developed for the introduction of steric constraints. First, the steric demand of the ligands could be tuned,^[^ ^1^ ^]^ for example, the Tolman cone angle of phosphines.^[^ ^2^, ^3^, ^4^ ^]^ Here, the steric constraint applies to the steric interactions with the coordinated metal and coligands. The electronic properties (frontier orbital energies) of the donor atom could also be affected by steric constraints, e.g. the P atom in bicyclic phosphines.^[^ ^5^ ^]^ A variety of *N*‐heterocyclic carbenes was synthesized in which the sterics control the metal coordination and the chemical properties of metal complexes.^[^ ^6^, ^7^, ^8^ ^]^ Moreover, supramolecular chemistry was used to form confined coordination spaces such as self‐assembled molecular cages and molecular cavities from specially designed ligand and metal building blocks.^[^ ^9^, ^10^, ^11^, ^12^, ^13^, ^14^ ^]^

**Figure 1:**
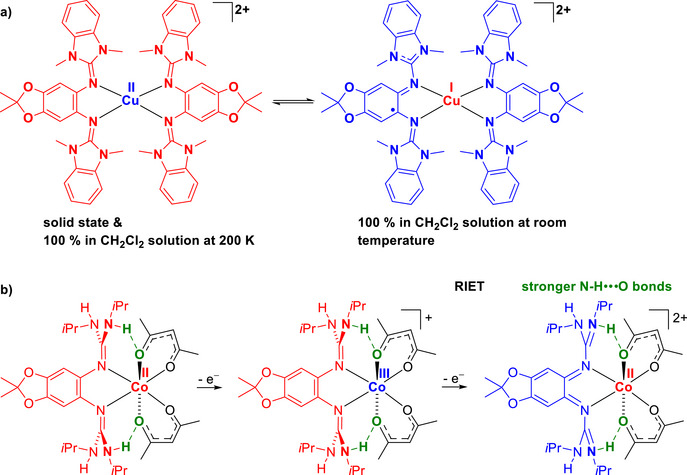
a) Homoleptic copper complex with two diguanidine ligands showing quantitative redox isomerism. b) Oxidation of a neutral Co^II^‐diguanidine complex in two steps to give first Co^III^ and then again Co^II^.

In the past, we intensively studied redox‐active guanidino‐functionalized aromatic compounds and used them in coordination chemistry for the design of switchable bistable molecules.^[^ ^15^, ^16^, ^17^ ^]^ Two examples are shown in Figure 1. The homoleptic copper complex in Figure 1a is present as Cu^I^ complex with a ligand‐centered unpaired electron at room temperature, and is quantitatively converted to the Cu^II^ redox isomer with two neutral, reduced diguanidine ligands at low temperature.^[^ ^18^ ^]^ In the case of the cobalt complex with acetylacetonate (acac) coligands and N─H functions at the guanidino groups sketched in Figure 1b, the first one‐electron oxidation is metal‐centered, converting the neutral high‐spin Co^II^ complex into a monocationic low‐spin Co^III^ complex.^[^ ^19^ ^]^ The second one‐electron oxidation leads to a high‐spin Co^II^ complex with fully‐oxidized, dicationic diguanidine ligand. Overall oxidation triggers metal reduction, being an example of a redox‐induced electron transfer (RIET) process.

In the examples shown in Figure 1, the diguanidine ligand is substituted para to the guanidino groups. The substituents fulfill two tasks. First, they allow a fine‐tuning of the redox properties. Second, they prohibit attacks at the aromatic ring upon oxidation.

**Figure 2:**
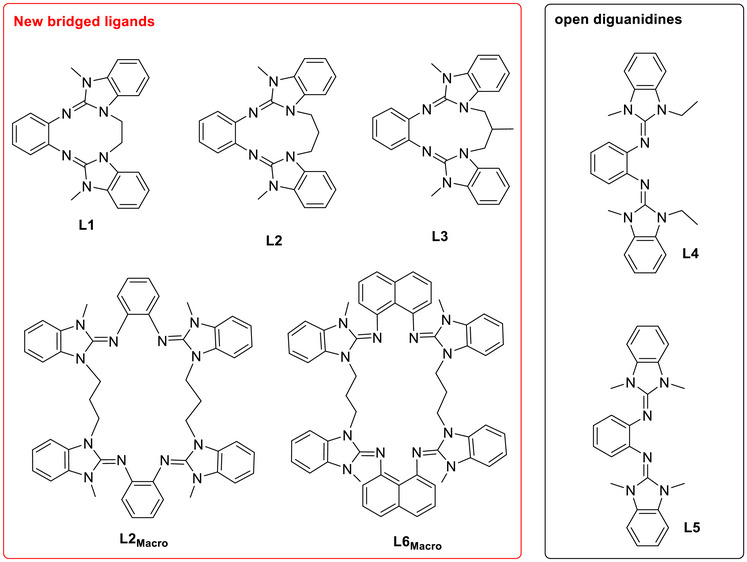
Cylic (L1‐L3) and open (L4, L5) diguanidine ligands and the macrocyclic tetraguanidines L2_Macro_ and L6_Macro_ that are used in this work. L5 was reported previously, (ref. [20]) all other compounds are new.

In this paper, we report the first redox‐active cyclic diguanidine ligands, L1‐L3 (Figure 2), in which the two guanidino groups are connected not only through the aromatic core, but also through an alkyl bridge. Macrocyclic tetraguanidine dimers, in which the linker connects two guanidino groups of different redox‐active diguanidine units, are also obtained. We show that the linker between the two guanidino groups significantly influences the chemical properties. To better compare the properties of the compounds with connected guanidino groups (cyclic structures) and with unconnected guanidino groups (open structures), we synthesized the open diguanidines L4 and L5 (already known from previous work^[^ ^20^ ^]^) through reaction of *o‐*phenylenediamine with a bis(imidazolium) salt. In this work, we do not protect the aromatic positions para to the guanidino groups by introduction of substituents, that generally prohibit coupling reactions upon oxidation (see discussion below).

## 2 Results and Discussion

### 2.1 Ethylene‐Bridged Ligand

#### 2.1.1 Synthesis and Characterization

**Scheme 1:**
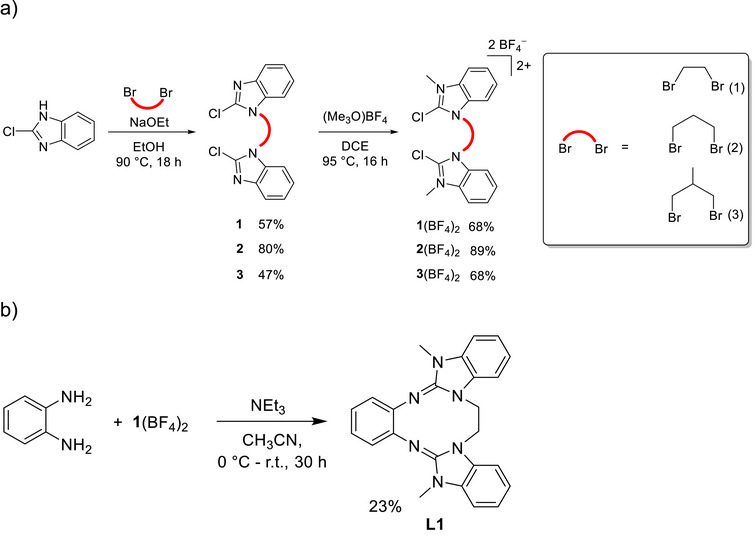
a) General scheme for the synthesis of the new bridged, activated urea species **1**(BF_4_)_2_–**3**(BF_4_)_2_. b) Synthesis of the new cyclic diguanidine L1 with ethylene‐bridged guanidino groups.

We started with the synthesis of the new bis‐2‐chloro‐1,3‐dialkyl‐imidazolinium salts (activated ureas) **1**(BF_4_)_2_, **2**(BF_4_)_2_, and **3**(BF_4_)_2_ through reaction of the respective dibromo derivative with 2‐chlor‐1H‐benzimidazole. Methylation of **1**, **2**, and **3** with (Me_3_O)BF_4_ leads to the bis‐2‐chloro‐1,3‐dialkyl‐imidazolinium salts in yields of 68–89% (Scheme 1a). Then, *o*‐phenylenediamine was reacted with **1**(BF_4_)_2_ to give the new ethylene‐bridged, cyclic diguanidine L1 in 23% isolated yield (Scheme 1b). The product precipitates from the reaction mixture and can therefore be easily isolated by filtration.

#### 2.1.2 Coordination Chemistry

**Figure 3:**
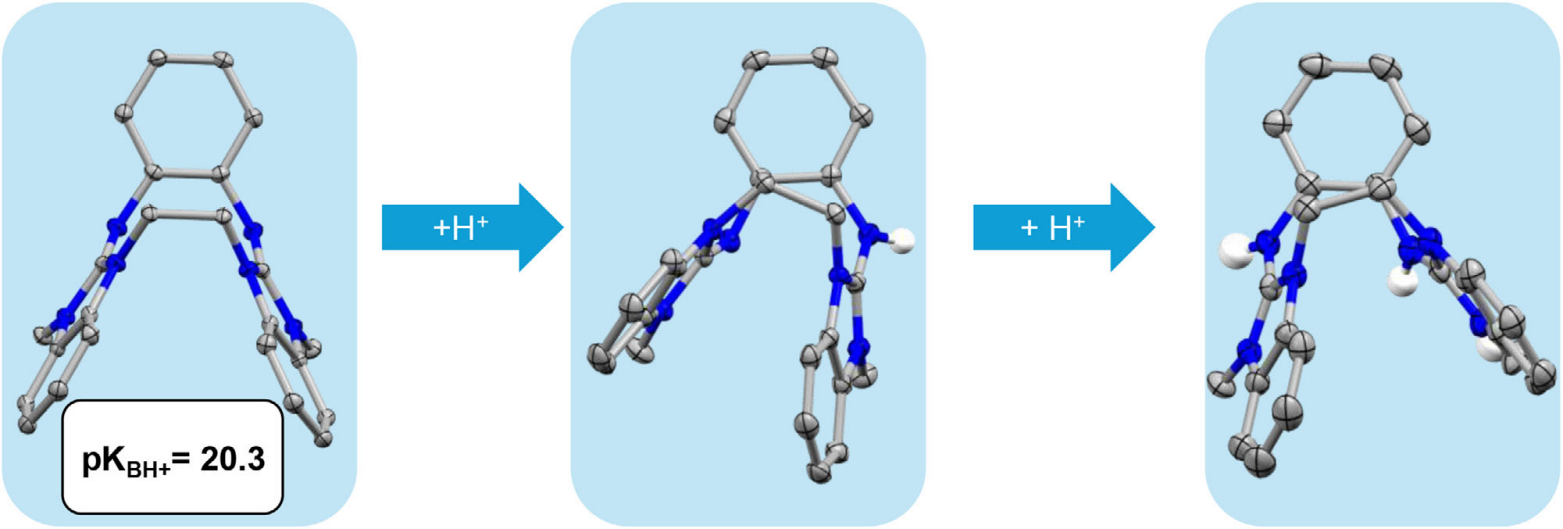
Illustration of the solid‐state structures of L1, (L1 + H)^+^ and (L1 + 2H)^2+^. Displacement ellipsoids drawn at the 50% probability level. C─H hydrogen atoms and counterions are omitted. Color code: H white, C grey, N blue.

The solid‐state structure of L1 (Figure 3) exhibits a saddle‐like shape, that is well reproduced by quantum‐chemical calculations on the isolated molecule (see discussion below and Supporting Information for details). Generally, metals coordinate preferably to the imino nitrogen of the guanidino groups and are located in the plane of the aromatic core. Due to the presence of the ethylene bridge, metal coordination to L1 is disfavored by steric restraint. We tested reactions with cobalt (CoCl_2_, CoBr_2_, Co(hfac)_2_) (hfac = hexafluoro‐acetylacetonate), zinc (ZnCl_2_, Zn(OAc)_2_), and copper compounds (CuBr_2_ and Cu(BF_4_)_2_), that usually bind strongly to *o*‐diguanidino‐benzene derivatives. However, in the case of L1, no coordination was observed. Also, L1 does not coordinate to LiCl, Al(acac)_3_, BF_3_·Et_2_O, and NiCl_2_·DME. The results show that L1 is the first noncoordinating *o*‐diguanidino‐benzene derivative.

#### 2.1.3 Brønsted basicity and applications in synthetic chemistry

Although L1 is a noncoordinating diguanidine, it still is a strong two‐protonic Brønsted base like all *o*‐diguanidino‐benzene molecules. Since the experimental determination of the Brønsted basicity is hampered by protonation equilibria, we utilized the well‐established method developed originally by Maksić and Kovačević to estimate the basicity constants in acetonitrile.^[^ ^21^, ^22^ ^]^ This method has already been shown to give reliable values for GFA molecules.^[^ ^23^, ^24^, ^25^ ^]^ A linear correlation was obtained by plotting the calculated proton affinities corrected by the solvent effect for acetonitrile, PA(CH_3_CN), as a function of the experimentally known values for various strong nitrogen bases in CH_3_CN (Equation 1).

(1) $$\mathrm{pK}\left(BH^{+}\right)=0.4953\mathrm{PA}\left({\mathrm{CH}}_{3}\mathrm{CN}\right)/\left(\mathrm{kcal}\;\mathrm{mo}l^{-1}\right)-119.7,$$

where BH^+^ is the conjugated acid of the base B. The proton affinity (PA) in CH_3_CN follows from Equation 2.

(2) $$\begin{array}{ccc} \mathrm{PA}\left(CH_{3}\mathrm{CN}\right) & = & \left[E_{\mathrm{el}}\left(B\right)-E_{\mathrm{el}}\left(BH^{+}\right)]+[\mathrm{ZPVE}\left(B\right)-\mathrm{ZPVE}\left(BH^{+}\right)\right]\\ & = & \Delta E_{\mathrm{el}}+\Delta \left(\mathrm{ZPVE}\right), \end{array}$$

where ZPVE denotes the zero‐point vibrational energy in the gas‐phase (without environmental interactions) and *E*_el_ the solvent‐corrected electronic energy.^[^ ^12^ ^]^

The calculations (B3LYP/def2‐TZVP) confirm the large basicity of the newly synthesized L1, giving a p*K*(BH^+^) value (20.3) more than twice as high as triethylamine (9.0 in DMSO^[^ ^18^ ^]^) and Hünig‘s base (8.5 in DMSO^[^ ^26^ ^]^), and in the same range as the open diguanidine L5 (20.5). In experiments with two equivalents of HCl in diethylether, (L1 + 2H)Cl_2_ was obtained quantitatively. The solid‐state structures of (L1 + H)OTf and (L1 + 2H)Cl_2_ are shown in Figure 3. It can be seen that mono‐ and di‐protonation introduce a distortion to the highly‐symmetric saddle shape of the neutral form (Figure 3).

**Scheme 2:**
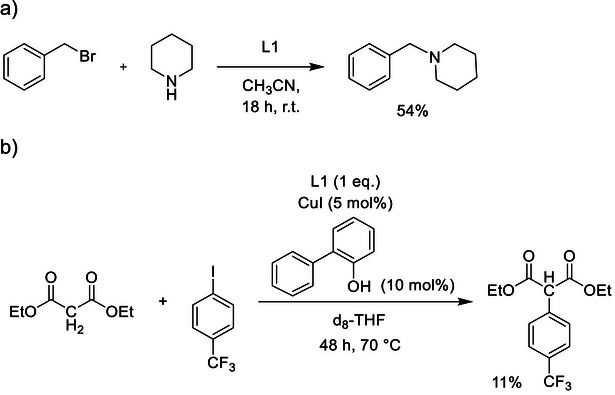
a) Test for the use of L1 and L5 in an amine alkylation reaction. b) Example for the use of L1 as a base in a catalytic coupling reaction.

Due to its large Brønsted basicity in combination with the noncoordinating behavior, L1 could be used like Huenig's base, NEt(*i*Pr)_2_, in (catalytic) reactions as a noncoordinating two‐protonic base. First, a typical amine alkylation reaction of piperidine with benzylbromide was tested in the presence of cyclic diguanidine L1 or open diguanidine L5 in place for Huenig's base.^[^ ^27^ ^]^ For both ligands, the expected product formation is observed with ^1^H NMR spectroscopy and mass spectrometry, in analogy to Huenig's base (Scheme 2a). For L1, the product was obtained in 54% yield. Some unprotonated neutral ligand remained (ca. 17% of the product mass) after filtration of the crude product and extraction with dichloromethane.^[^ ^28^ ^]^ In the case of L5, additional side products formed, and a larger amount of the protonated ligand could not be separated from the desired product. Hence, the synthesis works better with cyclic L1 than with open L5.

Additionally, we tested the use of L1 as a base in a catalytic coupling reaction with CuI as catalyst (Scheme 2b).^[^ ^29^ ^]^ Generally, diguanidines strongly coordinate to CuI,^[^ ^30^ ^]^ thereby prohibiting activation of the aryl iodide. However, the cyclic diguanidine L1 is a non‐coordinating base, that does not interact with CuI. In the literature, this reaction was carried out in the presence of Cs_2_CO_3_ as a base, while Hünig's base does not work at all for this reaction.^[^ ^29^ ^]^ Under the applied conditions (Scheme 2b), the product was obtained in 11% yield. Although this yield is not very high, the results still show that L1 is a more capable base than Huenig's base for this type of reactions.

#### 2.1.4 Redox Properties of L1 in Comparison to L4/L5

**Figure 4:**
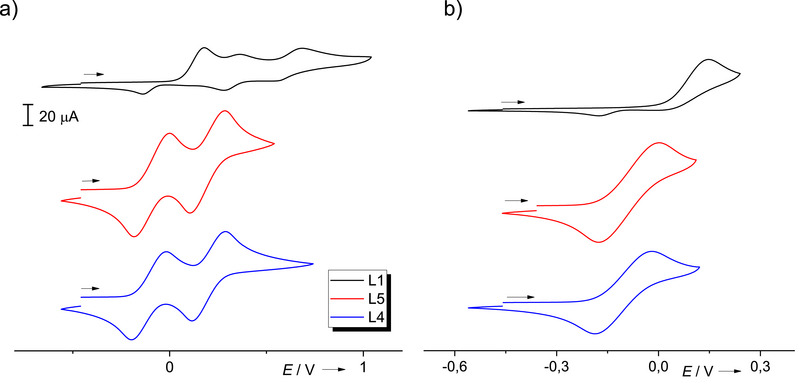
CV curves of cyclic L1 and open L4/L5 (*n*Bu_4_NPF_6_, Ag/AgCl electrode) in CH_2_Cl_2_ solution. Potentials are given relative to the reference redox couple ferrocenium/ferrocene. a) Complete CV curves. b) Scan reversed after the first oxidation wave.

**Figure 5:**
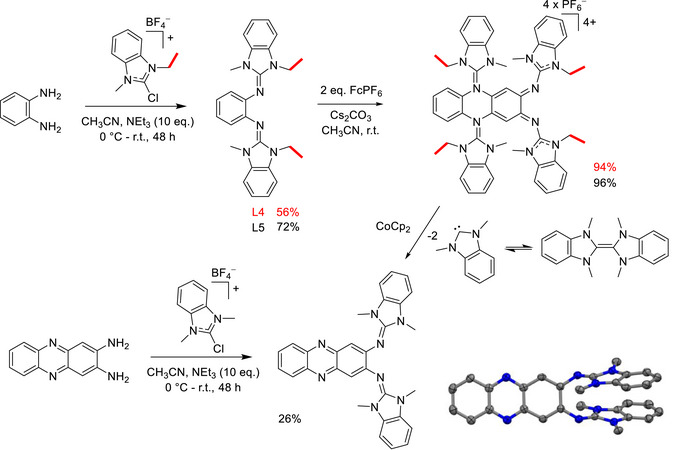
Scheme for the preparation of L4 and L5 and their reactions with FcPF_6_. Two‐electron oxidation is accompanied by dimerization to tetracationic dimers. The reduction of L5 leads to formal elimination of two NHC's and the formation of a 2,3‐diguanidino‐phenazine. Illustration of the solid‐state structure included. Displacement ellipsoids drawn at the 50% probability level. C─H hydrogen atoms are omitted. Color code: C grey, N blue.

The open diguanidines L4 and L5 exhibit two quasi‐reversible redox events at *E*_1/2_  =  −0.10 V (*E*_ox_ = −0.04 V) and *E*_1/2_ = 0.21 V (*E*_ox_ = 0.28 V) for L4 and *E*_1/2_ = −0.09 V (*E*_ox_  =  −0.02 V) and *E*_1/2_  =  0.19 V (*E*_ox_ = 0.25 V) for L5 ^[^ ^11^ ^]^ in dichloromethane (Figure 4). Although the reversible redox processes in the CV curves of L4 and L5 suggest some stability in the oxidized redox states, chemical oxidation experiments (see discussion below) show that they dimerize upon oxidation (Figure 5). The cyclic voltammetry (CV) curve recorded for L1 in CH_2_Cl_2_ shows a first oxidation wave at *E*_ox_ = 0.17 V, followed by oxidation waves at *E*_ox_ = 0.36 and 0.69 V, but no reversible redox events (Figure 4). Additional CV experiments in which the scan is reversed immediately after the first oxidation wave did not lead to reversible behavior. Presumably, the product rapidly reacts further upon oxidation (see discussion below). Quantum‐chemical calculations (B3LYP/def2‐TZVP) were carried out to further analyze the inherent redox properties of L1 and L4. The first ionization energy for L1 is 570 kJ mol^−1^, being slightly higher than the first ionization energy of L4 (563 kJ mol^−1^), in line with the trend in the *E*_ox_ values. The imposed conformation of the guanidino groups in L1 hampers charge delocalization in the radical monocationic redox state.

Chemical oxidation of L1 with two equivalents of ferrocenium hexafluorophosphate gave a mixture of products, among which only the monoprotonated ligand, (L1 + H)^+^, could be identified. Moreover, no isosbestic points were detected in a UV‐vis spectroscopic redox titration experiment, in which FcPF_6_ was added in portions to a solution of L1 (see Supporting Information for details). Oxidation of L1 leads to the formation of several products in a complex reaction sequence. We previously reported the intriguing redox chemistry of L5;^[^ ^20^ ^]^ oxidation of L5 initiates dimerization (Figure 5). Without doubt, the first step is the oxidation of the diguanidine with FcPF_6_ to the radical monocation.] Then, there are two possibilities. First, the monocation can dimerize directly to a dicationic dimer, that is subsequently oxidized with FcPF_6_ to the tetracation. Alternatively, the dication is formed in small amounts, together with the neutral diguanidine, in a disproportionation equilibrium from the radical monocation (Δ*G*_disp_ = 29.9 kJ mol^−1^ for L4 and 27.0 kJ mol^−1^ for L5 in CH_2_Cl_2_ from the experimental *E*_1/2_ values), and dimerizes to the tetracation. Other reported causes for dimerization, for example of phenoxazinone or *o*‐phenylenediamine, include photoactivation,^[^ ^31^ ^]^ enzymatic activation ^[^ ^32^, ^33^ ^]^ and metal‐induced reactions,^[^ ^34^, ^35^ ^]^ or the use of specific PCET reagents.^[^ ^36^ ^]^ Compound L4 reacts similar to L5 (see Supporting Information for details). The different reactivities of L4/L5 and L1 could be rationalized by the presence of the aliphatic bridge in L1, which destabilizes the oxidized redox state and prohibits the attack of the guanidino groups at the aromatic backbone. In new experiments, we made a surprising discovery. Reduction of the tetracationic dimeric oxidation product of L5 with cobaltocene (CoCp_2_) leads to 2,3‐diguanidino‐phenazine (Figure 5). In this reaction, the bridging guanidino groups formally lose two NHC's upon reduction. The 2,3‐diguanidino‐phenazine product could not be separated completely from by‐products, but it crystallized from the reaction mixture and was structurally characterized (Figure 5). To obtain this new diguanidine cleanly, we synthesized it starting with 2,3‐diamino‐phenazine (see Figure 5 and Supporting Information for details).

The ethylene bridge in compound L1 leads to an unfavorable conformation of the guanidino groups, causing the radical monocationic redox state of L1 to be less stable than that of L4 or L5. The reduced stability expresses itself in the higher *E*_ox_ value. Presumably, L1 quickly oligomerizes upon oxidation. Dimerization with formation of a new stable C_4_N_2_ ring, as observed for L4 and L5 (see Figure 5) is prohibited by the ethylene bridge and the conformational inflexibility of the two guanidino groups.

### 2.2 Propylene‐Bridged Ligands

#### 2.2.1 Synthesis and Characterization

**Figure 6:**
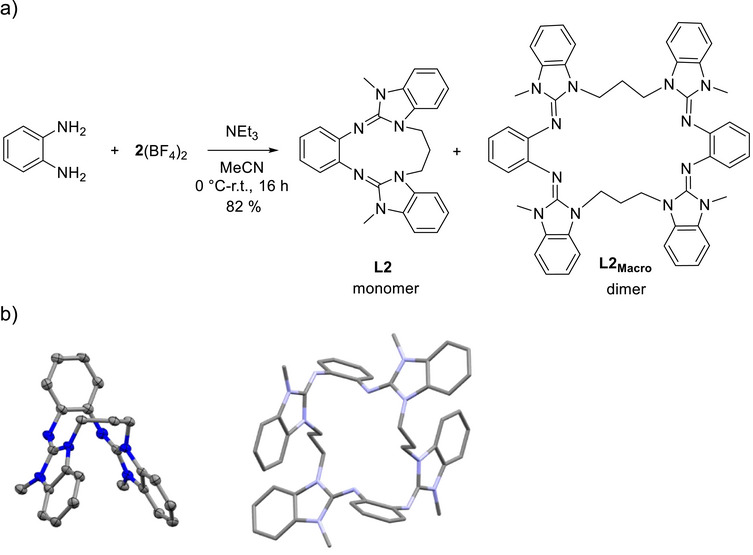
a) Synthesis of the cyclic diguanidine ligand L2 together with macrocyclic tetraguanidine L2_Macro_. b) Illustrations of the crystal structures of cyclic diguanidine L2 from SC‐XRD analysis and the macrocyclic tetraguanidine L2_Macro_ from quantum‐chemical calculations. Displacement ellipsoids drawn at the 50% probability level. C─H hydrogen atoms are omitted. Color code: C grey, N blue. Right: Structure calculated (with B3LYP/def2‐TZVP) for L2_Macro_.

Next, we tested the possibility to increase the alkyl chain connecting the two guanidino groups. Cyclic L2 with a propylene bridge was prepared according to Figure 6. However, the product turned out to contain not only the cyclic diguanidine L2, but also its macrocyclic tetraguanidine dimer L2_Macro_ in a variable L2:L2_Macro_ ratio of approximately 1.5:1–1:1.5 (estimated from the methyl proton signal in the ^1^H NMR spectra (see Supporting Information)). It was not possible to isolate cyclic diguanidine L2 or macrocyclic L2_Macro_ directly from this mixture, due to their similar solubility in organic solvents. Column chromatography on silica or aluminum oxide leads to the decomposition of the guanidine ligands. Fortunately, the neutral cyclic diguanidine crystallized from the reaction mixture and was structurally characterized by SC‐XRD, while the structure of L2_Macro_ was calculated (see Figure 6b and Supporting Information for details). Moreover, we obtained crystal structures for the mono‐ and di‐protonated compounds (L2 + H)^+^ and (L2 + 2H)^2+^. In L2_Macro_, the angle between the two guanidino groups (calculated α = 126.01°) is significantly larger than in the monomer L2 (calculated α = 57.1° for the isolated molecule, experimentally derived α = 64.82° in the solid state), highlighting the constraint imposed by the propylene bridge.

Interestingly, for the ethylene‐bridged compound L1, the formation of macrocyclic tetraguanidine L1_Macro_ was not observed. To clarify this issue, we carried out DFT calculations with the B3LYP functional and the def2‐TZVP basis set on L1, L2, L2_Macro_, and the hypothetical molecule L1_Macro_ and determined the thermodynamical values Δ*H*, Δ*S*, and Δ*G* for the dimerization of the cyclic diguanidines into macrocyclic tetraguanidines (Table 1). All calculations were carried out without inclusion of the solvent effect to obtain information about the inherent properties of the compounds. Both dimerization reactions are exothermic, but the Δ*H* value is significantly more negative for L2 than for L1. Due to the positive entropy of dimerization, both dimerizations are endergonic. However, the Gibbs free energy for dimerization of L2 is low (+10.2 kJ mol^−1^), in line with the observation of the formation of both L2 and L2_Macro_ in almost equal quantities. By contrast, the dimerization of L1 to L1_Macro_ is endergonic by + 42.2 kJ mol^−1^. This high value offers an explanation for the failure to detect L1_Macro_ in the experiments.

**Table 1:** Thermodynamic data Δ*H*, Δ*S*, and Δ*G* for the dimerization reactions of the cyclic diguanidines L1 and L2 (from B3LYP/def2‐TZVP calculations).

Reaction	Δ*H* / kJ mol^−1^	Δ*S* / J K^−1^mol^−1^	Δ*G* / kJ mol^−1^
2 L1 → L1_Macro_	−17.1	+199	+42.2
2 L2 → L2_Macro_	−41.8	+175	+10.2

#### 2.2.2 Coordination Chemistry

**Figure 7:**
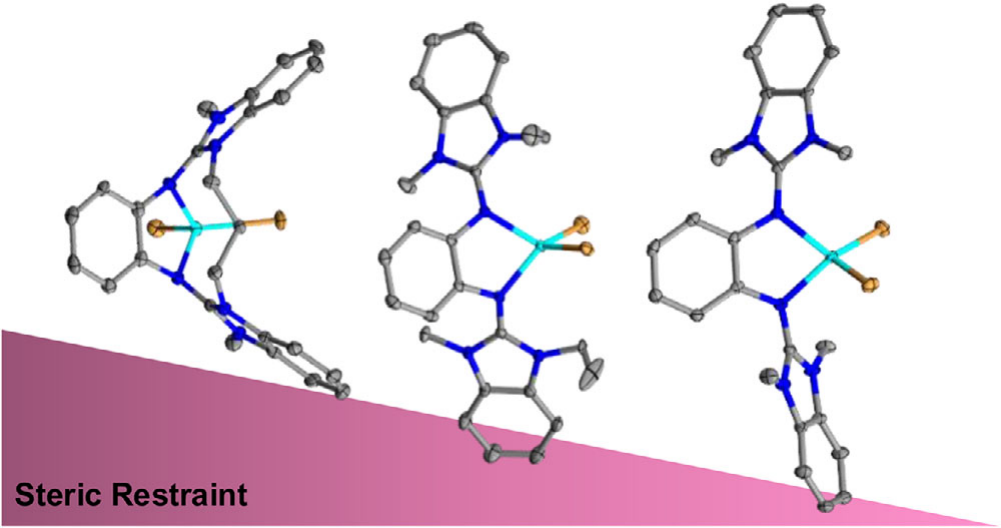
Illustration of the molecular structures for [CoBr_2_(L2)], [CoBr_2_(L4)], and [CoBr_2_(L5)]. Displacement ellipsoids drawn at the 50% probability level. Hydrogen atoms are omitted. Color code: C grey, N blue, Co light blue, Br brown.

In contrast to L1, L2 readily forms coordination compounds. Several transition metal complexes were prepared, including [NiCl_2_(L2)], [CuCl_2_(L2)], and [CuBr_2_(L2)], as well as [Cu(L2)_2_](BF_4_)_2_ (see Supporting Information for details). The following discussion focusses on coordination compounds for which a structural characterization by SC‐XRD analysis was accomplished for different diguanidine ligands. Reaction of the mixture of L2 and L2_Macro_ with CoBr_2_ in CH_2_Cl_2_ at room temperature gave a complex with the stochiometric composition [CoBr_2_(L2)]. After workup, the product was obtained in a yield of 82%. Crystals of the mononuclear complex were grown from a saturated dichloromethane solution through slow solvent evaporation (Figure 7), indicating that the monomer complex is the main product, while both species [CoBr_2_(L2)] and [(CoBr_2_)_2_(L2_Macro_)] coexist. The structural properties of this complex can be compared to the respective complexes of the open ligands L4 and L5 (see Table 2 and Figure 7), which were synthesized for comparison. From the Co─N and Co─Br distances, it is clear that all compounds are high‐spin Co^II^ complexes, in line with the weak ligand‐field induced generally by guanidine ligands, due to significant π‐donor bond contributions.^[^ ^37^, ^38^, ^39^, ^40^ ^]^

**Table 2:** Selected bond lengths and angles of [CoBr_2_(L2)], [CoBr_2_(L4)], and [CoBr_2_(L5)], experimentally derived from SC‐XRD measurements.

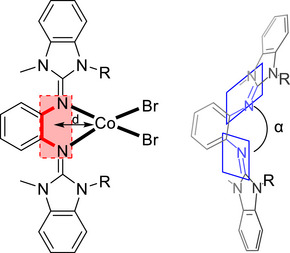		
Complex	Parameter	Distance [Å] or Angle [°]
[CoBr_2_(L2)]	Co─N	2.020(4)/2.023(4)
	Co─Br	2.3699(8)/ 2.3746(7)
	*d*(N─C─C‐N) plane‐Co	0.640
	α	80.86
[CoBr_2_(L4)]	Co─N	2.015(2)/2.010(2)
	Co─Br	2.3907(4)/ 2.3916(5)
	*d*(N─C─C─N) plane‐Co	0.645
	α	125.97
[CoBr_2_(L5)]	Co─N	2.003(3)/2.006(3)
	Co─Br	2.4007(6)/ 2.3821(6)
	*d*(N─C─C─N) plane‐Co	0.398
	α	156.49

The structural parameters indicate that the steric restraint of the coordination to the imine nitrogen of the guanidines decreases in the row L2 >> L4 > L5. The dihedral angle α between the guanidino units increases in the row [CoBr_2_(L2)] (80.86°) < [CoBr_2_(L4)] (125.97°) < [CoBr_2_(L5)] (156.49°). The least sterically hindered complex [CoBr_2_(L5)] also exhibits the shortest Co─N bonds. Furthermore, for all complexes, the cobalt atom is displaced from the central C_6_ ring plane, but this displacement is much smaller for [CoBr_2_(L5)] (0.398 Å) than for the complexes of L2 and L4 (0.640 Å for [CoBr_2_(L2)] and 0.645 Å for [CoBr_2_(L4)]). Although the structures show some differences, the UV‐vis spectra are quite similar. In the visible region, three bands due to d‐d transitions are visible at 568, 630, and 688 nm for [CoBr_2_(L5)], and at 570, 632, and 691 nm for [CoBr_2_(L4)]. The same bands can also be observed for the complex of cyclic diguanidine L2, [CoBr_2_(L2)], at 572, 637, and 685 nm. The electrochemical properties of the complexes show the expected trend. In the cyclic voltammogram recorded for [CoBr_2_(L4)], a reversible redox process is visible at *E*_1/2_ = 0.18 V (*E*_ox_  =  0.25 V). In analogy, a wave at *E*_1/2_ = 0.21 V (*E*_ox_ = 0.29 V) is measured for [CoBr_2_(L5)] and a wave at *E*_1/2 _= 0.25 (*E*_ox _= 0.30 V) for [CoBr_2_(L2)]. On the basis of previous work on cobalt complexes,^[^ ^41^ ^]^ the first oxidation wave is tentatively assigned to ligand‐centered oxidation. In line with the potentials measured for the uncoordinated ligands, the complex with cyclic diguanidine L2 has a higher potential than the complexes with the open diguanidines L4/L5.

**Figure 8:**
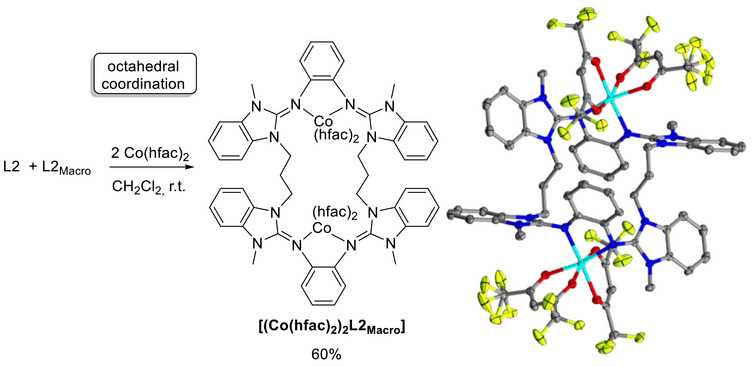
Reaction scheme for the synthesis of [{Co(hfac)_2_}_2_(L2_Macro_)], together with an illustration of the solid‐state structure of the dinuclear cobalt complex. Displacement ellipsoids drawn at the 50% probability level. Hydrogen atoms are omitted. Color code: C grey, N blue, Co light blue, O red, F yellow.

Interestingly, coordination could be used to quantitatively separate the dimer L2_Macro_ from L2. Reaction with Co(hfac)_2_ (hfac = hexafluoro‐acetylacetonate) gave [{Co(hfac)_2_}_2_(L2_Macro_)] in a maximum yield of 60% (Figure 8). Crystals of [{Co(hfac)_2_}_2_(L2_Macro_)] were grown from a concentrated dichloromethane solution at −18 °C. Hence, only the dinuclear complex with L2_Macro_ is formed and crystallizes. Most likely, the sterically demanding hfac coligands prohibit complex formation with the cyclic diguanidine L2 (Figure 8).

**Figure 9:**
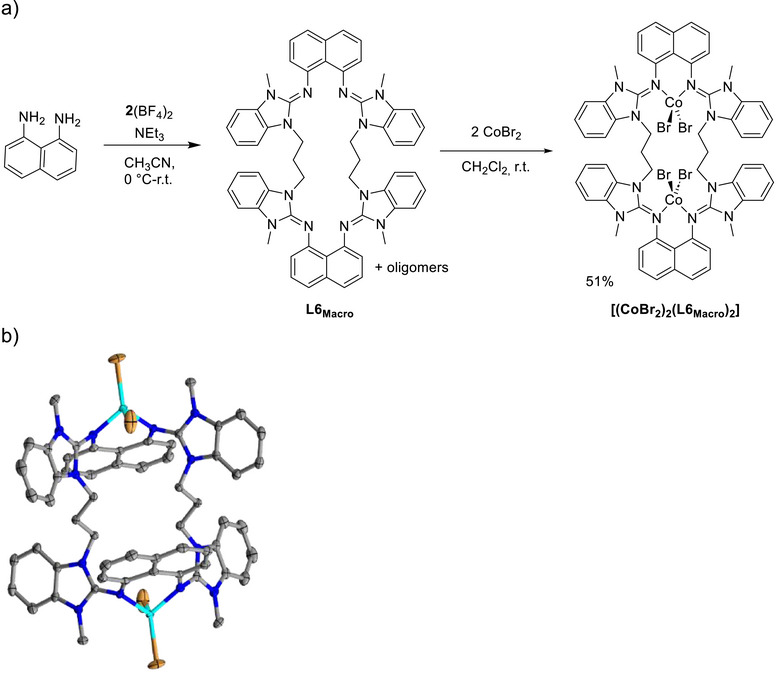
a) Scheme for the formation of the macrocyclic tetraguanidine L6_Macro_ and its use for the preparation of a dinuclear cobalt complex. b) Illustration of the solid state structure of the dinuclear cobalt complex [(CoBr_2_)_2_(L6_Macro_)]. Displacement ellipsoids drawn at the 50% probability level. C─H hydrogen atoms are omitted. Color code: C grey, N blue, Co light blue, Br brown.

#### 2.2.3 Further System With Propylene‐bridged Guanidino Groups

Finally, we reacted **2**(BF_4_)_2_ with 1,8‐diamino‐naphthalene. A preferred formation of the macrocyclic tetraguanidine instead of the small cycle is expected, since the amino groups are further apart and the C─N bonds to the aromatic C atoms oriented parallel to each other. A mixture of several oligomers (presumably open oligoguanidine chains) was observed next to the macrocyclic tetraguanidine, that could not be separated from one another. On the other hand, the addition of CoBr_2_ to the mixture leads selectively to the crystallization of the dinuclear complex with the macrocyclic tetraguanidine, [(CoBr_2_)_2_(L6_Macro_)], in a yield of 51% (Figure 9). The cobalt atoms are displaced by 0.621 Å from the naphthalene plane. However, in this case already metal complexes of open 1,8‐diguanidino‐naphthalene ligands show large displacements of the metal, arising from the distance and relative orientation of the lone pairs on the imino N donor atoms.^[^ ^42^, ^43^ ^]^

### 2.3 Isobutylene‐Bridged Ligand

#### 2.3.1 Synthesis and Characterization

**Figure 10:**
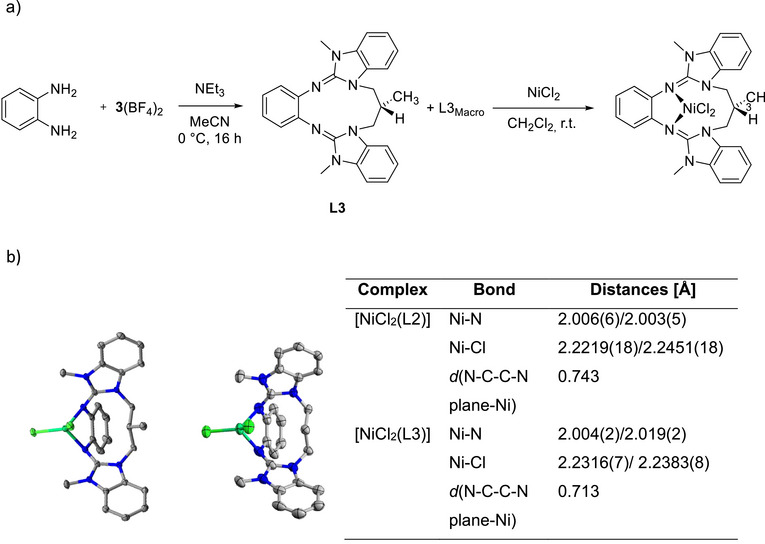
a) Synthesis of L3, and reaction with NiCl_2_ to give the complex [NiCl_2_(L3)]. b) Solid state structures of [NiCl_2_(L3)] and [NiCl_2_(L2)] and selected structural parameters. Color code: C grey, N blue, Ni light green, Cl dark green.

In further experiments, a methyl group was introduced at the central C atom of the propylene bridge to test its effect on the steric restraint. The reaction between *o*‐phenylenediamine and **3**(BF_4_)_2_ afforded a mixture of cyclic diguanidine L3 and its macrocyclic dimer L3_Macro_ (Figure 10a). However, in difference to the reaction leading to L2, the L3:L3_Macro_ ratio shifts to approximately 10:1 (estimated from ^1^H NMR spectroscopy), possibly due to a favorable preorientation of the benzimidazolium units induced by the methyl group. It was not possible to grow crystals of L3, but the twofold protonated form crystallized together with two chloride counterions (see Supporting Information for details). In the solid‐state structure, the methyl group points in the opposite direction to the imino N atoms, indicating that the methyl group does not lead to a large steric restraint.

### 2.4 Coordination Chemistry

Since the methyl group points away from the imino N atoms, its presence should not significantly hamper metal coordination. Indeed, reaction of L3 with NiCl_2_ furnished the complex [NiCl_2_(L3)] with tetrahedral metal coordination in 85% yield (Figure 10). Further complexes [CuCl_2_(L3)] and [CuBr_2_(L3)] were synthesized by reaction with CuCl_2_ and CuBr_2_, as well as the homoleptic complex [Cu(L3)_2_](BF_4_)_2_ with the copper salt Cu(BF_4_)_2_ (see Supporting Information for details), confirming that cyclic L3 could bind to transition metals. In solid [NiCl_2_(L3)], the metal is displaced from the central C_6_ ring plane by 0.713 Å, in comparison to only 0.398 Å in [CoBr_2_(L5)]. To allow for a more direct comparison, the analog complex [NiCl_2_(L2)] was synthesized (see Supporting Information for details), and the structural parameters of the complexes of L2 and L3 in the solid‐state were compared (Figure 10). As could be seen from Figure 10, both complexes have similar structural parameters. The Ni─N bonds vary only slightly. The additional methyl group in [NiCl_2_(L3)] shifts the nickel atom 0.03 Å closer to the aromatic NCCN plane (0.713 Å in [NiCl_2_(L3)] and 0.743 Å [NiCl_2_(L2)]). The Ni─Cl bonds in [NiCl_2_(L3)] are slightly longer than in [NiCl_2_(L2)]. These results confirm that metal coordination is not significantly hampered by the presence of the methyl group.

### 2.5 Quantum‐Chemical Calculations on the Coordination Properties of L1–L3

**Scheme 3:**
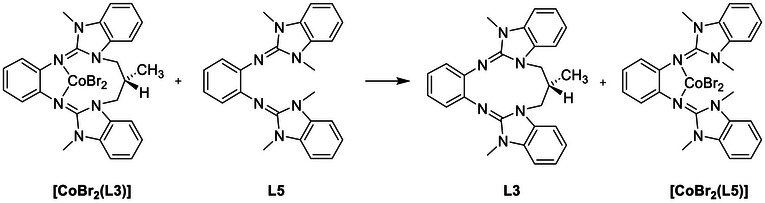
Example for a ligand substitution reaction.

Quantum‐chemical calculations were carried out (B3LYP/def2‐TZVP) to compare the ability of the three new cyclic diguanidine ligands L1‐L3 to form stable complexes. The coordination to cobalt dibromide was used for this purpose, because experimental structures for validation of the calculations are now available for a complex with a cyclic diguanidine, [CoBr_2_(L2)] as well as a complex with an open diguanidine, [CoBr_2_(L5)]. As already mentioned, all complexes contain high‐spin Co^II^ atoms (S = 3/2). The Gibbs free energy and enthalpy changes, Δ*G* and Δ*H*, were determined for ligand exchange reactions between the cobalt dibromide complex of one of the cyclic diguanidines and the open diguanidine ligand L5. The reaction [CoBr_2_(L3)] + L5 → [CoBr_2_(L5)] + L3 (Scheme 3) is mildly endothermic, but exergonic (Δ*G* = −3 kJ mol^−1^, Δ*H* = 3 kJ mol^−1^). The substitution of L2 in the analog complex [CoBr_2_(L2)] by L5 is weakly exothermic and exergonic (Δ*H* = −4 kJ mol^−1^, Δ*G* = −8 kJ mol^−1^). Thus, the calculations confirm the similar coordination behavior of complexes of L2 and L3. In the case of L1, the calculations found a minimum‐energy structure for a complex [CoBr_2_(L1)], although complex formation was not observed in the experiments. However, the thermodynamics show, that the enthalpy and Gibbs free energy change for substitution of cyclic diguanidine L1 in [CoBr_2_(L1)] with the open diguanidine L5 are highly negative (Δ*G* = −51 kJ mol^−1^, Δ*H* = −47 kJ mol^−1^). This implies a weak metal‐ligand bond in [CoBr_2_(L1)] compared to [CoBr_2_(L5)], explaining why no complex formation with L1 is observed in our experiments (Table 3).

To highlight the steric restraint in [CoBr_2_(L1)], selected calculated structural parameters are compared with the complexes [CoBr_2_(L2)], [CoBr_2_(L3)], and [CoBr_2_(L5)] in Table 4. It can be seen that the calculated structural data are quite close to the experimental ones for [CoBr_2_(L2)] and [CoBr_2_(L5)]. The Co─N bond lengths, calculated without inclusion of environmental effects, are slightly larger than the experimentally derived values for the compounds in the solid state (by a factor of 1.026 for [CoBr_2_(L2)] and 1.023 for [CoBr_2_(L5)]). The slightly shorter bonds in the experimentally obtained structures most likely result from intermolecular interactions in the solid state. The displacement of the metal from the central aromatic plane clearly increases with the steric restraint. It is minimal for the complex of the open diguanidine (0.128 Å) and reaches its maximum in the complex with the cyclic diguanidine L1 (1.072 Å). The dihedral angle between the guanidino groups (α), measures only 56.71° in [CoBr_2_(L1)], arguing for strong steric restraint, and increases to 149.04° in [CoBr_2_(L5)]. Also, the complex [CoBr_2_(L1)] exhibits the longest Co─N bond (2.123/2.158 Å), while [CoBr_2_(L5)] contains the shortest C─N bond (2.048/2.052 Å). The analysis of the experimental and calculated structural data that clearly shows that the steric restraint becomes apparent in three parameters, namely the metal‐N bond lengths, the displacement of the metal from the central ligand aromatic plane, and the dihedral angle between the two guanidino groups.

**Table 3:** Thermodynamic data Δ*G* and Δ*H* (B3LYP/def2‐TZVP) for the ligand exchange reactions.

Reaction	Δ*H* / kJ mol^−1^	Δ*G* / kJ mol^−1^
[CoBr_2_(L3)] + L5→ [CoBr_2_(L5)] + L3	3	−3
[CoBr_2_(L2)] + L5 → [CoBr_2_(L5)] + L2	−4	−8
[CoBr_2_(L1)] + L5→ [CoBr_2_(L5)] + L1	−47	−51

**Table 4:** Structural parameters of the calculated complexes.

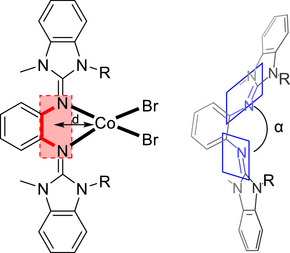		
Complex	Parameter	Distance [Å] or Angle [°]
[CoBr_2_(L1)]	Co─N	2.123/2.158
	Co─Br	2.346/ 2.417
	*d*(N─C─C─N) plane‐Co	1.072
	α	56.71
[CoBr_2_(L2)]	Co─N	2.073/2.075
	Co─Br	2.359/ 2.423
	*d*(N─C─C─N) plane‐Co	0.667
	α	85.34
[CoBr_2_(L3)]	Co─N	2.075/2.076
	Co─Br	2.426/ 2.358
	*d*(N─C─C─N) plane‐Co	0.663
	α	86.29
[CoBr_2_(L5)]	Co─N	2.048/2.052
	Co─Br	2.399/2.397
	*d*(N─C─C─N) plane‐Co	0.128
	α	149.04

The differences in the coordination ability of the cyclic diguanidine ligand L1 compared to the L2 and L3 ligands clearly could be explained by the differences in the steric restraints. The steric restraint also leads to different electronic properties of the ligands. The HOMOs of all three ligands L1‐L3 are mainly composed of the lone‐pair orbitals at the imino N atoms and a π orbital of the benzene core. However, the HOMO energy (calculated with B3LYP/def2‐TZVP) is much lower for L1 (−4.994 eV) than for L2 (−4.225 eV) and L3 (−4.122 eV). Therefore, L1 is a weaker donor than L2 and L3.

## 3 Conclusions

The design of chelating ligands with steric restraint is of high interest for a variety of applications in coordination chemistry. In this work, novel redox‐active *o*‐diguanidino‐benzene derivatives are reported, in which the guanidino groups are not only connected through the aromatic core, but in addition by an alkyl chain linker between the amino moieties within the two guanidino groups. The results show that the size and shape of the linker significantly influence the coordination chemistry. An ethylene bridge prohibits entirely any metal coordination, leading to noncoordinating, but still highly Brønsted basic molecules that could be used as substitutes for Huenig's base in synthetic chemistry. The change from an ethylene bridge to a propylene or isobutylene bridge switches back on the coordination ability, as shown by the synthesis of a variety of complexes. The synthesis of these cyclic diguanidines does not lead only to diguanidines, but also to macrocyclic tetraguanidines, that form dinuclear complexes. Complexation could be used to separate the macrocyclic tetraguanidine from the cyclic diguanidine. Oxidation of the cyclic diguanidines leads to product mixtures, while oxidation of the open diguanidines initiates dimerization. Interestingly, reduction of the dimerization products yields diguanidino‐phenazines.

The structures of metal complexes of the cyclic diguanidines are compared with analog complexes of open diguanidines, and the analysis is complemented by quantum‐chemical calculations. Thereby, the steric restraint induced by the alkyl bridge is systematically evaluated. Three structural parameters were identified that most adequately describe the degree of steric restraint in the complexes.

The synthetic pathway to cyclic diguanidines and macrocyclic tetraguanidines developed in this work will be used in future work to introduce different bridges between the guanidino groups. The bridge could not only be used to vary the steric restraint but also allows the introduction of functional groups (e.g., additional donor atoms or optically‐active units).

## 4 Experimental Details

The synthesis details and analytical data for all compounds and information about the quantum‐chemical calculations are included in the Supporting Information. Deposition Numbers “2477529” for L4, 2477530 for (L4 + 2H)Cl_2_, 2477531 for (L4 + H)PF_6_, 2477532 for [Cu(BF_4_)_2_(L5)_2_], 2477533 for [CoBr_2_(L2)], 2477534 for [Ni_3_Cl_6_(L4)_3_], 2477535 for [Co_2_(hfac)_4_(L2_Macro_)], 2477536 for (L1 + H)OTf, 2477537 for (L2 + H)BF_4_, 2477538 for [Co_2_Br_4_(L6_Macro_)], 2477539 for [CoBr_2_(L5)], 2477540 for [CoBr_2_(L4)], 2477541 for *o*‐Diguanidinophenazine, 2477542 for [NiCl_2_(L2)], 2477543 for [NiCl_2_(L3)], 2477544 for L2, 2477545 for L1, 2477546 for (L1 + 2H)Cl_2_, 2477547 for (L2 + 2H)Cl_2_, 2477548 for (L1 + H)PF_6_ 2477549 for (L3 + 2H)Cl_2_, and 2477550 for [Cu(BF_4_)_2_(L4)_2_] contain the supplementary crystallographic data for this paper. These data are provided free of charge by the joint Cambridge Crystallographic Data Centre and Fachinformationszentrum Karlsruhe“http://www.ccdc.cam.ac.uk/structures” Access Structures service.

## Supporting Information

The authors have cited additional references within the Supporting Information.^[^ ^44^, ^45^, ^46^, ^47^, ^48^, ^49^, ^50^, ^51^, ^52^, ^53^, ^54^, ^55^, ^56^, ^57^, ^58^, ^59^, ^60^ ^]^

## Conflict of Interest

The authors declare no conflict of interest.

## Supporting information



Supporting Information

Supporting Information

## References

[1] T. L. Brown , K. J. Lee , Coord. Chem. Rev. 1993, 128, 89.

[2] C. A. Tolman , Chem. Rev. 1977, 77, 313.

[3] C. A. Tolman , J. Am. Chem. Soc. 1970, 92, 2956.

[4] C. A. Tolman , W. C. Seidel , L. W. Gosser , J. Am. Chem. Soc. 1974, 96, 53.

[5] A. Brand , W. Uhl , Chem. Eur. J. 2019, 25, 1391. 30126018 10.1002/chem.201803331

[6] S. Würtz , F. Glorius , Acc. Chem. Res. 2008, 41, 1523. 18720995 10.1021/ar8000876

[7] M. N. Hopkinson , C. Richter , M. Schedler , F. Glorius , Nature 2014, 510, 485. 24965649 10.1038/nature13384

[8] <number>[8]number> S. Roland , J. M. Suarez , M. Sollogoub , Chem. Eur J. 2018, 24, 12464 . 29617045 10.1002/chem.201801278

[9] Z.‐Z. Zhu , C.‐B. Tian , Q.‐F. Sun , Chem. Rec. 2021, 21, 498. 33270374 10.1002/tcr.202000130

[10] E. G. Percástegui , T. K. Ronson , J. R. Nitschke , Chem. Rev. 2020, 120, 13480. 33238092 10.1021/acs.chemrev.0c00672 PMC7760102

[11] S. R. Seidel , P. J. Stang , Acc. Chem. Res. 2002, 35, 972. 12437322 10.1021/ar010142d

[12] R. Saha , B. Mondal , P. S. Mukherjee , Chem. Rev. 2022, 122, 12244. 35438968 10.1021/acs.chemrev.1c00811

[13] I. Sinha , P. S. Mukherjee , Inorg. Chem. 2018, 57, 4205. 29578701 10.1021/acs.inorgchem.7b03067

[14] E. Benchimol , B.‐N. T. Nguyen , T. K. Ronson , J. R. Nitschke , Chem. Soc. Rev. 2022, 51, 5101. 35661155 10.1039/d0cs00801j PMC9207707

[15] H.‐J. Himmel , Chapter 7 (pp. 199–248) in Redox‐Active Ligands: Concepts and Catalysis, Ed. M. Desage‐El Murr , Ed., Wiley 2024, ISBN: 978‐3‐527‐34850‐3;

[16] H.‐J. Himmel , Synlett 2018, 29, 1957;

[17] H.‐J. Himmel , Inorg. Chim. Acta 2018, 481, 56.

[18] J. Osterbrink , P. Walter , S. Leingang , H. Pfisterer , E. Kaifer , H.‐J. Himmel , Chem. Eur. J. 2023, 29, e202300514. 36924243 10.1002/chem.202300514

[19] L. Lohmeyer , F. Schön , E. Kaifer , H.‐J. Himmel , Angew. Chem. Int. Ed. 2021, 60, 10415. 10.1002/anie.202101423 PMC8252010 33616266

[20] U. Wild , E. Engels , O. Hübner , E. Kaifer , H.‐J. Himmel , Chem. Eur. J. 2024, 30, e202403080 (1–14). 39387154 10.1002/chem.202403080

[21] B. Kovačević , Z. B. Maksić , Org. Lett. 2001, 3, 1523; 11388857 10.1021/ol0158415

[22] B. Kovačević , Z. B. Maksić , R. Vianello , M. Primorac , New J. Chem. 2002, 26, 1329‒1334.

[23] A. Peters , H. Herrmann , M. Magg , E. Kaifer , H.‐J. Himmel , Eur. J. Inorg. Chem. 2012 pp. 1620.

[24] A. Peters , U. Wild , O. Hübner , E. Kaifer , H.‐J. Himmel , Chem. Eur. J. 2008, 14, 7813. 18637648 10.1002/chem.200800244

[25] H.‐J. Himmel , Synlett 2018, 29, 1957.

[26] S. D. Lepore , A. Khoram , D. C. Bromfield , P. Cohn , V. Jairaj , M. A. Silvestri , J. Org. Chem. 2005, 70, 7443. 16122274 10.1021/jo051040u

[27] S. Hünig , M. Kiessel , Chem. Ber. 1958, 91, 380.

[28] J. L. Moore , S. M. Taylor , V. A. Soloshonok , Arkivoc. 2005 (part vi): 287.

[29] S. L. Buchwald , E. J. Hennesy , Org. Lett. 2002, 4, 269. 11796067 10.1021/ol017038g

[30] S. Wiesner , A. Wagner , O. Hübner , E. Kaifer , H.‐J. Himmel , Chem. Eur. J. 2015, 21, 16494. 26418042 10.1002/chem.201502584

[31] A. K. Dhara , S. Maity , B. B. Dhar , Org. Lett. 2021, 23, 3269–3273. 33880922 10.1021/acs.orglett.1c00725

[32] S. Fornera , P. Walde , Anal. Biochem. 2010, 407, 293. 20692226 10.1016/j.ab.2010.07.034

[33] H. Liu , Z. Wang , Y. Liu , J. Xiao , C. Wang , Thermochim. Acta 2006, 443, 173.

[34] A. Panja , P. Guionneaua , Dalton Trans. 2013, 42, 5068. 23396321 10.1039/c3dt32788d

[35] A. K. Dhara , U. P. Singh , K. Ghosh , Inorg. Chem. Front. 2016, 3, 1543.

[36] U. Wild , O. Hübner , H.‐J. Himmel , Chem. Eur. J. 2019, 25, 15988. 31535741 10.1002/chem.201903438 PMC7065378

[37] B. Kintzel , M. Fittipaldi , M. Böhme , A. Cini , L. Tesi , A. Buchholz , R. Sessoli , W. Plass , Angew. Chem. Int. Ed. 2021, 60, 8832. 10.1002/anie.202017116 PMC8048656 33511751

[38] D. Plaul , M. Böhme , S. Ostrovsky , Z. Tomkowicz , H. Görls , W. Haase , W. Plass , Inorg. Chem. 2018, 57, 106. 29227093 10.1021/acs.inorgchem.7b02229

[39] J. E. Allen , W. S. Kassel , N. A. Piro , Polyhedron 2018, 155, 77.

[40] P. Roquette , A. Maronna , A. Peters , E. Kaifer , H.‐J. Himmel , C. Hauf , V. Herz , E.‐W. Scheidt , W. Scherer , Chem. Eur. J. 2010, 16, 1336. 19967729 10.1002/chem.200901479

[41] L. Lohmeyer , E. Kaifer , M. Enders , H.‐J. Himmel , Chem. Eur. J. 2021, 27, 11852. 34101917 10.1002/chem.202101364 PMC8457109

[42] U. Wild , O. Hübner , A. Maronna , M. Enders , E. Kaifer , H. Wadepohl , H.‐J. Himmel , Eur. J. Inorg. Chem. 2008, 4440;

[43] V. Vitske , C. König , O. Hübner , E. Kaifer , H.‐J. Himmel , Eur. J. Inorg. Chem. 2010, 115.

[44] U. Jahn , P. Hartmann , I. Dix , P. G. Jones , J. Org. Chem. 2001, 17, 3333.

[45] SAINT (APEX III/IV) Bruker AXS GmbH, Karlsruhe, Germany 2016/2021.

[46] G. M. Sheldrick , SADABS, Bruker AXS GmbH, Karlsruhe, Germany 2004–2014;

[47] L. Krause , R. Herbst‐Irmer , G. M. Sheldrick , D. Stalke , J. Appl. Cryst. 2015, 48, 3. 26089746 10.1107/S1600576714022985 PMC4453166

[48] G. M. Sheldrick , SHELXT, Program for Crystal Structure Solution, University of Göttingen, Germany 2014–2018;

[49] G. M. Sheldrick , Acta Cryst 2015, C71, 3.

[50] G. M. Sheldrick , SHELXL‐20xx, University of Göttingen and Bruker AXS GmbH, Karlsruhe, Germany 2012–2018;

[51] W. Robinson , G. M. Sheldrick , in: N. W. Isaaks , M. R. Taylor , Eds. ”Crystallographic Computing 4“, Ch. 22, IUCr and Oxford University Press, Oxford, UK, 1988;

[52] G. M. Sheldrick , ActaCryst. 2008, A64, 112.

[53] O. V. Dolomanov , L. J. Bourhis , R. J. Gildea , J. A. K. Howard , H. Puschmann , J. Appl. Cryst. 2009, 42, 339. 10.1107/S0021889811041161 PMC3236671 22199401

[54] A. Thorn , B. Dittrich , G. M. Sheldrick , Acta Cryst. 2012, A68, 448.

[55] P. v. d. Sluis , A. L. Spek , Acta Cryst 1990, A46, 194;

[56] A. L. Spek , Acta Cryst. 2015, C71, 9.

[57] A. L. Spek , PLATON, Utrecht University, The Netherlands;

[58] A. L. Spek , J. Appl. Cryst. 2003, 36, 7.

[59] M. Werr , Dissertation, Ruprecht‐Karls‐Universität Heidelberg, Heidelberg, 2022.

[60] V. P. W. Böhm , W. A. Herrmann , Angew. Chem. Int. Ed. 2000, 39, 4036. 10.1002/1521-3773(20001117)39:22<4036::aid-anie4036>3.0.co;2-l 11093196

