# Supplementary material for: Steric Restraints in Redox‐Active Guanidine Ligands and Their Impact on Coordination Chemistry
Source: Chemistry. 2025 Oct 25;31(66):e02457. doi: 10.1002/chem.202502457 (PMC12648461; doi:10.1002/chem.202502457)
Supplement: Supplementary file 1 — Supporting Information [file CHEM-31-e02457-s001.pdf]

Supporting Information

Table of contents

1. General experimental details 2

2. Synthesis protocols and analytical data of the activated urea species 4

3. Synthesis protocols, analytical data and quantum chemical calculations of the ligands 18

4. Synthesis protocols, analytic data and quantum chemical calculations of the complexes.. 66

5. Crystal data 109

1. General experimental details

All synthetic work was carried out using standard Schlenk techniques under argon atmosphere. The solvents acetonitrile (stored oved CaH_2), dichloromethane, diethylether, toluene and tetrahydrofuran were dried with a MBraun MB-SPS-800 Solvent Purification System and stored over molecular sieves. Other solvents were purchased from Acros Organics. The following chemicals were purchased and used as delivered: *o*-phenylenediamine (98 %, Alfa Aesar), 2-Chlor-1*H*-benzimidazole (BLD Pharm), 1,2-Dibromoethane (Sigma-Aldrich), 1,3-Dibromopropane (ABCR), 1,3-Dibromo-2-methylpropane (BLD Pharm), hydrochloride solution (2.0 M in diethylether, Sigma-Aldrich), triethylamine (Sigma-Aldrich). The synthesis of ferrocenium hexafluorophosphate[1] followed the literature procedure. Elemental analyses were performed at the Microanalytical Laboratory of Heidelberg University using the vario EL and vario MICRO cube devices from Elementar Analysensysteme GmbH. Please note that the compounds are strong Brønsted bases and redox-active. Therefore, some deviations are caused by reactions with traces of water or dioxygen during the elemental analysis measurements. NMR spectra were recorded on a Bruker Avance II 400, Bruker AVANCE III 600 or Bruker 600 Ultrashield system. Solvent resonances were taken as references for all 1H NMR spectra. UV-vis spectra were recorded with a Cary 5000 spectrophotometer. CV measurements were carried out with a Metrohm Autolab PGSTAT 204 potentiostate/galvanostate and an Ag/AgCl reference electrode, Pt rod counter electrode and glassy carbon working electrode. All voltammograms were recorded at room temperature. CH_2Cl_2 and CH_3CN were used as solvents for the individual compounds (concentration around 10^{-3} M, if not stated otherwise), whereas nBu$_4$N(PF$_6$) (electrochemical grade (≥99.0%), Fluka) was employed as supporting electrolyte (c = 0.1 M). The potentials are given vs. the redox reference pair ferrocenium/ferrocene (Fc^+/Fc), measured at $E_{1/2}$ = 0.43 V in CH_3CN and 0.47 V in CH_2Cl_2. HR-ESI spectra were recorded with a Bruker ApexQe hybrid 9.4 T FT-ICR spectrometer and HR-EI spectra with a JEOL AccuTOF GCx. Infrared spectra were recorded as solids on an ATR crystal with an AGILENT Cary 630 FTIR spectrometer. EPR measurements were recorded on a magnettech MiniScope MS400 spectrometer at room temperature in the X-band frequency range (9.30-9.55 GHz) with a modulation frequency of 100 kHz.

<u>X-ray crystallography</u>

Suitable crystals for single-crystal structure determination were taken directly from the mother liquor, taken up in per-fluorinated polyether oil and fixed on a cryo loop. Full shells of intensity data were collected at low temperature with a Bruker D8 Venture, dual source (Mo-Kα radiation, microfocus X-ray tube, Photon III detector). Data were processed with the standard Bruker (SAINT, APEX3/4) software package.[i2] Multiscan absorption correction was applied using the SADABS program.[3i] The structures were solved by intrinsic phasing[4] and refined using the SHELXTL software package (Version 2018/3).[5] Graphical handling of the structural data during solution and refinement were performed with OLEX2.[6] All non-hydrogen atoms were given anisotropic displacement parameters. Hydrogen atoms bound to carbon were input at calculated positions and refined with a riding model. Hydrogen atoms bound to nitrogen were located in difference Fourier syntheses and refined, either fully or with appropriate distance and/or symmetry. Split atom models were used to refine disordered groups and/or solvent molecules. When found necessary, suitable geometry and adp restraints were applied.[6,7] Due to severe disorder and fractional occupancy, electron density attributed to the

solvent of crystallization was removed from some of the structures with the BYPASS procedure,[8] as implemented in PLATON (squeeze/hybrid).[9]

Deposition Numbers 2477529 for L4, 2477530 for (L4+2H)Cl_2, 2477531 for (L4+H)PF_6, 2477532 for [Cu(BF_4)$_2$(L5)$_2$], 2477533 for [$CoBr_2$(L2)], 2477534 for [Ni_3Cl_6(L4)$_3$], 2477535 for [Co_2(hfac)$_4$(L2$_{Macro}$)], 2477536 for (L1+H)OTf, 2477537 for (L2+H)BF_4, 2477538 for [Co_2Br_4(L6$_{Macro}$)], 2477539 for [$CoBr_2$(L5)], 2477540 for [$CoBr_2$(L4)], 2477541 for *o*-Diguanidinophenazine, 2477542 for [$NiCl_2$(L2)], 2477543 for [$NiCl_2$(L3)], 2477544 for L2, 2477545 for L1, 2477546 for (L1+2H)Cl_2, 2477547 for (L2+2H)Cl_2, 2477548 for (L1+H)PF_6 2477549 for (L3+2H)Cl_2 and 2477550 for [Cu(BF_4)$_2$(L4)$_2$] contain the supplementary crystallographic data for this paper. These data are provided free of charge by the joint Cambridge Crystallographic Data Centre and Fachinformationszentrum Karlsruhe Access Structure service.

2. Synthesis protocols and analytical data of the activated urea species

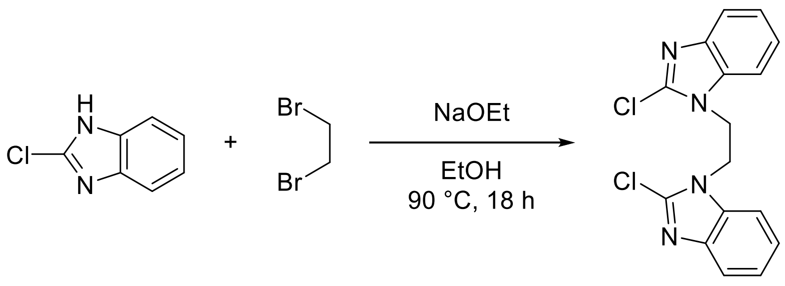

1

NaOEt (914 mg, 13.4 mmol, 2.05 eq.) was suspended in EtOH (40.0 mL). 2-Chlor-1*H*-benzimidazole (2.00 g, 13.1 mmol, 2.00 eq.) and 1,2-Dibromoethane (0.60 mL, 1.30 g, 6.88 mmol, 1.05 eq.) was added to the suspension. The reaction mixture was refluxed for 16 h at 90 °C. After cooling to room temperature water was added (200 mL). The resulting precipitate was filtered, washed with water and dried *in vacuo*. Product **1** could be obtained as a colorless solid (1.23 g, 3.71 mmol, 57%).[10]

^{1}H NMR (400 MHz, CD_2Cl_2, 295 K): δ = 7.60 (dt, *J* = 8.2, 0.9 Hz, 2 H, CH), 7.21 (dtd, *J* = 21.4, 7.9, 1.2 Hz, 4 H, CH), 6,95 (dt, *J* = 7.9, 1.0 Hz, 2 H, CH), 4,60 (s, 4 H, CH_2) ppm.

^{13}C{^{1}H} NMR (150.90 MHz, DMSO-d6, 295 K): δ = 140.98 (Cq.), 139.61 (Cq), 134.87 (Cq.), 123.10 (CH), 122.66 (CH), 118.71 (CH), 109.73 (Cq), 43.04 (CH_2-CH_2) ppm.

MS (EI^+): *m/z* = 330.0429 $[M]^+$, (calc. 330.0439).

EA (C, H, N in %) (x1 H_2O) $C_{16}H_{12}Cl_2N_4$: calc.: C 55.03, H 4.04, N 16.04
found: C 54.76, H 3.66, N 16.67.

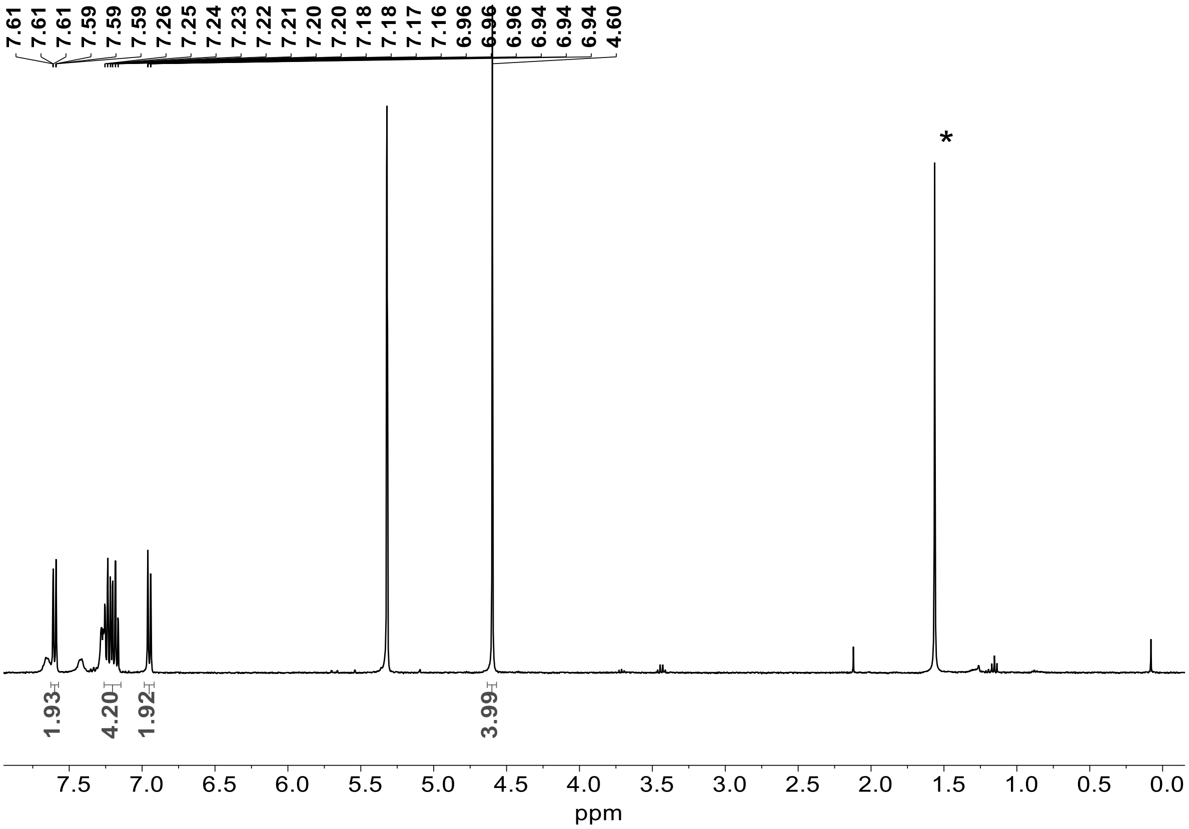

Figure 1: ^{1}H NMR spectrum of **1**, measured at 400 MHz, 295 K in CD_2Cl_2, *H_2O.

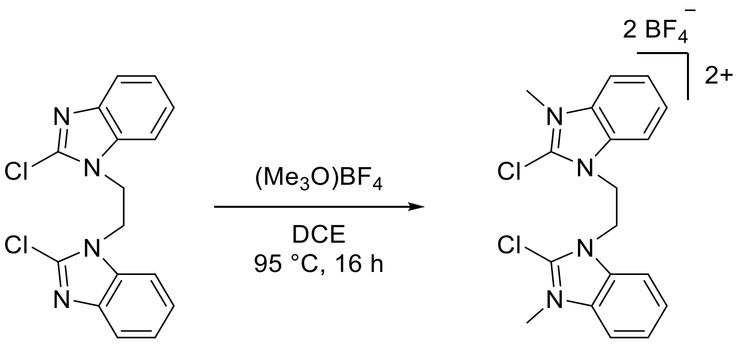

1 **1(BF$_4$)$_2$**

Trimethyloxonium tetrafluoroborate (2.08 g, 14.0 mmol, 3.00 eq.) was suspended in DCE (40 mL). **1** (1.55 g, 4.68 mmol, 1.00 eq.) was added and the reaction mixture was stirred at 95 °C für 19 h. After cooling first to r.t. and then to -15 °C for 4 h, the resulting precipitate was isolated and washed with EtOH (40 mL). After drying *in vacuo*, the product **1(BF$_4$)$_2$** was obtained as a colorless solid (1.69 g, 3.16 mmol, 68%).

^{1}H NMR (400 MHz, CD_3CN, 295 K): δ =7.92–7.87 (m, 4 H, CH_{Ar}), 7.81–7.74 (m, 4 H, CH_{Ar}), 5.04 (s, 4 H, CH_2), 4.00 (s, 6 H, CH_3) ppm.

^{13}C{^{1}H} NMR (101 MHz, CD_3CN, 295 K): δ = 142.3 (2 C, C_q), 132.6 (2 C, C_q), 131.4 (2 C, C_q), 129.1 (2 C, CH), 128.9 (2 C, CH), 114.5 (2 C, CH), 113.6 (2 C, CH), 45.0 (2 C, CH_2), 34.2 (2 C, CH_3) ppm.

MS (ESI$^+$) *m/z* = 345.0669 [M-Me]$^+$,(calc. 345.0668).

EA (C, H, N in %) (x0,5 DCE) $C_{18}H_{18}N_4B_2Cl_2F_8$: calc.: C 39.05, H 3.45, N 9.59
found: C 38.71, H 3.46, N 10.54.

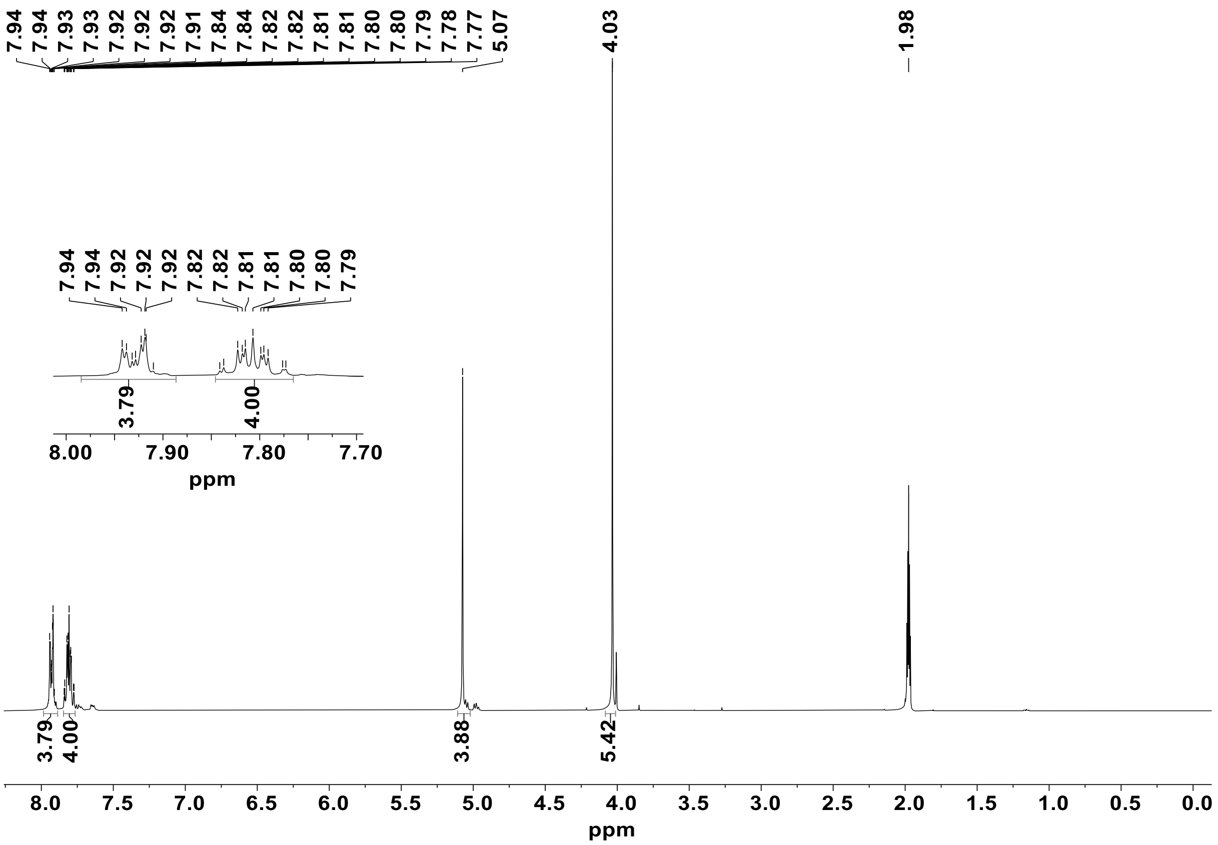

Figure 2: ^{1}H NMR spectrum of **1(BF$_4$)$_2$**, measured at 400 MHz, 295 K in CD_3CN.

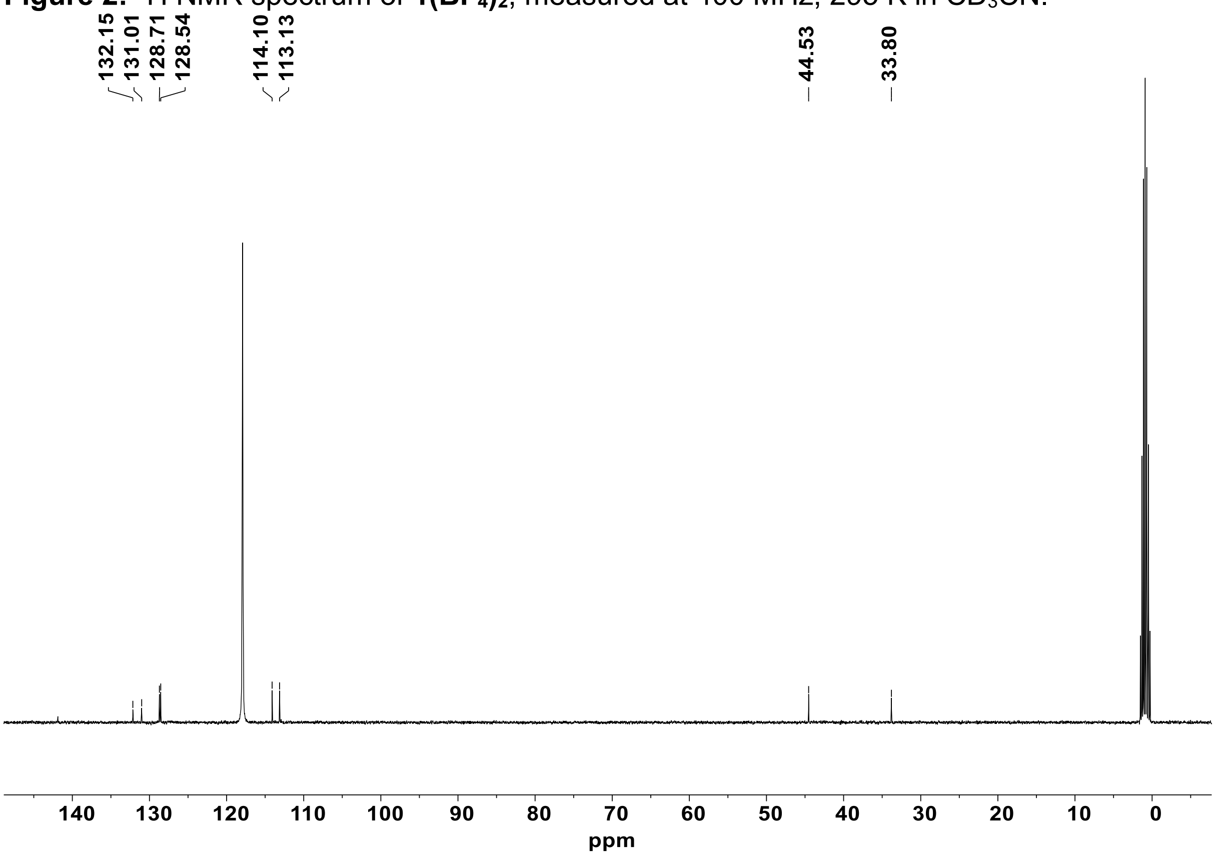

Figure 3: ^{13}C NMR spectrum of 1**(BF$_4$)$_2$**, measured at 100.55 MHz in CD_2Cl_2, 295 K.

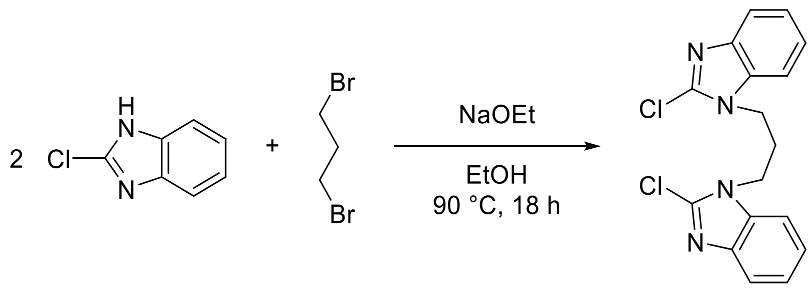

2

NaOEt (914 mg, 13.4 mmol, 2.05 eq.) was suspended in EtOH (40.0 mL). 2-Chlor-1*H*-benzimidazole (2.00 g, 13.1 mmol, 2.00 eq.) and 1,3-Dibromopropane (0.72 mL, 1.39 g, 6.88 mmol, 1.05 eq.) were added. The reaction mixture was stirred for 16 h at 90 °C. After cooling to r.t. water (200 mL) was added. The resulting precipitate was filtered, washed with water and dried *in vacuo*. Product **2** could be obtained as a colorless solid (1.81 g, 5.25 mmol, 80%).

^{1}H NMR (400 MHz, CD_2Cl_2, 295 K): δ = 7.70–7.62 (m, 2 H, CH_{Ar}), 7.33 – 7.24 (m, 4 H, CH_{Ar}), 7.24–7.16 (m, 2 H, CH_{Ar}), 4.28 (d, *J* = 7.4 Hz, 4 H, CH_2), 2.38 (q, *J* = 7.6 Hz, 2 H, CH_2) ppm.

^{13}C{^{1}H} NMR (101 MHz, CD_2Cl_2, 295 K): δ = 142.3 (2 C, C_q), 140.6 (2 C, C_q), 135.2 (2 C, C_q), 123.7 (2 C, CH_{Ar}), 123.2 (2 C, CH_{Ar}), 119.9 (2 C, CH_{Ar}), 109.5 (2 C, CH_{Ar}), 42.0 (2 C, CH_2), 29.2 (1 C, CH_2) ppm.

MS (EI^+) *m/z* = 344.0605 $[M]^+$, (calc. 344.0596).

EA (C, H, N in %) (x0,2 H_2O) $C_{17}H_{14}Cl_2N_4$: calc: C 59.42, H 4.82, N 15.40
found: C 58.47, H 3.81, N 15.96.

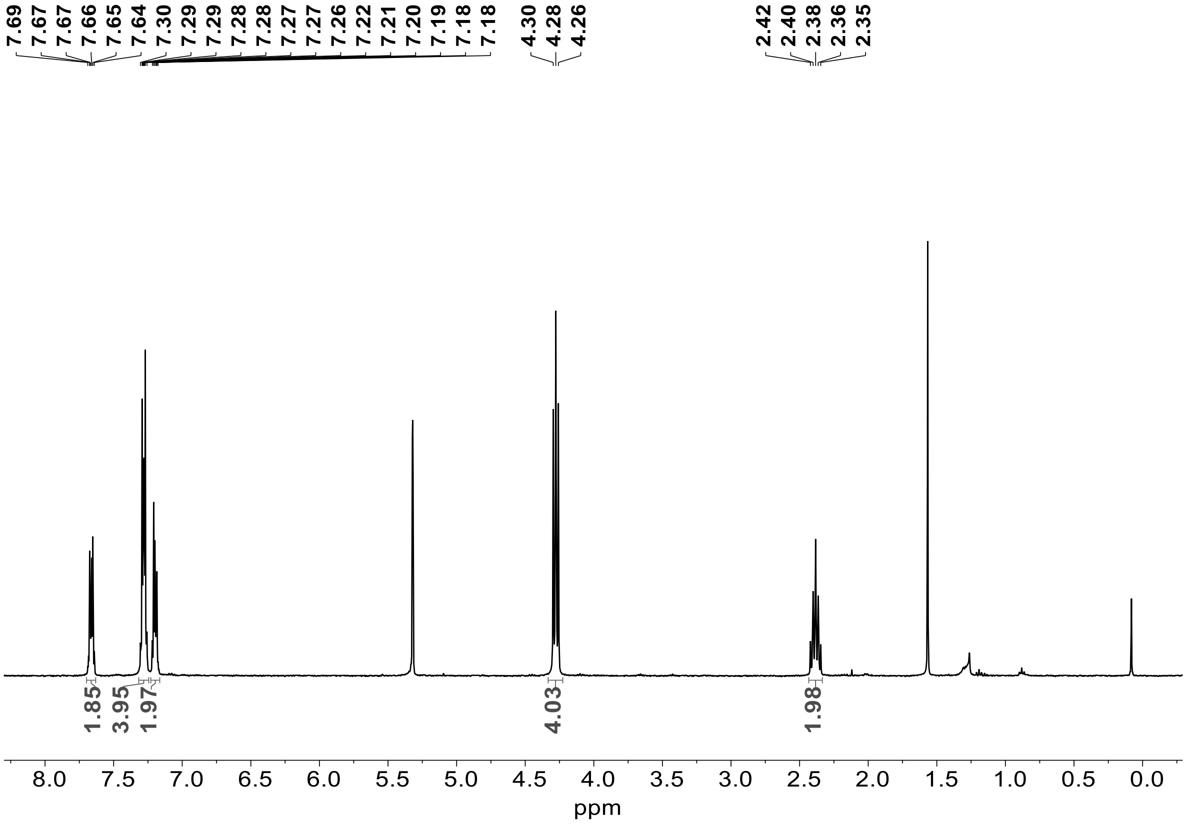

Figure 4: ^{1}H NMR spectrum of **2**, measured at 400 MHz, 295 K in CD_2Cl_2.

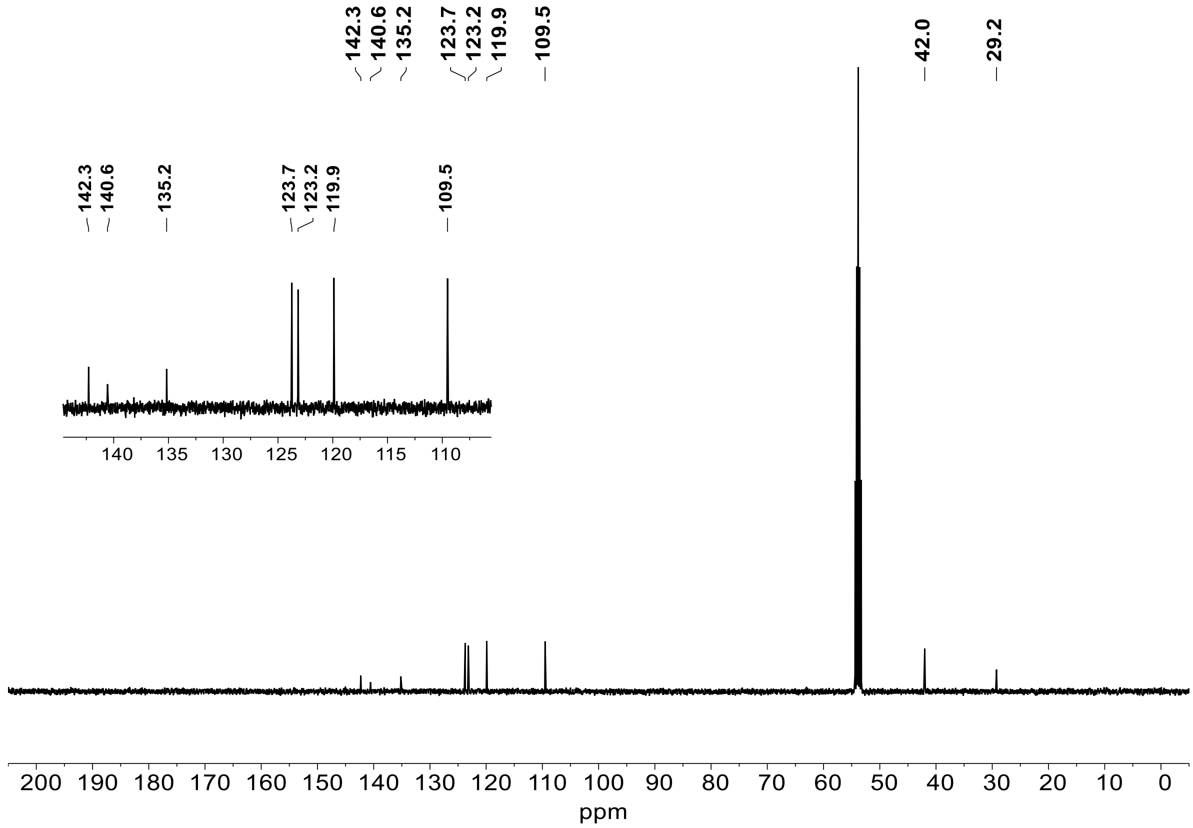

Figure 5: ^{13}C NMR spectrum of **2**, measured at 101 MHz in CD_2Cl_2, 295 K.

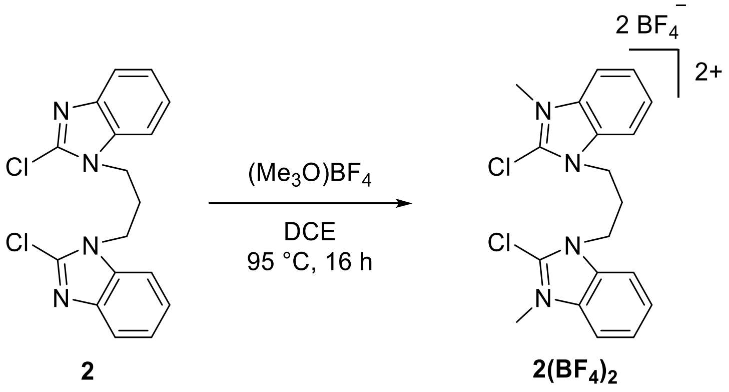

Trimethyloxonium tetrafluoroborate (2.02 g, 13.6 mmol, 2.6 eq.) was suspended in DCE (35 mL). **2** (1.81 g, 5.24 mmol, 1.00 eq.) was added and the reaction mixture was stirred at 95 °C für 16 h. After cooling first to r.t. and then to -15 °C for 4 h, the resulting precipitate was isolated and washed with EtOH (50 mL). After drying *in vacuo* product **2(BF$_4$)$_2$** was obtained as a colorless solid (2.55 g, 4.65 mmol, 89%).

^{1}H NMR (600 MHz, CD_3CN, 295 K): δ = 7.94–7.92 (m, 2 H, CH_{Ar}), 7.88–7.86 (m, 2 H, CH_{Ar}), 7.77–7.72 (m, 4 H, CH_{Ar}), 4.73–4.64 (m, 4 H, CH_2), 4.02 (s, 6 H, CH_3), 2.56–2.43 (m, 2 H, CH_2) ppm.

^{13}C{^{1}H} NMR (100.55 MHz, CD_3CN, 295 K): δ = 132.3 (2 C, C_q), 131.21 (2 C, C_q), 128.3 (2 C, CH), 127.8 (2 C, CH), 114.9 (2 C, CH), 113.3 (2 C, CH), 44.3 (2C, CH_2), 33.4 (2 C, CH_3), 27.46 (1 C, CH_2) ppm.

^{19}F NMR (376.27 MHz, CD_3CN, 295 K): δ = -151.58 ppm.

MS (ESI$^+$ in DCM): *m/z* = 461.1089 ([L+BF_4^-]$^+$), calc.: 461.1089).

EA (C, H, N in %) $C_{19}H_{20}B_2Cl_2F_8N_4$: calc.: C 41.58, H 3.67, N 10.21.
found: C 41.41, H 3.97, N 10.36.

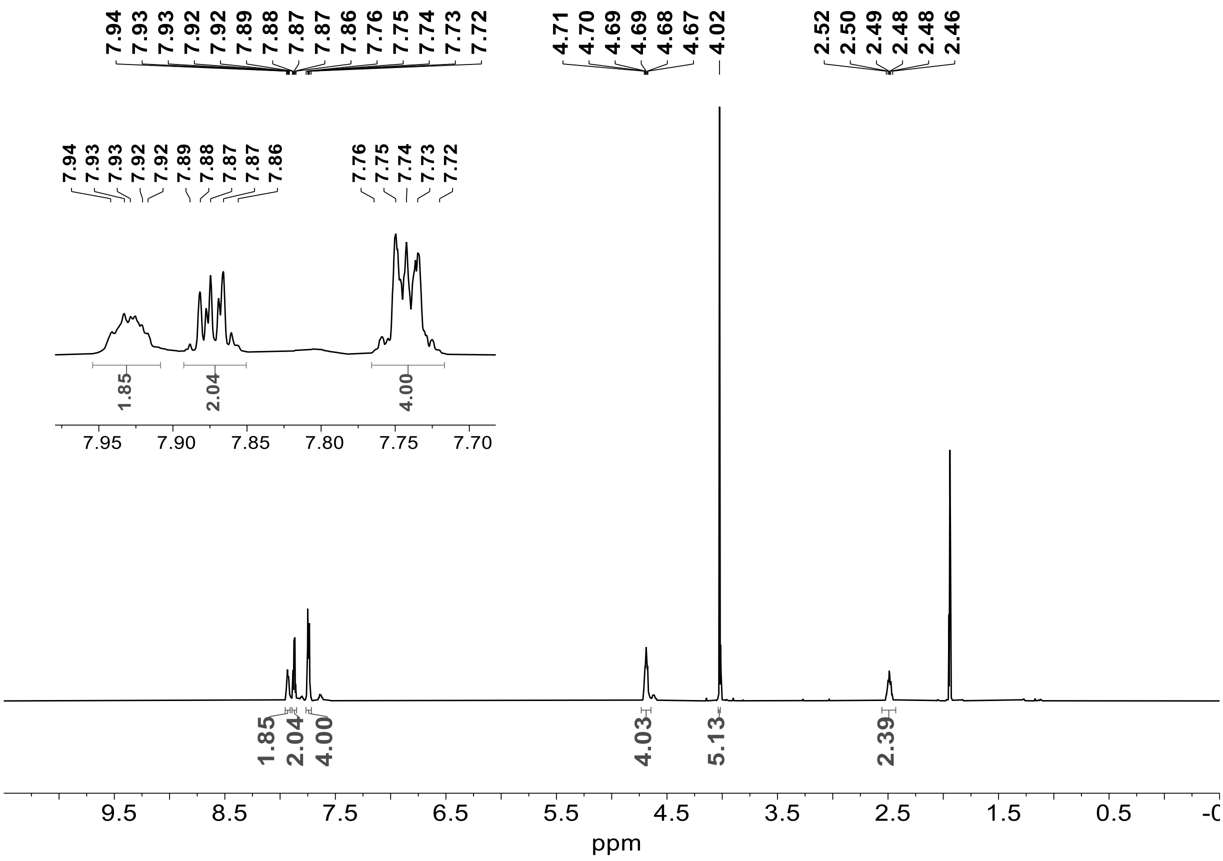

Figure 6: ^{1}H NMR spectrum of **2(BF$_4$)$_2$**, measured at 600 MHz, 295 K in CD_3CN.

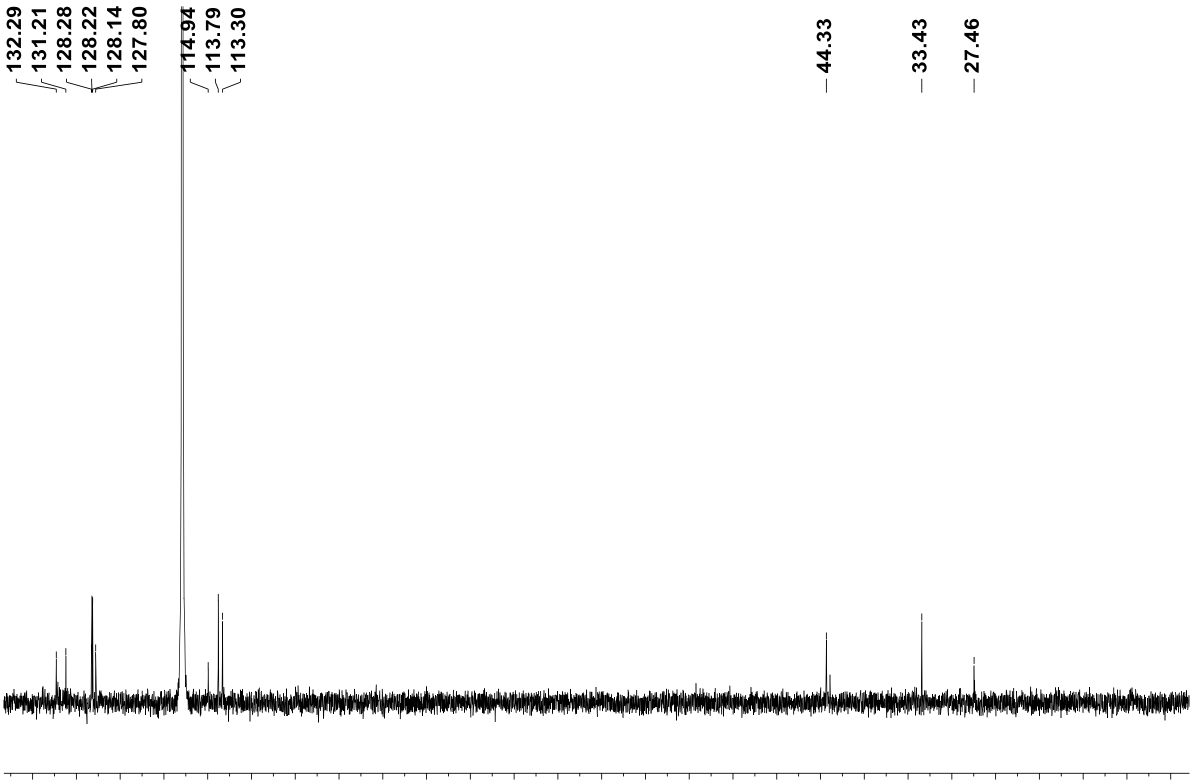

Figure 7: ^{13}C NMR spectrum of **2(BF$_4$)$_2$**, measured at 100.55 MHz, 295 K in CD_3CN.

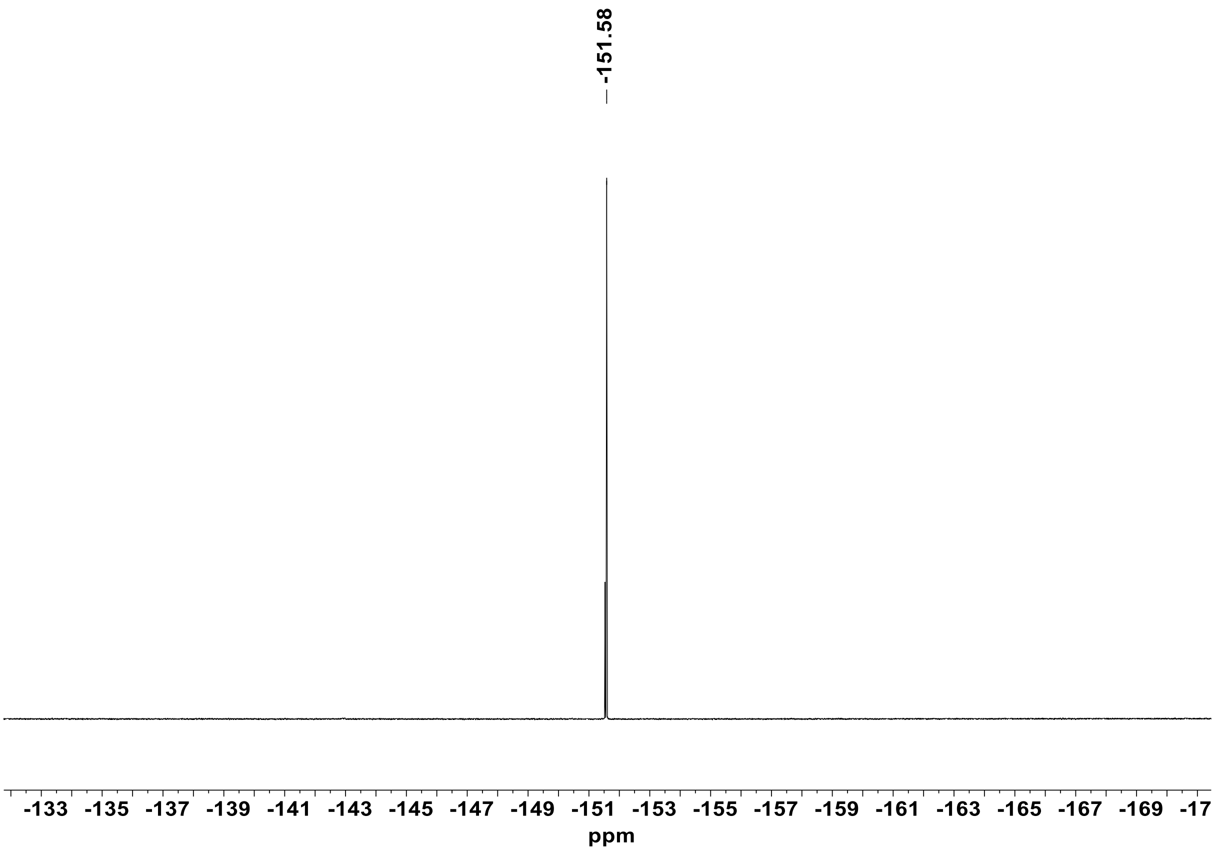

Figure 8: ^{19}F NMR spectrum of **2(BF$_4$)$_2$**, measured at 376.27 MHz, 295 K in CD_3CN.

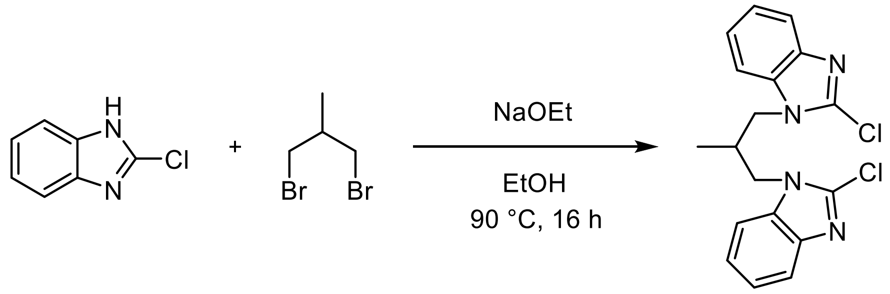

3

NaOEt (914 mg, 13.4 mmol, 2.05 eq.) was suspended in EtOH (40.0 mL). 2-Chlor-1*H*-benzimidazole (2.00 g, 13.1 mmol, 2.00 eq.) and 1,3-Dibromo-2-methylpropane (1.49 g, 6.88 mmol, 1.05 äq.) were added. The reaction mixture was stirred for 16 h at 90 °C. After cooling to r.t. water (200 mL) was added. The resulting precipitate was filtered, washed with water and dried *in vacuo*. Product **3** could be obtained as a colorless solid (1.11 g, 3.2 mmol, 47%).

^{1}H NMR (600 MHz, CD_2Cl_2): δ = 7.68–7.64 (m, 2 H), 7.30–7.26 (m, 4 H), 7.20–7.17 (m, 2 H), 4.19–4.11 (m, 4 H), 2.89–2.81 (m, 1 H), 1.01 (d, *J*= 6.7 Hz, 3 H) ppm.

^{13}C NMR (151 MHz, $CDCl_3$): δ = 141.75 (2 C, Cq), 140.51 (2 C, Cq), 134.98 (2 C, Cq), 123.62 (2 C, C_{Ar}), 123.11 (2 C, C_{Ar}), 119.89 (2 C, C_{Ar}), 109.20(2 C, C_{Ar}), 47.83 (1 C, CH_2), 34.70 (1 C, CH_2), 16.11 (1 C, CH_3) ppm.

MS (EI in DCM): *m/z* = 358.0736 ($[M^-]^+$, calc.: 358.0752).

EA (C, H, N in %) $C_{18}H_{16}Cl_2N_4$: calc.: C 60.18, H 4.49, N 15.60

found: C 59.83, H 4.55, N 15.57.

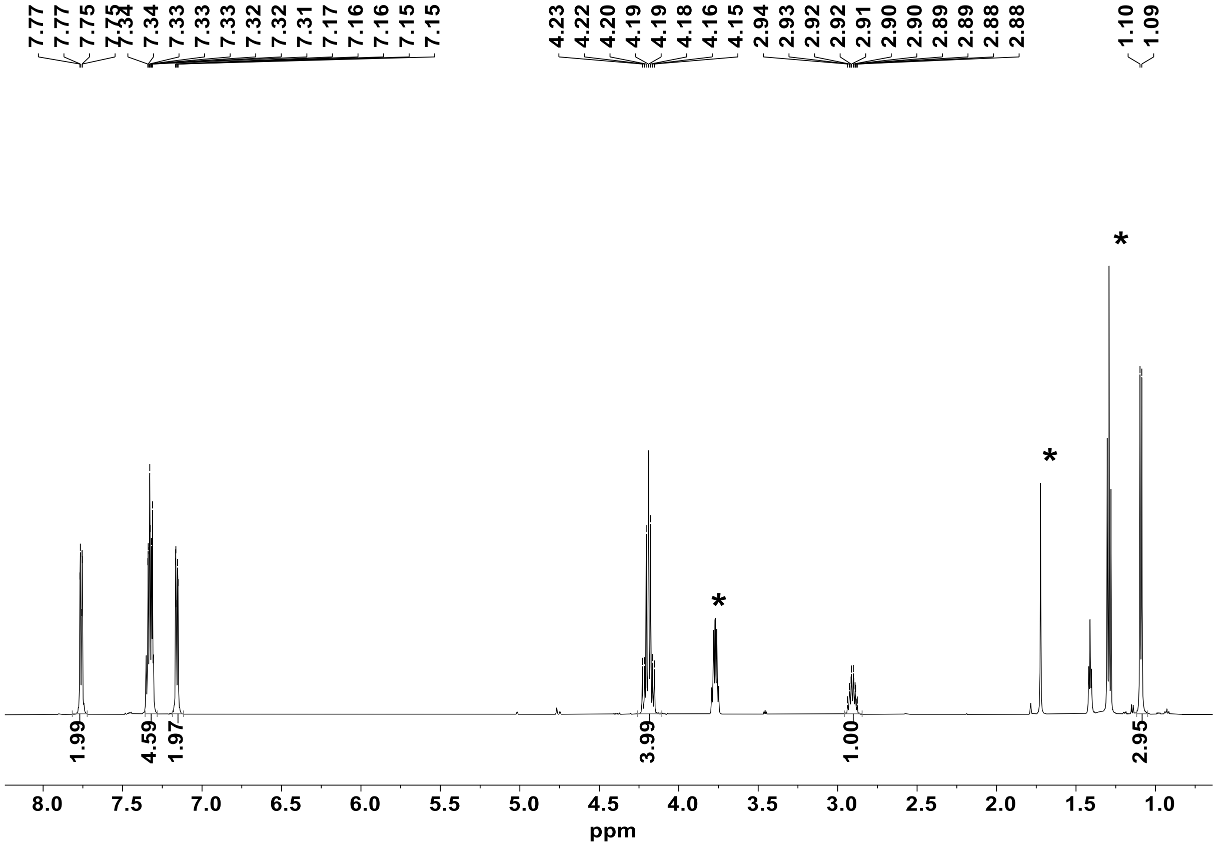

Figure 9: ^{1}H NMR spectrum of **3**, measured at 400 MHz, 295 K in $CDCl_3$, * H_2O, Ethanol.

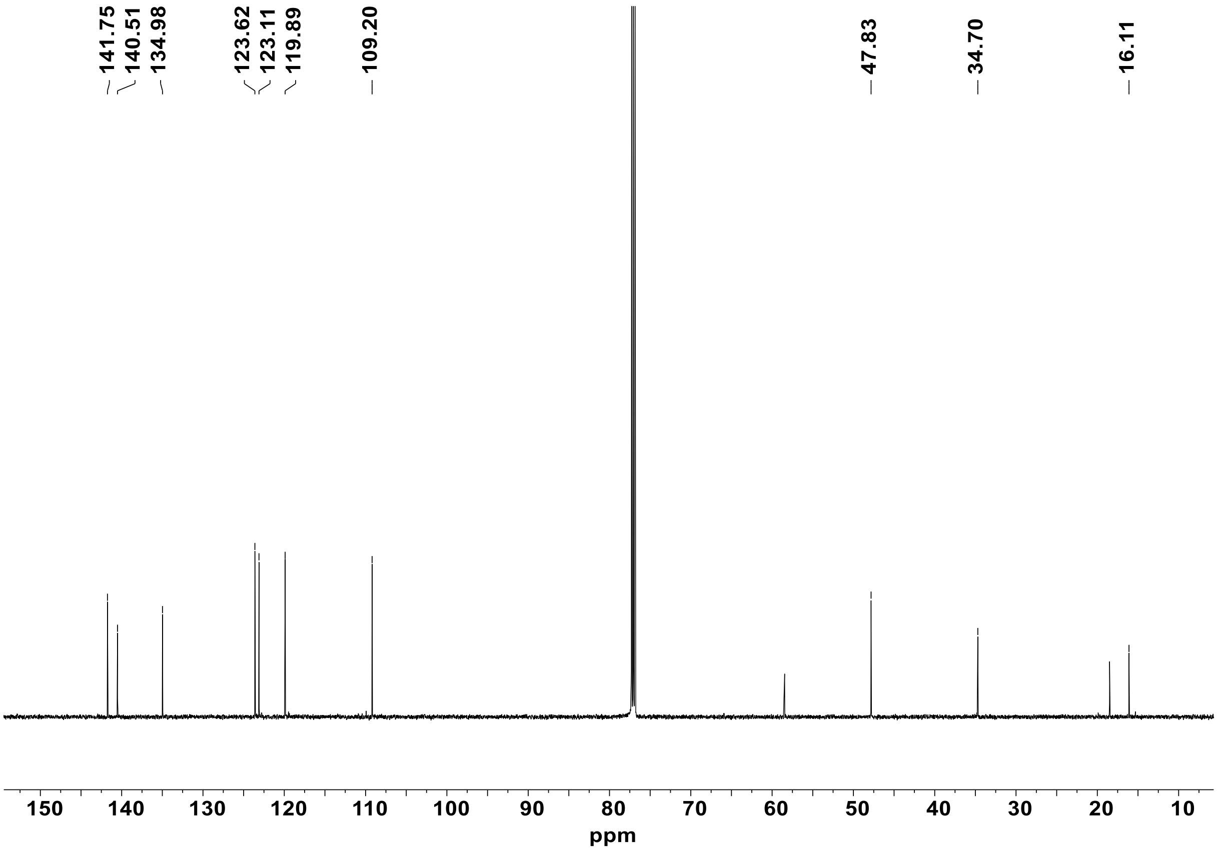

Figure 10: ^{13}C NMR spectrum of **3**, measured at 151 MHz, 295 K in CD_3CN.

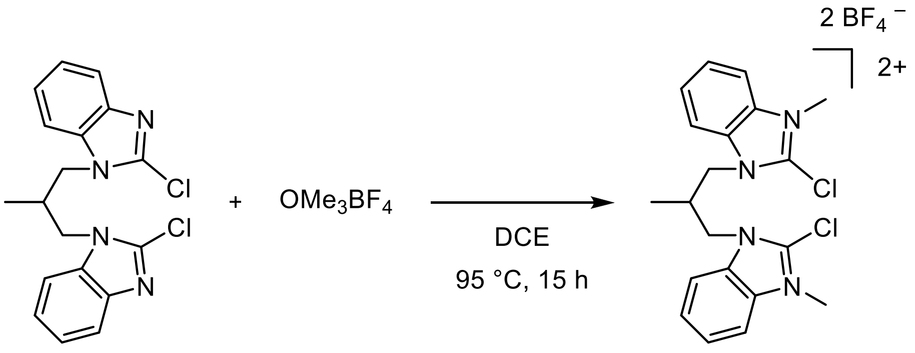

3 **3(BF₄)₂**

Trimethyloxonium tetrafluoroborate (944 mg, 6.38 mmol, 3.20 eq) was suspended in DCE (35 mL). **3** (716 mg, 1.99 mmol, 1.00 äq.) was added and the reaction mixture was stirred at 95 °C für 16 h. After cooling first to r.t. and then to -15 °C for 4 h, the resulting precipitate was isolated and washed with EtOH (50 mL). After drying *in vacuo,* the product **3(BF$_4$)$_2$** was obtained as a colorless solid (788 mg, 1.37 mmol, 68%)

^{1}H NMR (600 MHz, CD_3CN): δ = 7.90–7.87 (m, 4 H), 7.77–7.75 (m, 4 H), 4.65–4.54 (m, 4 H), 4.04 (s, 6 H), 2.93 (m, 1 H), 1.00 (d, J = 6.6 Hz, 3 H) ppm.

^{13}C NMR (101 MHz, CD_3CN): δ = 141.60, 132.26, 131.78, 128.30, 128.16, 113.83, 113.50, 49.81, 34.00, 33.56, 14.65 ppm.

^{19}F NMR (376.27 MHz, CD_3CN, 295 K): δ = -151.61 ppm.

MS (ESI$^+$ in DCM): m/z = 475.1248 ([L+BF_4^-]$^+$, calc.: 475.1245).

EA (C, H, N in %) $C_{20}H_{22}B_2Cl_2F_8N_4$: calc.: C 42.67, H 3.94, N 9.95
found: C 42.27, H 4.17, N 9.69

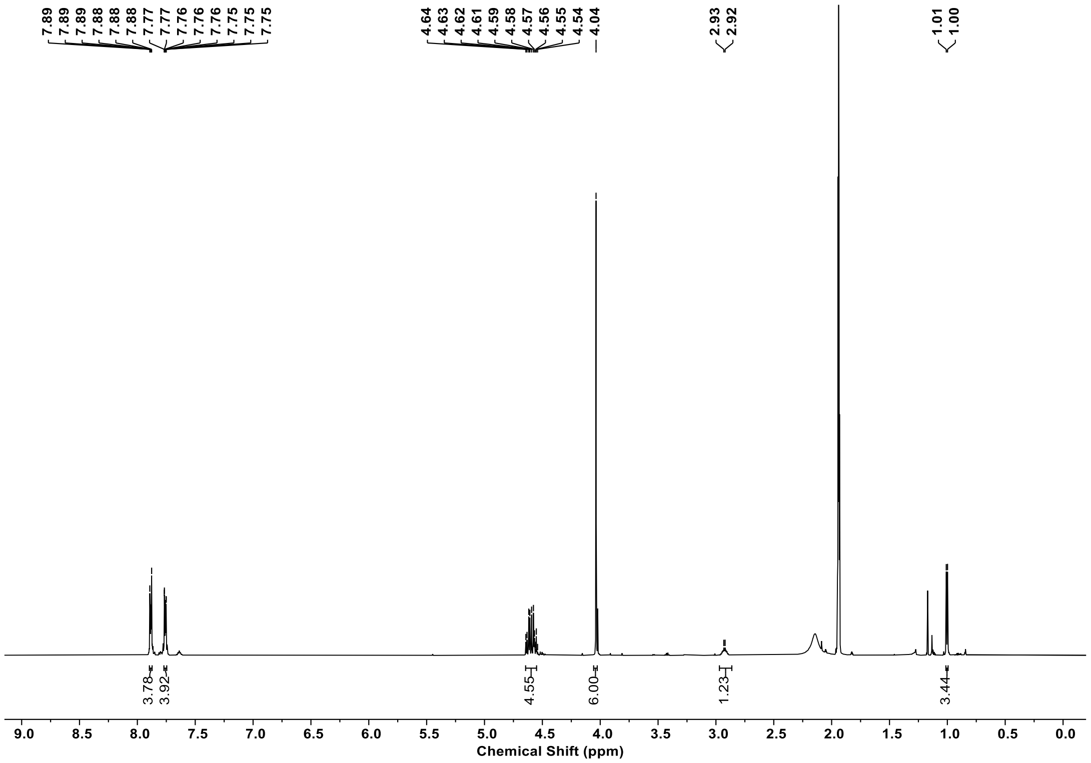

Figure 11: ^{1}H NMR spectrum of **3(BF$_4$)$_2$**, measured at 600 MHz, 295 K in CD_3CN.

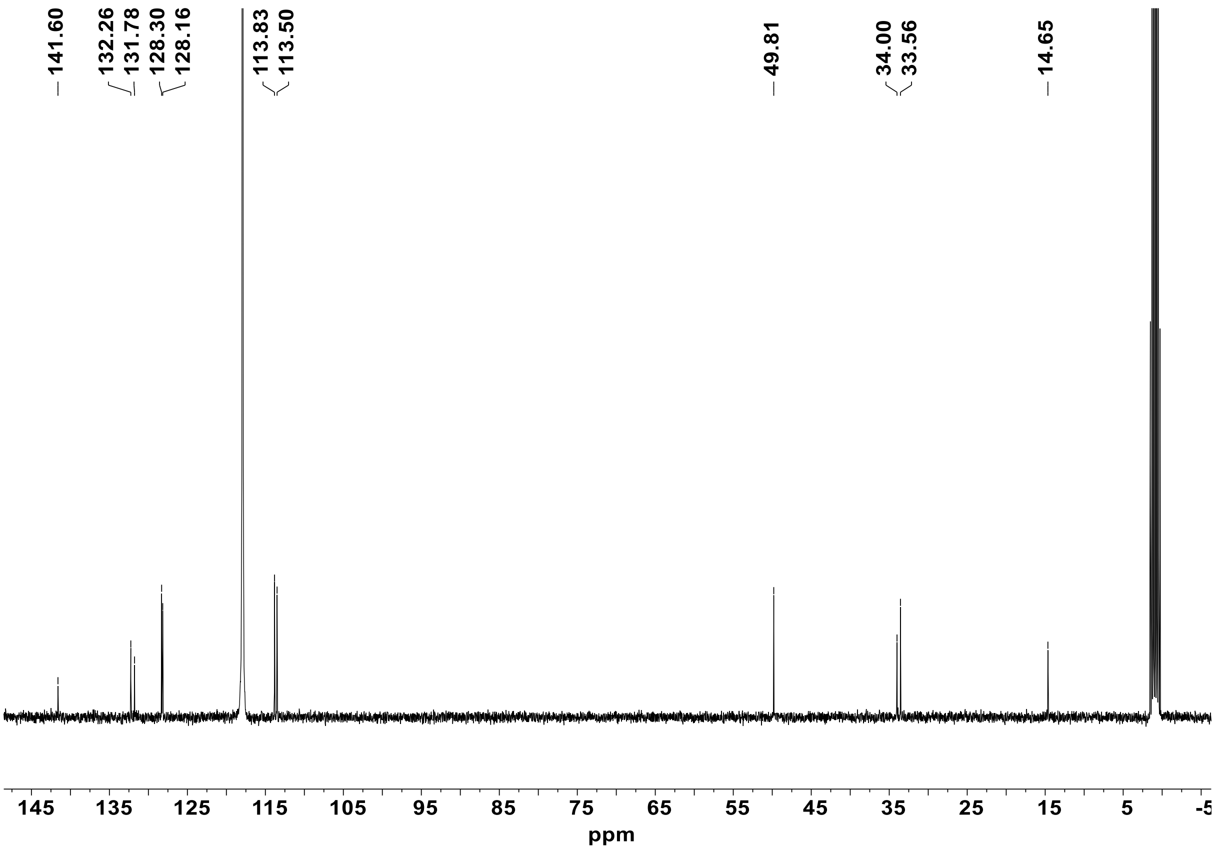

Figure 12: ^{13}C NMR spectrum of **3(BF$_4$)$_2$**, measured at 100.55 MHz, 295 K in CD_3CN.

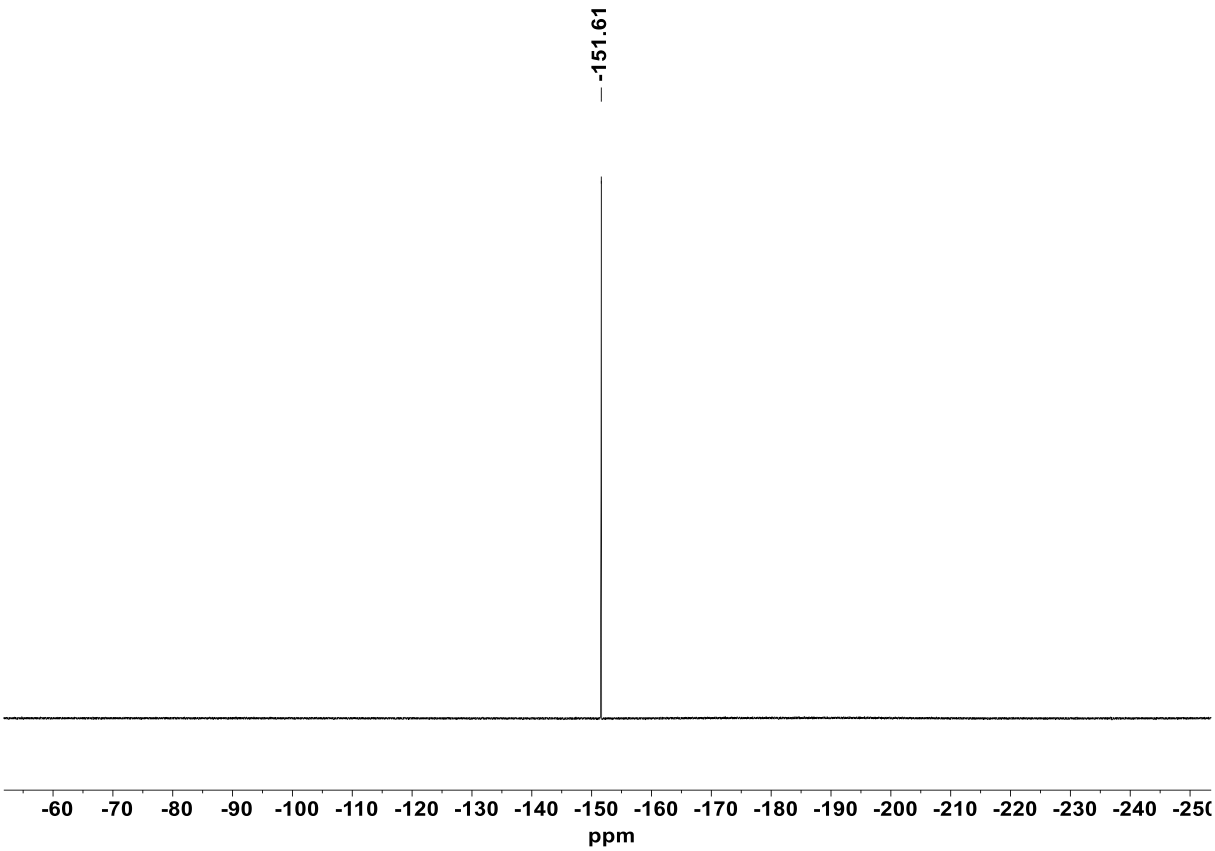

Figure 13: ^{19}F NMR spectrum of **$3(BF_4)_2$**, measured at 376.27 MHz, 295 K in CD_3CN.

3. Synthesis protocols, analytical data and quantum chemical calculations of the ligands

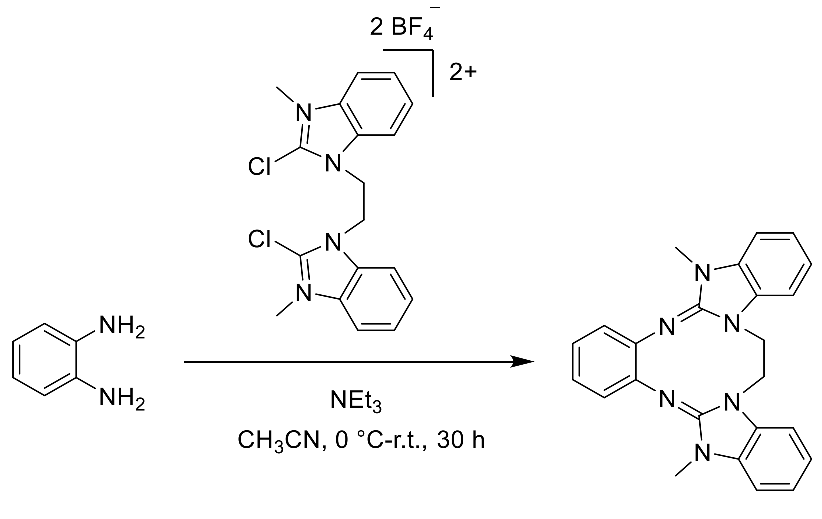

L1

Compound **1(BF$_4$)$_2$** (1.20 g, 2.24 mmol, 1.50 eq.) was suspended at 0 °C in MeCN (30.0 mL). *o*-Phenylendiamine (161 mg, 1.50 mmol, 1.00 eq.) and NEt$_3$ (2.07 mL, 1.51 g, 14.9 mmol, 10.0 eq.) were slowly added to the reaction mixture at 0 °C. While stirring for 30 h the suspension was brought to room temperature. The resulting precipitate was filtered and washed with MeCN (10 mL). **L1** was obtained (136 mg, 176 mmol, 23 %) as a colorless solid. Crystals for structural analysis were obtained out of a saturated DCM-solution.

^{1}H NMR (400 MHz, CD$_2$Cl$_2$, 295 K): δ = 7.01–6,92 (m, 4 H, CH$_{Ar}$), 6,85 (td, *J*=7.8, 1.1 Hz, 2 H, CH$_{Ar,}$), 6,73 (td, *J*=7.7, 1.2 Hz, 2 H, CH$_{Ar}$), 6,65 (d, *J*=7,7 Hz, 2 H, CH$_{Ar,}$), 6,52 (d, *J*=7.7 Hz, 2 H, CH$_{Ar,}$), 3,92–3,80 (m, 4 H, CH$_2$), 3,25 (s, 6 H, CH$_3$) ppm.

^{13}C{^{1}H} NMR (101 MHz, CD$_2$Cl$_2$, 295 K): δ = 147.9 (2 C, C$_q$), 140.2 (2 C, C$_q$), 132.5 (2 C, C$_q$), 132.4 (2 C, C$_q$), 124,5 (2 C, CH), 122,6 (2 C, CH), 121,6 (2C, CH), 120,2 (2 C, CH), 106,6 (2 C, CH), 106,5 (2 C, CH), 46,8 (1 C, CH$_2$), 39,9 (1 C, CH$_2$), 28,4 (1 C, CH$_3$), 9,2 (1 C, CH$_3$) ppm.

MS (ESI$^+$ in DCM): *m/z* = 395.1977 [L+H]$^+$, (calc.: 395.1979).

EA (C, H, N in %) (x0,5 DCM) C$_{24}$H$_{22}$N$_6$: calc.: C 67.35, H 5.31, N 19.23
found: C 66.98, H 5.54, N 18.88.

UV/Vis-spectrum (DCM): λ (ε) = 311 (12800), 262 (shoulder, 6640), 228 (24400) nm (M^{-1} cm^1).

CV (DCM, [*n*-Bu$_4$N][PF$_6$], 100 mV s^{-1}, vs. Fc/Fc$^+$): E$_{Ox}$=178 mV und E$_{Red}$=-140 mV $E_{1/2}{}^1$=366 mV, $E_{1/2}{}^2$=642 mV.

No macrocyclic compound could be isolated out of the remaining reaction mixture after isolation of L1 through filtration. The mixture also showed no sign of complexation through addition of metal salts. Even though the mass of the macrocycle could be observed in the mass spectrum (calc:789.3885, found: 789.3879), this observation is probably due to chain formation and not due to the macrocyclic closed ring structure.

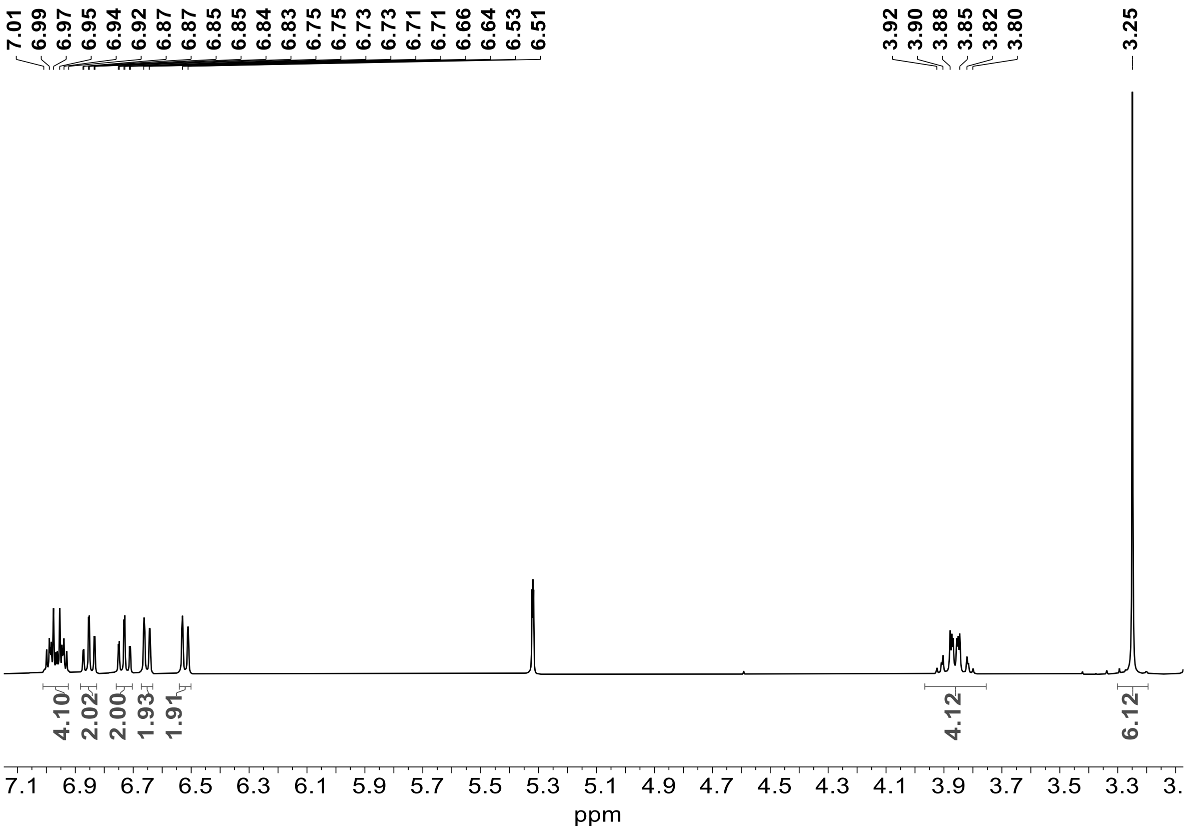

Figure 14: ^{1}H NMR spectrum of **L1**, measured at 400 MHz, 295 K in CD_2Cl_2.

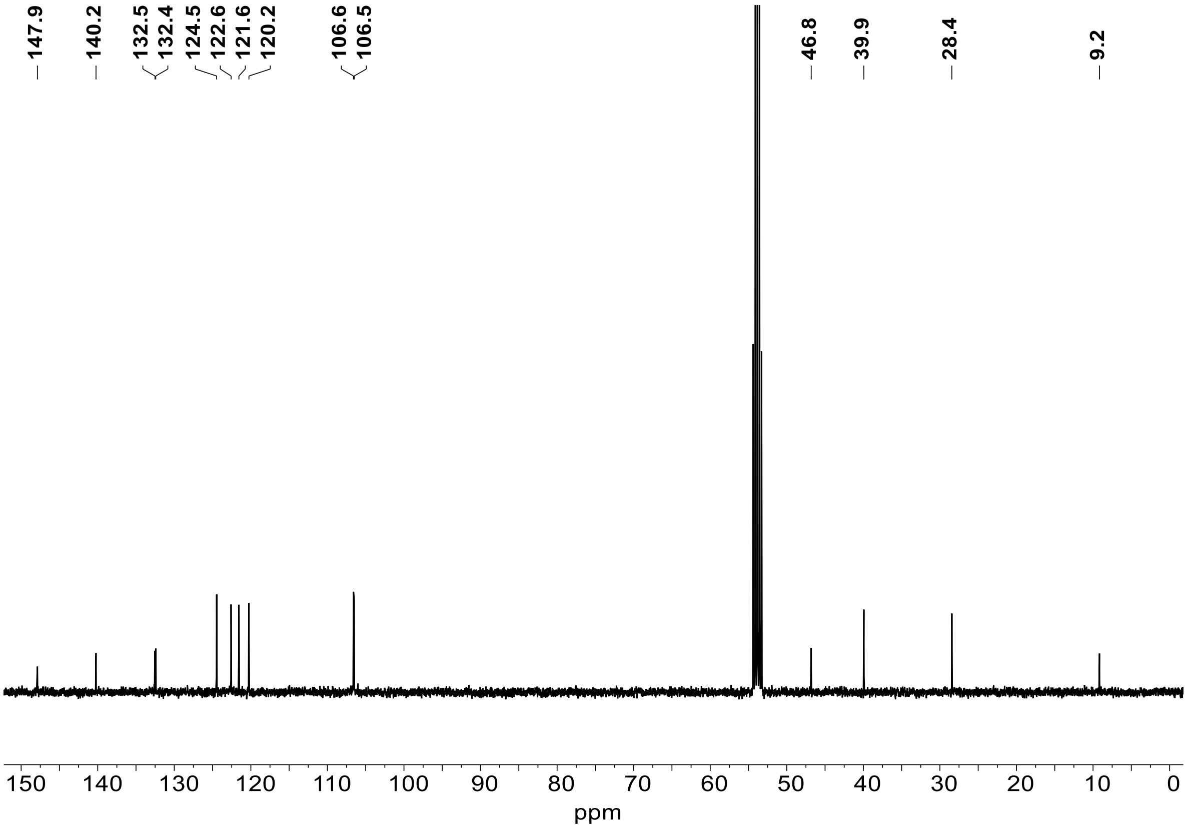

Figure 15: ^{13}C NMR spectrum of **L1**, measured at 101 MHz, 295 K in CD_2Cl_2.

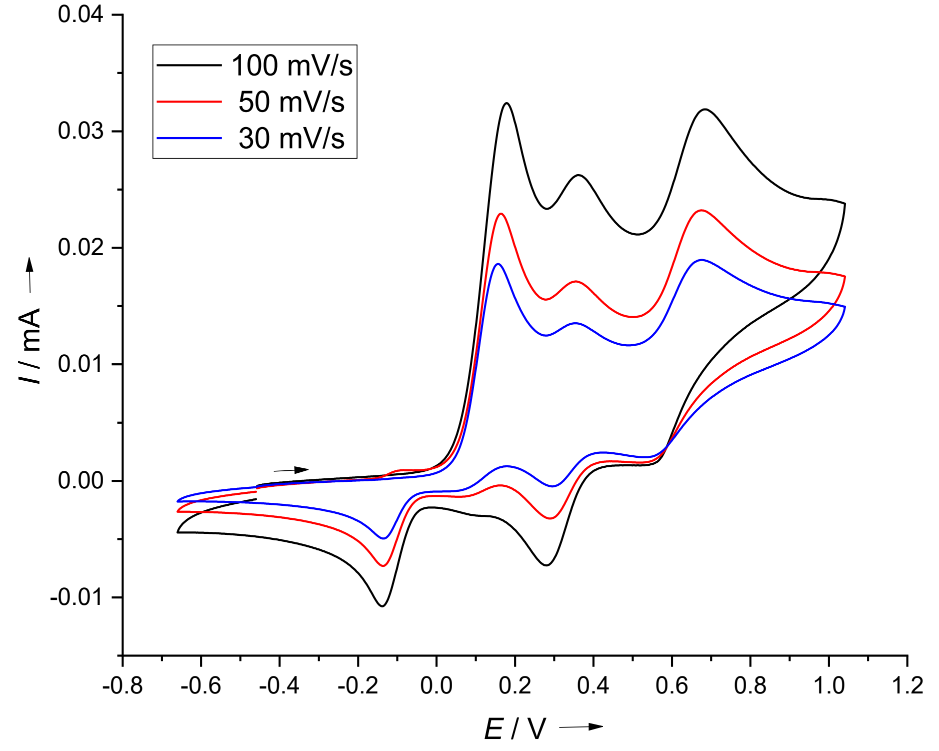

Figure 16: CV curve of **L1** (Fc/Fc$^+$ reference, *n*Bu_4NPF_6, Ag/AgCl electrode, DCM).

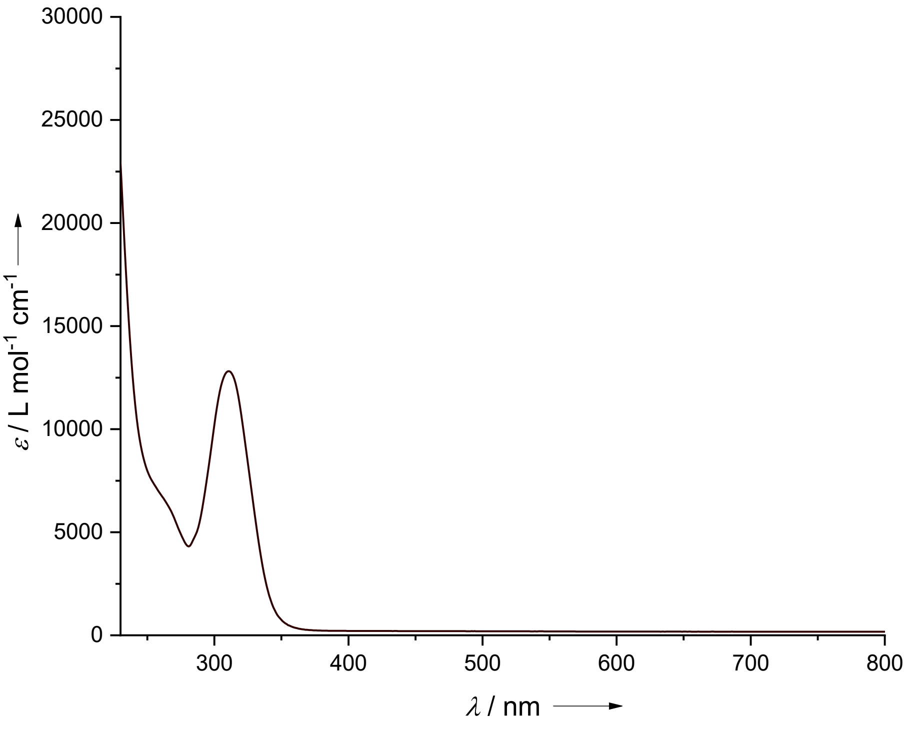

Figure 17: UV/Vis spectrum of **L1** in DCM.

UV/Vis experiment: titration of a solution of L1 in DCM with FcPF6

In a 5 mL volumetric flask, 0.17 mg of **2** were dissolved in CH_2Cl_2, giving a $8.45 \cdot 10^{-5}$ M solution. Then, 3.0 mL of this solution was given into a 1 cm UV-vis cuvette equipped with a septum and titrated with 10 µl of a solution of ferrocenium hexafluorophosphate in CH_2Cl_2 (10 µl corresponds to 0.42 µmol or 0.50 eq. with respect to **L1**).

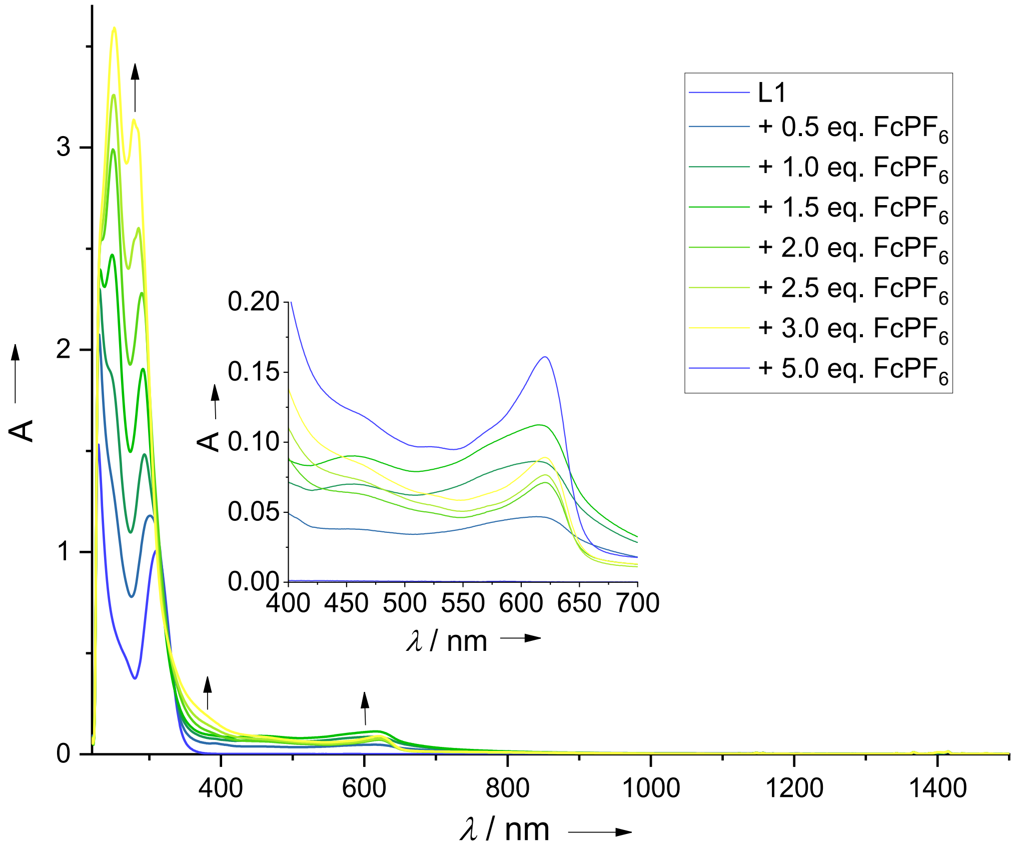

Figure 18: UV/Vis spectra recorded for a solution of **L1** in CH_2Cl_2 without and with addition of various equivalents of $FcPF_6$ to the solution.

(L1+2H)Cl_2

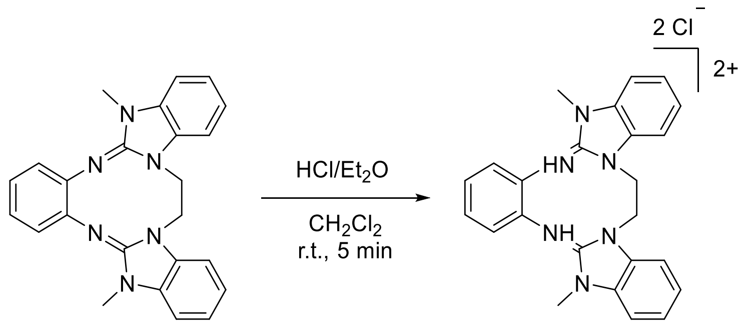

L1 **(L1+2H)Cl_2**

L1 (10.0 mg, 25.4 mmol, 1.00 eq.) was dissolved in DCM (4.00 mL) and HCl in diethylether (0.20 mL, 2.00 M) was added. The resulting precipitate was filtered and dried *in vacuo*. **(L1+2H)Cl_2** could be obtained in quantitative yield as a colorless solid. Crystals for structural analysis could be obtained through diffusion of diethylether in a saturated methanol solution.

^{1}H NMR (400 MHz, D_2O, 295 K): δ = 7.62–7.56 (m, 4 H, CH_{Ar}), 7,34–7,29 (m, 4 H, CH_{Ar}), 7.22–7.17 (m, 4 H, CH_{Ar}), 4.67–4.62 (m, 2 H, CH_2), 4.11–4.06 (m, 2 H, CH_2), 3.62 (s, 6 H, CH_3) ppm.

MS (ESI^+ in DCM): *m/z* = 395.1977 ($[L+H]^+$, calc.: 395.1979).

.

Usage of L1 as replacement of Cs_2CO_3[11]:

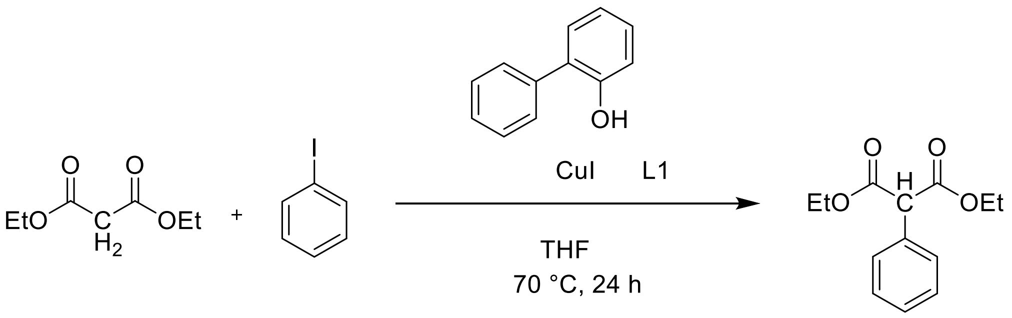

L1 (140 mg, 357 µmol, 1.00 eq.) was mixed with CuI (3.38 mg, 17.7 µmol., 0.05 eq.) and 2-phenylphenol (6.04 mg, 35.49 µmol, 0.10 eq.) and suspended in 1 mL of THF. Iodobenzene (0.03 mL, 355 µmol, 1.00 eq.) and diethyl malonate (0.10 mL, 710 µmol, 2.00 eq.) were added. The mixture was heated to 70°C for 24 h. After cooling to room temperature, the violet suspension was filtered and the solvent removed *in vacuo*. The product was purified via column chromatography (PE:EE 80:20). The solvent was removed from the collected organic phases in vacuo and the product isolated as a yellow oil (9.00 mg, 76.2 µmol, 13%).

^{1}H-NMR (600 MHz, CD_2Cl_2, 295 K): *δ* = 7.52 (m, 4 H, CH_{Ar}), 7.30-7.28 (m, 1 H, CH_{Ar}), 5.26 (s, 1 H, CH), 3.69 (q, J = 7,3 Hz, 4 H, CH_2), 2.06 (t, J = 7.1 Hz, 6 H, CH_3) ppm.

MS (EI in DCM): *m/z* = [M+H$^+$] 237.1127 ([L+H]$^+$, calc.: 237.1121).

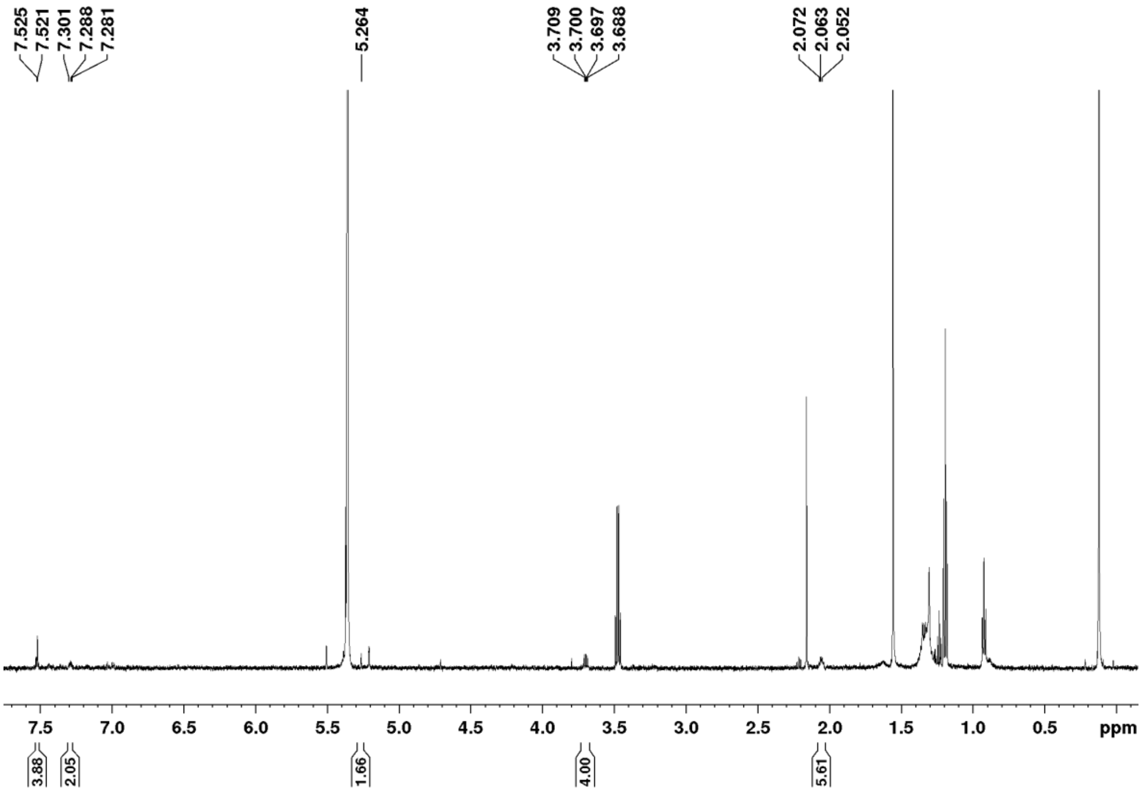

Figure 19: ^{1}H NMR spectrum in CD_2Cl_2 after column chromatography (impurities: ether, acetone, grease from solvent).

NMR Experiment:

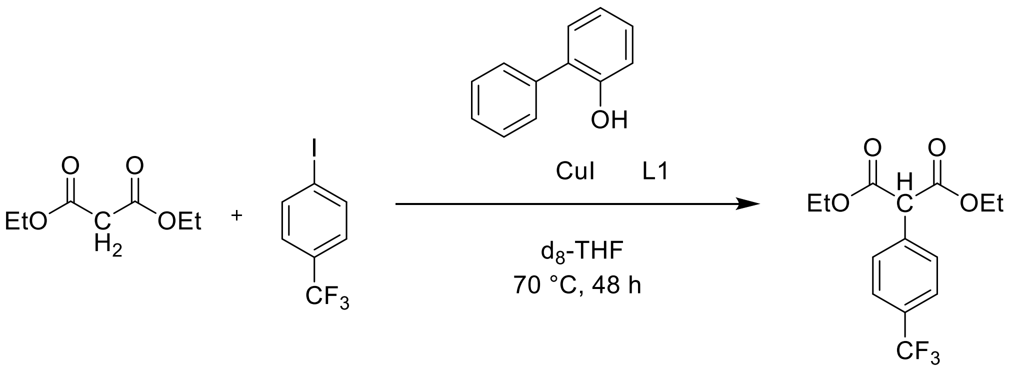

L1 (10 mg, 25.35 µmol, 1.00 eq.) was mixed with CuI (0.24 mg, 1.27 µmol., 0.05 eq.) and 2-phenylphenol (0.43 mg, 2.53 µmol, 0.10 eq.) and suspended in 0.50 mL of d_8-THF. Iodobenzene (3.77 µL, 25.35 µmol, 1.00 eq.) and diethyl malonate (7.33 µL, 50.70 µmol, 2.00 eq.) were added. The mixture was heated to 70 °C for a total of 48 h. ^{19}F-NMR measurements were carried out after 0 h, 24 h, 36 h and 42 h respectively. The maximum yield of 11% was determined through the addition of 1.00 eq. of *o*-DFB after 36 h reaction time.

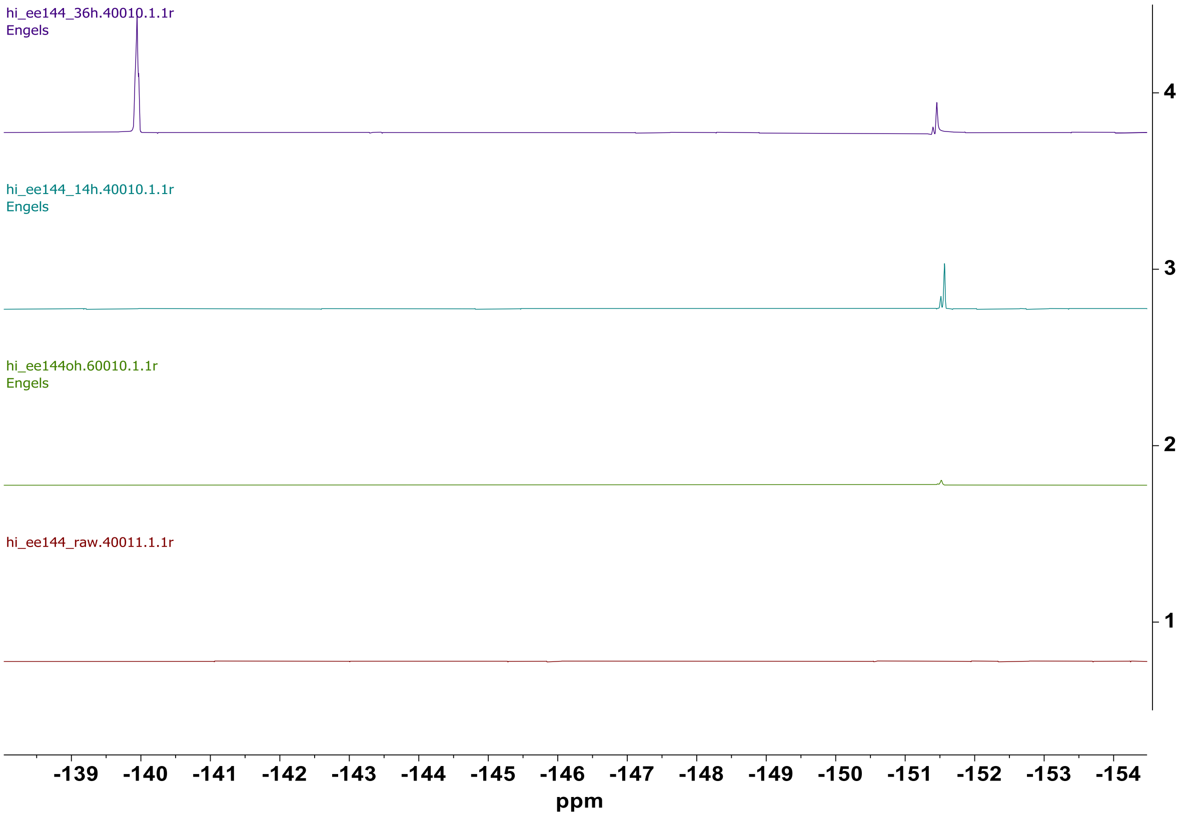

Figure 20: ^{19}F NMR spectra over 48 h in d_8-THF, 1 eq. of *o*-DFB used as an internal marker for the determination of the yield (11% after 36h).

Usage of L1 as Hunigs-Base[12]

NMR experiment

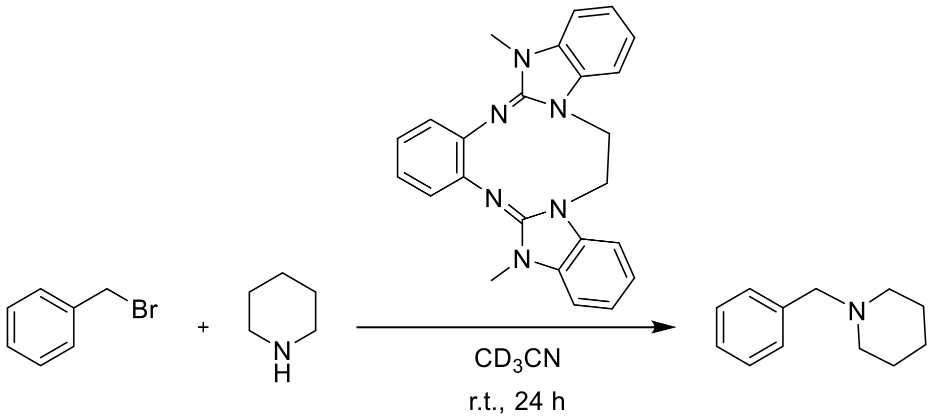

L1 (15 mg, 38 µmol, 1.0 eq.) was dissolved in CD_3CN in a Young NMR Tube. Benzylbromide (4.97 µl, 42 µmol, 1.1 eq.) and piperidine (3.76 µl, 38 µmol,1.0 eq.) were added to the NMR tube and the reaction mixture observed with ^1H NMR spectroscopy over 24h.

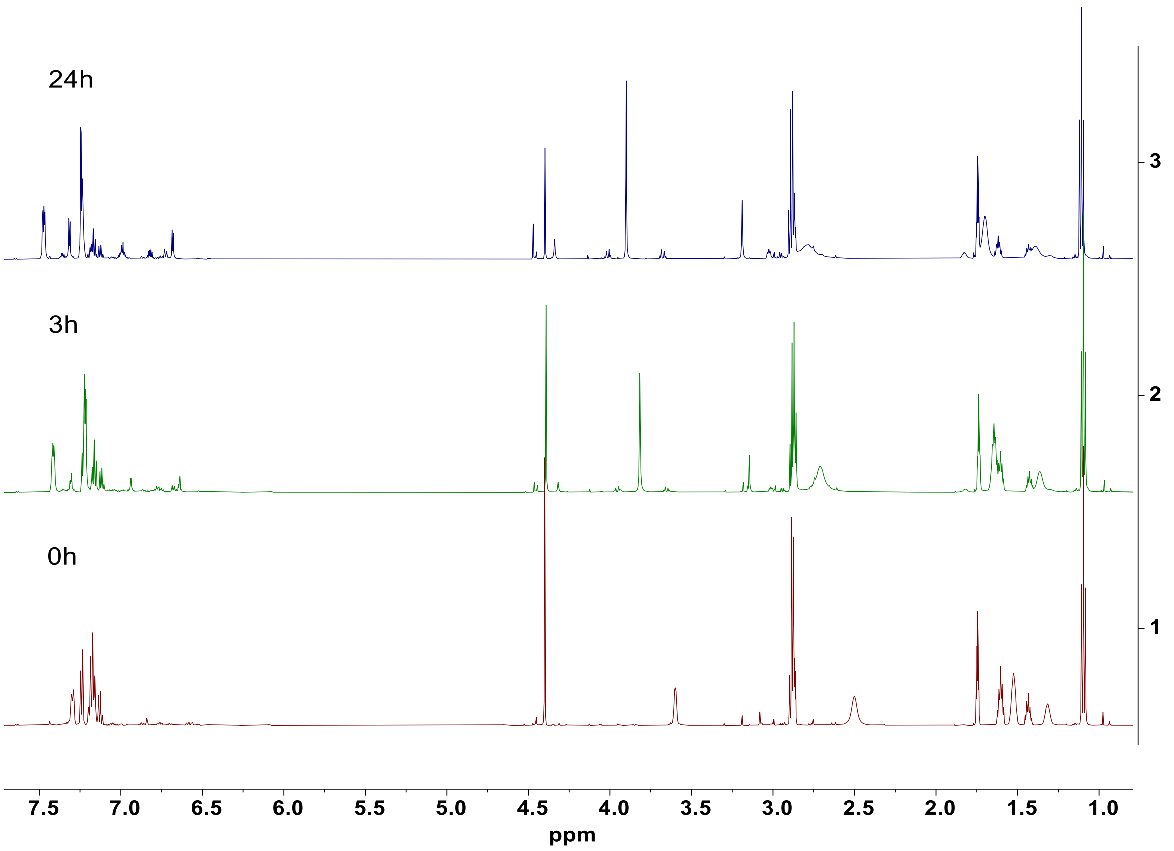

Figure 21: ^1H NMR spectra over 24 h in CD_3CN.

The experiment was repeated on a greater scale, using L1 and L5 for a comparison of the restrained system to an open system.

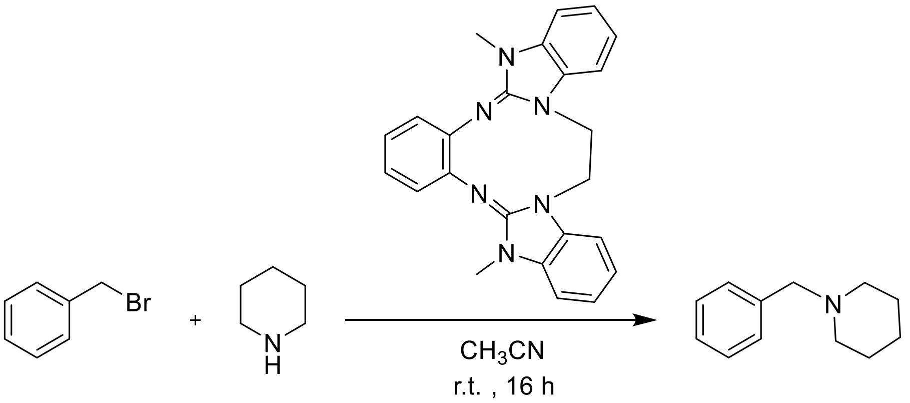

Therefore L1 (45.0 mg, 114 µmol, 0.75 eq.) was suspended in 5.00 mL of MeCN and benzylbromide (28.6 mg, 19.8 µl, 167 µmol, 1.10 eq.) and piperidine (12.9 mg, 15.1 µl, 152 µmol, 1.00 eq.) were added. The reaction mixture was stirred for 16 h at r.t. The reaction was filtered and the solvent removed *in vacuo*. The residue was dissolved in 5 mL of dichloromethane and washed with 5 mL of distilled water. The aqueous layer was washed with 3 x 3 mL fractions of dichloromethane. The collected organic fractions were dried over $MgSO_4$, and the solvent was removed under reduced pressure to yield the crude product as a colorless solid (17.2 mg, 64%). As L1 (17% of the product mass) could be found in the ^{1}H NMR spectrum, this brings the yield of the product to 54%.

^{1}H NMR (600 MHz, $CDCl_3$, 295 K): δ = 7.61-7.50 (d, J=7.3 Hz, 2 H), 7.49-7.45 (m, 3 H), 3.49-3.46 (m, J=7.0 Hz, 3 H), 1.22 (t, 7.0 Hz, 3 H) ppm.

MS (EI, DIP): *m/z* = 174.1296 [M-H] (calc.: 174.1288), 91.0564 [C7H7$^•$] (calc.: 91.0548).

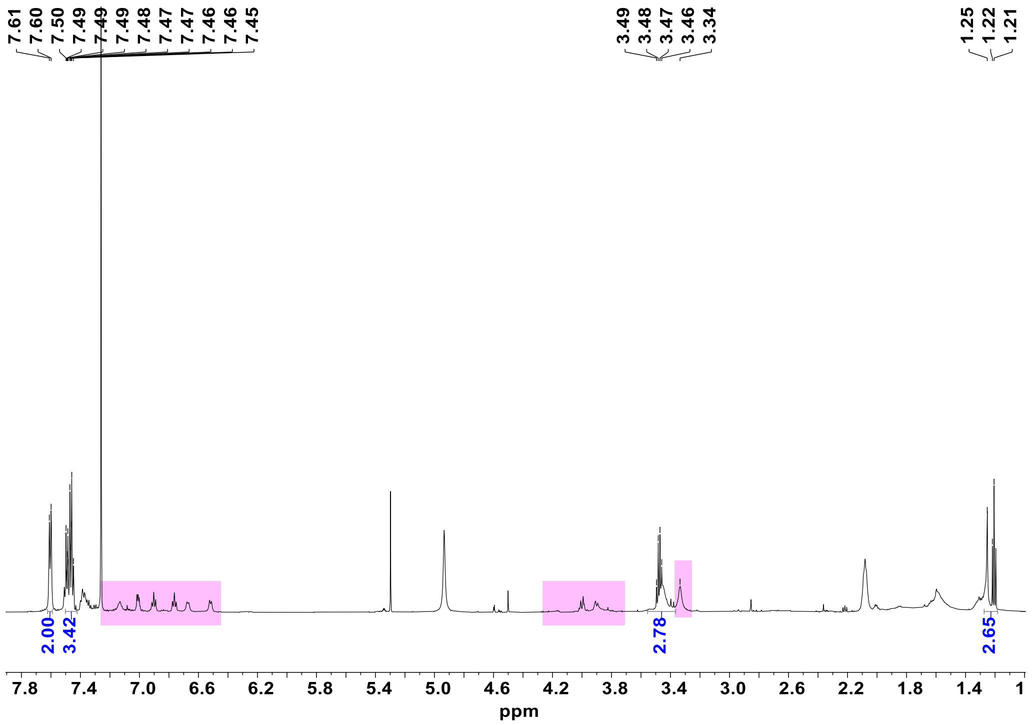

Figure 22: ^{1}H NMR spectrum in $CDCl_3$ after extraction, remaining traces of L1 are marked in violet.

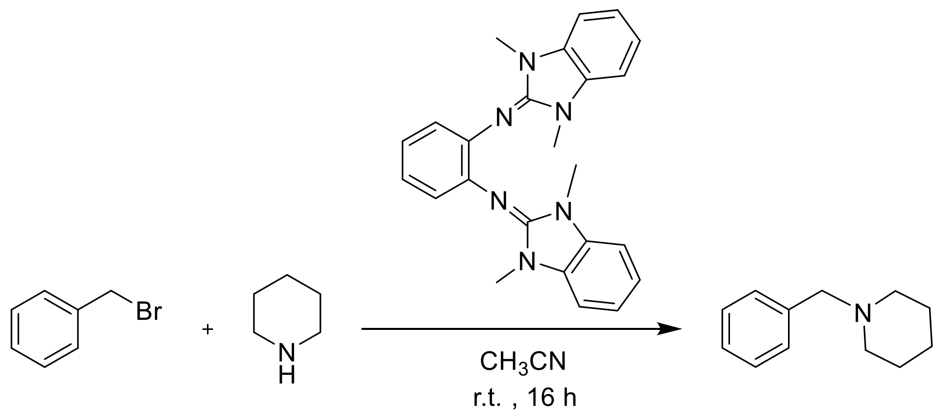

With L5 the reaction was carried out in the same manner, following the literature procedure[12] (45 mg, 113 µmol, 0.75 eq.). The resulting crude product was a yellow oil (43.2 mg, 162%), which contained impurities of protonated and neutral ligand, as well as remaining starting materials in the ^{1}H NMR spectrum. The product formation could also be observed in the ^{1}H NMR and MS.

^{1}H NMR (600 MHz, $CDCl_3$, 295 K): δ = 7.67 (d, J=7.5 Hz, 2 H), 7.47 (m, 3 H), 3.49-3.48 (q, J=7.0 Hz, 2 H), 3.43 (s, 2 H, CH_2), 1.21 (t, 7.0 Hz, 3 H) ppm.

MS (EI, DIP): *m/z* = 174.1257 [M-H] (calc.: 174.1288), 91.0531 [$C7H7^{\bullet}$] (calc.: 91.0548).

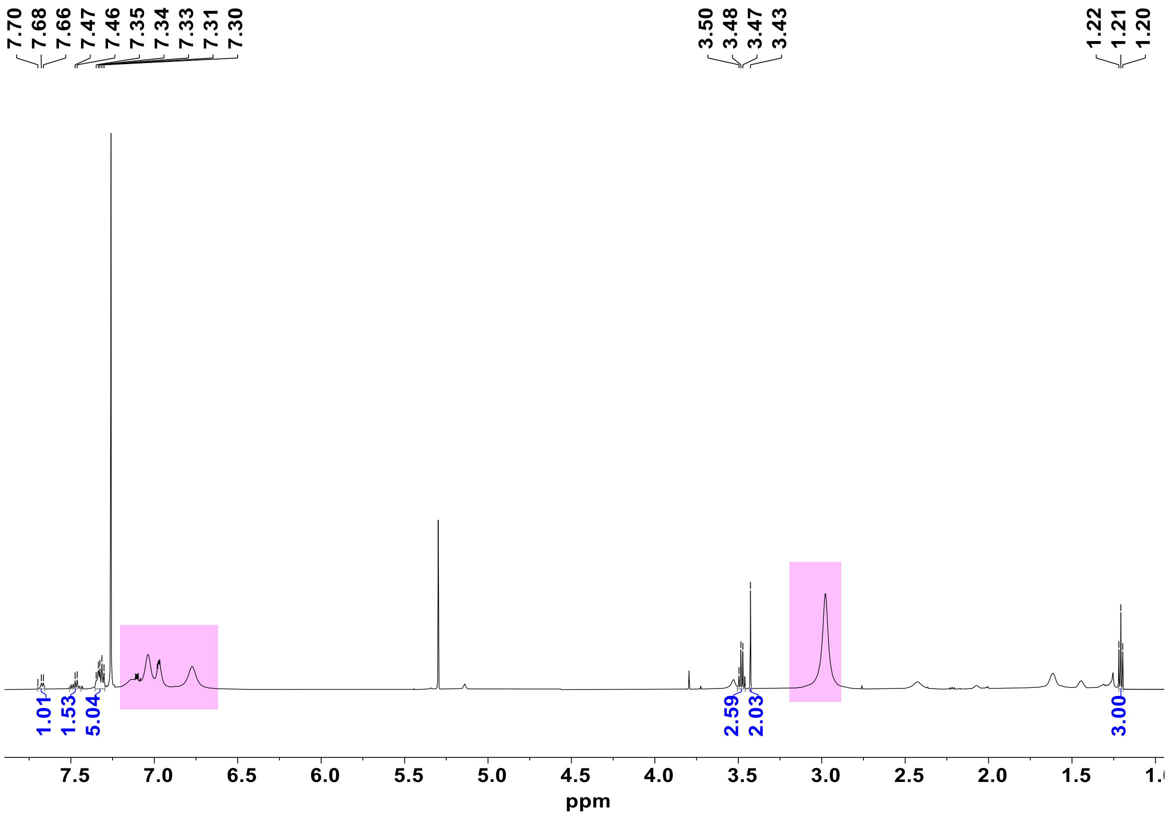

Figure 23: ^{1}H NMR spectrum in $CDCl_3$ after extraction, remaining traces of neutral and protonated L5 are marked in violet.

L2

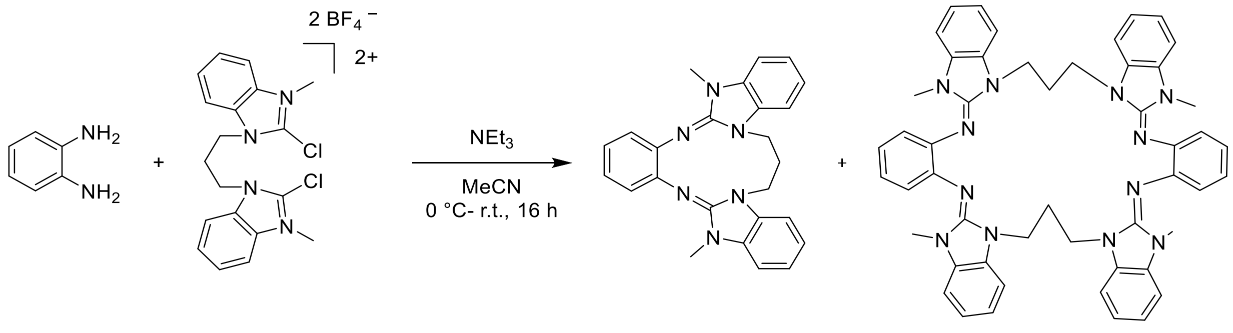

2(BF$_4$)$_2$ **L2** **L2$_{Macro}$**

Compound **2(BF$_4$)$_2$** (837 mg, 1.53 mmol, 1.10 eq.) was suspended in MeCN at 0 °C (30.0 mL). NEt_3 (1.93 mL, 1.40 g, 13.9 mmol, 10.0 eq.) and *o*-phenylenediamine were added slowly (150 mg, 1.39 mmol, 1.00 eq.) at 0 °C. The reaction mixture was warmed to r.t. while stirring for 16 h. The solvent was removed *in vacuo* and the residue was dissolved in HCl_{aq} (10 mL, 15 %). $NaOH_{aq}$ (10 mL, 30 %) was added while stirring. The aqueous phases were extracted first with Et_2O (3x10 mL) and then with DCM (5x10 mL). The solvent of the dichloromethane phase was removed *in vacuo*. The residue was repeatedly dissolved using HCl_{aq} (10 mL, 15 %) and $NaOH_{aq}$ (10 mL, 30 %) and reextracted using DCM (5·10 mL). After removal of the solvent of the organic phase *in vacuo*, and recrystallization out of toluene, a yellow solid was isolated (L2:L2$_{Macro}$ = 1.5:1) in a 82% yield however, this value should be considered an approximation due to remaining toluene.

^{1}H NMR L2 (600 MHz, CD_2Cl_2): δ = 6.97-6.94 (m, 2 H, CH_{Ar}), 6.89-6.85 (m, 2 H, CH_{Ar}), 6.70-6.66 (m, 2 H, CH_{Ar}), 6.61-6.59 (m, 2 H, CH_{Ar}), 6.56 (d, J=7.7 Hz 2 H, CH_{Ar}), 3.86 (m, 4 H, CH_2), 3.62 (t, 2 H, J=7.9 Hz, CH_2), 3.29 (s, 6 H, CH_3) ppm.

^{1}H NMR L2$_{Macro}$ (600 MHz, CD_2Cl_2): δ = 7.61-7.59 (m, 2H, CH_{Ar}), 7.04-7.02 (m, 6H, CH_{Ar}), 6.76-6.72 (m, 4H, CH_{Ar}), 6.42-6.41 (m, 6H, CH_{Ar}), 6.36-6.34 (m, 6H, CH_{Ar}), 3.55 (m, 8H, CH_2), 3.20 (s, 12H, CH_3), 2.95 (m, 4H, CH_2) ppm.

^{13}C NMR (101 MHz, CD_2Cl_2) δ = 153.86, 132.63, 131.79, 131.26, 128.96, 128.15, 125.23, 123.48, 122.34, 121.35, 120.77, 120.69, 120.14, 119.86, 119.55, 119.13, 116.31, 107.12, 106.61, 105.67, 105.58, 42.94, 27.77, 26.81, 25.51 ppm.

MS (Maldi$^+$ in DCTB): *m/z* = 409.2126 ([L2+H]$^+$, calc.: 409.2135), 817.4172 ([L2$_{Macro}$]$^+$, calc: 817.4198).

EA (C, H, N in %) (x 0,5 DCM) $C_{25}H_{24}N_6$: calc.: C 67.93, H 5.59, N 18.64
found: C 67.79, H 5.42, N 17.79.

CV (DCM, 100 mV/s, vs Fc^+/Fc): $E_{Ox}{}^1$=25 mV, $E_{Ox}{}^2$=260 mV, $E_{Ox}{}^3$=605 mV, E_{Red}=120 mV.

UV/Vis spectrum (DCM): λ (ε) = 228 (33662), 315 (18046) nm (M^{-1} cm^{-1}).

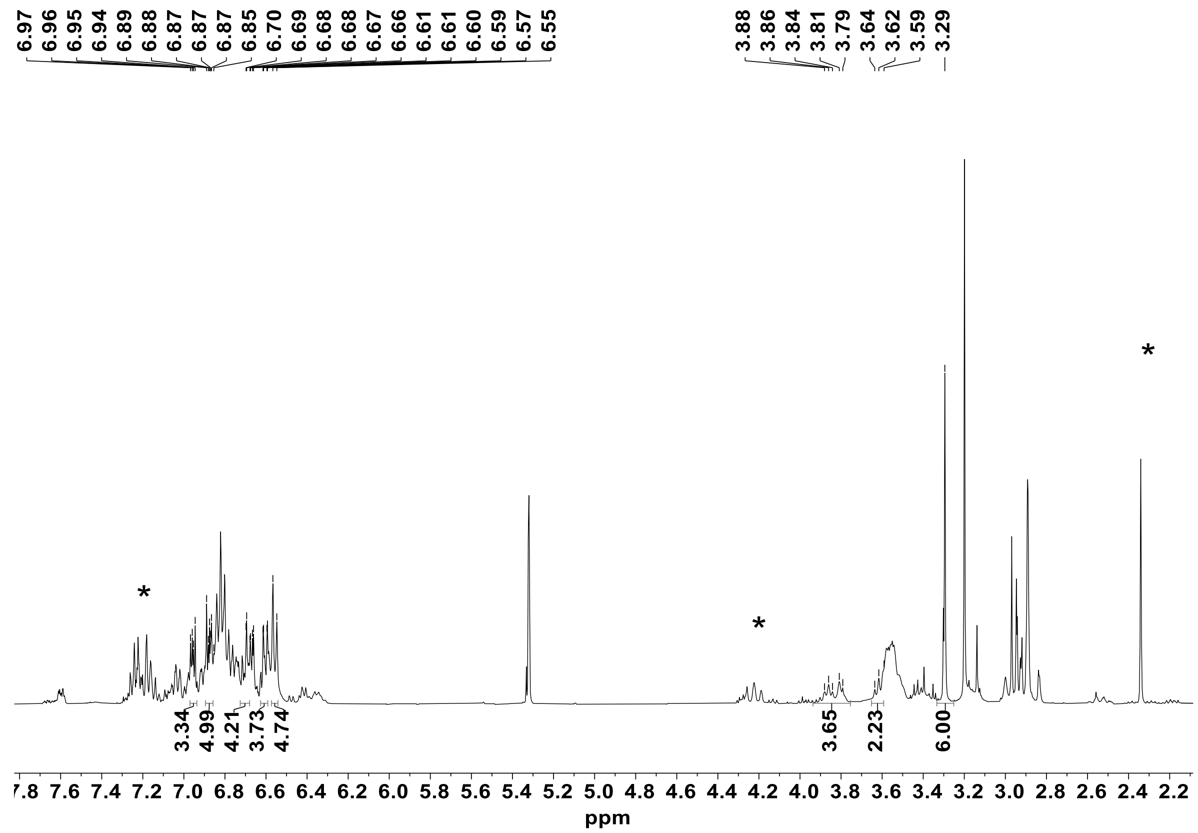

Figure 24: ^{1}H NMR (400 MHz, CD_2Cl_2) indicating the signals of L2, *toluene, urea traces. As the aromatic signals overlap with the macrocyclic signals, integrals may not include the right amount of H atoms.

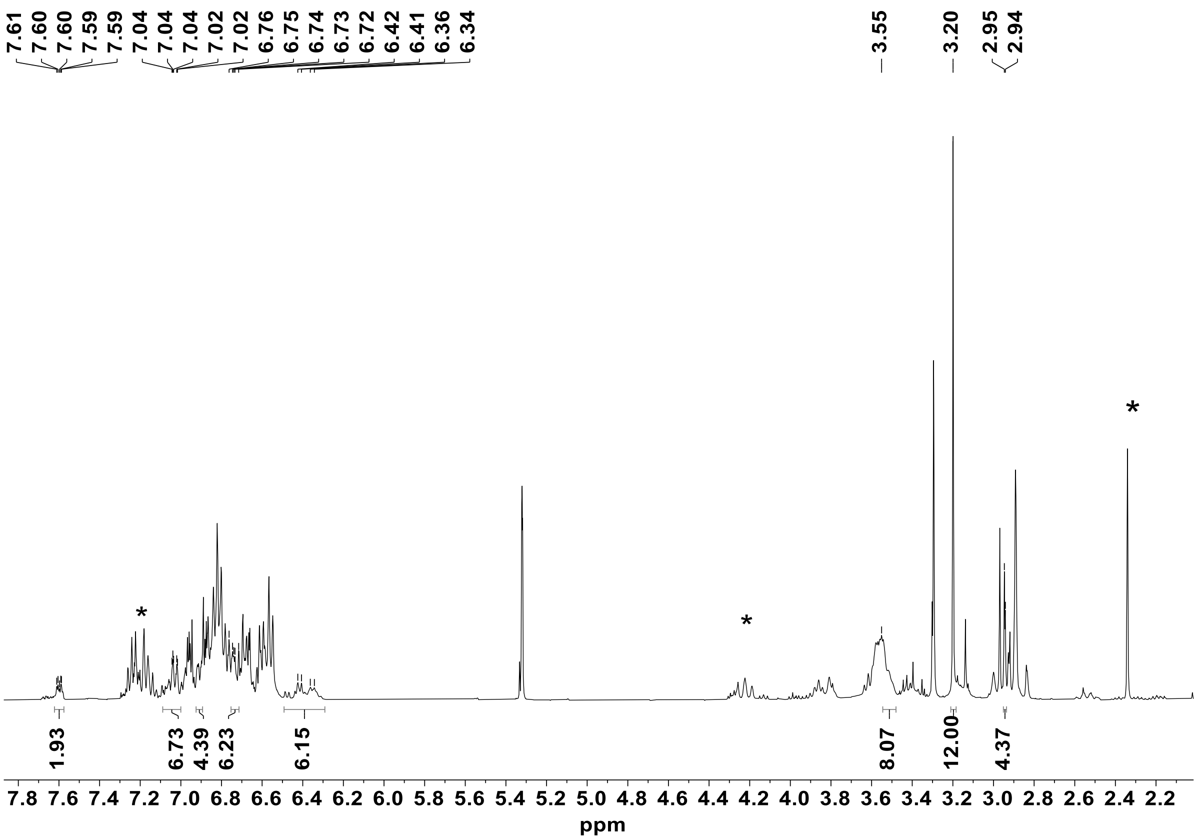

Figure 25: ^{1}H NMR (600 MHz, CD_2Cl_2) indicating the signals of $L2_{Macro}$, * toluene, urea traces.

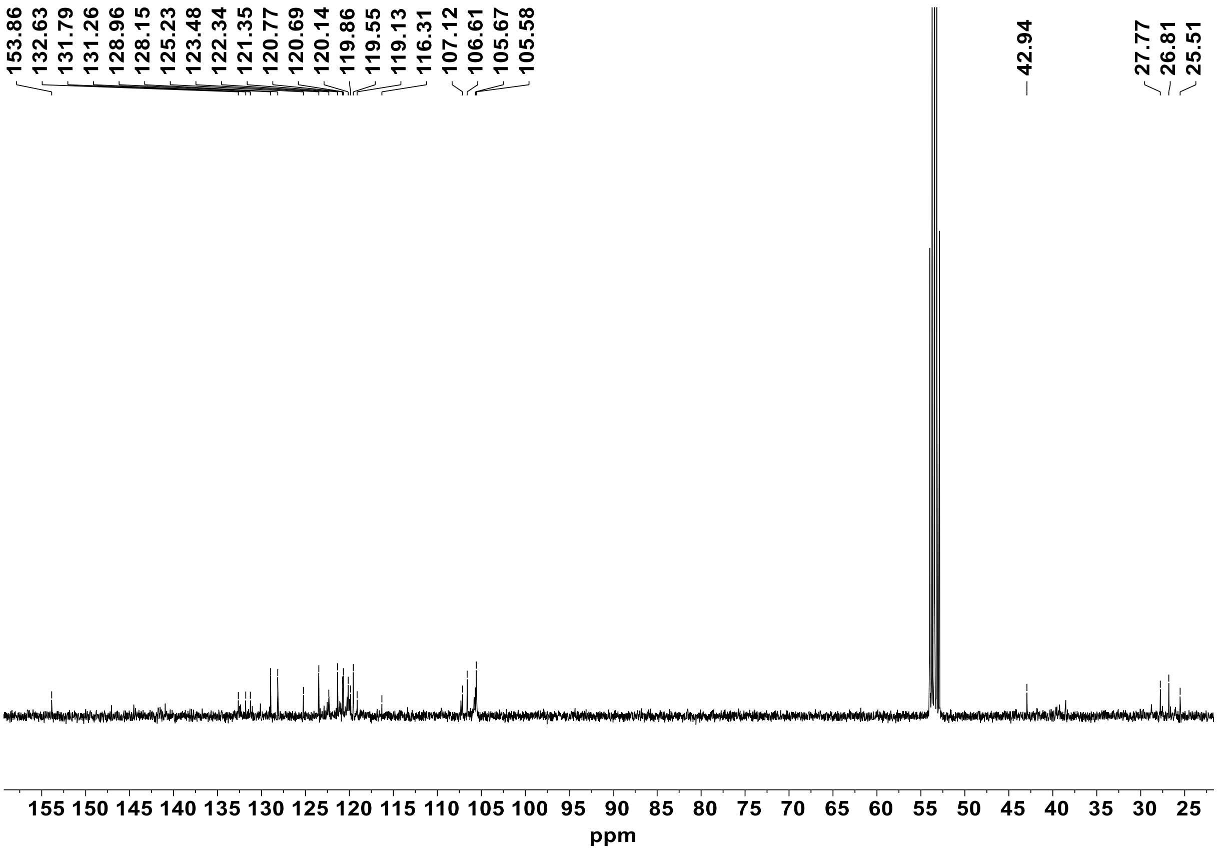

Figure 26: ^{13}C NMR (101 MHz, CD_2Cl_2) of L2.

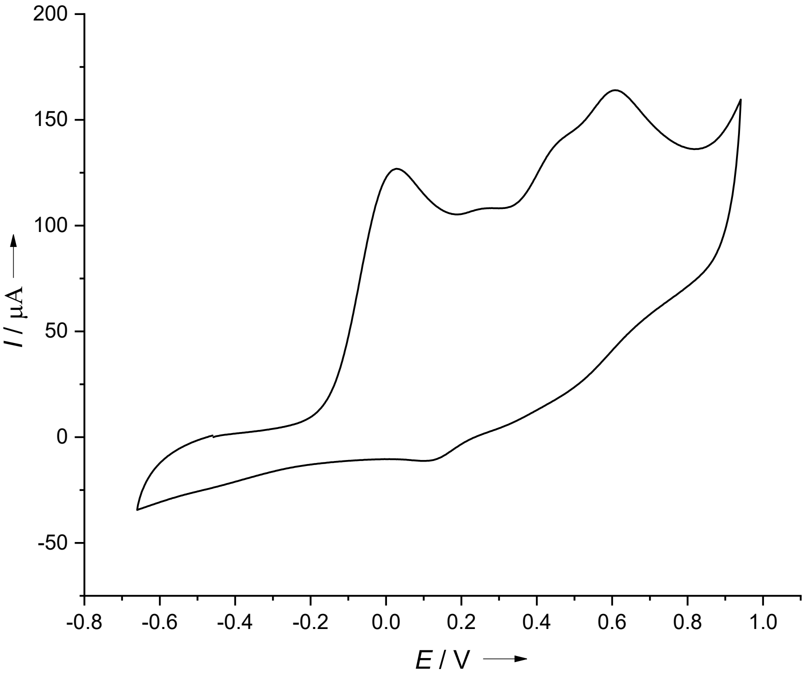

Figure 27: CV curve of **L2** (Fc/Fc$^+$ reference, 100 mV/s, *n*Bu_4NPF_6, Ag/AgCl electrode, DCM).

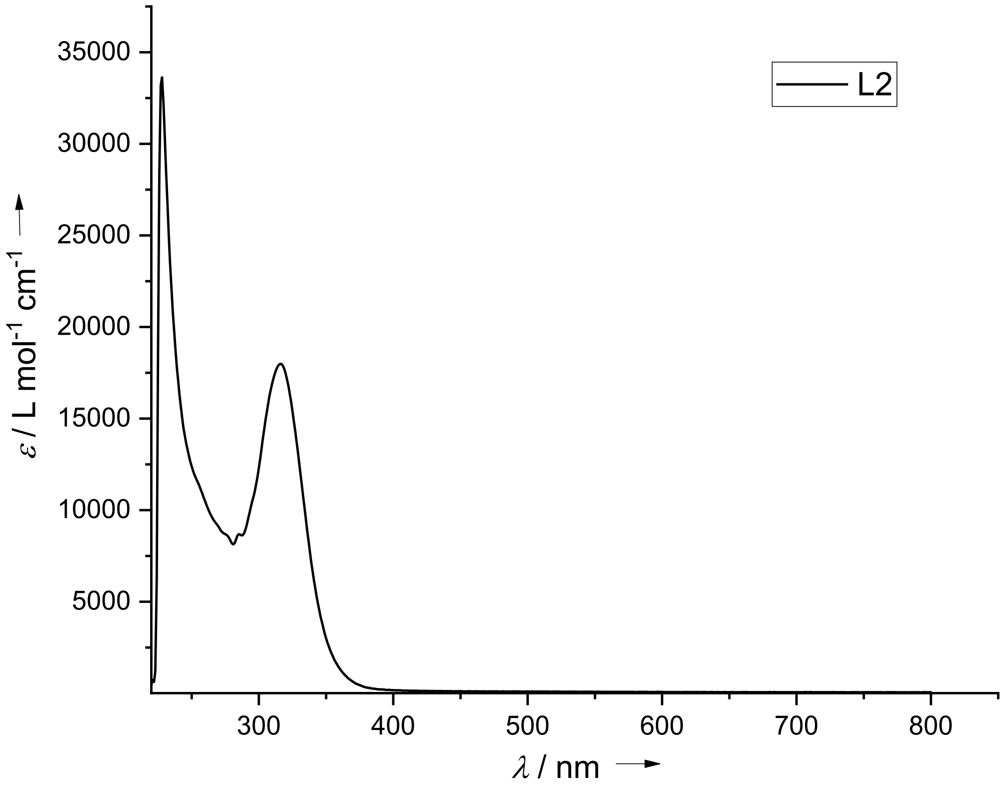

Figure 28: UV/Vis spectrum of **L2** in DCM.

(L2+2H)Cl₂

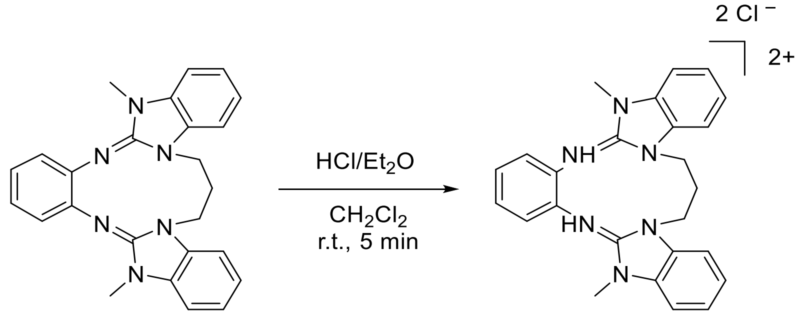

L2 (380 mg, 930 mmol, 1.00 eq.) was dissolved in DCM (4.0 mL) and HCl in diethylether (0.7 mL, 2.00 M) was added. The resulting precipitate was filtered and dried *in vacuo*. **(L2+2H)Cl₂** could be obtained as a colorless solid in quantitative yield. Crystals for structural analysis could be obtained through diffusion of diethylether in a saturated methanol solution.

^{1}H-NMR (600 MHz, D_2O): δ = 7.01-6.99 (m, 4 H, CH_{Ar}), 6.86-6.84 (m, 4 H, CH_{Ar}), 6.73-6.72 (m, 4 H, CH_{Ar}), 3.35 (bs, NH), 2.31-2.30 (m, 2 H, CH_2), 2.22-2.21 (t, 4 H, CH_2), 2.15 (s, 6 H, CH_3) ppm.

MS (ESI^+ in MeOH): *m/z* = 409,2125 ($[L+H]^+$, calc.: 409,2135).

L3

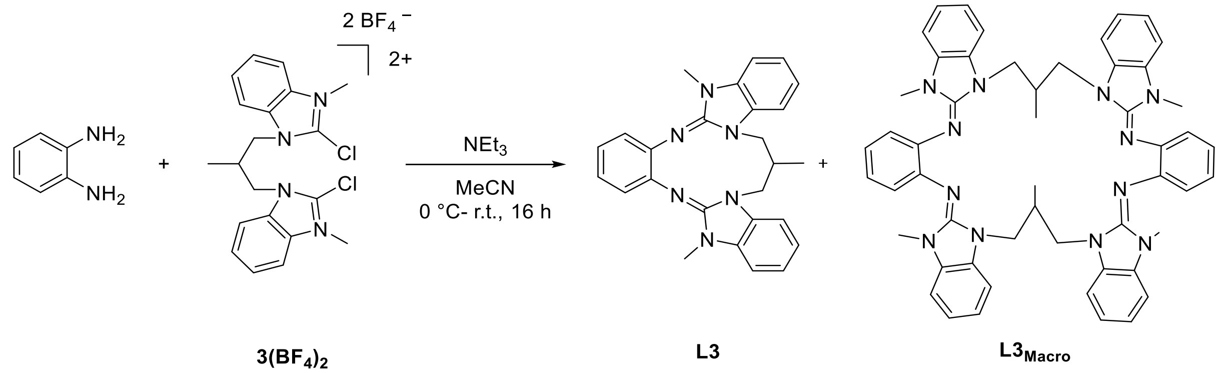

Compound **$3(BF_4)_2$** (1.00 g, 1.77 mmol, 1.20 eq.) was suspended in MeCN (40 mL) and cooled to 0°C. *o*-Phenylenediamine (160 mg, 1.48 mmol, 1.00 eq) was added and NEt_3 (2.04 mL, 1.49 g, 14.8 mmol, 10.0 eq.) slowly given to the solution. After 16 h the solvent was removed *in vacuo.* The residue was dissolved in HCl_{aq} (10.0 mL, 15%). $NaOH_{aq}$ (10.0 mL, 30%) was added while stirring. The aqueous phases were extracted first with Et_2O (3·10 mL) and then with DCM (5x10 mL). The solvent of the dichloromethane phase was removed *in vacuo*. The residue was repeatedly dissolved using HCl_{aq} (10.0 mL, 15%) and $NaOH_{aq}$ (10.0 mL, 30%) and reextracted using DCM (5x10 mL). After removal of the solvent of the organic phase *in vacuo*, and recrystallization out of toluene a yellow solid was isolated ($L3:L3_{Macro}$ ca.10:1) in a 79% yield however, this value should be considered an approximation due to remaining toluene.

^{1}H NMR (600 MHz, CD_2Cl_2): δ = 7.00 (dd, *J* = 5.8, 3.5 Hz, 2 H), 6.92 (dd, *J* = 5.8, 3.6 Hz, 2 H), 6.84 (td, *J* = 7.7, 1.0 Hz, 2 H), 6.71 (d, *J* = 1.1 Hz, 2 H), 6.61 (d, *J* = 7.8 Hz, 2 H), 6.54 (d, *J* = 7.8 Hz, 2 H), 4.03 (dd, *J* = 15.1, 11.6 Hz, 1 H), 3.39 (dd, *J* = 15.2, 3.4 Hz, 4 H), 3.23 (s, 6 H), 2.97 (s, 3 H) ppm.

^{13}C NMR (101 MHz, CD_2Cl_2): δ =128.96, 128.15, 125.23, 122.50, 121.36, 120.67, 120.16, 119.51, 107.03, 106.71, 105.50, 49.31, 21.14 ppm.

MS (ESI$^+$ in DCM): *m/z* = 423.2296 ([M+H]$^+$, calc.: 423.2297), 845.4518 ([$L2_{Macro}$+H]$^+$; calc.: 845.4516).

EA (C, H, N): (x1 DCM) $C_{26}H_{26}N_6$: found: C 64.80, H 6.05, N 16.55.
calc: C 63.91, H 5.56, N 16.56.

UV/Vis (DCM): λ (ε) = 316 (9195), 227 (17576) nm (M^{-1} cm^{-1}).

CV (DCM, [*n*Bu_4N][PF_6], 100 mV/s, vs Fc^+/Fc): $E_{Ox}{}^1$=17 mV, $E_{Ox}{}^2$=616 mV, $E_{Ox}{}^3$=996 mV, E_{Red}=97 mV.

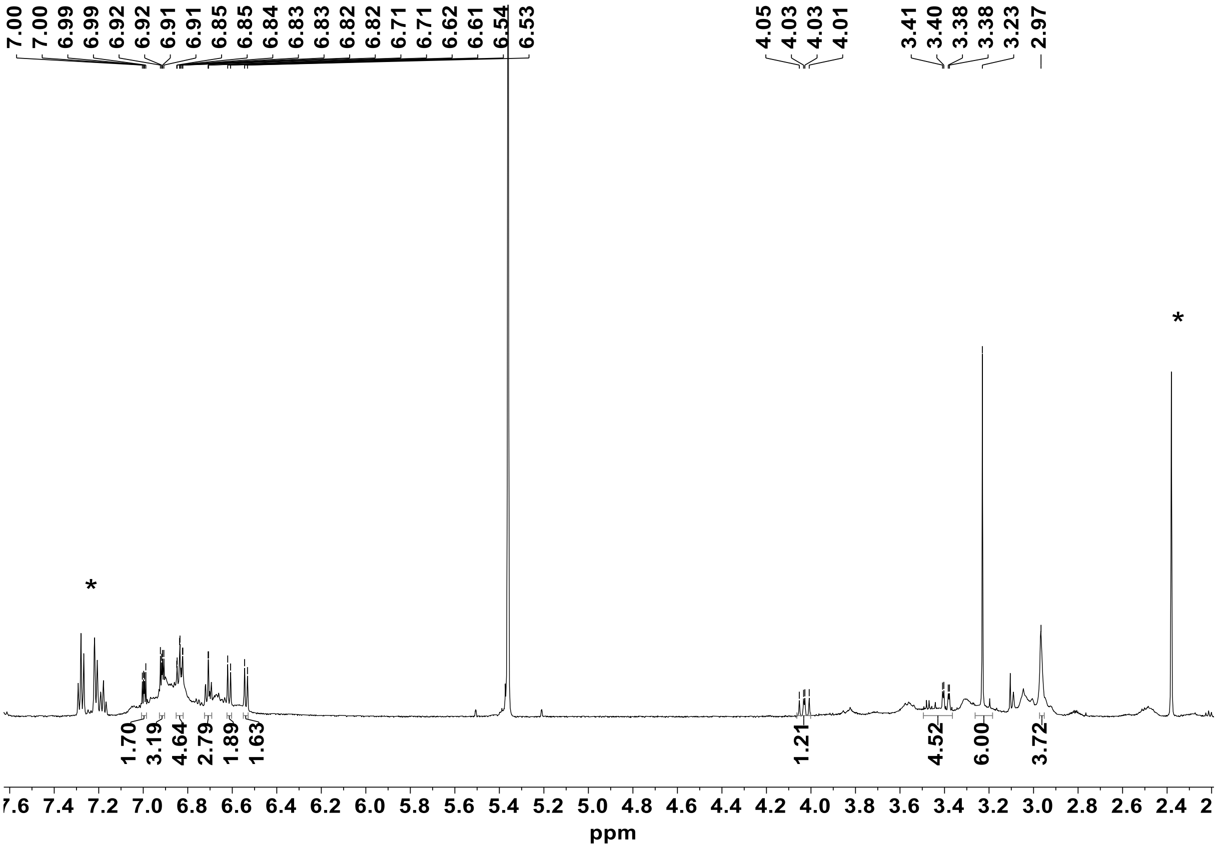

Figure 29: ^{1}H-NMR spectrum (600 MHz in CD_2Cl_2).of **L3**. As the aromatic signals overlap with the macrocyclic signals, integrals may not include the right amount of H atoms. *toluene

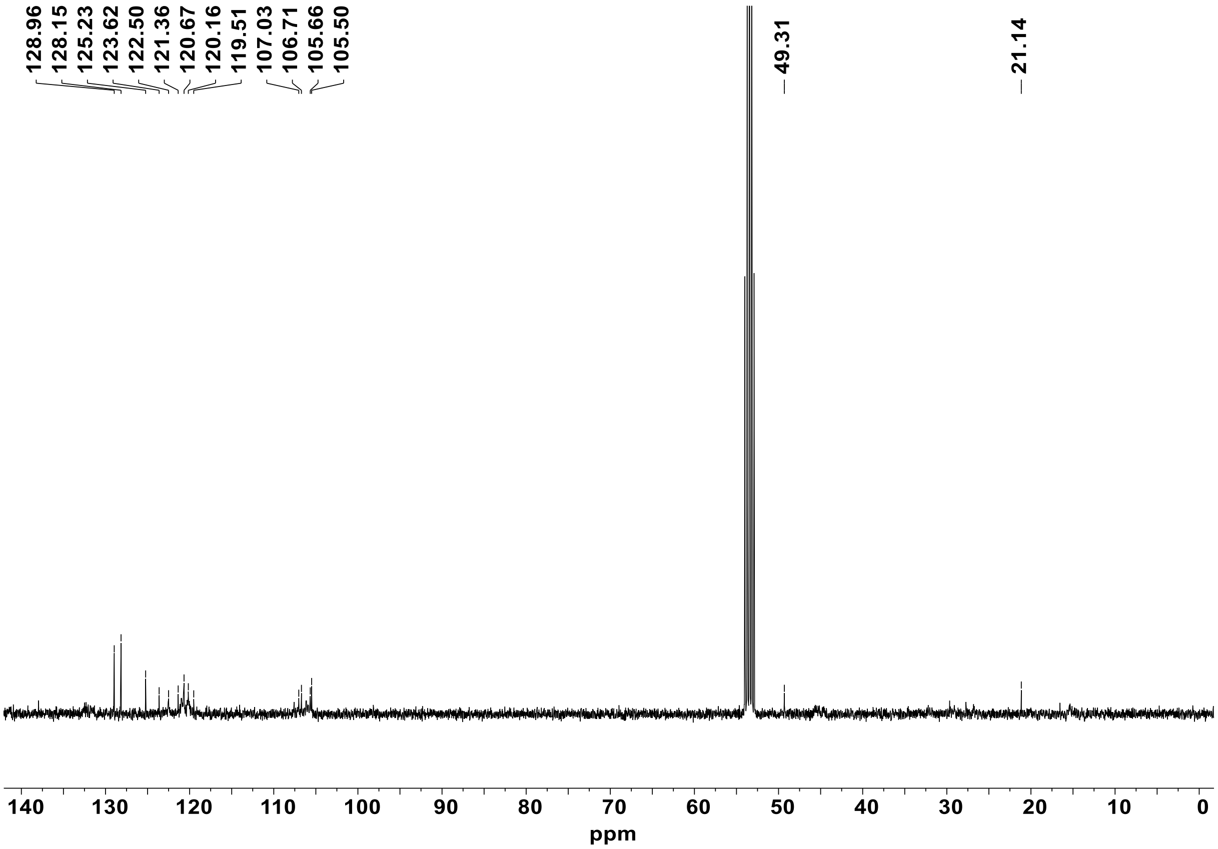

Figure 30: ^{13}C NMR (101 MHz, CD_2Cl_2) of **L3**.

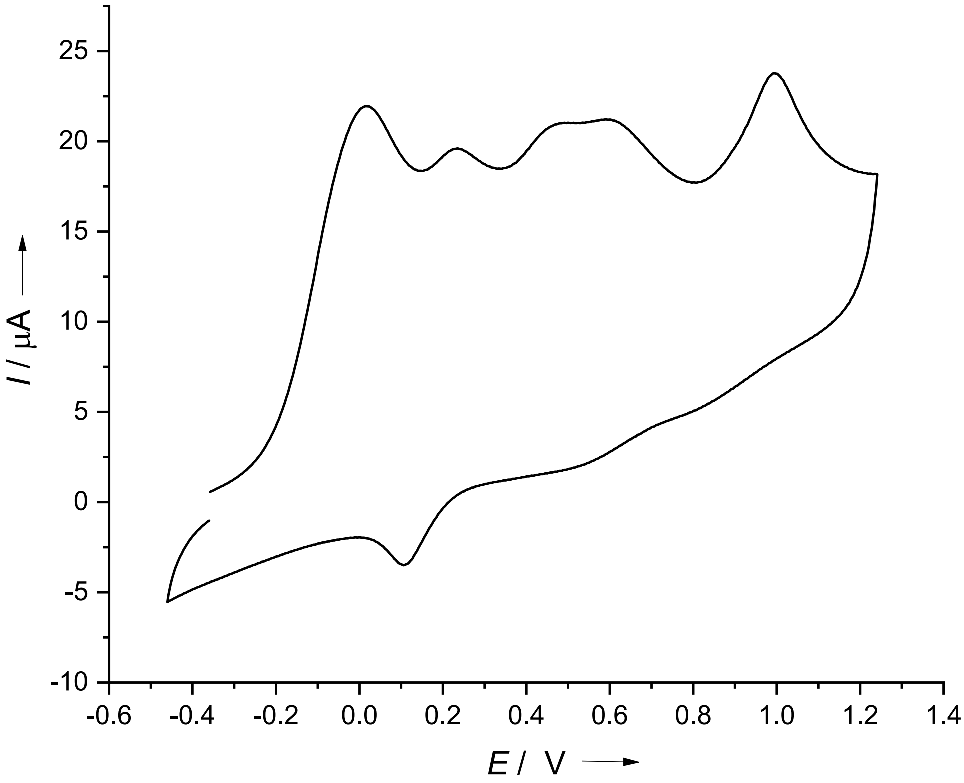

Figure 31: CV curve of **L3** (Fc/Fc+ reference, 100 mV/s, *n*Bu_4NPF_6, Ag/AgCl electrode, DCM).

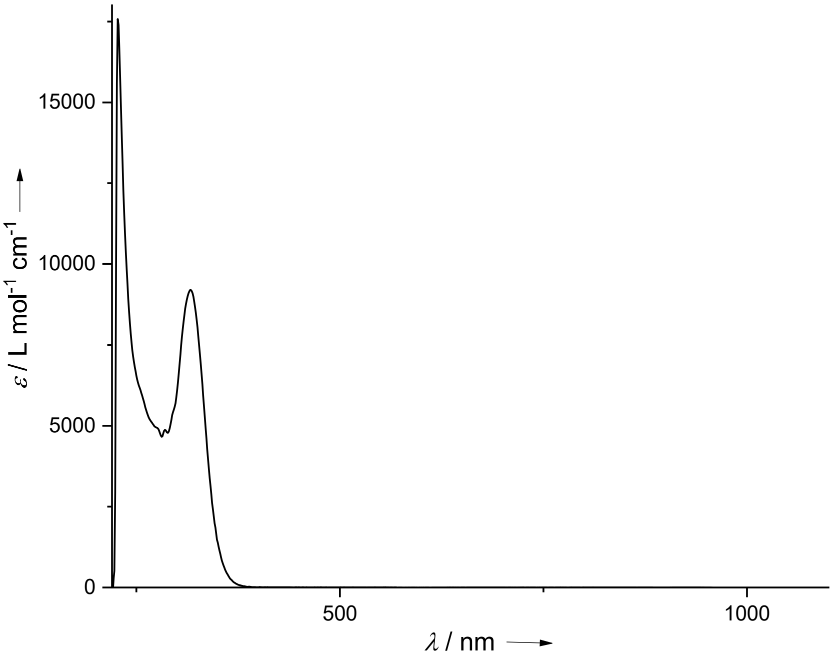

Figure 32: UV/Vis spectrum of **L3** in DCM.

(L3+2H)Cl₂

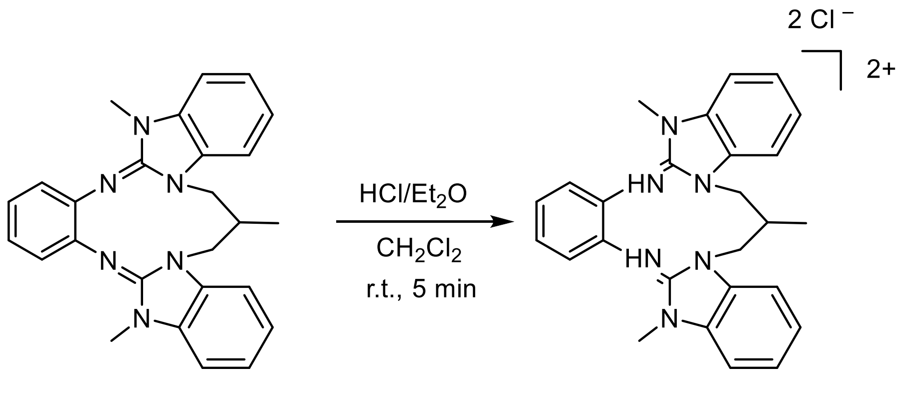

L3 **(L3+2H)Cl2**

L3 (20.0 mg, 47.3 µmol, 1.0 eq) was dissolved in DCM (4.0 mL) and HCl in diethylether (0.5 mL, 2.0 M) was added. The resulting precipitate was filtered and dried *in vacuo* **(L3+2H)Cl₂** could be obtained after washing with diethylether (2 x 2 mL) as a colorless solid in quantitative yield. Crystals for structural analysis could be obtained through diffusion of diethylether in a saturated methanol solution.

^{1}H-NMR (600 MHz, D_2O): δ =7.67 (d, *J* = 5.6 Hz, 3 H), 7.27 (d, *J* = 4.2 Hz, 3 H), 7.23 (d, *J*=8.2 Hz, 2 H), 7.11 (dt, *J* = 8.6, 4.6 Hz, 4 H), 3.63 (s, 6 H), 3.18 (q, *J* = 7.4 Hz, 2 H), 1.26 (t, *J*= 7.3 Hz, 2 H) ppm.

MS (ESI^+ in DCM): *m/z* = 423.2293 ($[L2+H]^+$, calc.: 423.2297).

L4

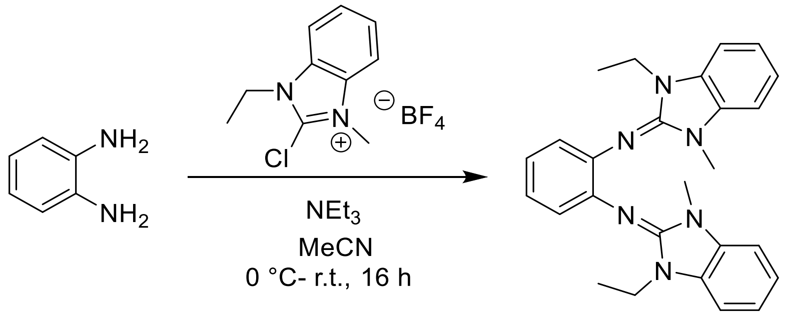

Ethylmethylbenzimidazoliumtetrafluoroborate (548 mg, 1.94 mmol, 2.00 eq.) was suspended at 0 °C in MeCN (30 mL). *o*-Phenylenediamine (100 mg, 0.92 mmol, 1.00 eq.) and NEt_3 (1.28 mL, 0.93 g, 9.25 mmol, 10.0 eq.) were slowly added to the reaction mixture at 0 °C. While stirring for 48 h the suspension was allowed to warm to room temperature. The solvent was removed *in vacuo* and the residue dissolved in aqueous HCl(15%). Aqueous NaOH solution was added and the solution was extracted with diethylether (3 x 20 mL). After removal of the solvent of the organic phase *in vacuo*, the product was suspended with n-pentane in the ultrasonic bath. After isolation of the precipitate, **L4** was obtained (220 mg, 0.51 mmol, 56 %) as a colorless solid. Crystals for structural analysis were obtained out of a saturated diethylether-solution.

^{1}H NMR (200 MHz, CD_2Cl_2): δ = 6.97-6.88 (m, 8 H, CH_{Ar}), 6.75-6.70 (m, 4 H, CH_{Ar}), 3.59-3.48 (q, J = 7.9 Hz, 4 H, CH_2) 2.99 (s, 6 H, CH_3), 0.96-0.89 (t, J = 7.9 Hz, 6 H, CH_3) ppm.

^{13}C{^{1}H} NMR (151 MHz, CD_2Cl_2, 295 K): *δ* = 144.5 (2 C, C_q), 141.7 (2 C, C_q), 132.7 (2 C, C_q), 131.4 (2 C, C_q), 122.21 (2 C, CH), 120.9 (2 C, CH), 120.1 (2 C, CH), 119.9 (2 C, CH), 105.7 (2 C, CH), 105.7 (2 C, CH), 38.4 (2 C, CH_2), 28.8 (2 C, CH_3), 12.48 (2 C, CH_3) ppm.

MS (ESI^+ in DCM): 425.2446 [L+H], (calc: 425.2448 [L+H])

EA (C, H, N in %) $C_{26}H_{28}N_6$: calc..: C 73.56, H 6.65, N 19.80

found: C 73.40, H 6.79, N 19.34.

CV (DCM, [*n*Bu_4N][PF_6], 100 mV s^{-1}, vs. Fc/Fc^+): $E_{1/2}{}^1$ = - 94 mV $E_{1/2}{}^2$ = 210 mV.

UV/Vis-spectrum (DCM): *λ* (ε) = 229 (14988), 319 (10510) nm (M^{-1} cm^{-1})

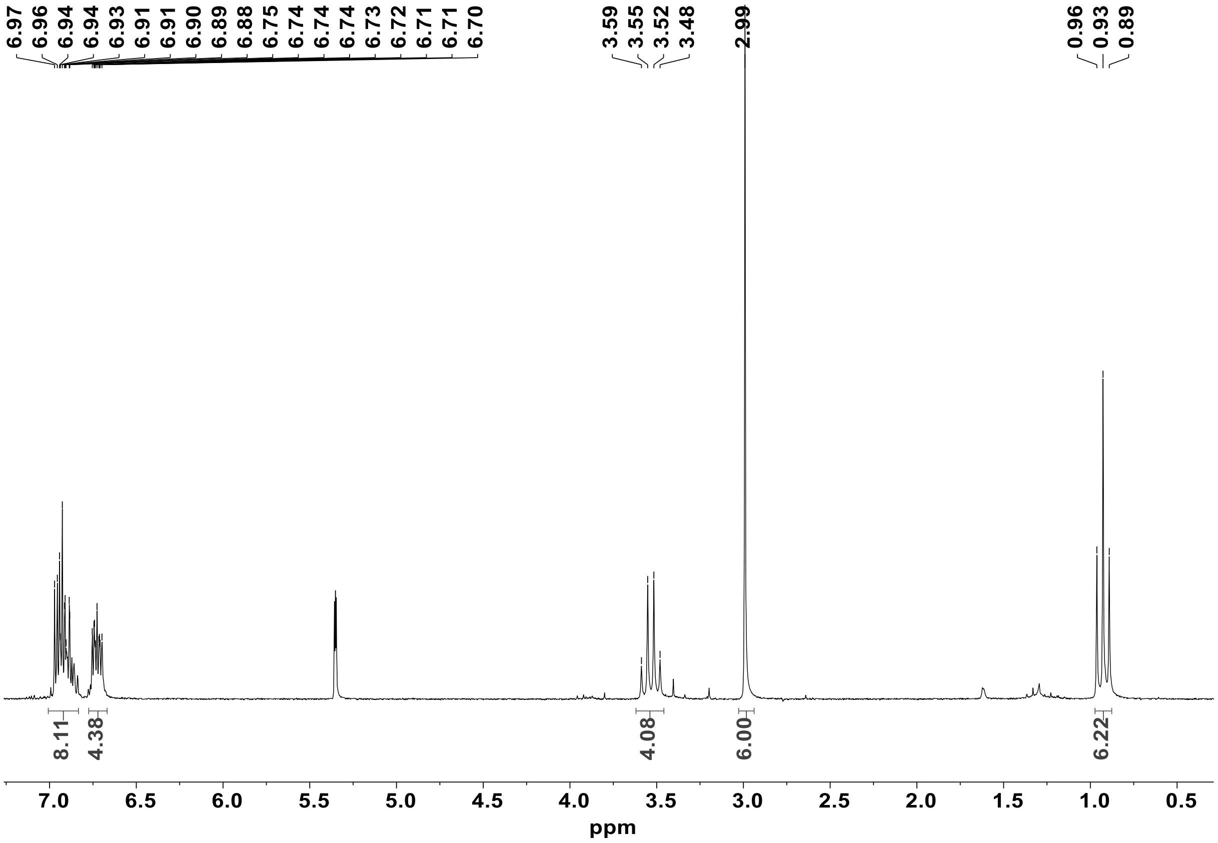

Figure 34: ^{1}H NMR spectrum of **L4**, measured at 200 MHz in CD_2Cl_2.

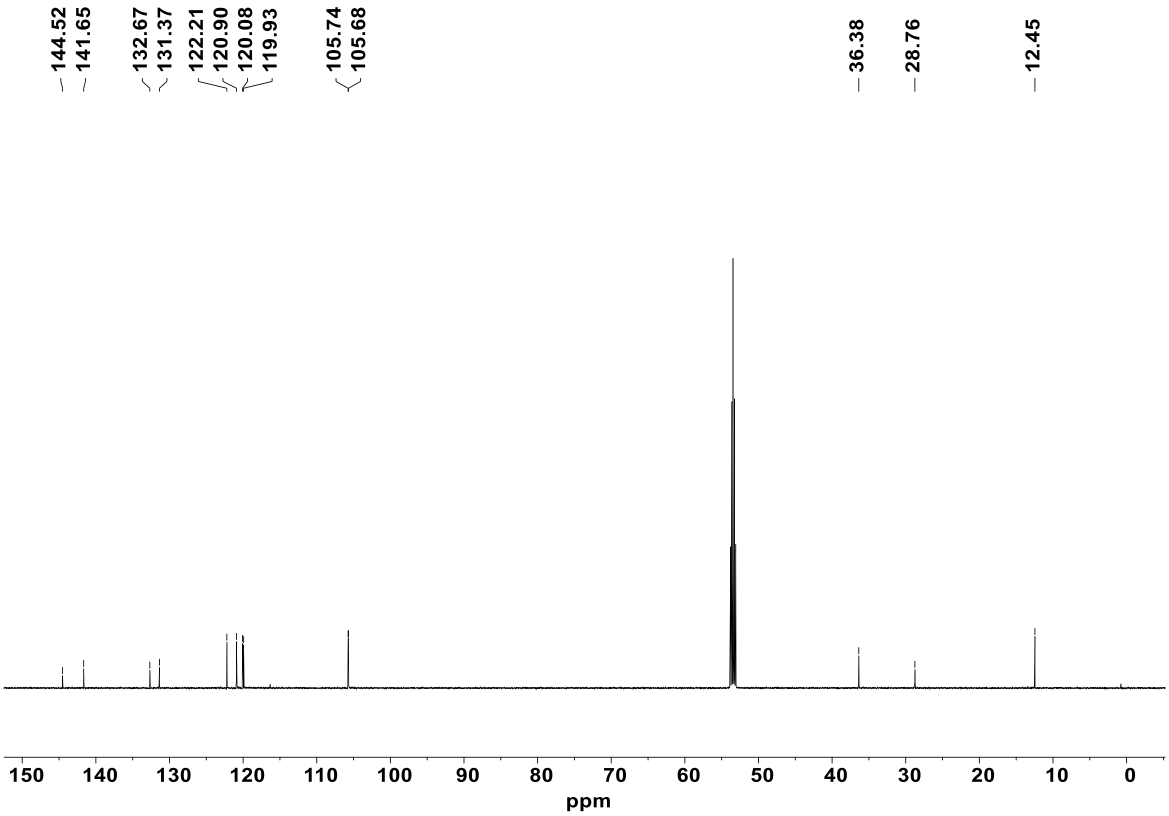

Figure 35: ^{13}C NMR spectrum of **L4**, measured in CD_2Cl_2 at 295 K at 150.92 MHz.

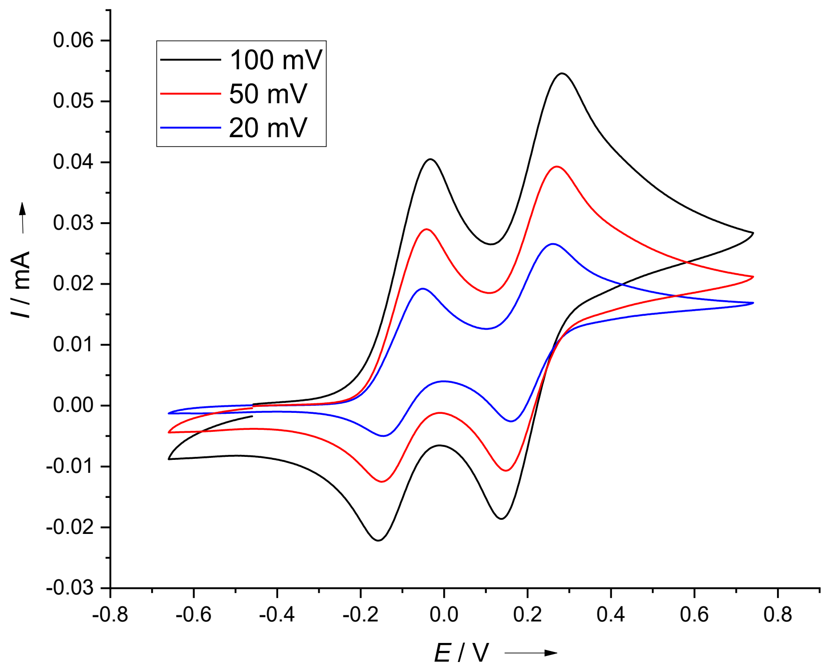

Figure 36: CV curve of **L4** (Fc/Fc$^+$ reference, *n*Bu_4NPF_6, Ag/AgCl electrode, DCM).

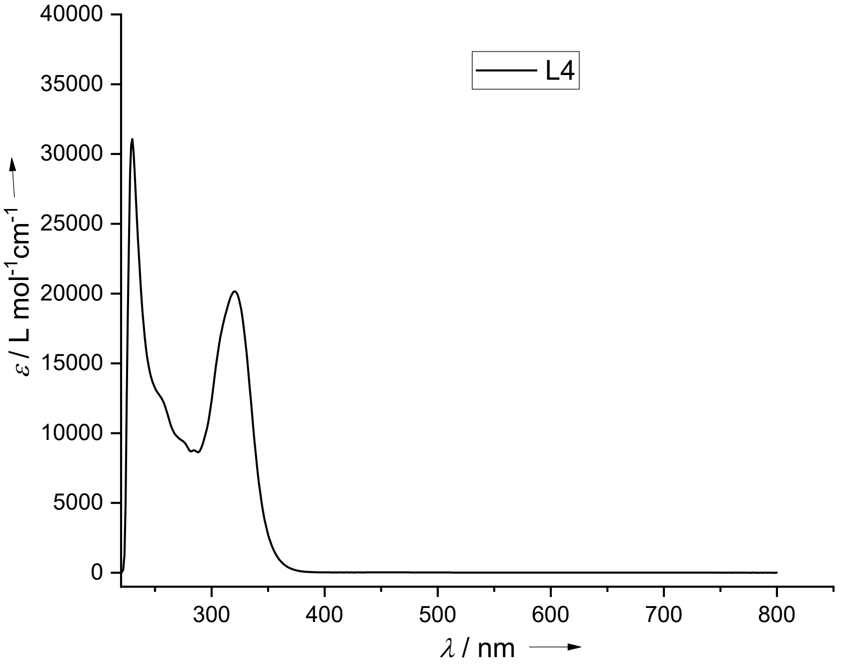

Figure 37: UV/Vis spectrum of **L4**$^-$ in MeCN.

(L4+2H)Cl₂

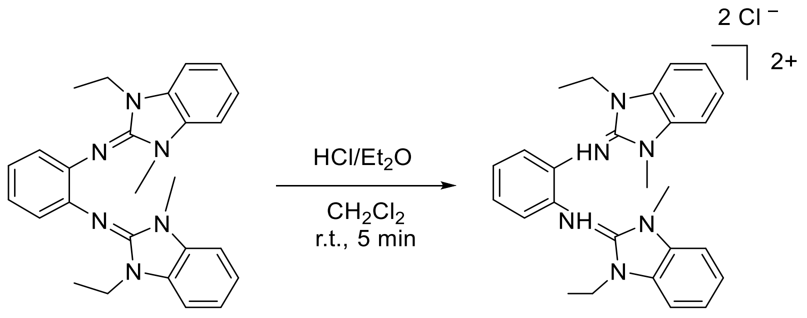

L4 **(L4+2H)Cl$_2$**

L4 (10.0 mg, 25.4 mmol, 1.00 eq.) was dissolved in DCM (4.0 mL) and HCl in diethylether (0.20 mL, 2.00 M) was added. The resulting precipitate was filtered and dried *in vacuo*. **(L4+2H)Cl$_2$** could be obtained as a colorless solid in quantitative yield. Crystals for structural analysis could be obtained through diffusion of diethylether in a saturated methanol solution.

^{1}H NMR (600 MHz, D_2O): δ = 7.56-7.53 (m, 10 H, CH_{Ar}), 7.49-7.48(m, 2 H, CH_{Ar}), 3.67 (q, J = 7.0 Hz, 4 H, CH_2), 3.32 (s, 6 H, CH_3), 1.17 (t, J = 7.1 Hz, 6 H, CH_3) ppm.

MS (ESI+ in DCM): 425.2446 [L+H], (calc: 425.2448 [L+H])

Oxidation von L4

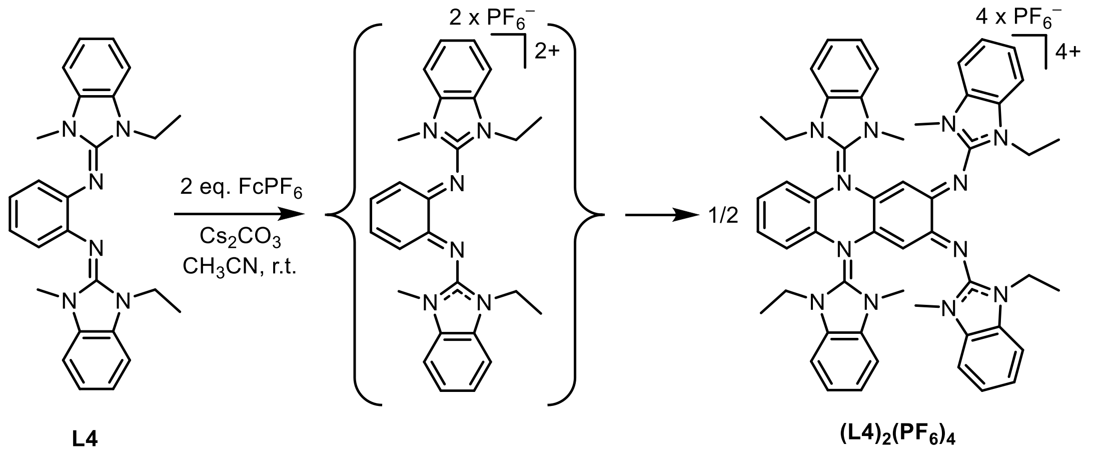

Cs_2CO_3 (15.4 mg, 47.1 µmol, 1.00 eq.) and ferrocenium hexafluorophosphate (37.4 mg, 113 µmol, 2.40 eq.) were dissolved in CH_3CN (5 mL). L4 (40.0 mg, 94.2 µmol, 2.00 eq.) was added and the reaction mixture was stirred for 3 h at room temperature. Then, the suspension was filtrated, the solvent was removed *in vacuo*, and the residue was washed with Et_2O (3 x 3 mL) to give 63.0 mg (44.3 µmol, 94%) product.

^{1}H NMR (600 MHz, CD_3CN): δ = 8.08 – 8.01 (m, 4 H), 7.88 – 7.84 (m, 4 H), 6.97 – 6.93 (m, 6 H), 6.90 (dt, J = 7.1, 3.6 Hz, 3 H), 6.86 (dd, J = 5.8, 3.6 Hz, 1 H), 6.82 – 6.76 (m, 6 H), 5.80 (d, J = 13.5 Hz, 2 H), 4.67 (q, J = 6.8, 6.4 Hz, 4 H), 4.18 (d, J = 10.5 Hz, 7 H), 3.49 – 3.44 (m, 4 H), 2.96 (d, J = 7.4 Hz, 8 H), 1.55 (t, J = 7.3 Hz, 6 H), 0.82 (t, J = 7.1 Hz, 6 H) ppm.

^{13}C{^{1}H} NMR (151 MHz, CD_2Cl_2, 295 K): δ = 145.14, 139.87, 132.20, 131.48, 130.86, 130.07,120.09, 127.92, 120.52 (d, J = 23.6 Hz), 114.73, 114.28, 108.34, 106.33, 67.80, 41.82 36.29, 28.57, 12.02 ppm.

MS (ESI+ in DCM): 991.4246 [M-PF_6], (calc: 991.4220 [M-PF_6]).

UV/Vis-spectrum (DCM): λ (ε) = 296 (17293), 310 (16869), 349 (10103, shoulder) nm (M^{-1} cm^{-1}).

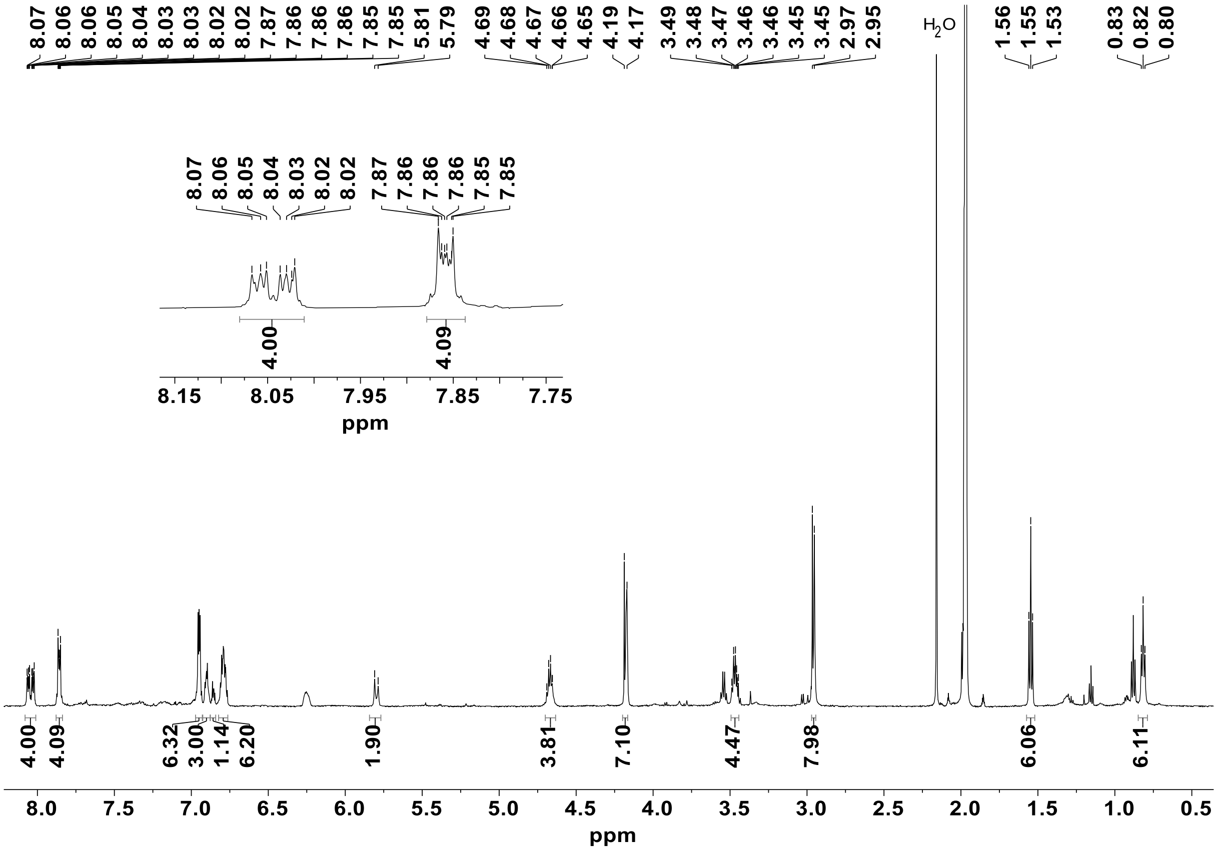

Figure 38: ^{1}H NMR spectrum of **$(L4)_2(PF_6)_4$**, measured at 600 MHz, 295 K in CD_3CN.

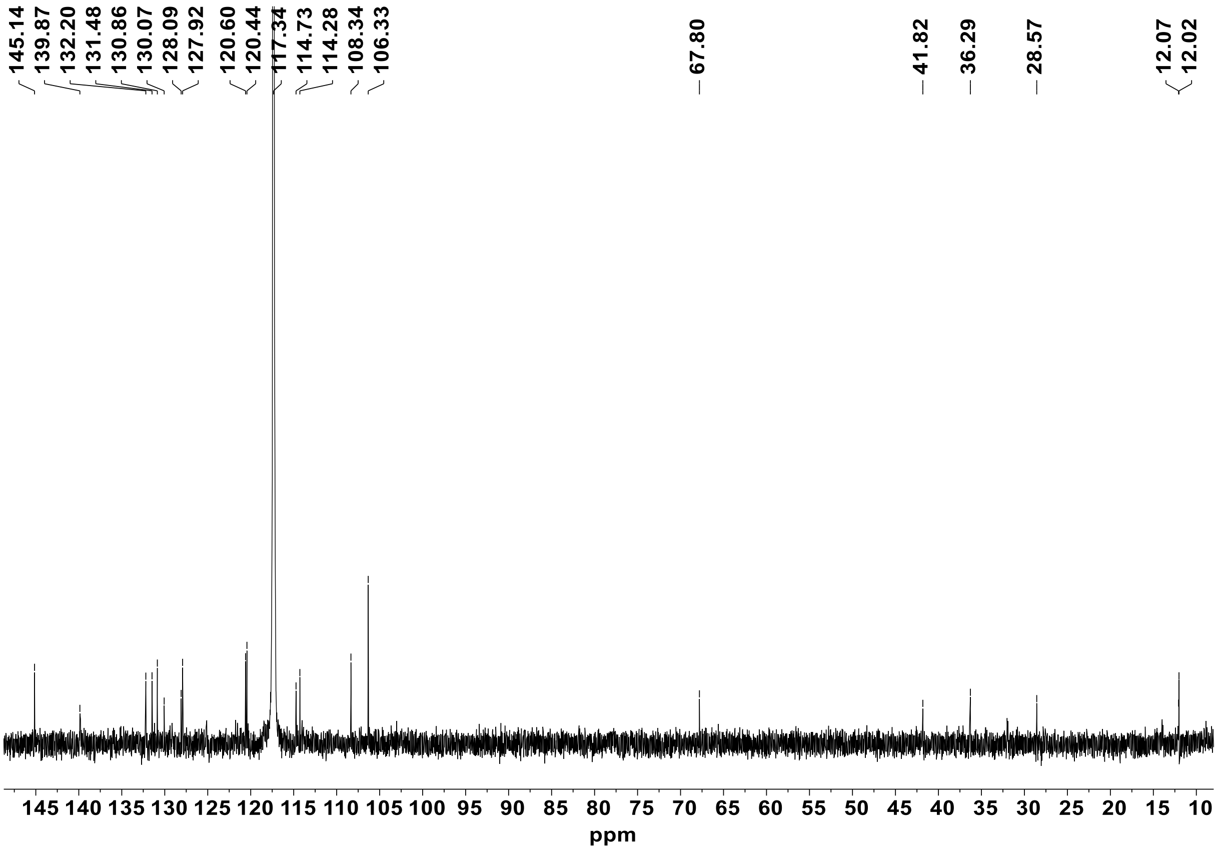

Figure 39: ^{13}C NMR spectrum of **$(L4)_2(PF_6)_4$**, measured in CD_3CN at 295 K at 150.92 MHz.

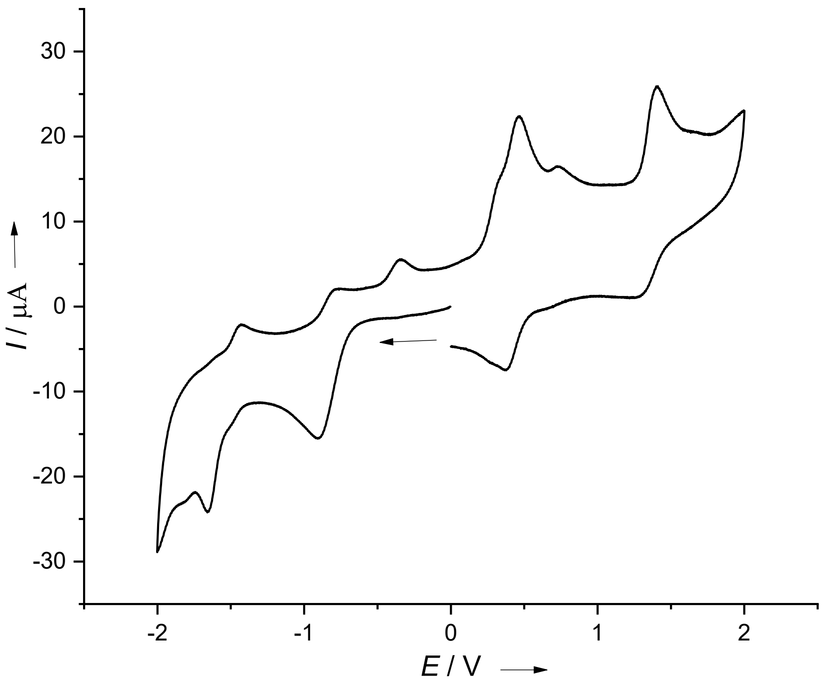

Figure 40: Cyclic voltammetry curve for **$(L4)_2(PF_6)_4$** in CH_3CN (Ag/AgCl reference electrode, $n$$Bu_4NPF_6$ as supporting electrolyte). Potentials given vs. the ferrocenium/ferrocene (Fc^+/Fc) reference redox couple.

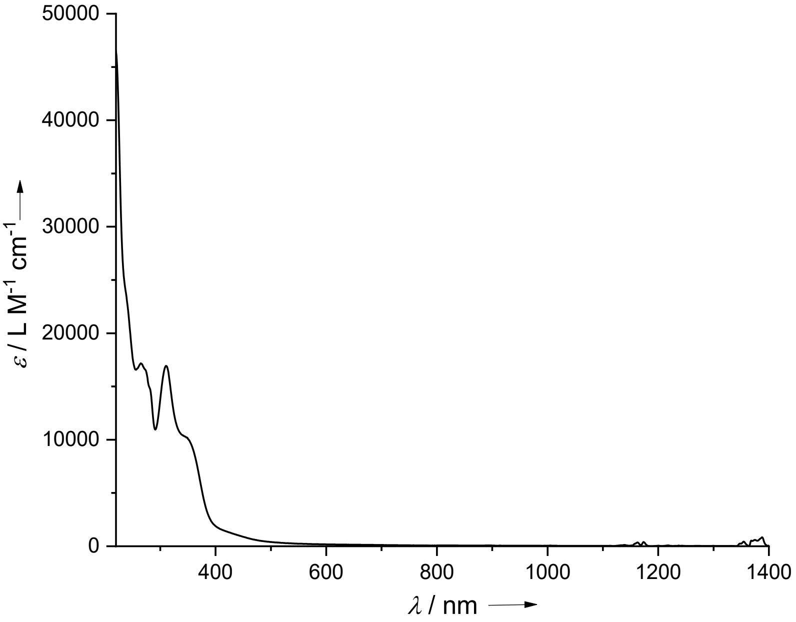

Figure 41: UV/VIS of **$(L4)_2(PF_6)_4$** in MeCN.

L5

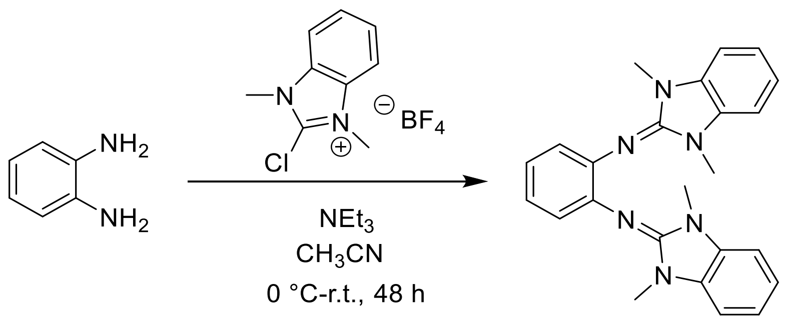

Dimethylbenzimidazoliumtetrafluoroborate (521 mg, 1.94 mmol, 2.10 eq.) was suspended at 0 °C in MeCN (30 mL). *o*-phenylenediamine (100 mg, 0.92 mmol, 1.00 eq.) and NEt_3 (1.28 mL, 0.93 g, 9.25 mmol, 10.0 eq.) were slowly added to the reaction mixture at 0 °C. While stirring for 48 h the suspension was allowed to warm to room temperature. The solvent was removed *in vacuo* and the residue dissolved in aqueous HCl (15%). Aqueous NaOH solution was added, and the solution was extracted with diethylether (3 x 20 mL). After removal of the solvent of the organic phase *in vacuo*, the product was suspended with n-pentane in the ultrasonic bath. After isolation of the precipitate, **L5** was obtained (264 mg, 0.7 mmol, 72 %) as a colorless solid.

^{1}H-NMR (400 MHz, CD_2Cl_2): δ = 6.94–6.91 (m, 6 H, CH_{Ar}), 6.84–6.82 (m, 2 H, CH_{Ar}), 6,67–6.65 (m, 4 H, CH_{Ar}), 2.90 (s, 6 H, CH_3) ppm.

^{13}C{^{1}H}-NMR (151 MHz, CD_2Cl_2, 295 K): δ = 145.8 (2 C, C_q), 141.65 (2 C, C_q), 132.2 (4 C, C_q),122.5 (4 C, CH), 120.9 (4 C, CH), 120.2 (4 C, CH), 105.6 (4 C, CH), 28.4 (4 C, CH_3) ppm.

MS (ESI^+ in DCM): 397.2135 [L+H], (calc.: 397.2135 [L+H]).

EA (C, H, N in %) $C_{24}H_{24}N_6$: calc.: C 72.70, H 6.10, N 21.20
found: C 72.50, H 5.85, N 21.33.

CV (DCM, [*n*Bu_4N][PF_6], 100 mV s^{-1}, vs. Fc/Fc^+): $E_{1/2}{}^1$ = - 87 mV $E_{1/2}{}^2$ = 191 mV.

UV/Vis spectrum (DCM): λ (ε) = 229 (31127), 319 (20104) nm (M^{-1} cm^{-1}).

Synthesized as reported previously [13].

Reduction of $L5_2(PF_6)_4$ with Cobaltocene

As reported previously [13], L5 dimerizes in almost quantitative yield in an oxidative substitution reaction when treated with ferrocenium hexafluorophosphate as an oxidation agent to give $L5_2(PF_6)_4$.

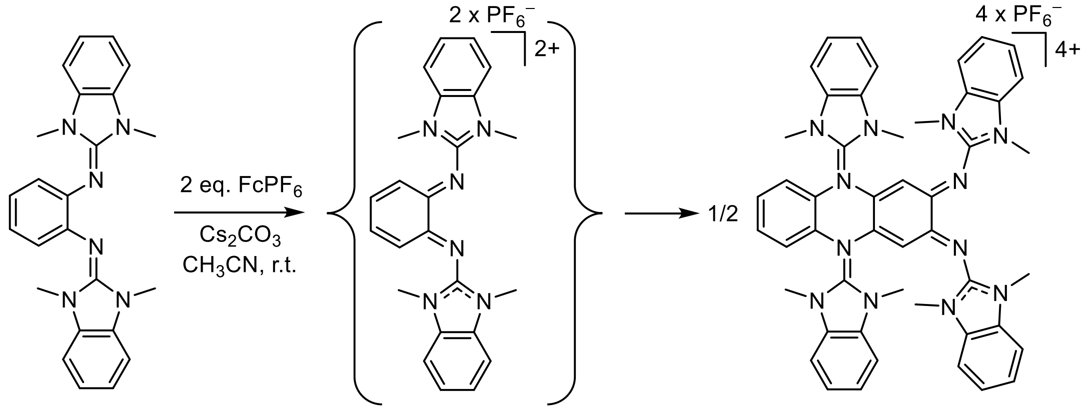

In consequence the reduction of $L5_2(PF_6)_4$ with cobaltocene was studied.

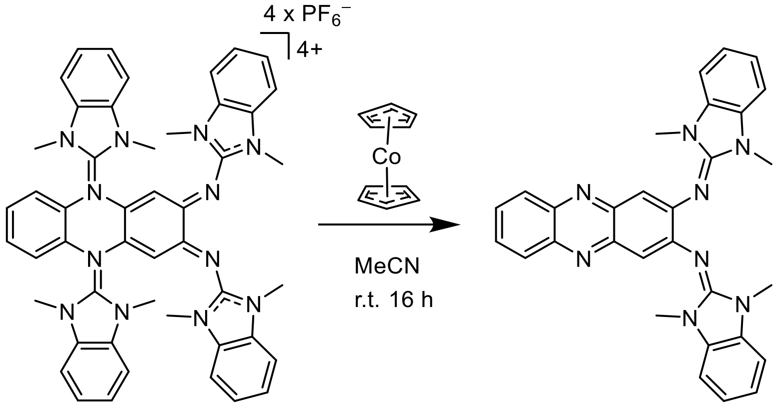

Cobaltocene (11.04 mg, 58.36 µmol, 4 eq.) and $L5(PF_6)_4$ (20 mg, 14.59 µmol, 1 eq.) were diluted in 4 mL of MECN. The reaction mixture was stirred at room temperature overnight (16 h). The solvent was removed *in vacuo* and the resulting red solid washed with hexane (3x2 mL) to give the product (5 mg, 10,21 µmol 70%). Crystals suitable for structural characterization were grown out of a saturated MeCN solution, overlayered with diethylether.

^{1}H NMR (600 MHz, CD_3CN) δ = 7.74 (dd, *J* = 7.1 Hz, 4 H), 7.29 (s, 2 H), 6.73 (dd, *J*= 2.9 Hz, 8 H), 2.91 (s, 12 H) ppm.

MS (MALDI in DCM): *m/z* = 498.2284 (calc 498.2280).

UV/Vis spectrum (DCM): λ (ε) = 463 (2399), 366 (4893), 311(10714), 264 (23982) nm (M^{-1} cm^{-1}).

The product still contains impurities through the cleavage of two of the guanidine functions as carbenes. Possibly following the "Wanzlick equilibrium" and forming the Cobaltocenium PF_6 salt as a side product. [14]

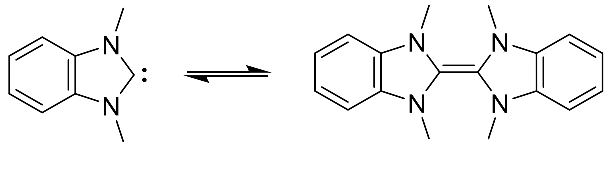

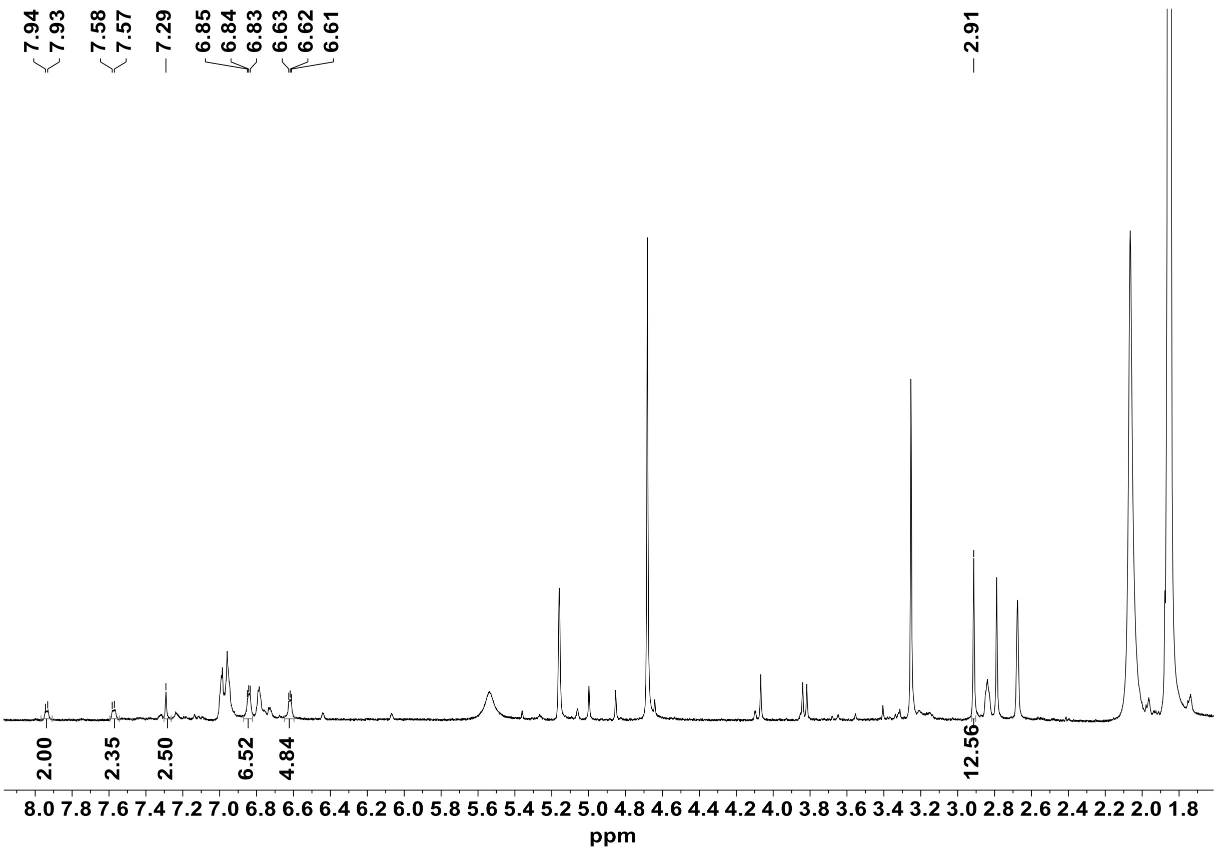

Figure 42: ^{1}H NMR spectrum, measured at 600 MHz in CD_3CN.

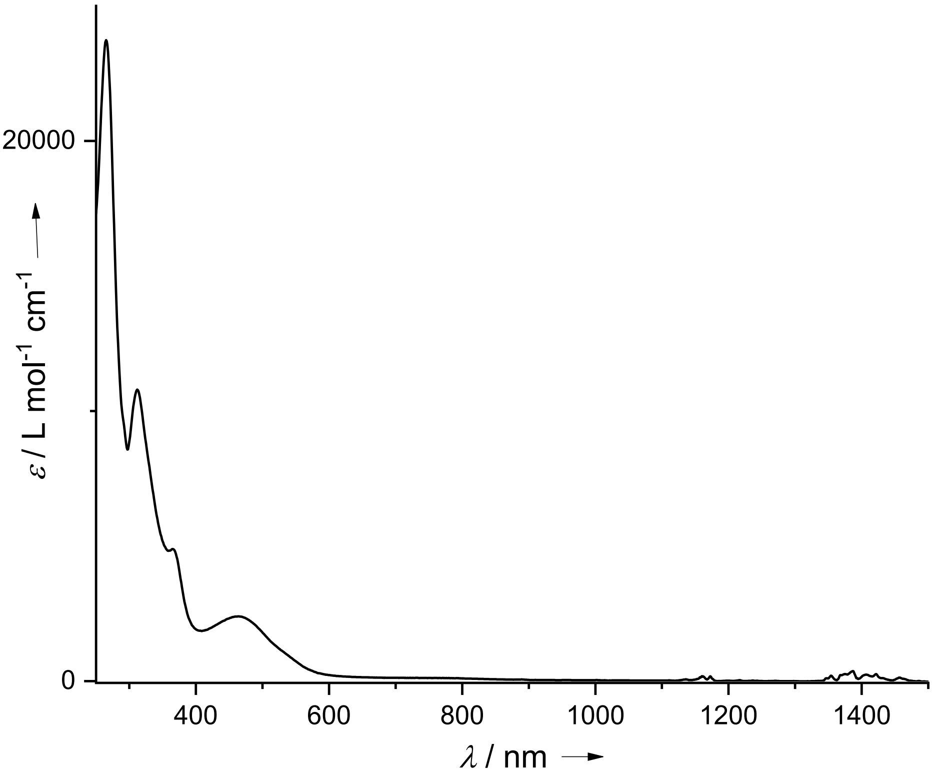

Figure 43: UV/Vis after reduction of $L5_2(PF_6)_4$ with cobaltocene.

The resulting product can also be synthesized in a similar procedure to L1-L5.

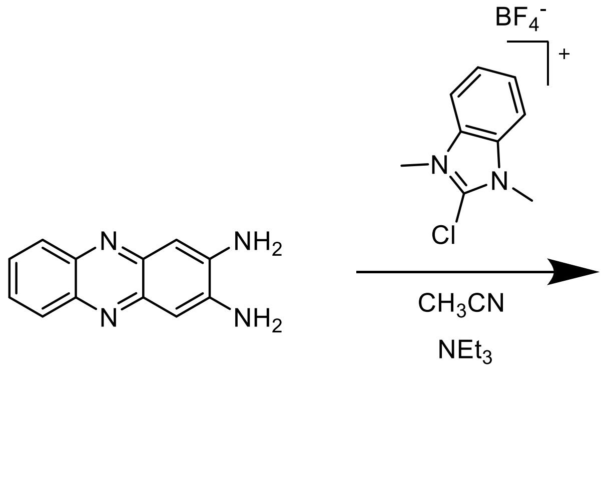

For the reaction, 383 mg of 2-chloro-1,3-dimethyl-1H-benzimidazol-3-ium tetrafluoroborate (1.43 mmol, 2.00 equiv.) was dissolved in 10 mL of acetonitrile, and the solution was cooled to 0°C. Subsequently, 2,3-diaminophenazine (150 mg, 713 µmol, 1.00 equiv.) and triethylamine (1.00 mL, 7.13 mmol, 10.0 equiv.) were added, upon which a distinct color change to an intense red was observed. After warming to room temperature, the reaction mixture was stirred continuously for 68 h. The resulting precipitate was separated by filtration and initially washed with acetonitrile (4 mL). This was followed by extraction with diethylether (3 × 3 mL) and a final wash with dichloromethane (15 mL). The residue was dried *in vacuo*.

The product was obtained in a yield of 98.9 mg (198.28 µm, 28%), however, this value should be considered approximate due to the presence of residual urea.

^{1}H NMR (600 MHz, $CDCl_3$) δ 7.93 (ddd, *J* = 277.9, 6.5, 3.5 Hz, 4 H), 7.59 (s, 2 H), 6.98 (dd, *J*=148.8, 2.9 Hz, 8 H), 3.11 (s, 12 H) ppm.

^{13}C{^{1}H}-NMR (151 MHz, CD_2Cl_2, 295 K): δ = 27.2 (4 C, CH_3), 106.9 (2 C, CH_2), 107.5 (4 C, CH_2), 114.4 (2 C, CH_2), 121.2 (4 C, CH_2), 127.9 (2 C, CH_2), 128.9 (2 C, CH_2), 130.3 (2 C, CH_2), 131.9 (2 C, C_q), 142.1 (2 C, C_q), 143.5 (2 C, C_q), 147.5 (2 C, C_q).

MS (MALDI in DCM): *m/z* = 498.2283 (calc 498.2280).

EA (C, H, N in %) (x1.5 DCM) $C_{30}H_{26}N_8$: calc.: C 60.44, H 4.67, N 17.90
found: C 61.01, H 5.26, N 17.82.

CV (DCM, [*n*Bu_4N][PF_6], 100 mV s^{-1}, vs. Fc/Fc^+): E_{pa} = 1,45 V.

UV/Vis spectrum (DCM): λ (ε) = 248 (shoulder, 41355), 280 (43852), 313 (63426), 460 (29805), nm ($M^{-1}cm^{-1}$).

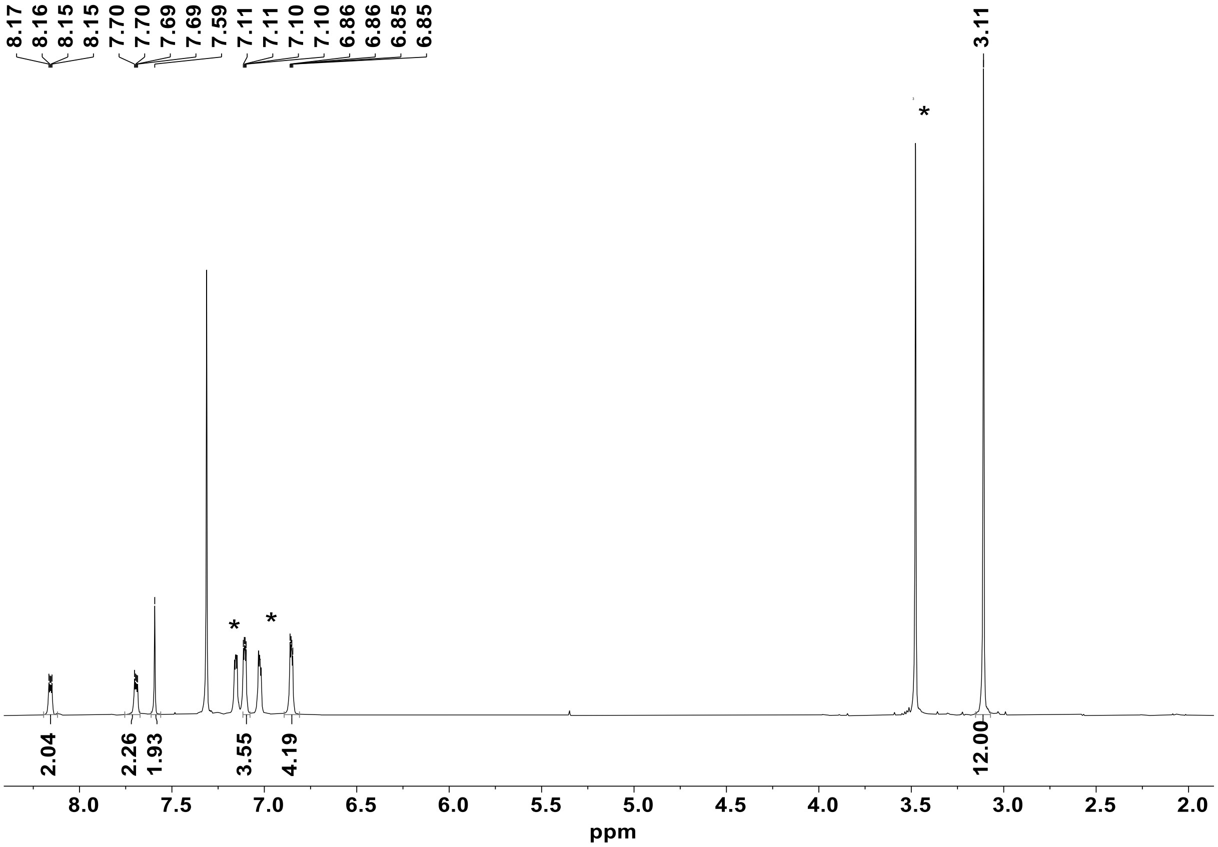

Figure 44: ^{1}H NMR, measured at 600 MHz in $CDCl_3$, *residual urea.

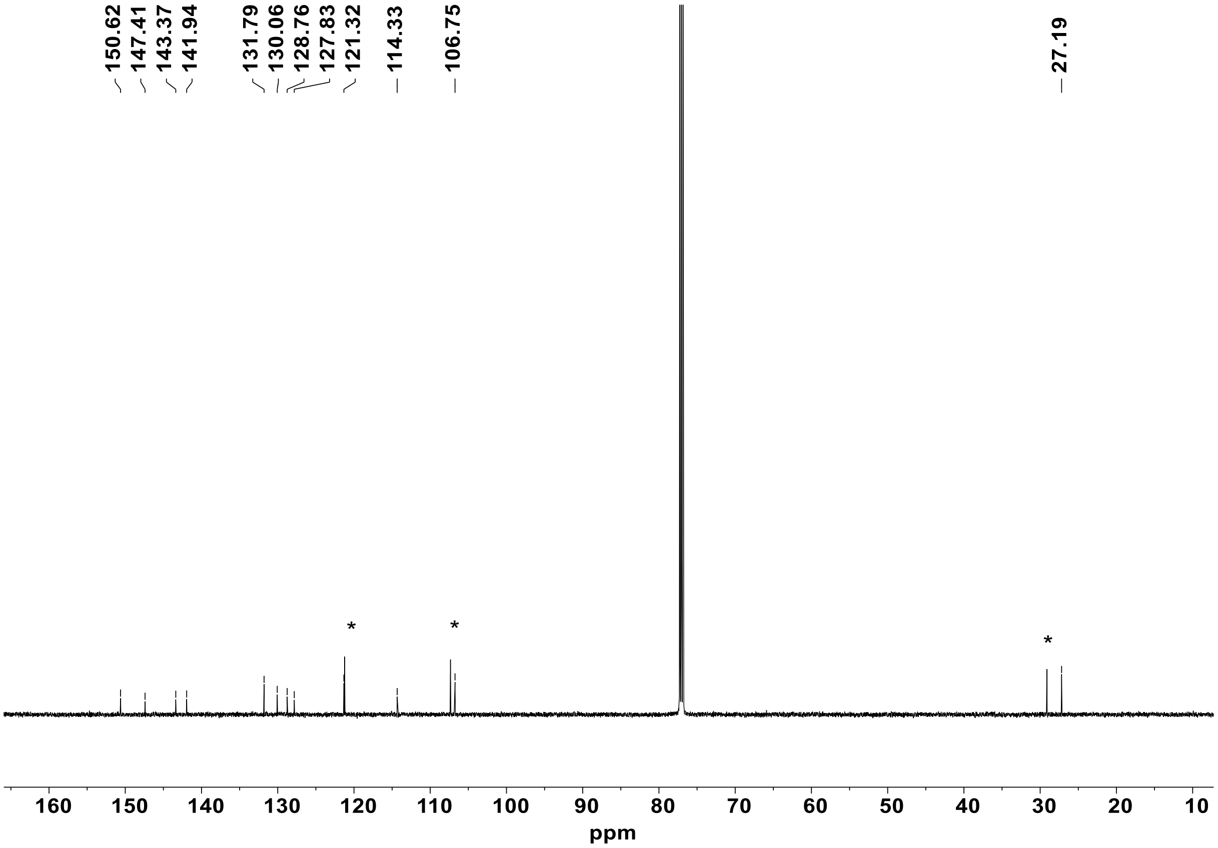

Figure 45: ^{13}C-NMR spectrum, measured at 151 MHz in $CDCl_3$, *residual urea.

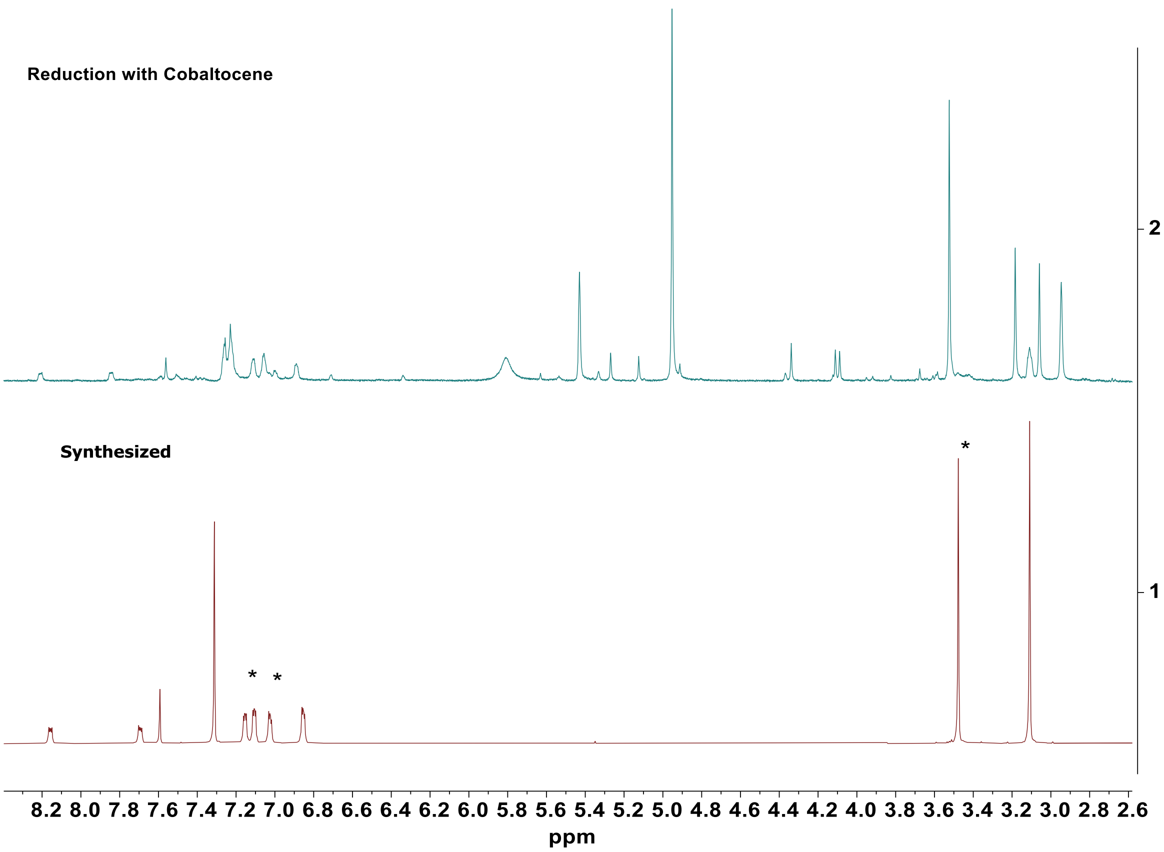

Figure 46: Comparison of the ^{1}H-NMR of reduction of $L5_2(PF_6)_4$ (green) and the through guanidinylation synthesized product (red), *residual urea.

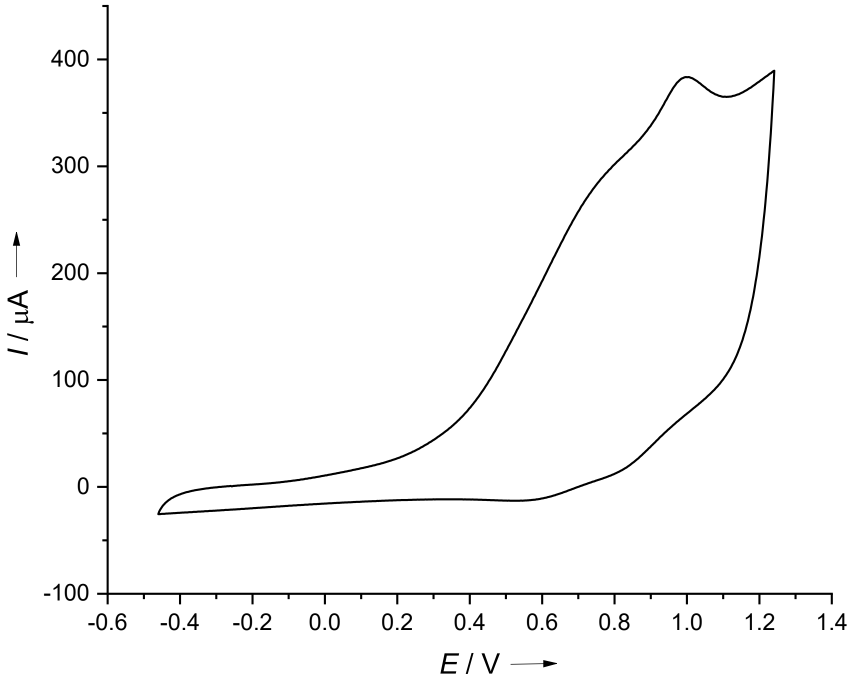

Figure 47: CV curve (Fc/Fc^+ reference, 100mV/s, $n Bu_4NPF_6$, Ag/AgCl electrode, DCM).

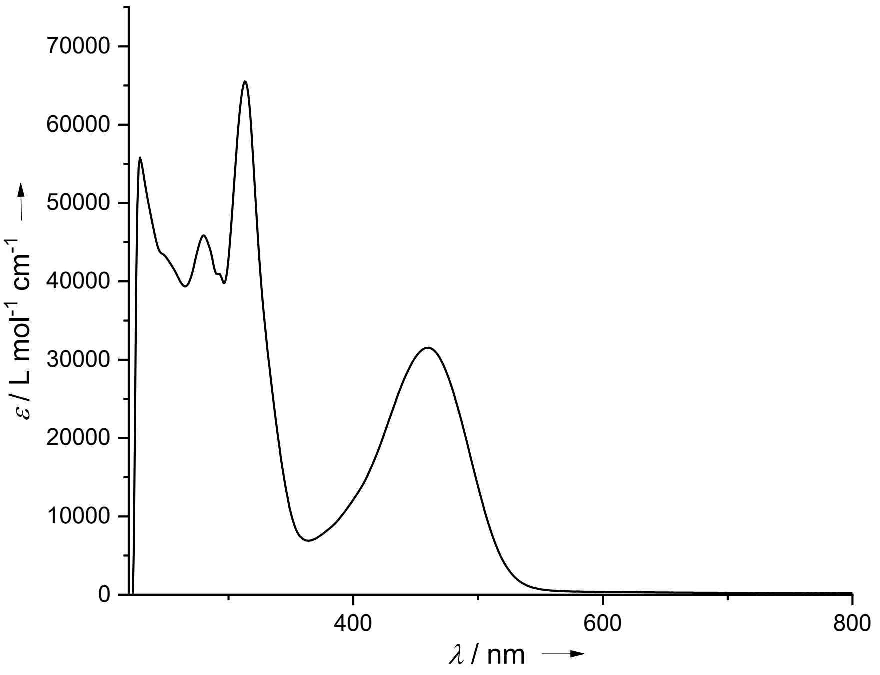

Figure 48: UV/Vis in DCM.

L6$_{Macro}$

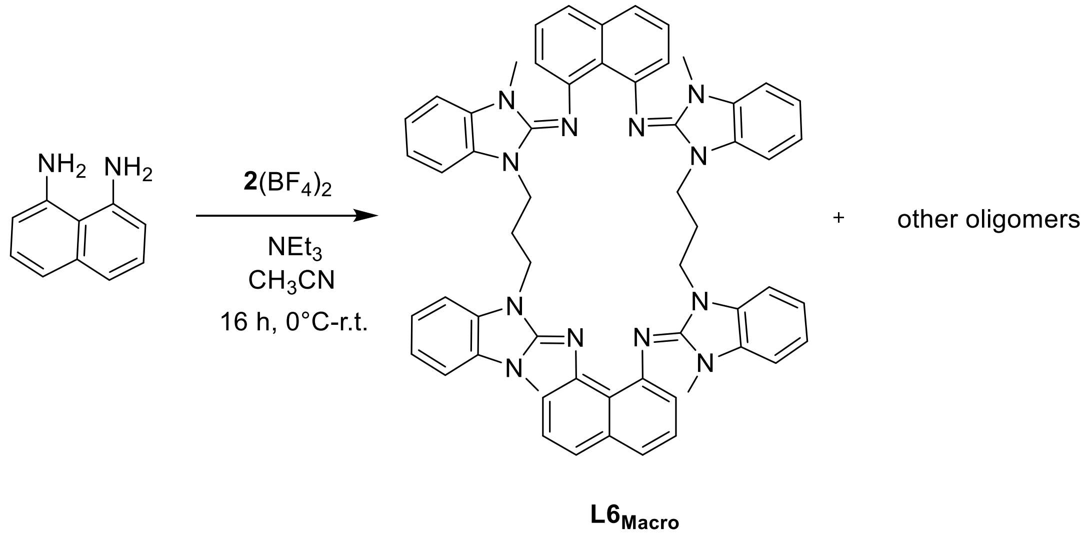

L6$_{Macro}$

2(BF$_4$)$_2$ (582.9 mg, 1.06 mmol, 1.05 eq.) was suspended in MeCN at 0 °C (30 mL). NEt$_3$ (1,40 mL, 1.02 g, 10.11 mmol, 10.0 eq.) and naphthalenediamine were added slowly (160 mg, 1.01 mmol, 1.00 eq.) at 0 °C. The reaction mixture was allowed to warm to r.t. while stirring for 16 h. The solvent was removed *in vacuo* and the residue was dissolved in HCl$_{aq}$ (10.0 mL, 15%). NaOH$_{aq}$ (10.0 mL, 30%) was added while stirring. The aqueous phases were extracted first with Et$_2$O (3·10 mL) and then with acetonitrile (5x10 mL). The solvent of the acetonitrile phase was removed *in vacuo*. The red residue was washed with pentane to give **L6$_{Macro}$** and other oligomers. Due to remaining impurities no yield is given.

^{1}H-NMR (600 MHz, CD$_2$Cl$_2$): No clear assignment of peaks possible due to overlayering of signals and broadened integrals. NMR is shown in Figure 49.

MS (ESI+ in DCM): 917.4519 [L+H$^+$], (calc. 917.4511 [L+H$^+$]).

UV/Vis-Spectrum (MeCN) λ (ε) = 211 (71595), 321 (15376), 349 (15221) nm (M^{-1} cm^{-1}).

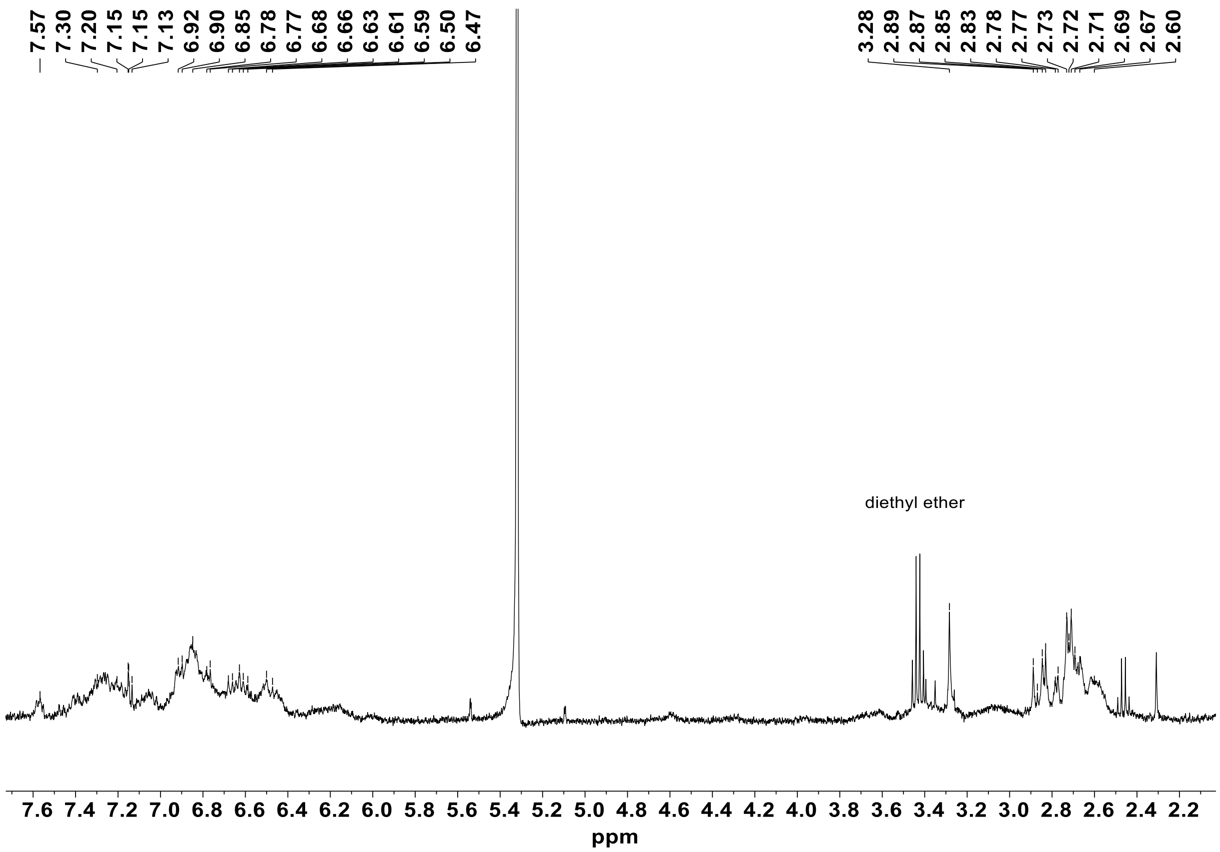

Figure 49: ^{1}H NMR, measured at 600 MHz in CD_2Cl_2,

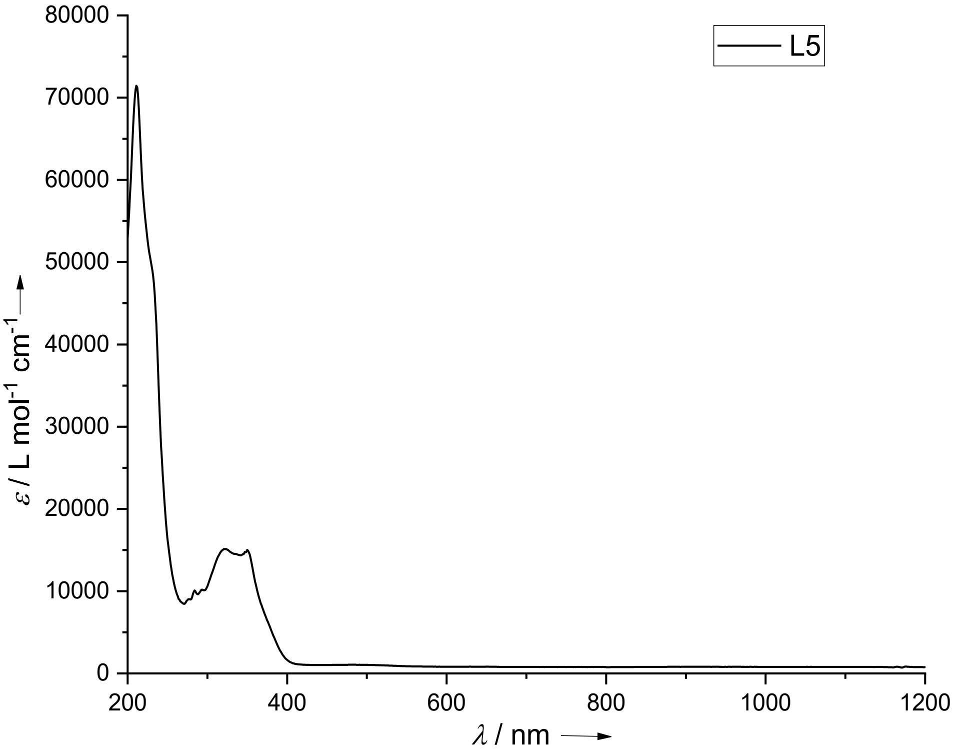

Figure 50: UV/VIS of the crude product of **L6$_{Macro}$** in MeCN.

Quantum-chemical calculations to determine the pK_A Vvalues of the ligands L1, L2 and L5 (B3LYP/def2-TZVP)[15]:

**Ligand**	**pA**	**pK_{BH+} (theory)**	**pK_{BH+} (exp.)**	**ΔpK_{BH+}**
Tetramethylguanidino-benzene		21.1	20.6 (H_2O)	0.5
Btmgb[16]		24.4	-	-
Ttmgn	296.975	27.4	-	-
L1 (ethylene)	282.724	20.3	-	-
L2 (propylene)	282.548	20.2	-	-
L5 (MeMe)	283.812	20.9	-	
Huenigs base			8.5 (DMSO)[17]	
Triethylamine			9.0 (DMSO)[17]	

PA(B) = [E_{el}(B)-E_{el}(BH+)] + [ZPVE(B)- ZPVE(BH+)] = ΔE_{el} + ΔZPVE

pK_{BH+}(theor) = 0.4953 *PA*(CH_3CN)-119.7

ZPVE, engl. zero-point vibrational energy; E_{el}, Influence of the solvent

Calculated structures of L1-L6 and their corresponding dimers after optimization (B3LYP/def2-TZVP).

L1 and L1$_{Macro}$

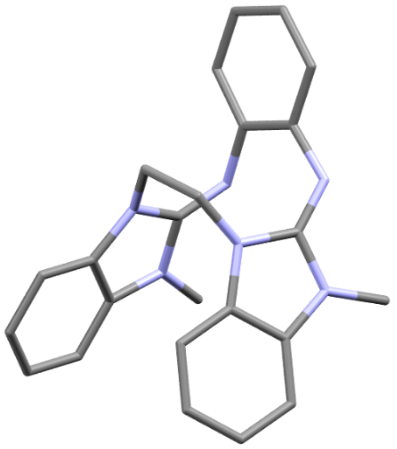

L1 cartesian coordinates [Å]

N	-0.240219	9.913887	10.856815
N	1.318503	10.489427	9.204193
N	2.151879	9.456932	10.986430
N	0.108131	7.152762	10.366620
N	1.963846	6.293959	9.224780
N	2.278399	6.711126	11.390569
C	-0.741718	8.973717	11.749954
C	-0.582190	7.586032	11.506696
C	-1.199759	6.670769	12.360366
H	-1.084842	5.615187	12.145971
C	-1.942563	7.092222	13.456320
H	-2.405838	6.362401	14.107810
C	-2.099108	8.453480	13.696697
H	-2.685900	8.795614	14.539762
C	-1.515156	9.378242	12.842013
H	-1.645255	10.440837	13.00460
C	0.958994	9.922570	10.419658
C	2.675890	10.342040	8.984744
C	3.473917	10.697005	7.912134
H	3.061629	11.197322	7.046454

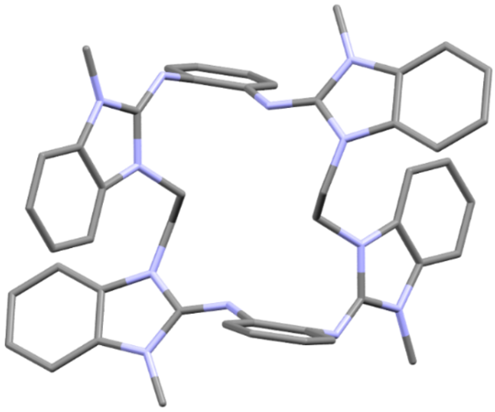

L1$_{Macro}$ cartesian coordinates [Å]

N	2.068227	10.971372	9.643435
N	0.253468	11.095922	8.025690
N	0.409301	12.621839	9.630435
N	3.557266	8.840850	10.819095
N	3.627038	8.360366	13.102334
N	4.358067	10.389497	12.536530
C	3.124233	10.392006	8.955276
C	3.948339	9.455504	9.627889
C	5.130517	9.037509	9.010880
H	5.736978	8.305737	9.530267
C	5.518866	9.513591	7.764487
H	6.443133	9.170331	7.317822
C	4.707241	10.423983	7.099333
H	4.992163	10.811633	6.129480
C	3.529476	10.855003	7.693049
H	2.924375	11.602788	7.195128
C	1.015859	11.485190	9.118283
C	-0.801577	11.986113	7.851417
C	-1.806340	12.040264	6.902513
H	-1.881829	11.299759	6.117870
C	-2.714095	13.098496	6.983796
H	-3.509693	13.169472	6.254349
C	-2.603806	14.066512	7.978517
H	-3.312845	14.882929	8.008718

C	4.835506	10.387564	7.989075
H	5.483816	10.647069	7.162651
C	5.366347	9.752472	9.106157
H	6.421390	9.516797	9.139162
C	4.556542	9.394383	10.188355
H	4.972995	8.872078	11.036512
C	3.210372	9.691760	10.108915
C	0.359199	10.983890	8.246831
H	0.463156	10.453385	7.297477
H	0.496135	12.053586	8.073467
H	-0.631986	10.808231	8.657189
C	2.319163	9.029488	12.358622
H	1.588729	9.552580	12.973246
H	3.311105	9.358292	12.668970
C	1.319674	6.761534	10.366391
C	3.288954	6.010996	9.500144
C	4.319555	5.575093	8.688585
H	4.163881	5.385360	7.635444
C	5.577386	5.403328	9.275194
H	6.404091	5.067465	8.663522
C	5.781774	5.657388	10.626322
H	6.764151	5.514393	11.056204
C	4.735188	6.097887	11.444420
H	4.903180	6.301062	12.493599
C	3.495508	6.274251	10.863423
C	1.342285	6.239582	7.924740
H	0.299736	6.521887	8.047238
H	1.402315	5.231072	7.510143
H	1.828664	6.936993	7.237535
C	2.171597	7.496091	12.601284
H	2.953157	7.163223	13.282219
H	1.218744	7.288141	13.074690
C	-1.590886	14.009781	8.937310
H	-1.504556	14.770522	9.700651
C	-0.697892	12.956335	8.864322
C	0.426129	9.851174	7.310186
H	0.965632	9.997968	6.372382
H	-0.552451	9.418316	7.102063
H	0.996018	9.164387	7.930820
C	1.105790	13.452929	10.602340
C	3.830354	9.200499	12.008180
C	3.991755	8.995810	14.273963
C	3.960811	8.575006	15.591105
H	3.610610	7.587021	15.857586
C	4.389648	9.478323	16.569513
H	4.380564	9.176166	17.608417
C	4.823207	10.753614	16.227655
H	5.149404	11.435705	17.001395
C	4.855296	11.173273	14.893416
H	5.203211	12.161485	14.639345
C	4.444521	10.278416	13.925416
C	3.141480	7.007943	12.993564
H	3.022927	6.785749	11.936104
H	2.176525	6.899029	13.495091
H	3.852261	6.307559	13.438784
C	4.278337	11.664344	11.844100
N	4.585248	14.723404	12.288843
N	6.350310	14.619779	13.960335
N	6.255644	13.083748	12.360480
N	3.126372	16.850972	11.067035
N	3.021854	17.340420	8.783761
N	2.290998	15.313910	9.361301
C	3.508638	15.302420	12.945423
C	2.703517	16.237463	12.249073
C	1.500386	16.649970	12.828312
H	0.907214	17.379130	12.290138
C	1.074472	16.171325	14.061234
H	0.135256	16.510637	14.478814
C	1.868560	15.263870	14.751130

H	1.554730	14.874470	15.711290
C	3.065720	14.837200	14.194250
H	3.656630	14.090210	14.710000
C	5.624270	14.217580	12.847860
C	7.412190	13.744590	14.166480
C	8.393260	13.708360	15.140680
H	8.436980	14.452160	15.924530
C	9.319330	12.664560	15.085510
H	10.09738	12.608340	15.834890
C	9.249480	11.692290	14.091250
H	9.972310	10.887570	14.080720
C	8.259740	11.730210	13.107770
H	8.205300	10.966770	12.344230
C	7.348640	12.769360	13.155170
C	6.143310	15.869460	14.658630
H	5.536670	15.734480	15.556100
H	7.110630	16.289650	14.932990
H	5.628360	16.561900	13.996900
C	5.609140	12.247830	11.358270
C	2.831740	16.501040	9.879440
C	2.631020	16.707610	7.618230
C	2.638550	17.129630	6.301210
H	2.991080	18.115090	6.028500
C	2.183480	16.230470	5.330890
H	2.173860	16.533580	4.292270
C	1.747540	14.957900	5.680260
H	1.400610	14.279340	4.912470
C	1.739520	14.536710	7.014300
H	1.387190	13.551400	7.274740
C	2.176930	15.427470	7.974490
C	3.504720	18.694330	8.887430
H	3.606090	18.926880	9.944570
H	4.477930	18.801250	8.401570
H	2.799730	19.388100	8.423470
C	2.408540	14.038490	10.048610
H	6.294310	11.453710	11.071340
H	5.423650	12.860050	10.476710

H	3.80090	12.37880	12.51348
H	3.60237	11.53680	11.00724
H	1.33747	12.83379	11.46760
H	0.43783	14.24531	10.93138
H	3.12660	14.16678	10.85110
H	2.85180	13.32634	9.35364

L2 and L2$_{Macro}$

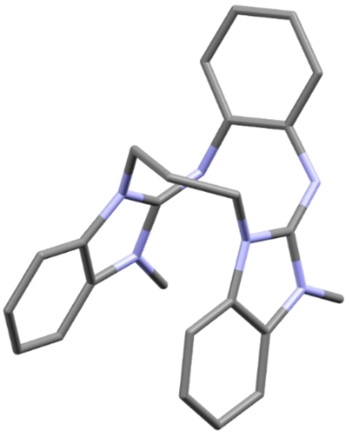

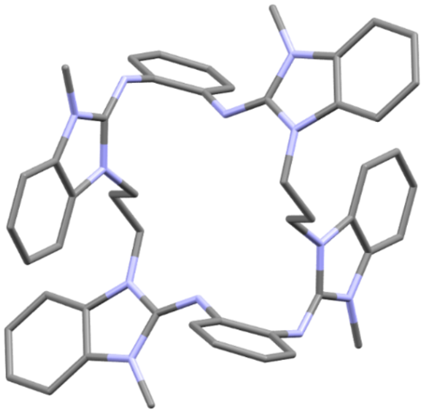

L2 cartesian coordinates [Å]

N	4.017312	6.189567	14.697828
N	2.091112	5.960646	16.018490
N	1.952056	5.125369	13.960306
N	4.398618	3.451057	15.024462
N	2.648689	2.136348	15.828511
N	2.809795	2.234707	13.613095
C	4.964497	5.533244	13.923192
C	5.174664	4.137347	14.088744
C	6.229187	3.525107	13.410549
H	6.397228	2.467480	13.574931
C	7.049341	4.244458	12.548353
H	7.856547	3.744481	12.028686
C	6.835597	5.607312	12.375198
H	7.473437	6.180778	11.714609
C	5.810927	6.241603	13.066099
H	5.648305	7.306831	12.959670
C	2.816013	5.777264	14.849956
C	0.837981	5.388245	15.900028

L2$_{Macro}$ cartesian coordinates [Å]

N	1.955448	11.068002	9.722417
N	0.186163	10.844632	8.065984
N	-0.091922	12.209584	9.797737
N	3.230749	8.708031	10.647911
N	2.909551	8.261800	12.916374
N	4.005785	10.143232	12.474515
C	3.01608	10.513931	9.005804
C	3.701267	9.396125	9.531845
C	4.804423	8.896105	8.833708
H	5.293244	8.014421	9.228712
C	5.268788	9.500599	7.672238
H	6.133573	9.096515	7.161610
C	4.618840	10.625085	7.177105
H	4.973037	11.117413	6.280488
C	3.504403	11.119955	7.841624
H	3.000589	12.002318	7.466433
C	0.809705	11.326857	9.215421
C	-1.060863	11.444321	7.923626

C	-0.188974	5.249807	16.815877	C	-2.040155	11.305328	6.958003
H	-0.102878	5.643149	17.819719	H	-1.907938	10.646673	6.110553
C	-1.345074	4.585059	16.395743	C	-3.210071	12.056612	7.108509
H	-2.163052	4.454113	17.091626	H	-3.992490	11.969990	6.366517
C	-1.457204	4.091109	15.101524	C	-3.379260	12.914546	8.189988
H	-2.358007	3.574058	14.800450	H	-4.291582	13.489075	8.278995
C	-0.415404	4.229853	14.179819	C	-2.388127	13.054371	9.166044
H	-0.509649	3.815757	13.188293	H	-2.522537	13.725505	10.003024
C	0.736049	4.872622	14.596075	C	-1.233851	12.306883	9.018993
C	2.673072	6.460227	17.240274	C	0.639185	9.714458	7.279490
H	2.630017	5.699282	18.023411	H	1.227327	10.029884	6.416223
H	2.146554	7.353286	17.582747	H	-0.232806	9.156066	6.939775
H	3.710296	6.707367	17.029498	H	1.254303	9.063971	7.894820
C	3.408243	2.686378	14.794400	C	0.196885	13.019944	10.969039
C	1.584454	1.418906	15.318674	H	0.928335	12.476258	11.565922
C	0.542013	0.758296	15.940418	H	-0.720271	13.094017	11.554745
H	0.455395	0.733394	17.017898	C	3.377213	9.054742	11.866600
C	-0.409328	0.137698	15.124751	C	3.266222	8.810634	14.132853
H	-1.239533	-0.382433	15.583689	C	3.046640	8.391509	15.432366
C	-0.306560	0.179867	13.739135	H	2.503960	7.480501	15.645517
H	-1.053876	-0.312370	13.131132	C	3.551423	9.190217	16.464781
C	0.748819	0.853293	13.112988	H	3.396203	8.886676	17.491625
H	0.818533	0.889987	12.034434	C	4.251441	10.359995	16.192021
C	1.683844	1.472691	13.919745	H	4.639336	10.955192	17.008012
C	2.857482	2.446569	17.220621	C	4.471522	10.781729	14.875635
H	3.745763	3.069310	17.289006	H	5.031296	11.683946	14.670587
H	3.007938	1.534892	17.802803	C	3.966045	9.996483	13.856801
H	1.996360	2.988271	17.621820	C	2.206362	7.017973	12.729496
C	2.143666	5.144204	12.515339	H	2.099593	6.857694	11.659910
H	3.113035	5.601295	12.334293	H	1.217356	7.061102	13.192201
H	1.383181	5.801803	12.081184	H	2.765341	6.186815	13.167257
C	2.069644	3.789288	11.810573	C	4.470770	11.364001	11.837546
H	1.060404	3.383237	11.876786	H	4.378703	12.148715	12.584325
H	2.252073	3.979664	10.749028	H	3.782746	11.625528	11.040474
C	3.071542	2.730464	12.272151	C	5.906614	11.303508	11.323832
H	3.028384	1.876682	11.591506	H	6.554892	10.856547	12.082045
H	4.084040	3.117460	12.236692	H	5.954343	10.670443	10.437205

N	4.667392	14.648908	12.214183
N	6.436592	14.872184	13.870743
N	6.714795	13.507363	12.138903
N	3.392078	17.009038	11.288955
N	3.713105	17.455746	9.020591
N	2.616811	15.574249	9.462068
C	3.606722	15.202885	12.930827
C	2.921578	16.320778	12.404929
C	1.818496	16.820792	13.103191
H	1.329734	17.702572	12.708329
C	1.354109	16.216168	14.264584
H	0.489378	16.620257	14.775300
C	2.003971	15.091554	14.759532
H	1.649753	14.599116	15.656081
C	3.118371	14.596716	14.094927
H	3.622183	13.714305	14.470013
C	5.813123	14.390055	12.721220
C	7.683556	14.272387	14.013198
C	8.662729	14.411212	14.978964
H	8.530457	15.069805	15.826455
C	9.832600	13.659842	14.828550
H	10.614926	13.746329	15.570655
C	10.001863	12.801989	13.747018
H	10.914149	12.227392	13.658082
C	9.010847	12.662327	12.770820
H	9.145317	11.991260	11.933795
C	7.856613	13.409898	12.917785
C	5.983581	16.002323	14.657297
H	5.395322	15.686861	15.520469
H	6.855585	16.560605	14.997160
H	5.368584	16.652928	14.041971
C	6.426038	12.697113	10.967513
H	5.694717	13.240933	10.370597
H	7.343244	12.622959	10.381894
C	3.245483	16.662572	10.070202
C	3.356231	16.907299	7.804006
C	3.575661	17.326812	6.504592

H	4.118398	18.237833	6.291646
C	3.070635	16.528488	5.472005
H	3.225729	16.832338	4.445233
C	2.370508	15.358717	5.744503
H	1.982398	14.763845	4.928378
C	2.150581	14.936590	7.060789
H	1.590679	14.034413	7.265638
C	2.656348	15.721423	8.079803
C	4.416437	18.699456	9.207728
H	4.523357	18.859427	10.277345
H	5.405381	18.656361	8.744886
H	3.857483	19.530787	8.770267
C	2.151903	14.353289	10.098754
H	2.243930	13.568744	9.351775
H	2.840001	14.091576	10.895716
C	0.716104	14.413601	10.612622
H	0.067676	14.860467	9.8544822
H	0.668385	15.046658	11.499251

L3 and L3$_{Macro}$

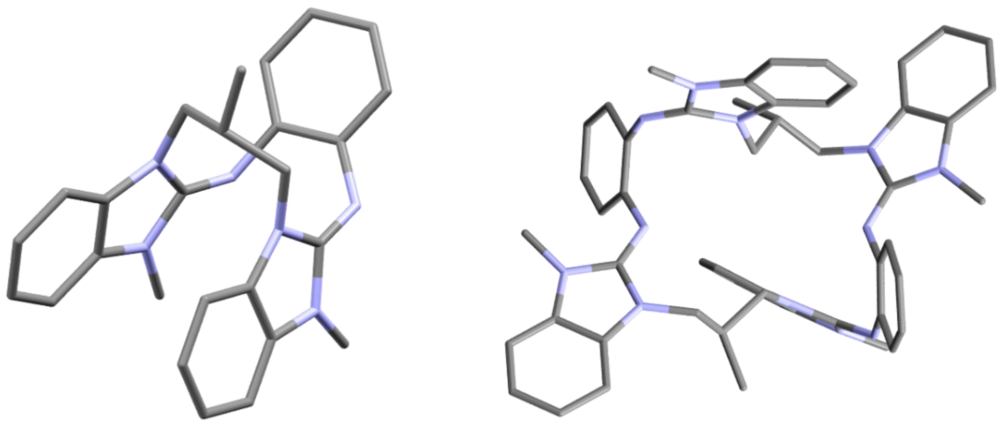

L4 and L5

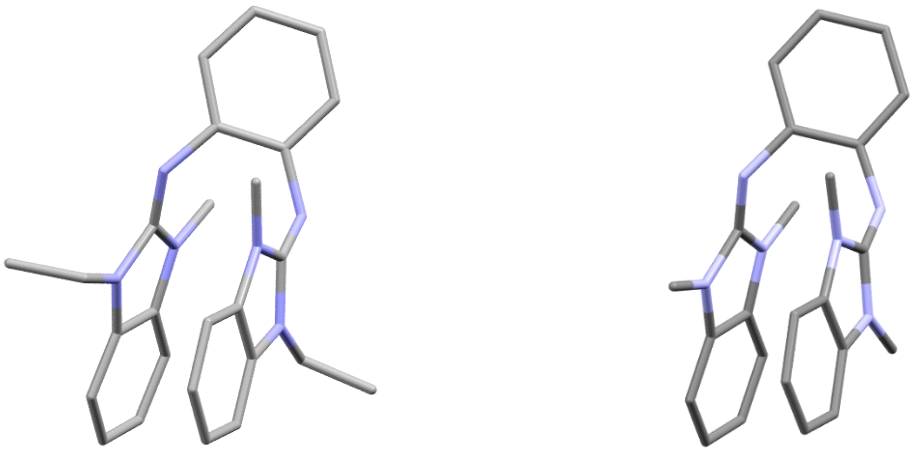

L6 and L6$_{Macro}$

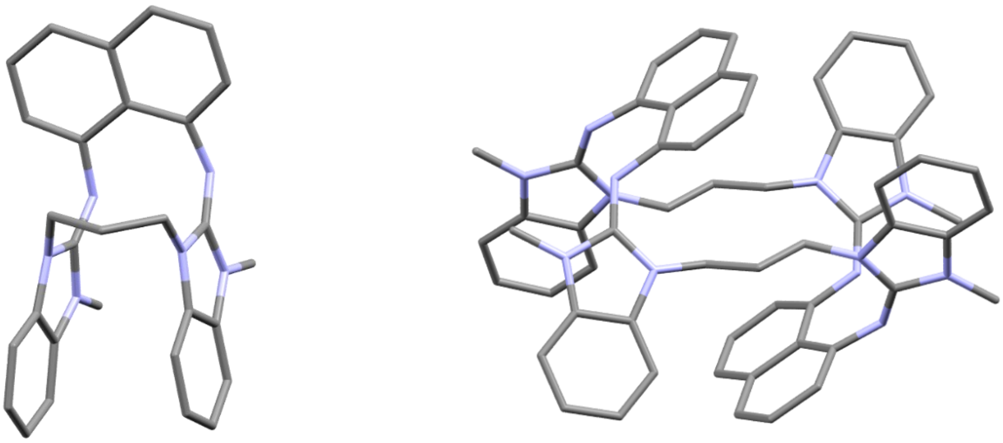

Quantum-chemical calculations for the dimerization of L1 and L2 (B3LYP/def2-TZVP)

Table 1: Thermodynamic data *ΔH, ΔS* and *ΔG* for the theoretical formation of L1 and L2 out of their respective macrocycles.

**Ligand**	**ΔH [kJ/mol]**	**ΔS [kJ K^{-1} mol^{-1}]**	**ΔG [kJ/mol]**
**L1$_{Macro}$→ 2 L1**	17.09	-0.199	-42.16
**L2$_{Macro}$ → 2 L2**	41.80	-0.174	-10.21

HOMO Plots (B3LYP/def2-TZVP, Iso-value 0.05) of L1-L5 (from left to right) and their respective relative energies [eV]. L1 (103) E = -4.9939 eV, L2 (107) E = - 4.2249 eV, L3 (111) E = -4.1219 eV, L4 (112) E = -4.6623 eV, L5 (104) E = -4.4523 eV.

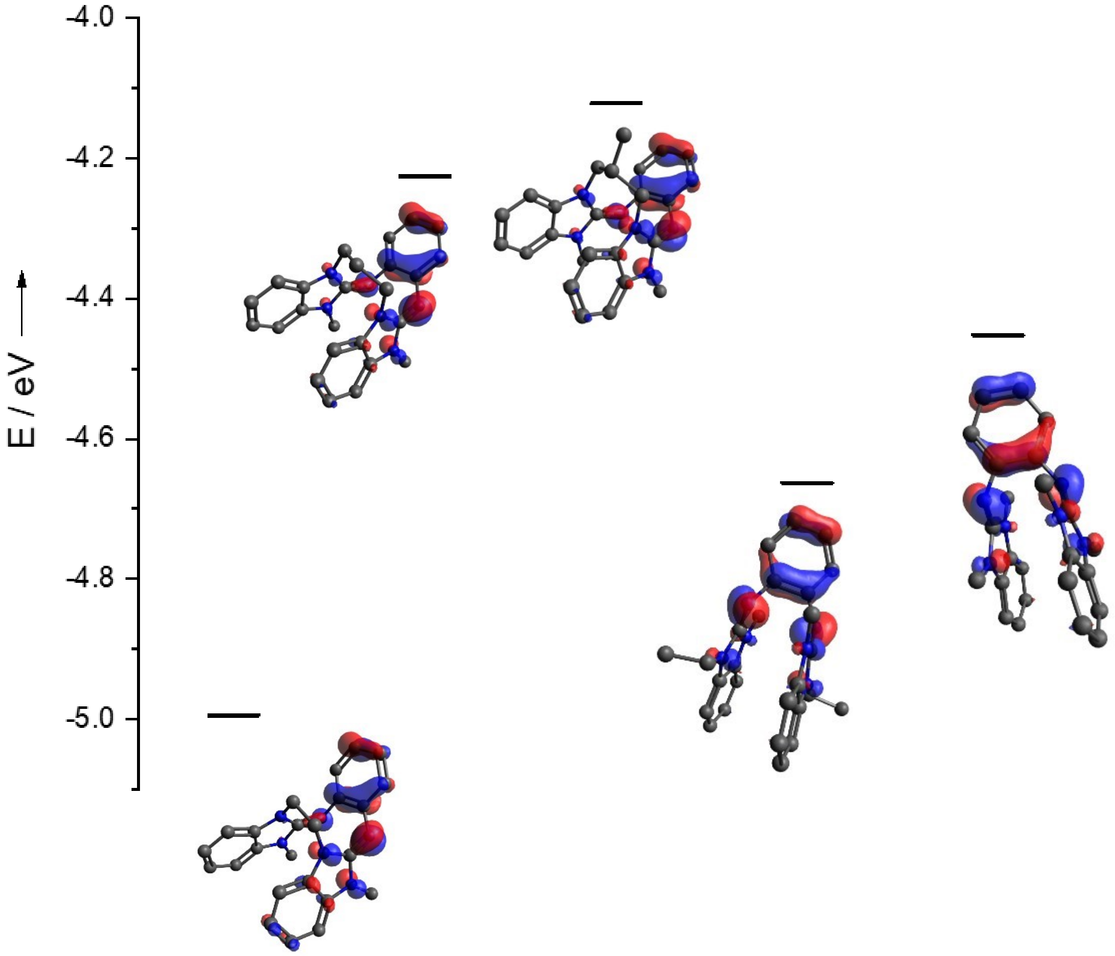

Quantum-chemical calculations for the oxidation of L1 and L4 (B3LYP/def2-TZVP) and their cartesian coordinates [Å].

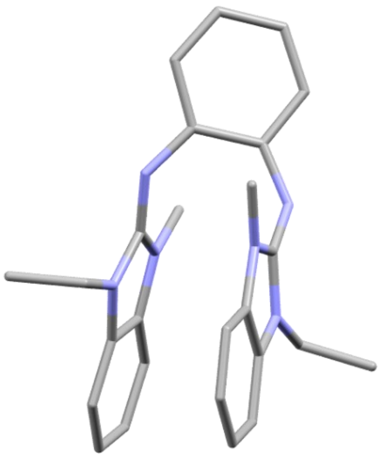

$L4^{+\cdot}$

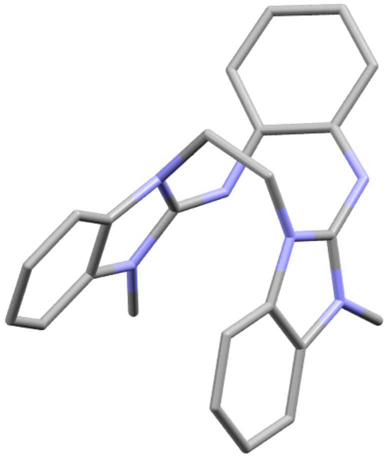

$L1^{+\cdot}$

$L1^{+\cdot}$

N	0.05651081146253	10.13295481811958	11.46393300246622
N	1.27667111469621	10.44385911451749	9.44840583237972
N	2.38310748336189	9.59698936827138	11.15441534126532
N	0.32339841262472	7.51931776716463	10.37468758551437
N	2.11133063917444	6.47274477300806	9.26396529674008
N	2.36693372063571	6.80677648244753	11.44251377884916
C	-0.70196739434713	9.12540352630434	11.88278043371479
C	-0.51395461160447	7.75438396174519	11.41889393501674
C	-1.27392725488208	6.73977000359824	12.01899944840302
H	-1.12454794254232	5.72127761280546	11.68580738210824
C	-2.21856171978971	7.03587399944572	12.97909666321871
H	-2.80351493008404	6.23810226673183	13.41737189434897
C	-2.45225438892302	8.37231253928020	13.38252562921661
H	-3.21205804579835	8.58076172322355	14.12336041515042
C	-1.72162875582469	9.39112504754624	12.84416354819111
H	-1.86983913913090	10.41967061933602	13.14333328244314
C	1.15707806690778	10.01020244710537	10.72243307036451
C	2.58110910789273	10.22359503154590	9.00779183920707
C	3.17851310659987	10.42023343320416	7.77291573376525
H	2.63133571966120	10.82698376070196	6.93406926151582

C	4.52065560865464	10.07075871202573	7.65957283547030
H	5.02377549047423	10.20927527320326	6.71252425661631
C	5.23016789169499	9.54594989336134	8.74265580602902
H	6.27018963113658	9.27891933323790	8.61718497657427
C	4.62780222771599	9.34858435387886	9.98066130736169
H	5.18243534670850	8.92076467478832	10.80243900395766
C	3.29059647647680	9.69312831700347	10.09222928867169
C	0.16880356820954	10.90588743070908	8.63092575672784
H	-0.16462805996736	10.11208914213274	7.96139978841226
H	0.48721409460605	11.76864888849896	8.04891460099616
H	-0.64796252698253	11.19393712549072	9.28705242573493
C	2.68470359224432	9.05761847904975	12.47306017370486
H	2.20139399790398	9.67937069096941	13.22663419863199
H	3.75993764249460	9.15523433003781	12.60298239307952
C	1.48151454017996	6.94626345097021	10.38248584404659
C	3.41464746791186	6.09613626657202	9.57979337573093
C	4.44554987305849	5.62102704013659	8.79010182639666
H	4.31969632973012	5.46017265452025	7.72859958485200
C	5.66427645617576	5.36799541875377	9.41864108124552
H	6.49312064869420	4.99577574056227	8.83239569906524
C	5.83390359783384	5.58209395522926	10.78574973719133
H	6.79068518990654	5.37177654492754	11.24346982666422
C	4.78903678296349	6.05718763142180	11.57915137094901
H	4.92716775663255	6.21711943031130	12.63990264668219
C	3.58306128036426	6.30906586130034	10.95228265135462
C	1.53432751734940	6.48056156539524	7.93586016579157
H	0.48075374473984	6.73053608449671	8.02413104374223
H	1.63583780189567	5.49712933025828	7.47749209315162
H	2.03401222125066	7.22397748347395	7.31104603175743
C	2.24792171301669	7.58616197280845	12.66033135969116
H	2.86436194404443	7.12451012831340	13.42985417288660
H	1.22224715279148	7.53285650005804	13.00933230295280

$L4^{+\cdot}$

N	5.89799091478505	5.63485199632444	8.02857681204400
C	5.18428977355201	6.74496997833573	8.29254037813868
N	5.91969239148882	4.38729509035589	10.10745022295596
C	5.73224532374353	7.99281358670583	7.89851211089970
H	6.67803629019371	7.97378660293841	7.37434534519513
N	6.14634002426393	3.30005109160826	8.19672983867424
C	5.12898930169634	9.17241058944382	8.23702207018915
H	5.58813983576283	10.11253771558283	7.96237824893031
C	5.94752369122684	4.53746182097840	8.74261788756466
C	6.07388142093545	3.03901423972492	10.42156590143121
C	6.07841818808430	2.37446158822680	11.63725747511440
H	5.96953595834596	2.90076307697550	12.57518260662341
C	6.21485197751408	0.98931445534693	11.60122704550921
H	6.22337850144844	0.43559888480551	12.53002504312345
C	6.33798839279312	0.30083445020343	10.39121515723398
H	6.43989730461624	-0.77564284873843	10.40210085545064
C	6.33816445127844	0.97096695457790	9.17129645434744
H	6.43242913664098	0.43502680939886	8.23774524630773
C	6.21013390791500	2.35024115478129	9.20889235971700
C	5.86265896729412	5.45947453700777	11.08463499153754
H	6.23105289116924	6.37791008815114	10.63626403431332
H	6.50282384632383	5.20044886310852	11.92568636472488
H	4.84287494083944	5.62060138708841	11.43422604702162
C	6.36630136421239	3.04142845193715	6.77668351118882
H	5.93817982118679	3.87932745227058	6.23291315252824
H	5.79947792538156	2.14868933931804	6.51229635907651
C	7.84490205882997	2.88597183177229	6.44732495737171
H	7.96519654290147	2.67090426159902	5.38495002188488
H	8.29423169481259	2.06868178559527	7.01201852046191
H	8.38767000934699	3.80378683683232	6.67481077878396
N	3.14915804772141	5.63477355104876	9.18153031775181
C	3.86295179300832	6.74487810996112	8.91783360012808
N	3.12808108971959	4.38702970314430	7.10275550341781

C	3.31496373633034	7.99266522107173	9.31210238687332
H	2.36917716916043	7.97350124686944	9.83627152276076
N	2.90085518694217	3.29992627554594	9.01348378173951
C	3.91819684080573	9.17233151089914	8.97382904457435
H	3.45904277432218	10.11240153757778	9.24866163979607
C	3.09986664000654	4.53728448812295	8.46756215822549
C	2.97360881079009	3.03877276847077	6.78866213802221
C	2.96922266743799	2.37416000169797	5.57300001808818
H	3.07840794986319	2.90039795487223	4.63507607156256
C	2.83246957153377	0.98905008725195	5.60906427252180
H	2.82398298517505	0.43528663509526	4.68029485659872
C	2.70898927918893	0.30065554089877	6.81909401928794
H	2.60683755098700	-0.77579975548660	6.80824384642120
C	2.70874840240437	0.97083898114557	8.03898216076535
H	2.61428625288598	0.43488277295653	8.97250174597482
C	2.83702992227657	2.35008880121650	8.00134781751481
C	3.18575501370892	5.45918329563854	6.12558221803746
H	2.81671837104547	6.37754774430100	6.57359375024300
H	2.54640272229923	5.19994152570876	5.28398933580231
H	4.20580893119664	5.62051723109846	5.77688731473524
C	2.68062276885723	3.04133021150524	10.43350424317884
H	3.10903257553811	3.87899862031644	10.97739380688866
H	3.24702739857893	2.14832945864903	10.69788105276739
C	1.20191743930520	2.88648367704798	10.76264826566486
H	1.08139357891762	2.67140325747852	11.82499543331269
H	0.75231438284080	2.06941432374425	10.19784945011091
H	0.65957329856854	3.80454314989469	10.53513842888981

Table 2: Thermodynamic data E_I and *ΔG* for the theoretical formation of $L1^{+\cdot}$ and $L4^{+\cdot}$ out of their respective neutral counterparts.

Ligand	E_i [kJ/mol]	ΔG [kJ/mol]
**L1→ $L1^{+\cdot}$**	570	568
**L4→ $L4^{+\cdot}$**	563	561

4. Synthesis protocols, analytic data and quantum chemical calculations of the complexes

[CoBr₂(L2)]

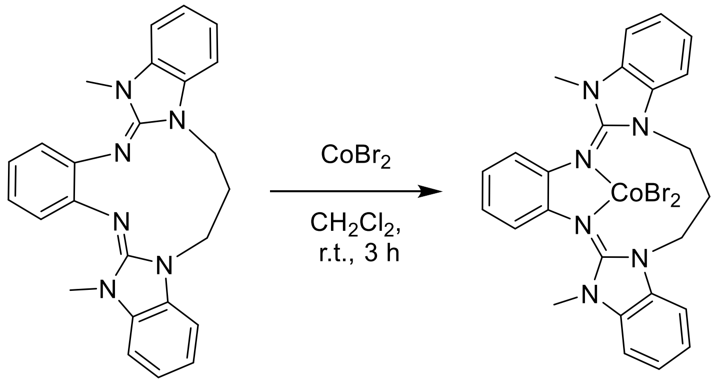

L2 (15 mg, 36.7 µmol, 1.0 eq.) and $CoBr_2$ (12 mg, 55.1 µmol, 1.5 eq.) were suspended in DCM (3 mL). After 3 h the suspension was filtered, and the solvent removed *in vacuo*. After washing with ether (3x3 mL) the product was yielded as a green solid (19 mg, 30.3 µmol, 82%). Crystals for structural analysis were obtained through slow evaporation of the solvent out of a saturated DCM solution.

MS (ESI^+ in DCM): *m/z* = 546.0577 $[CoBrL2]^+$ (calc: 546.0578).

EA (C, H, N in %) (x0.5 DCM) $C_{25}H_{24}N_6CoBr_2$: calc.: C: 45.73, H 3.76, N 12.55
found: C 45.73, H 4.41, N 12.11.

CV (DCM, $[nBu_4N][PF_6]$ 100 mV/s, reference Fc/Fc^+): $E_{1/2}$ = 0.249 V, E_{ox} = 0.745 V.

UV/Vis-spectrum (DCM): λ (ε) = 227 (25417), 331 (14522), 572 (284, dd), 637 (326, dd), 685 (294, dd) nm (M^{-1} cm^{-1}).

Table 3: Selected bondlengths of [$CoBr_2$(L2)].

Bond	Distance [Å]
Co-(N11,C14,C13,N12) plane	0.640
Co-Br	2.3699(8)/
	2.3746(7)
C10-N12	1.333(6)
C10-N9/C10-N8	1.352(6)/1.364(6)
Co-N12/Co-N11	2.020(4)/2.023(4)

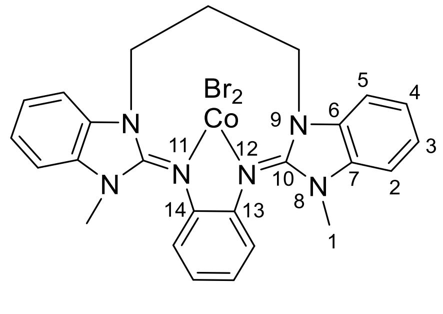

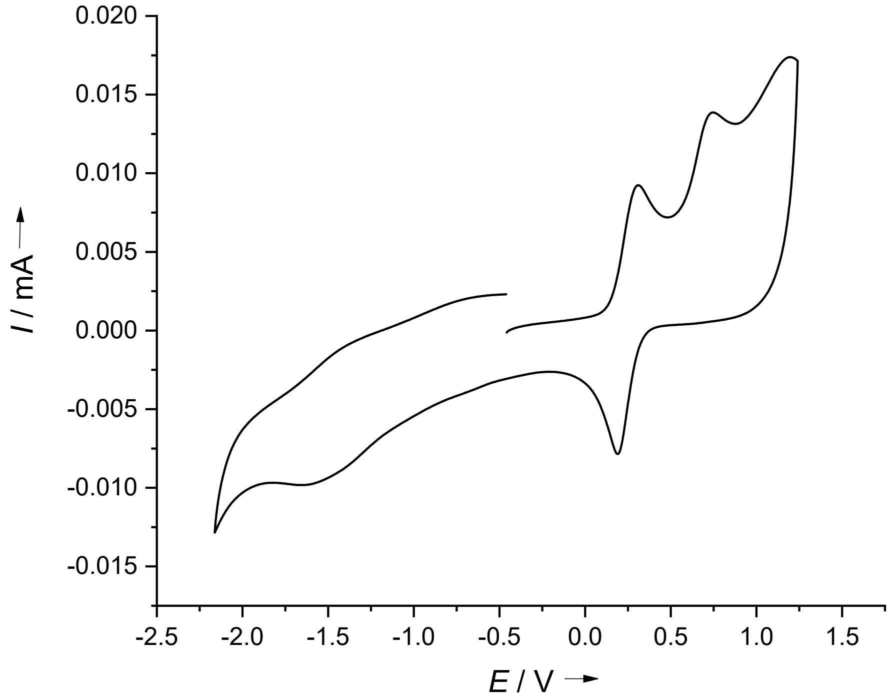

Figure 51: CV curve of [$CoBr_2$(L2)] (Fc/Fc^+ reference, 100 mV/s, nBu_4NPF_6, Ag/AgCl electrode, DCM).

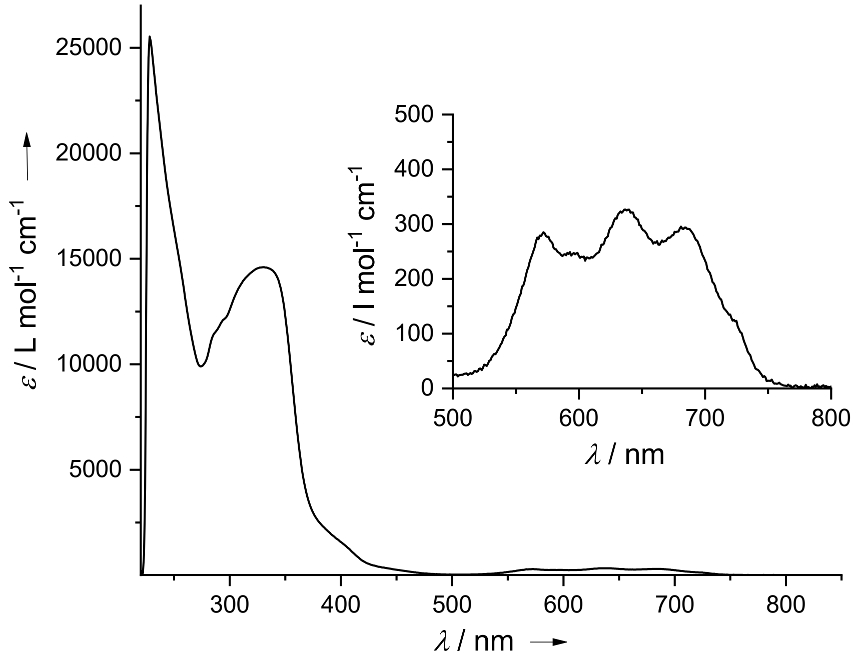

Figure 52: UV/Vis spectrum of [$CoBr_2$(L2)] in DCM.

$[Co_2(hfac)_4(L2_{Macro})]$

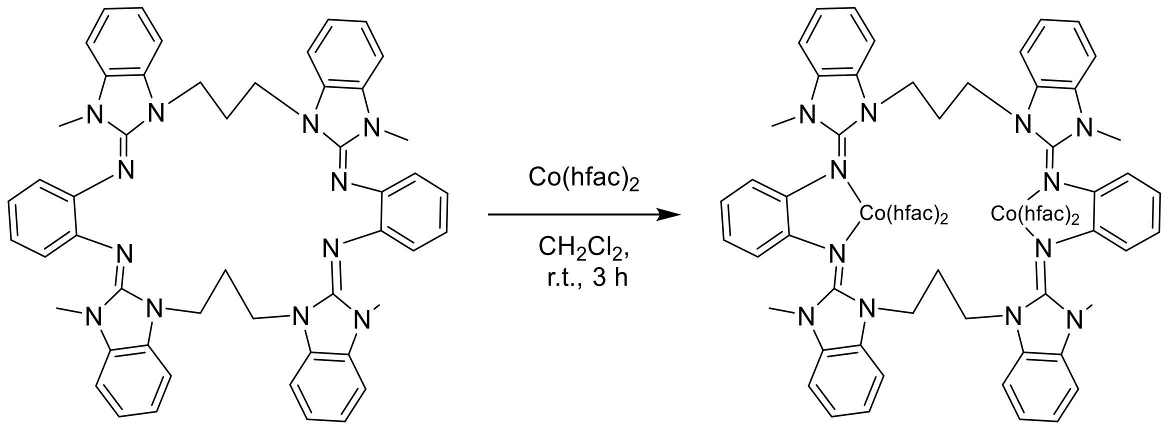

L2 (15 mg, 36.7 µmol, 1.0 eq.) and $Co(hfac)_2$ (19.11 mg, 55.1 µmol, 1.1 eq.) were suspended in DCM (3 mL). After 3 h the solvent was removed *in vacuo*. The residue was dissolved in diethylether and filtered. Then the solvent was removed *in vacuo* and the product was yielded as a red solid (19.45 mg, 22.06 µmol, 60%). Crystals for structural analysis were grown at -12 °C in a saturated DCM solution.

MS (ESI^+ in DCM): *m/z* = 1082.3343 $[Co(hfac)L2_{Macro}]^+$ (calc: 1082.3338).

EA (C, H, N in %) (x0,5 DCM) $C_{70}H_{52}N_{12}Co_2F_{24}$: calc: C: 44.74, H 2.92, N 8.70
found: C 43.91, H 3.07, N 8.35.

CV (DCM, $[nBu_4N][PF_6]$ 100 mV/s, vs. Fc/Fc^+): $E_{1/2}$ = 0.125 V E_{ox} = 0.675, 0.965 V.

UV/Vis spectrum (DCM): λ (ε) = 311 (16982), 228 (17739) nm (M^{-1} cm^{-1}).

Table 4: Selected bondlengths of $[Co_2(hfac)_4(L2_{Macro})]$.

Bond	Distance [Å]
Co-(N11,C14,C13,N12) plane	0.551
Co-O(hfac)	2.1058(19)/
	2.0751(18)/
	2.1166(18)/
	2.0764(18)
C10-N12	1.312(3)
C10-N9/C10-N8	1.369(3)/1.376(3)
Co-N12/Co-N11	2.084(2)/2.127(2)

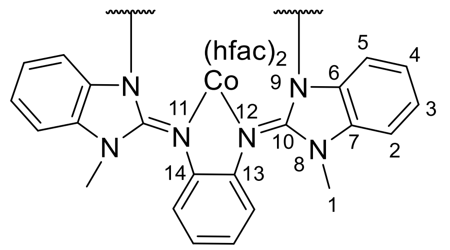

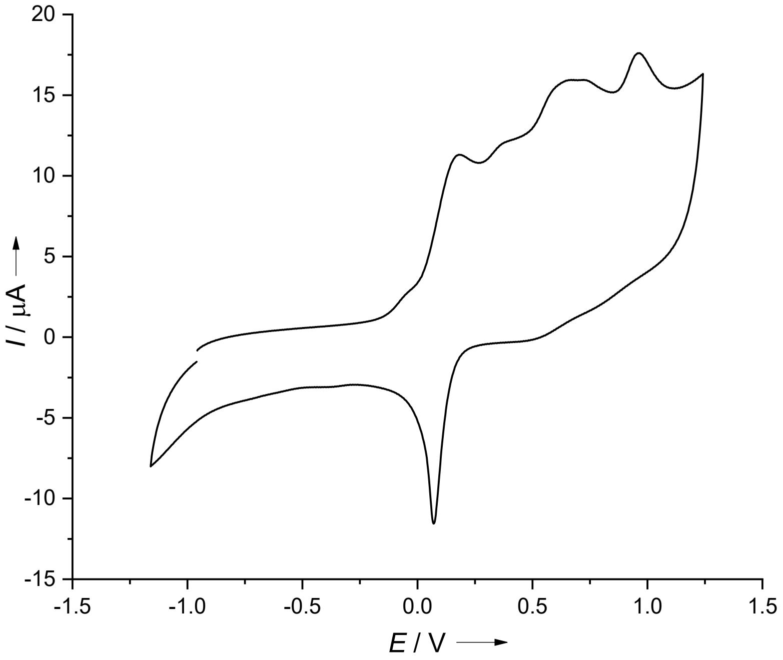

Figure 53: CV curve of [Co_2(hfac)$_4$(L2$_{Macro}$)] (Fc/Fc^+ reference, 100 mV/s, *n*Bu_4NPF_6, Ag/AgCl electrode, DCM).

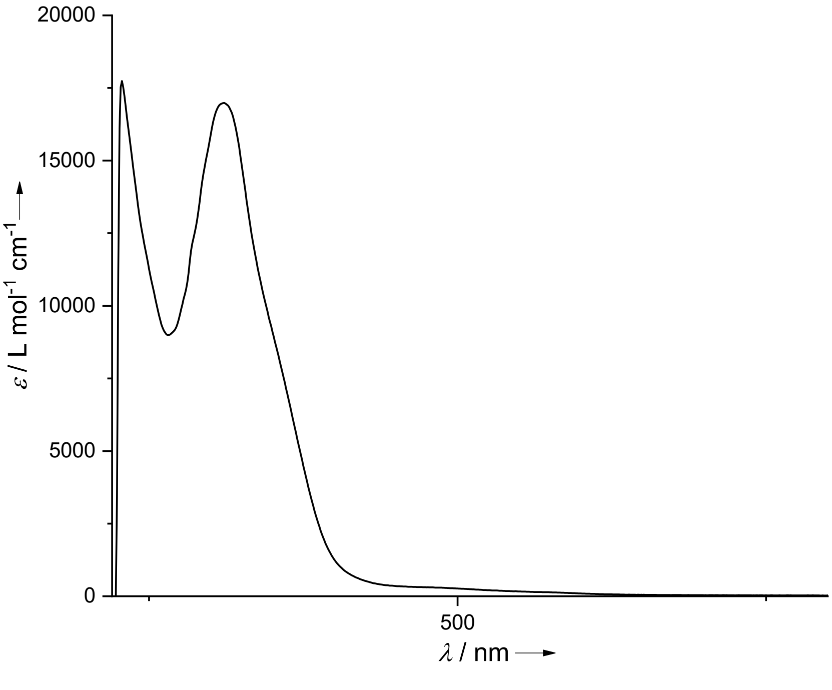

Figure 54: UV/Vis spectrum of [Co_2(hfac)$_4$(L2$_{Macro}$)] in DCM.

[NiCl$_2$(L2)]

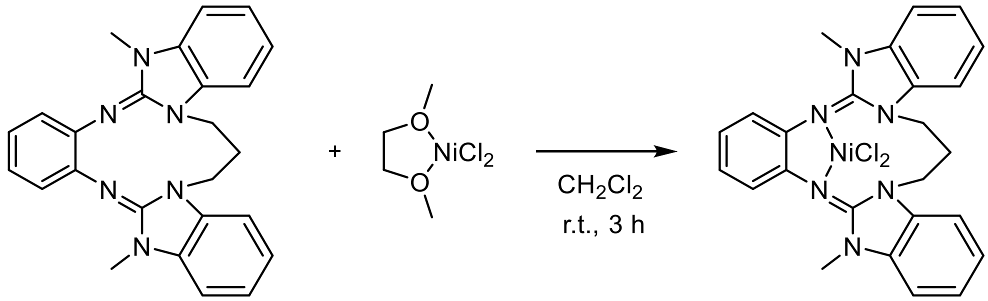

L2 (16.0 mg, 39.2 µmol, 1.0 eq.) and NiCl$_2$ x DME (8.32 mg, 39.2 µmol, 1.0 eq.) were suspended in DCM (3 mL). After 3 h the suspension was filtered, and the solvent removed *in vacuo*. After washing with ether (3x3 mL) the product was yielded as a red solid (16 mg, 30.3 µmol, 83%). Crystals suitable for structural analysis were grown through slow evaporation of diethylether in a saturated DCM solution.

MS (ESI$^+$DCM): *m/z* = 409.2140 ([L1+H]$^+$, calc: 409.2141).

EA (C, H, N): $C_{25}H_{24}Cl_2N_6Ni$ (x1 DCM): found: C 50-53, H 5.42, N 13.04.
calc: C 50.12, H 4.21, N 13.49.

UV/Vis (DCM): λ(ε) = 455 (666), 319 (13307), 294 (10508), 285 (10069), 227 (23723) nm (M^{-1} cm^{-1}).

CV (DCM, [*n*Bu$_4$N][PF$_6$] 100 mV/s, vs. Fc/Fc$^+$): $E_{Ox}{}^1$: 296 mV, $E_{Ox}{}^2$: 669 mV, $E_{Red}{}^1$: 103 mV.

Table 5: Selected bondlengths of [NiCl$_2$(L2)].

Bond	Distance [Å]	
Ni-(N11,C14,C13,N12) plane	0.743	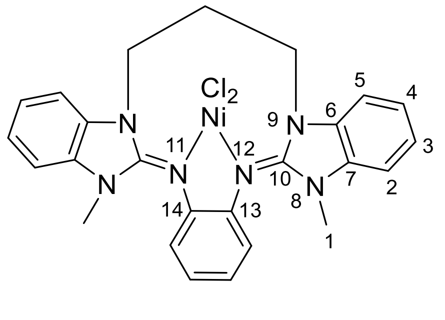
Ni-Cl	2.2219(18)/<br>2.2451(18)	
C10-N12	1.323(9)	
C10-N9/C10-N8	1.359(8)/1.364(9)	
Ni-N12/Ni-N11	2.006(6)/2.003(5)	

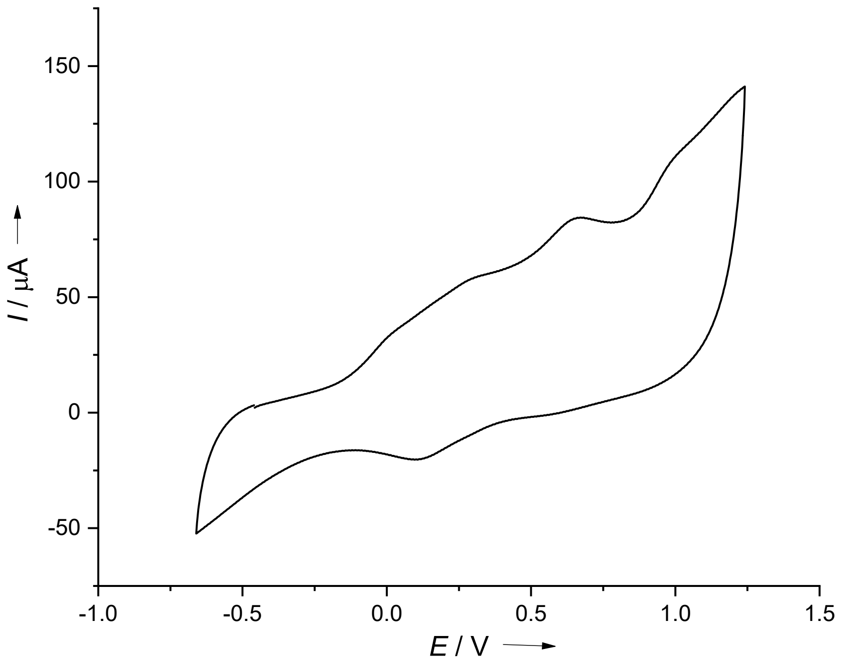

Figure 55: CV curve of [NiCl$_2$(L2)] (Fc/Fc$^+$ reference, 100 mV/s, nBu$_4$NPF$_6$, Ag/AgCl electrode, DCM).

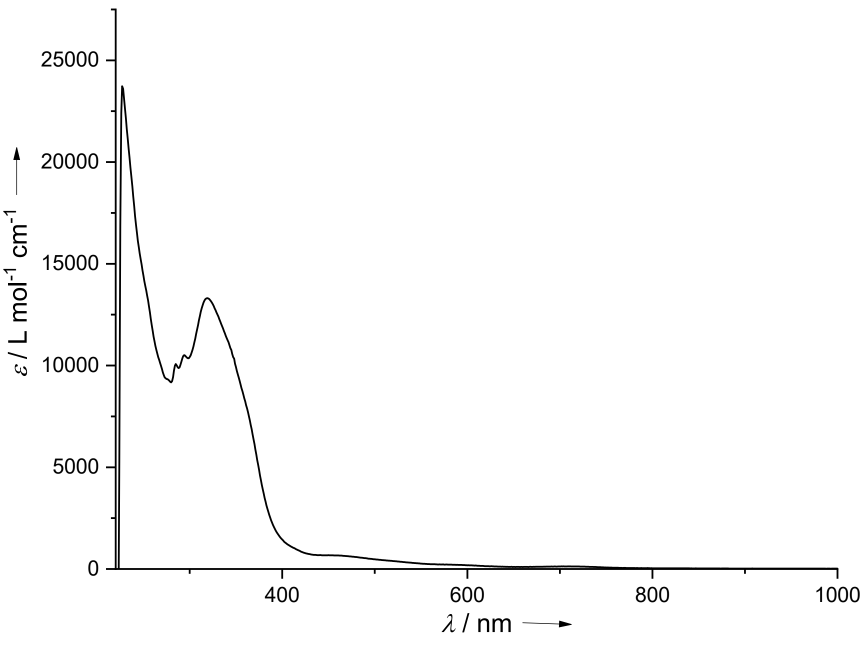

Figure 56: UV/Vis spectrum of [NiCl$_2$(L2)] in DCM.

[CuCl$_2$(L2)]

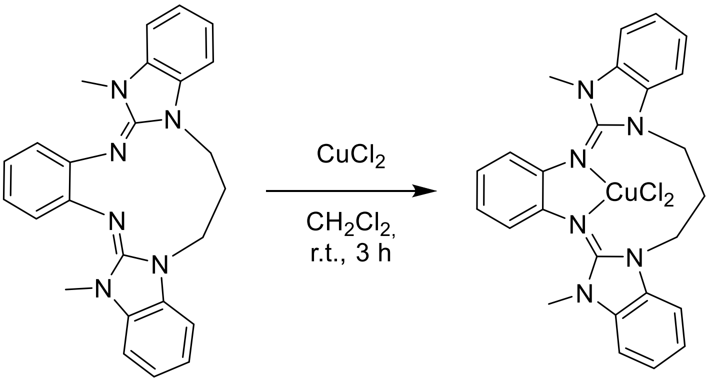

L2 (20 mg, 48.9 µmol, 1,0 eq.) and CuCl$_2$ (6.6 mg, 48.9 µmol, 1,0 eq.) were suspended in DCM (3 mL). After 3 h the suspension was filtered, and the solvent removed *in vacuo*. After washing with ether (3x3 mL) the product was yielded as a brown solid (22 mg, 40.52 µmol, 82%).

MS (ESI$^+$ in DCM): *m/z* = 409.2146 ([L1+H]$^+$, calc: 409.2141).

EA (C, H, N in %) (*1 DCM) $C_{25}H_{24}N_6CuCl_2$: calc: C: 49.74, H 4.17, N 13.38
found: C 48.92, H 4.48, N 13.18.

CV (DCM, [nBu$_4$N][PF$_6$] 100 mV/s, vs. Fc/Fc$^+$): E_{ox} = 0.437, E_{Red} = 0.271, 0.158, -0.165, -0.649.

UV/Vis (DCM): λ (ε) = 285 (12592), 345 (9781), 472 (2263), 720 (569) nm (M^{-1} cm^{-1}).
(MeCN) λ (ε) = 283 (13486), 315 (10135), 507 (1946), 763 (750) nm (M^{-1} cm^{-1}).

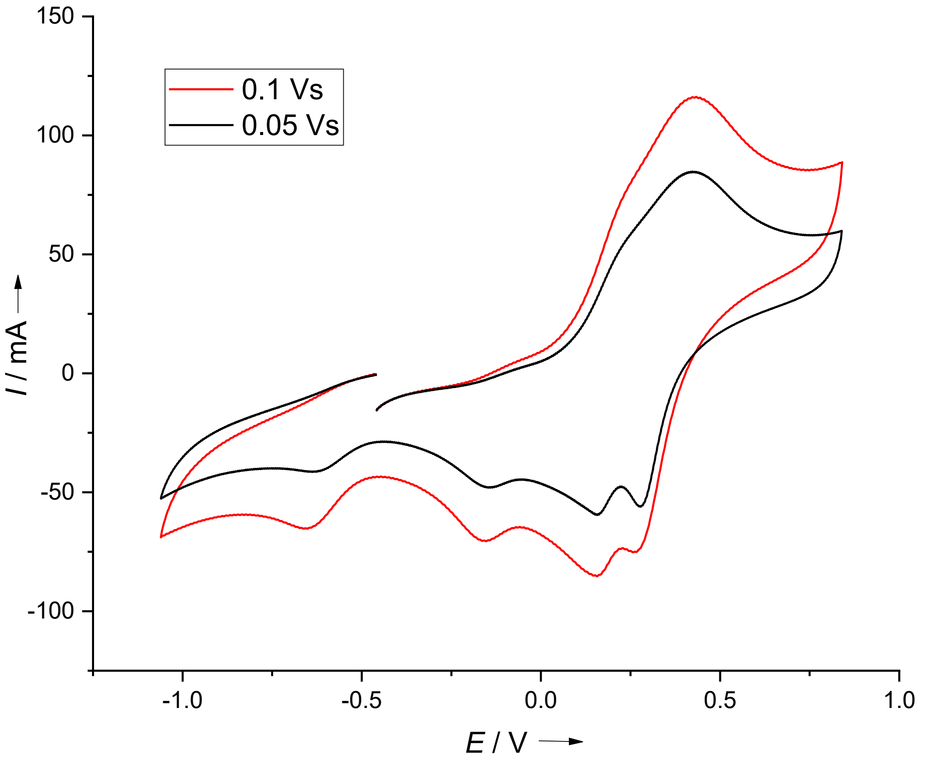

Figure 57: CV curve of [CuCl$_2$(L2)] (Fc/Fc$^+$ reference, 100 mV/s/50 mV/s, *n*Bu$_4$NPF$_6$, Ag/AgCl electrode, DCM).

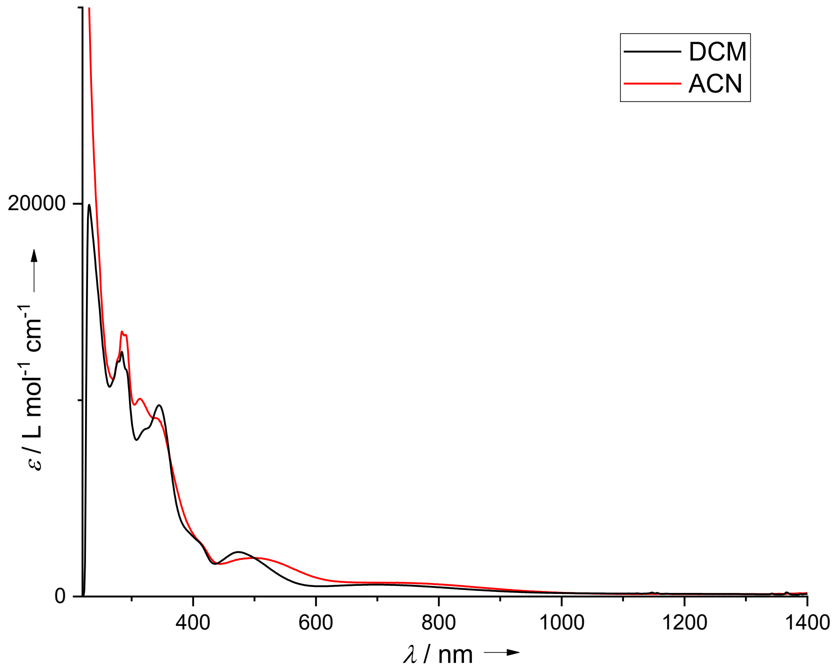

Figure 58: UV/Vis spectrum of [$CuCl_2$(L2)] in DCM and MeCN.

[CuBr2(L2)]

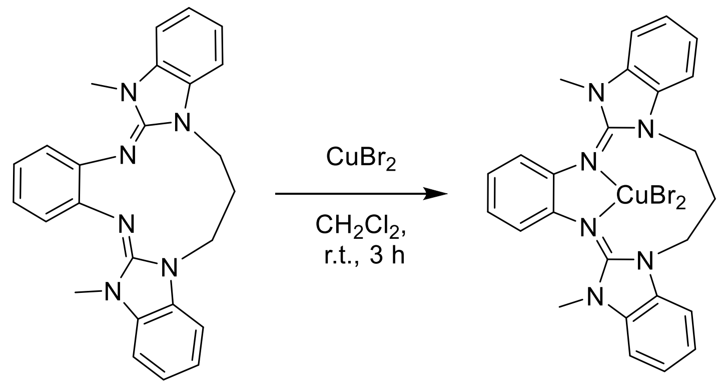

L2 (14.0 mg, 34.3 µmol, 1.0 äq.) and $CoBr_2$ (7.7 mg, 34.3 µmol, 1.0 eq.) were suspended in DCM (3 mL). After 3 h the solvent was removed *in vacuo*. After washing with ether (3x3 mL) the product was yielded as a violet solid (19 mg, 30.1 µmol, 88%).

MS (ESI^+ in DCM) *m/z* = 409.2146 ($[L1+H]^+$, calc.: 409.2141).

EA (C, H, N in %) $C_{25}H_{24}Br_2CuN_6$: found : C 47.49, H 4.01, N 12.49.
calc.: C 47.52, H 3.83, N 13.30.

CV (DCM, [*n*Bu_4N][PF_6] 100 mV/s, vs. Fc/Fc^+): $E_{Ox}{}^1$=435 mV, $E_{Ox}{}^2$=716 mV, $E_{Ox}{}^3$=1080 mV, $E_{Red}{}^1$=-653 mV, $E_{Red}{}^2$=110 mV, $E_{Red}{}^3$=196 mV, $E_{Red}{}^4$=377 mV.

UV/Vis-Spectrum (DCM): *λ* (ε) = 776 (850), 508 (1404), 402 (shoulder, 2469), 348 (5879), 322 (7951), 284 (10578) nm (M^{-1} cm^{-1}).

(MECN): *λ* (ε) = 476 (851), 410 (shoulder, 1114), 343 (3928), 314 (3643), 284 (4757) nm (M^{-1} cm^{-1}).

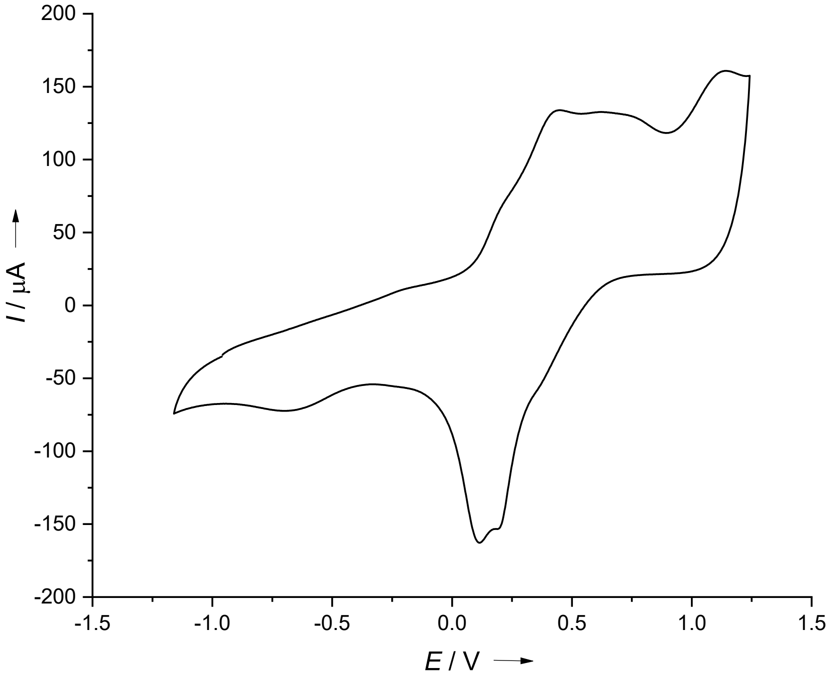

Figure 59: CV curve of [$CuBr_2$(L2)] (Fc/Fc^+ reference, 100 mV/s, *n*Bu_4NPF_6, Ag/AgCl electrode, DCM).

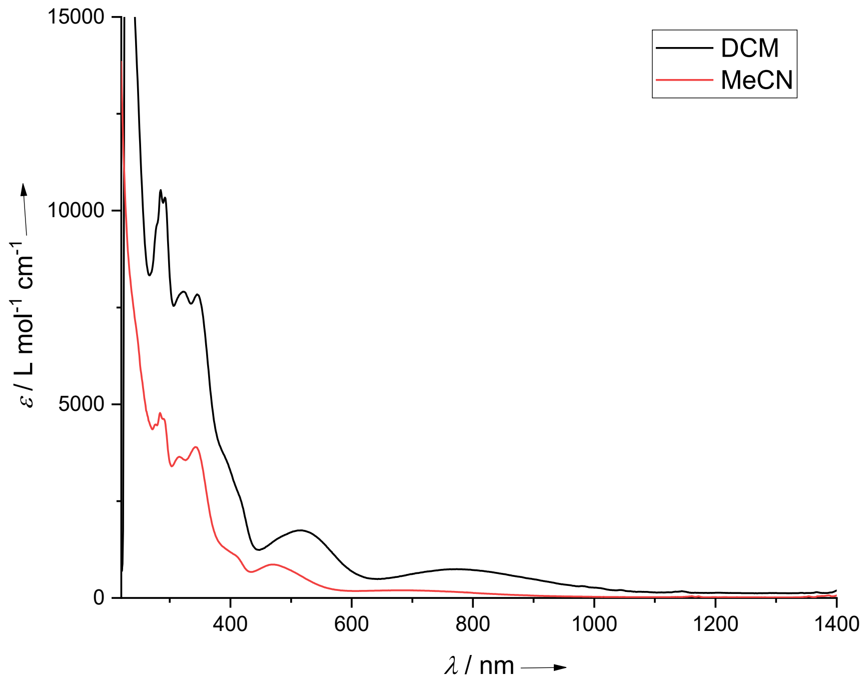

Figure 60: UV/Vis spectrum of $[CuBr_2(L2)]$ in DCM and MeCN.

[Cu(II)(BF4)2(L2)2]

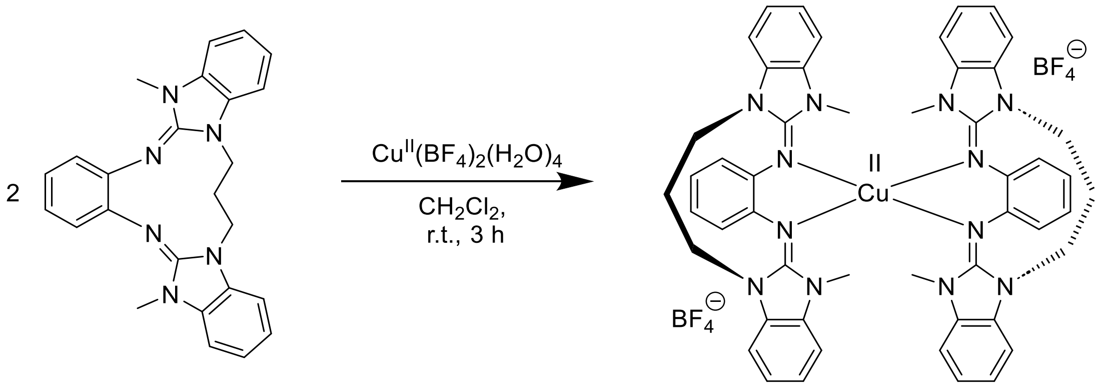

L2 (15.0 mg, 36.7 µmol, 2.0 eq.) and $Cu(BF_4)_2(H_2O)_4$ (5.7 mg, 18.4 µmol, 1.0 eq.) were suspended in DCM (3 mL). After 3 h the solvent was removed *in vacuo*. After washing with ether (3x3 mL) the product was yielded as a dark blue solid (15 mg, 17.0 µmol, 93%).

MS (ESI^+ in DCM): *m/z* = 879.3432 $[CuL1_2]^{2+}$ (calc: 879.3410).

EA (C, H, N in %) (*1 DCM) $C_{50}H_{48}B_2CuF_8N_{12}$: calc.: C 53.78, H 4.42, N 14.76
found: C 53.60, H 4.53, N 14.73.

CV (DCM, [*n*Bu_4N][PF_6] 50 mV/s, vs. Fc/Fc^+) $E_{Ox}{}^1$ = 600 mV, $E_{Ox}{}^2$ = 1.01 mV.

UV/Vis-Spectrum (DCM) λ (ε) = 316 (shoulder, 18349), 292 (21199), 285 (21281), 228 (42442) nm ($M^{-1}cm^{-1}$).

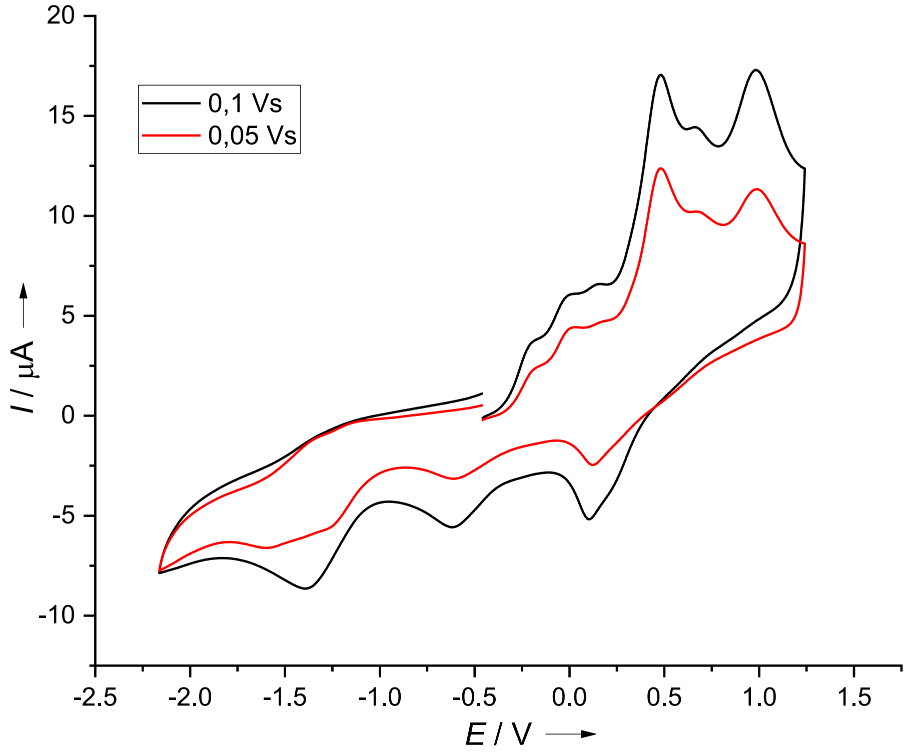

Figure 61: CV curve of $[Cu(BF_4)_2(L2)_2]$ (Fc/Fc^+ reference, 100 mV/s/50 mV/s, *n*Bu_4NPF_6, Ag/AgCl electrode, DCM).

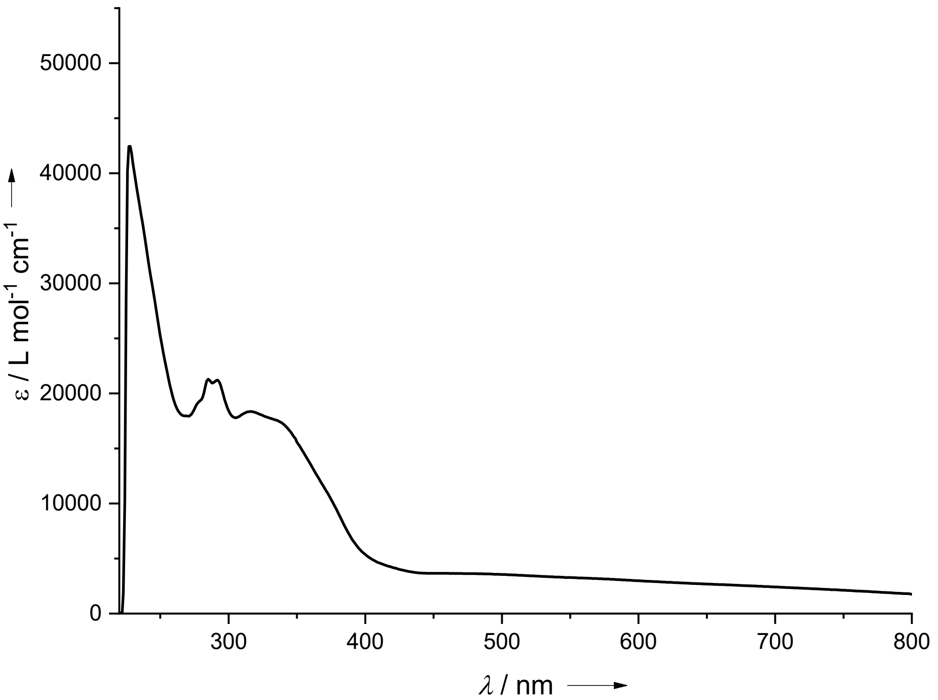

Figure 62: UV/Vis spectrum of $[Cu(BF_4)_2(L2)_2]$ in DCM.

[NiCl$_2$(L3)]

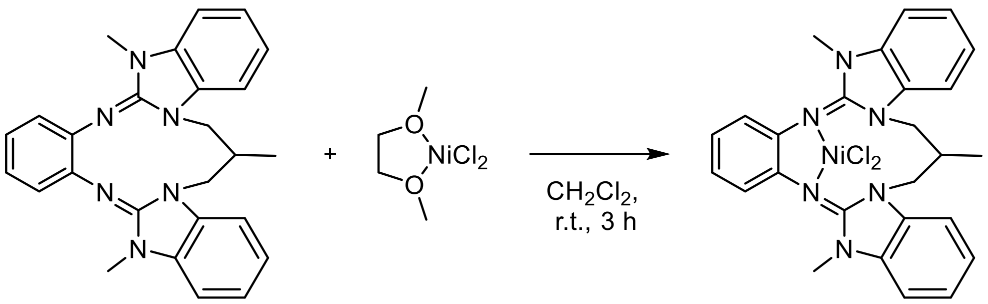

L3 (16.0 mg, 39.2 µmol, 1.0 eq.) and NiCl$_2$ x DME (8.62 mg, 39.2 µmol, 1,0 eq.) were suspended in DCM (3 mL). After 3 h the suspension was filtered, and the solvent removed *in vacuo*. After washing with ether (3x3 mL) the product was yielded as a red solid (17 mg, 30,2 µmol, 85%). Crystals for structural analysis were obtained out of a saturated DCM solution that was overlayered with diethylether.

MS (ESI$^+$ DCM): *m/z* = 423.2295 ([L2+H]$^+$, calc.: 423.2297).

EA (C, H, N): $C_{26}H_{26}Cl_2N_6Ni$ x (x1 DCM): found: C 50.09, H 5.41, N 12.95.
calc.: C 50.91, H 4.43, N 13.19.

UV/Vis (DCM): $\lambda(\varepsilon)$ = 463 (546), 324 (10717), 293 (8782), 285 (8700), 228 (19476) nm (M^{-1} cm^{-1}).

CV (DCM, [*n*Bu$_4$N][PF$_6$] 100 mV/s, vs. Fc/Fc$^+$): E_{Ox}^1=11 mV, E_{Ox}^2= 177 mV, E_{Ox}^3= 660 mV, E_{Ox}^4=995 mV, E_{Red}^1= 67 mV, E_{Red}^2= 473 mV.

Table 6: Selected bondlengths of [NiCl$_2$(L3)].

Bond	Distance [Å]	
Ni-(N11,C14,C13,N12) plane	0.713	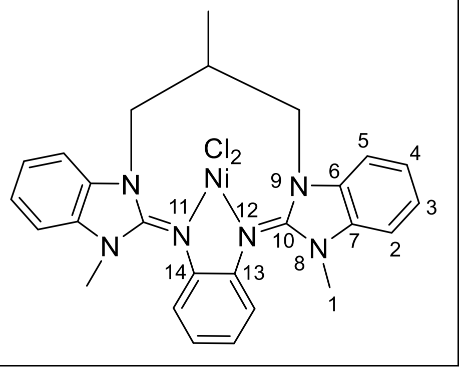
Ni-Cl	2.2316(7)/ 2.2383(8)	
C10-N12	1.322(3)	
C10-N9/C10-N8	1.370(3)/1.359(3)	
Ni-N12/Ni-N11	2.004(2)/2.019(2)	

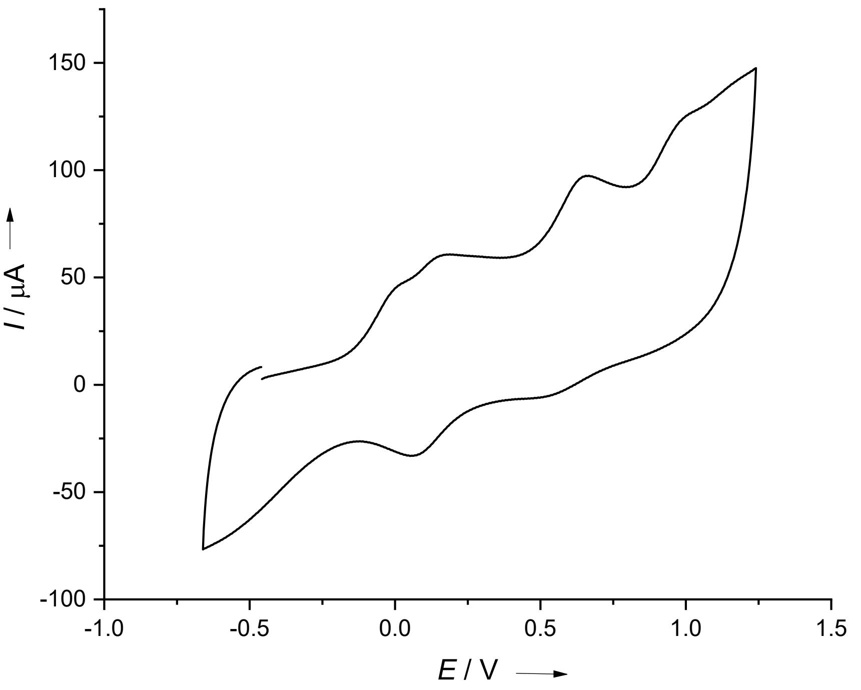

Figure 63: CV curve of [$NiCl_2$(L3)] (Fc/Fc^+ reference, 100 mV/s, *n*Bu_4NPF_6, Ag/AgCl electrode, DCM).

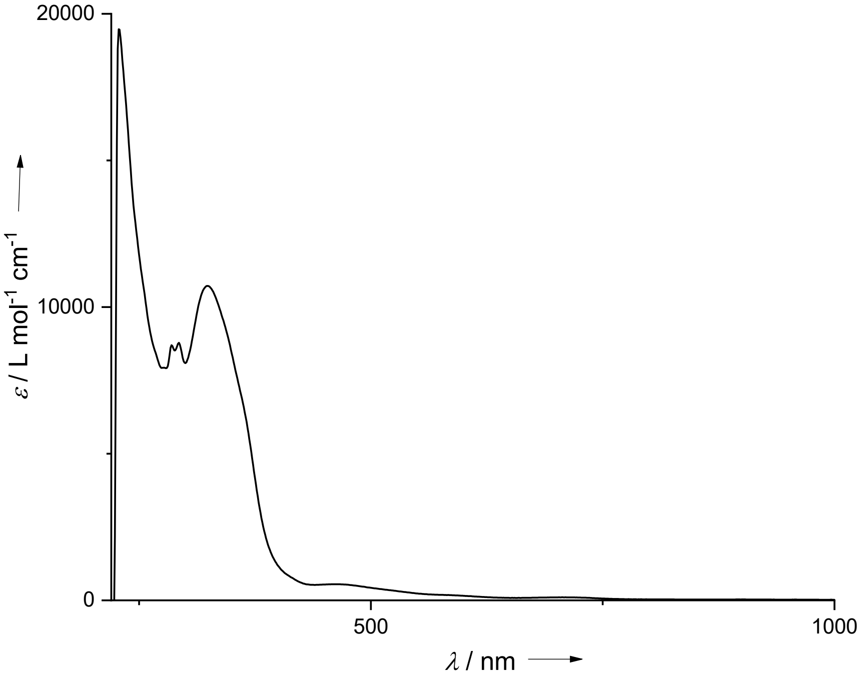

Figure 64: UV/Vis spectrum of [$NiCl_2$(L3)] in DCM.

[$CuCl_2$(L3)]

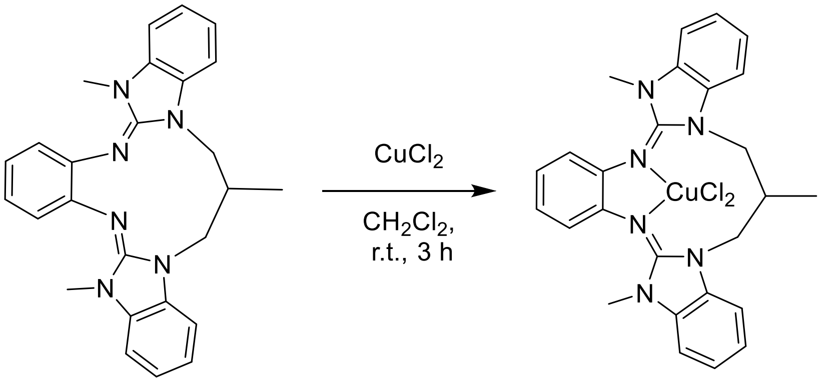

L3 (20 mg, 47.3 µmol, 1.0 eq.) and $CuCl_2$ (6.37 mg, 47.3 µmol, 1.0 eq.) were suspended in DCM (3 mL). After 3 h the suspension was filtered, and the solvent removed *in vacuo*. After washing with ether (3x3 mL) the product was yielded as a brown solid (21 mg, 37.7 µmol, 80%).

MS (ESI^+ in DCM): *m/z* = 409.2146 ($[L1+H]^+$, calc: 409.2141).

EA (C, H, N in %) (x2.1 DCM) $C_{25}H_{24}N_6CuCl_2$: calc: C: 44.9, H 4.14, N 11.43
found: C 45.3, H 4.57, N 11.53.

CV (DCM, [*n*Bu_4N][PF_6] 100 mV/s, vs. Fc/Fc^+): E_{ox} = 0.401 E_{Red} = 0.201, 0.110 V.

UV-Vis-Spectrum (DCM): λ (ε) = 278 (9195), 284 (9507), 319 (6584), 344 (7001), 473 (1914) nm (M^{-1} cm^{-1}) (MeCN) λ (ε) = 276 (9126), 284 (9369), 319 (6584), 342 (8616), 466 (2287) nm (M^{-1} cm^{-1}).

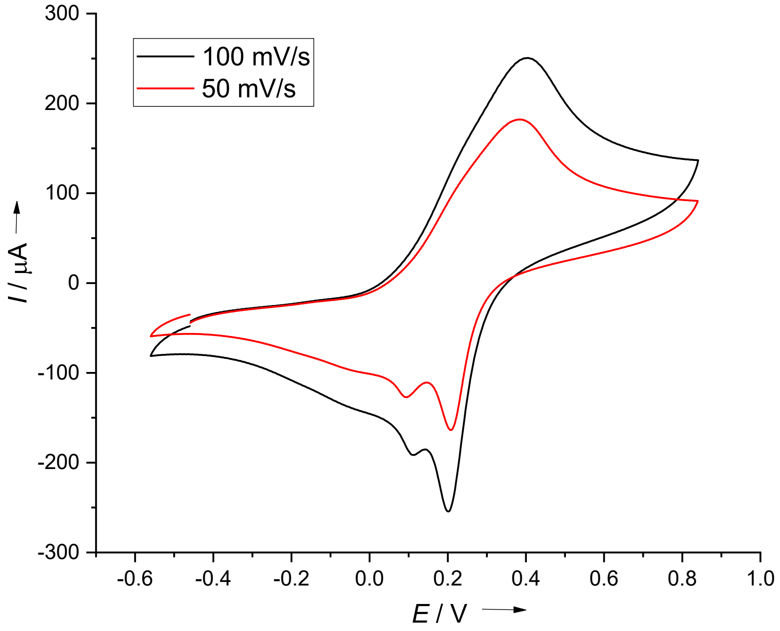

Figure 65: CV curve of [$CuCl_2$(L3)] (Fc/Fc^+ reference, 100 mV/s, *n*Bu_4NPF_6, Ag/AgCl electrode, DCM).

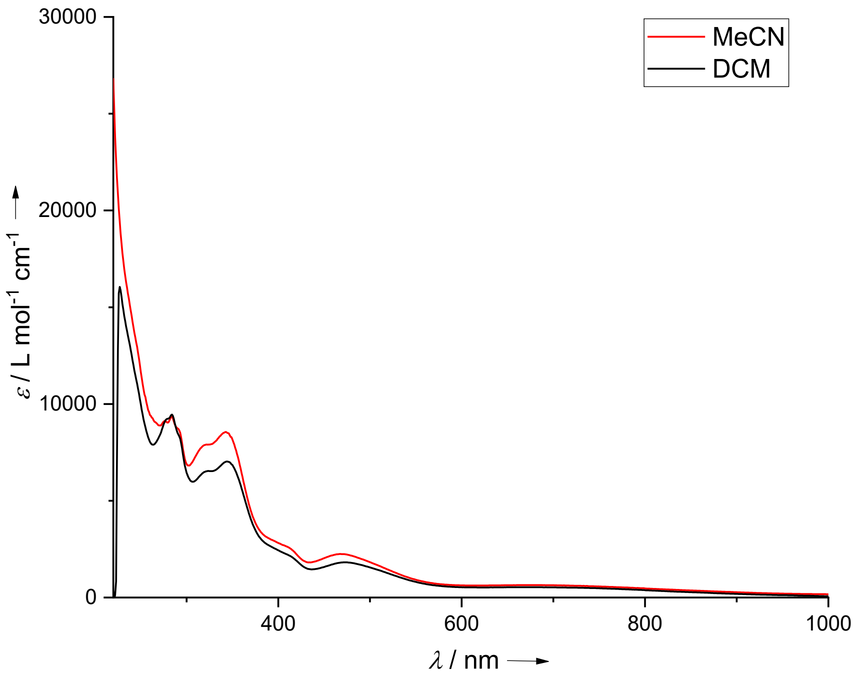

Figure 66: UV/Vis spectrum of [$CuCl_2$(L3)] in DCM and MeCN.

[$CuBr_2$(L3)]

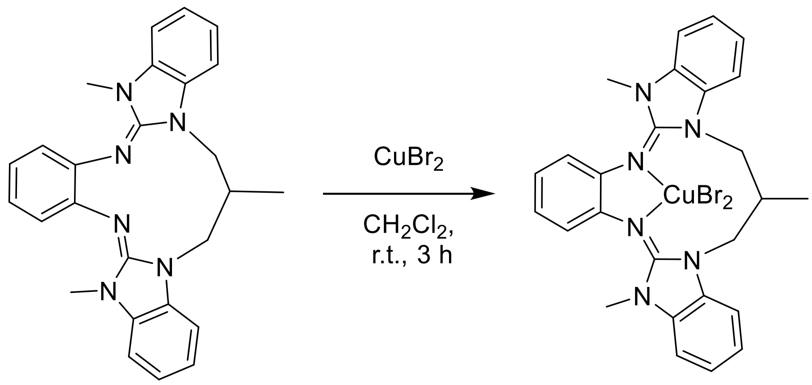

L2 (14.0 mg, 33.1 µmol, 1.00 äq.) and $CuBr_2$ (7.40 mg, 33.1 µmol, 1.00 eq.) were suspended in DCM (3 mL). After 3 h the solvent was removed *in vacuo*. After washing with ether (3x3 mL) the product was yielded as a violet solid (20.1 mg, 31.6 µmol, 92%).

MS (ESI^+ in DCM): *m/z* = 423.2299 $[L2+H]^+$ (calc: 423.2297).

EA (C, H, N in %) (x1 DCM) $C_{26}H_{26}Br_2CuN_6$: found: C 44.47, H 4.25, N 11.63.
calc: C 44.37, H 3.86, N 11.50.

CV (DCM, [*n*Bu_4N][PF_6] 100 mV/s, vs. Fc/Fc^+) E_{Ox}^1=442 mV, E_{Ox}^2=628 mV, E_{Ox}^3=1112 mV, E_{Red}^1= -678 mV, E_{Red}^2= 99 mV, E_{Red}^3=361 mV.

UV/Vis-Spectrum (DCM) λ (ε) = 738 (783), 478 (2070), 405 (shoulder, 3537), 346 (8580), 317 (9114), 292 (shoulder, 9890), 285 (10457), 279 (shoulder, 10020), 227 (21859) nm (M^{-1} cm^{-1}).

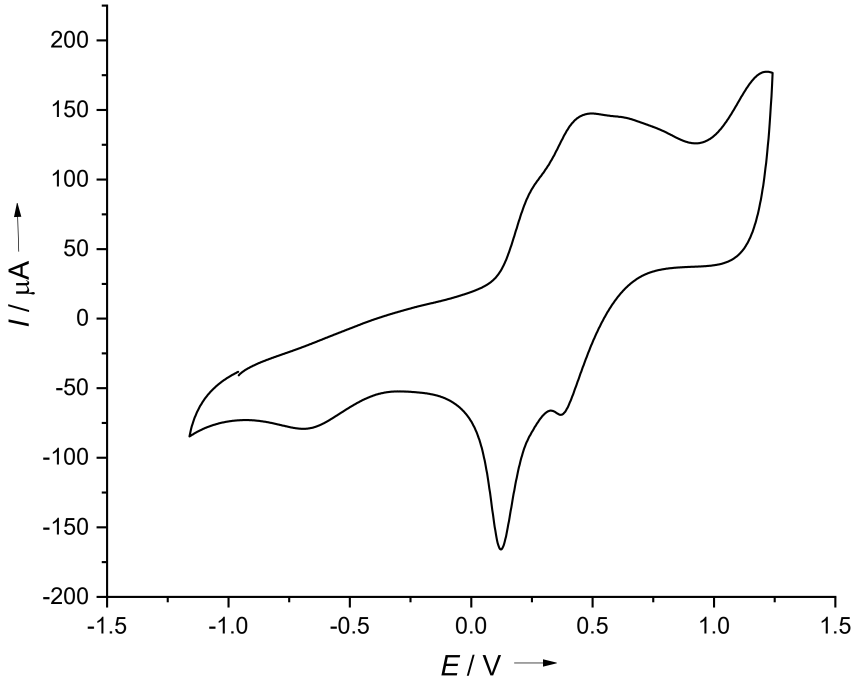

Figure 67: CV curve of [$CuBr_2$(L3)] (Fc/Fc^+ reference, 100 mV/s, *n*Bu_4NPF_6, Ag/AgCl electrode, DCM).

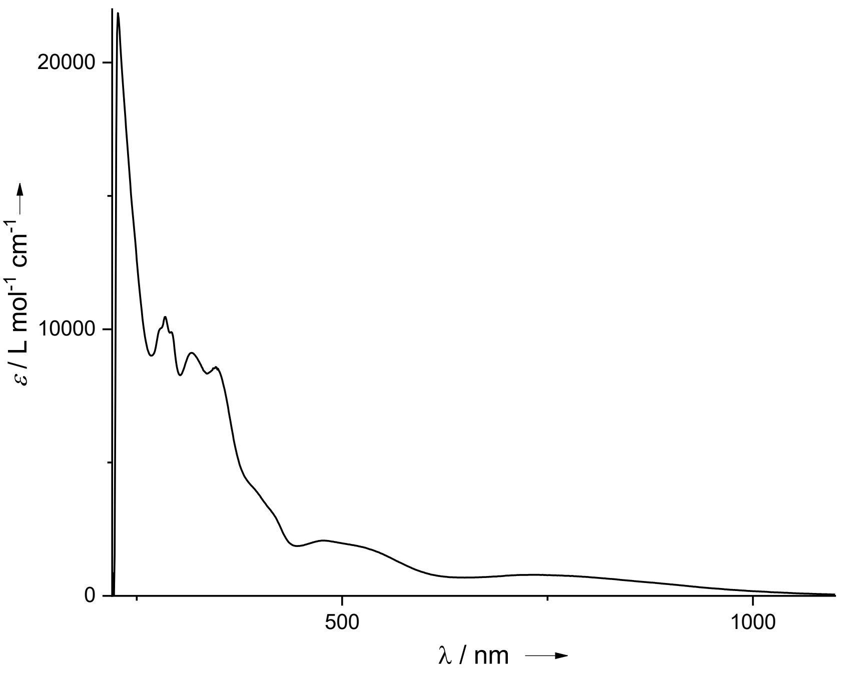

Figure 68: UV/Vis spectrum of $[CuBr_2(L3)]$ in DCM.

[Cu(II)(BF$_4$)$_2$(L3)$_2$]

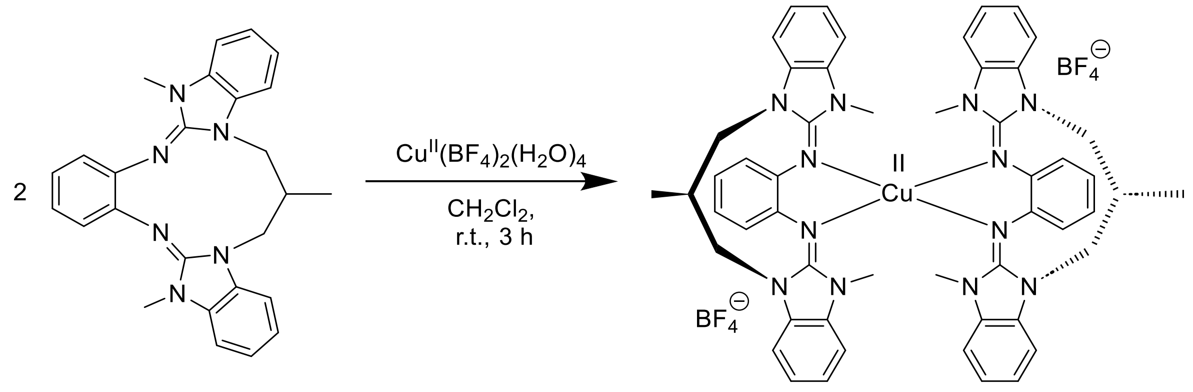

L3 (16.0 mg, 37.9 µmol, 2.0 eq.) and Cu(BF$_4$)$_2$(H$_2$O)$_4$ (6.5 mg, 18.9 µmol, 1.0 eq.) were suspended in DCM (3 mL). After 3 h the solvent was removed *in vacuo*. After washing with ether (3x3 mL) the product was yielded as a dark blue solid (15 mg, 17.0 µmol, 93%).

MS (ESI$^+$ in DCM): *m/z* = 907.3755 [M-2 BF$_4^-$]$^+$ (calc: 907.3734).

EA (C, H, N in %) (x1.5 DCM) $C_{52}H_{52}B_2CuF_8N_{12}$: found: C 53.48, H 4.96, N 13.65.
calc.: C 53.12, H 4.58, N 13.90.

CV (DCM, [*n*Bu$_4$N][PF$_6$] 50 mV/s, vs. Fc/Fc$^+$): $E_{Ox}{}^1$=-144 mV, $E_{Ox}{}^2$=573 mV, $E_{Ox}{}^3$=759 mV, $E_{Ox}{}^4$=1048 mV, $E_{Red}{}^1$=128 mV, $E_{Red}{}^2$=329 mV,

UV/Vis-Spectrum (DCM): λ(ε) = 595 (2021), 333 (19925), 285 (21170), 227 (44746) nm (M^{-1} cm^{-1}).

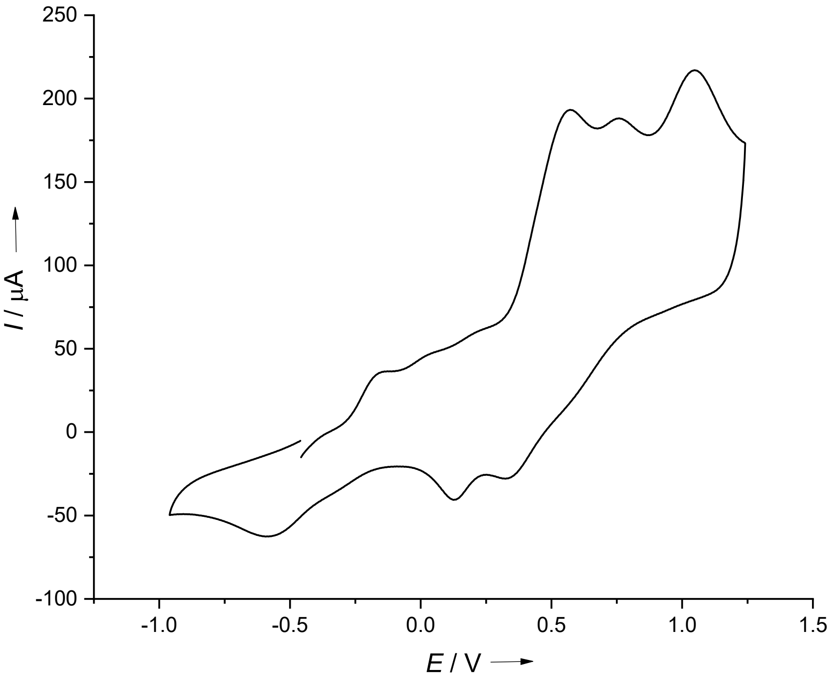

Figure 69: CV curve of [Cu(II)(BF$_4$)$_2$(L3)$_2$] (Fc/Fc$^+$ reference, 100 mV/s, *n*Bu$_4$NPF$_6$, Ag/AgCl electrode, DCM).

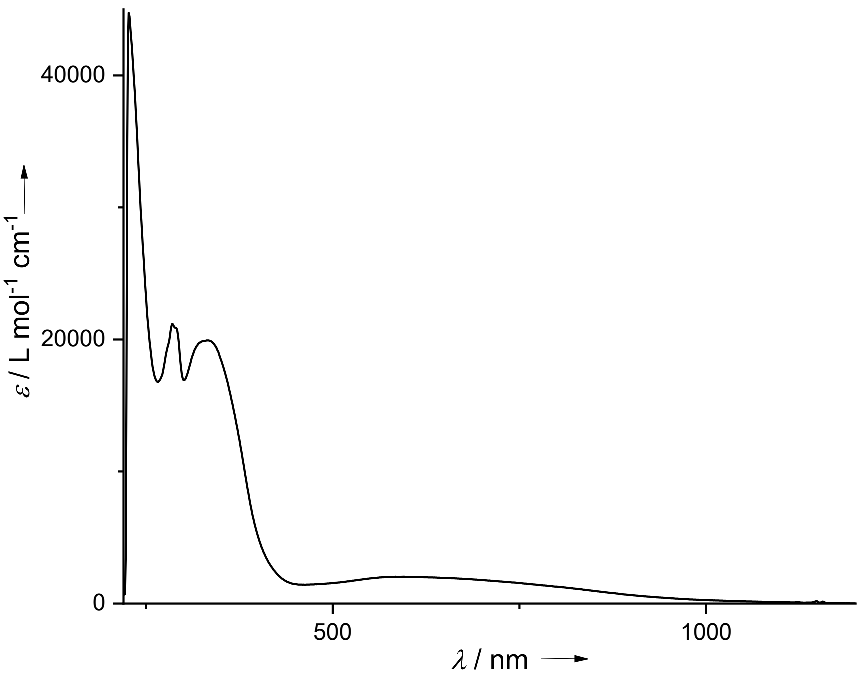

Figure 70: UV/Vis spectrum of [Cu(II)$(BF_4)_2$$(L3)_2$] in DCM.

[$CoBr_2$(L4)]

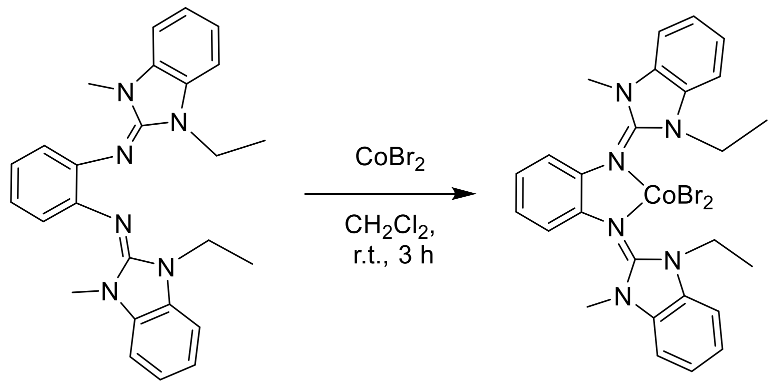

L4 (15 mg, 35.33 µmol, 1.0 eq.) and $CoBr_2$ (8.5 mg, 38.9 µmol, 1.1 eq.) were suspended in DCM (3 mL). After 3 h the suspension was filtered, and the solvent removed *in vacuo*. After washing with ether (3x3 mL) the product was yielded as a green solid (20.5 mg, 31.8 µmol, 90%). Crystals for structural analysis were obtained out of a saturated DCM solution that was overlayered with diethylether.

MS (ESI^+ in DCM): *m/z* = 641.0070 [$CoBr_2$L2]$^+$ (calc: 641.0074)

EA (C, H, N in %) (x1.2 DCM) $C_{25}H_{24}N_6CoBr_2$: calc: C 43.84, H 4.11, N 11.28
found: C 43.52, H 4.74, N 11.06.

CV (DCM, [$n$$Bu_4N$][$PF_6$], 100 mV/s, vs. Fc/$Fc^+$): $E_{1/2}$ = 0.1790 E_{ox} = 0.8808 V.

UV/Vis-Spectrum (DCM) λ (ε) = 229 (29180), 339 (25678), 570 (529, dd), 632 (251, dd), 691(337, dd) nm (M^{-1} cm^{-1})

Table 7: Selected bondlengths of [$CoBr_2$(L4)].

Bond	Distance [Å]
Co-(N11,C14,C13,N12) plane	0.645
Co-Br	2.3907(4)/
	2.3916(5)
C10-N12	1.324(3)
C10-N9/C10-N8	1.371(3)/1.362(3)
Co-N12/Co-N11	2.015(2)/2.010(2)

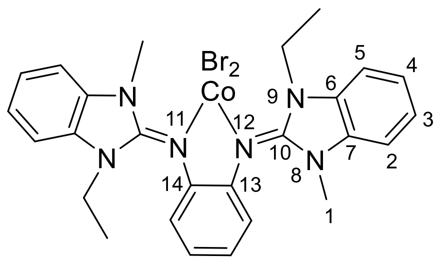

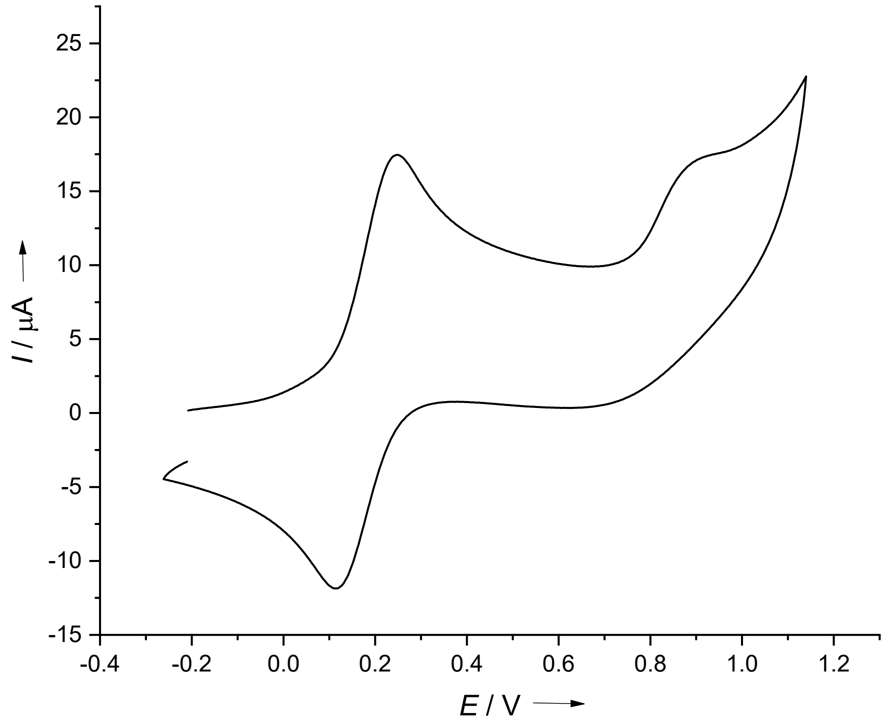

Figure 71: CV curve of [$CoBr_2$(L4)] (Fc/Fc^+ reference, 100mV/s, *n*Bu_4NPF_6, Ag/AgCl electrode, DCM)

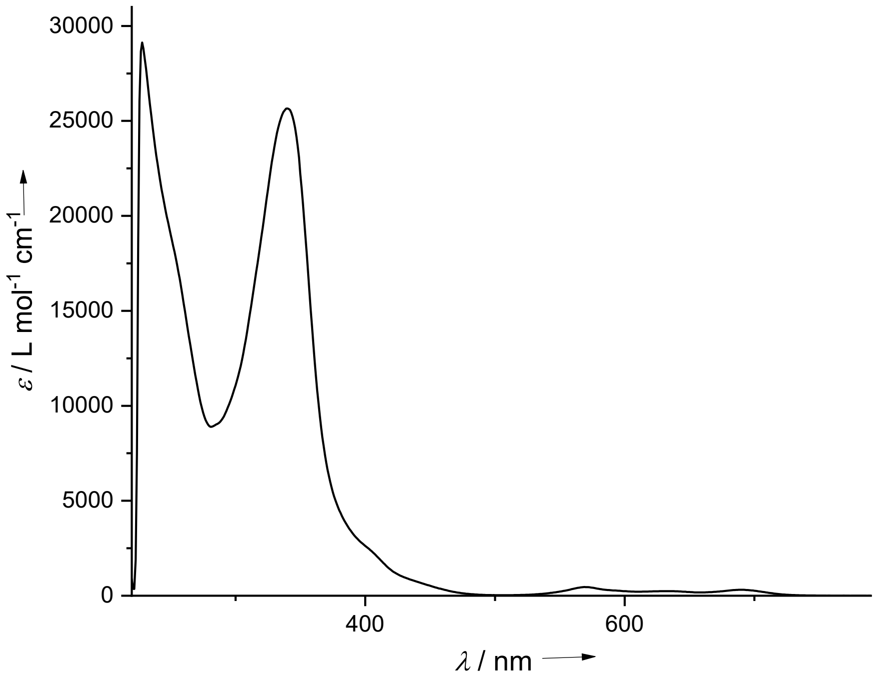

Figure 72: UV/Vis spectrum of [$CoBr_2$(L4)] in DCM.

$[NiCl_2(L4)]_3$

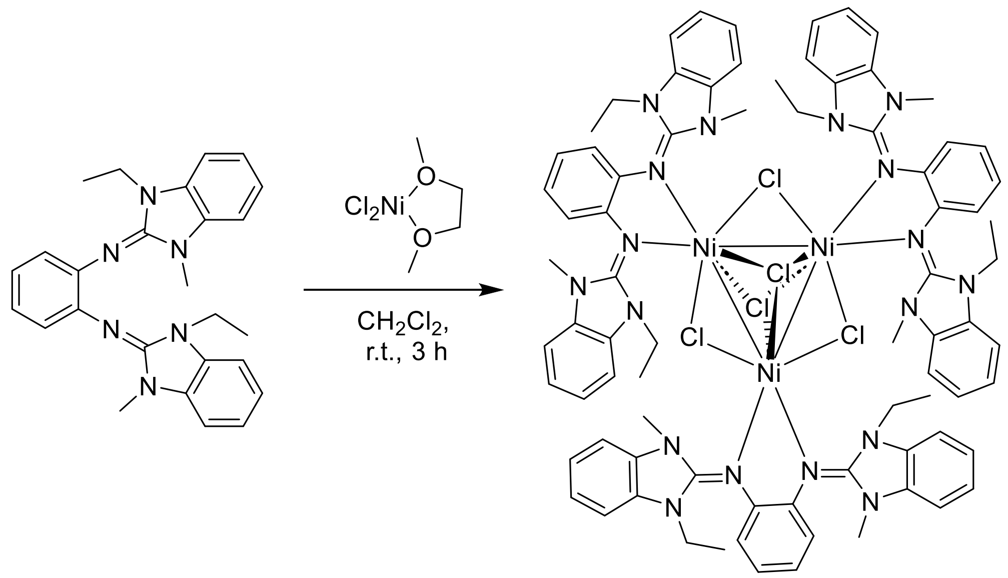

L4 (15.0 mg, 35.33 µmol, 1.0 eq.) and $NiCl_2$*DME (8.5 mg, 38.86 µmol, 1.1 eq.) were suspended in CH_3CN (4 mL). After 3 h the suspension was filtered, the solvent was removed *in vacuo*. After washing with ether (3x3 mL) the product was yielded as a red solid (18.0 mg, 32.5 µmol, 92%). Crystals for structural analysis were obtained through diffusion of Et_2O into a saturated CH_3CN solution.

MS (ESI^+ in DCM): 552.1106 [1/3 M^+], (calc. 522.1106 [1/3 M^+]).

EA (C, H, N in %) (x1 DCM) $(C_{26}H_{28}Cl_2N_6Ni)_3$: calc.: C 50.74, H 4.73, N 13.15
found: C 50.67, H 5.44, N 13.65.

CV (DCM, [*n*Bu_4N][PF_6] 100 mV/s, vs. Fc/Fc^+): $E_{1/2}$ = 0.086, $E_{1/2}$ = 0.568 V.

UV/Vis Spectrum (DCM): λ(ε) = 228 (46207), 336 (39639), 470 (2117) nm ($M^{-1}cm^{-1}$).

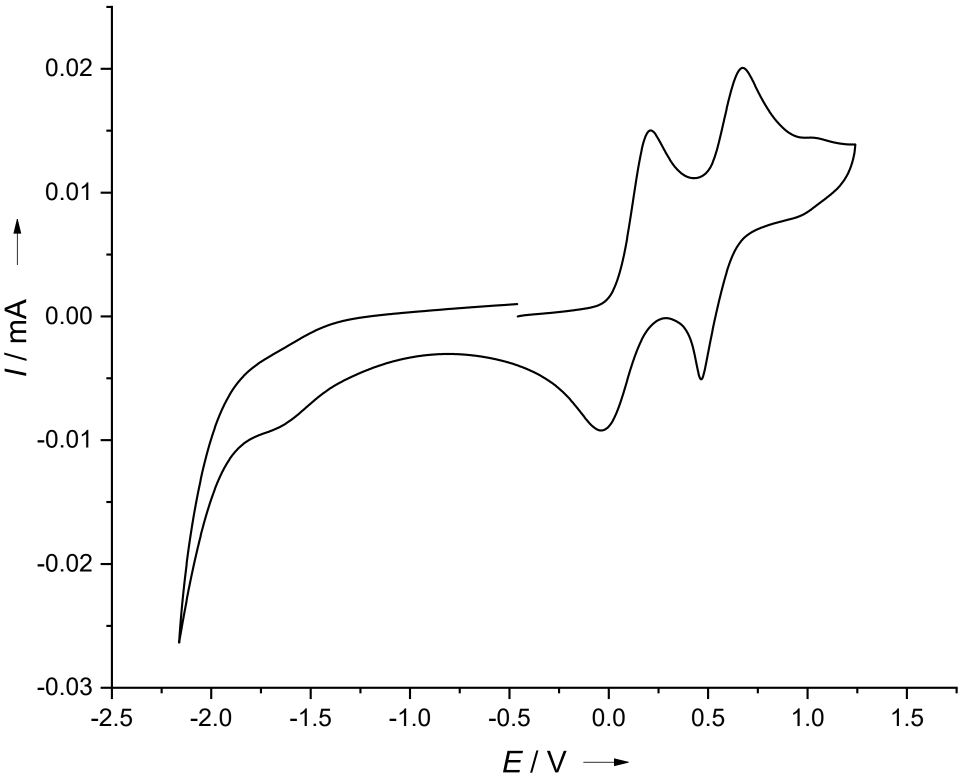

Figure 73: CV curve of $[NiCl_2(L4)]_3$ (Fc/Fc$^+$ reference, 100 mV/s, nBu$_4$NPF$_6$, Ag/AgCl electrode, DCM)

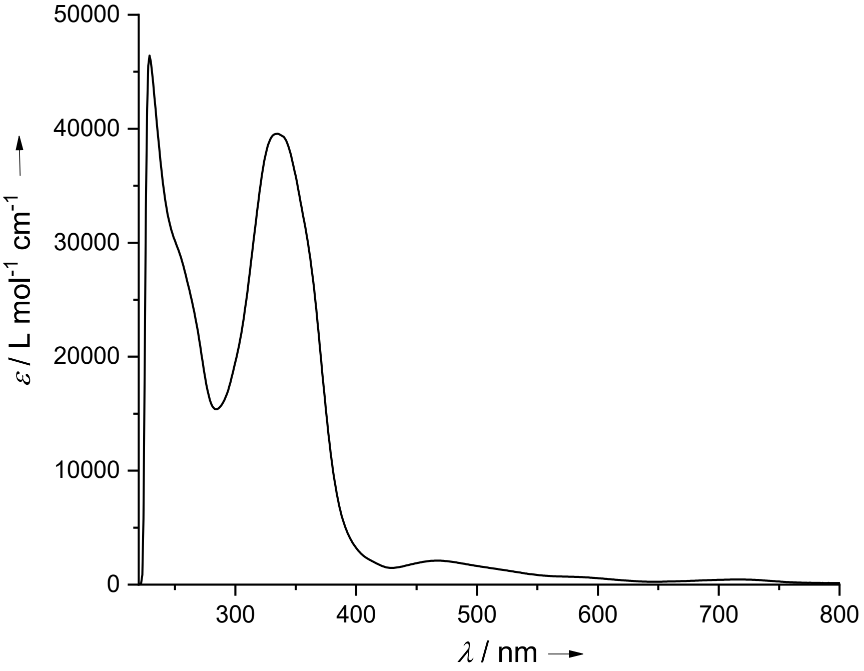

Figure 74: UV/Vis spectrum of $[NiCl_2(L4)]_3$ in MeCN.

[Cu(II)$(BF_4)_2$(L4)$_2$]

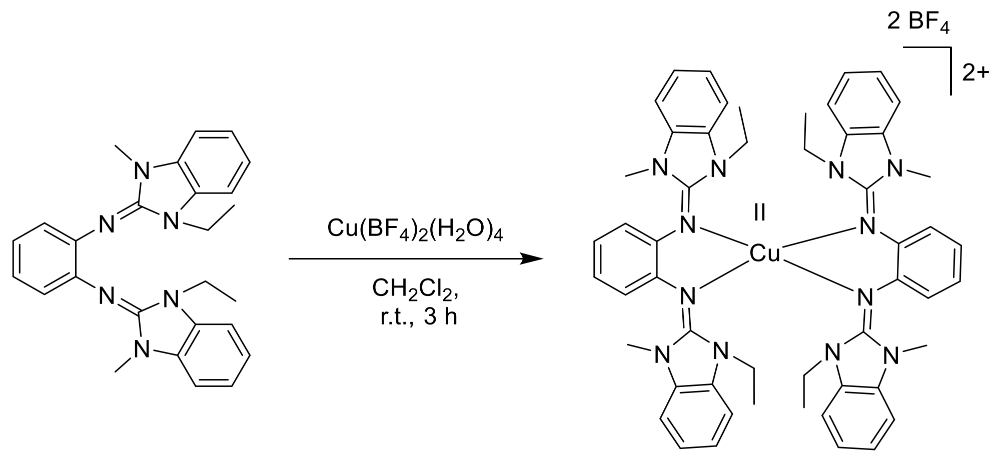

L4 (20.0 mg, 47.1 µmol, 2.0 eq.) and $Cu(BF_4)_2(H_2O)_4$ (7.3 mg, 23.6 µmol, 1.0 eq.) were suspended in DCM (3 mL). After 3 h the solvent was removed *in vacuo*. After washing with ether (3x3 mL) the product was yielded as a dark blue solid (19.5 mg, 21.4 µmol, 91%). Crystals for structural analysis were obtained through diffusion of Et_2O into a saturated CH_3CN solution.

MS (ESI^+ in DCM): 425.2446 [L+H], (calc. 424.2375 [L]), 911.4046 (calc. 911.4047 [M-2BF_4]).

EA (C, H, N in %) $C_{52}H_{56}N_{12}CuB_2F_8$: calc.: C: 57.50, H: 5.20, N: 15.47

found: C:56.95, H: 5.79, N: 15.14.

CV (DCM, [*n*Bu_4N][PF_6] 100 mV/s, vs. auf Fc/Fc^+): $E_{1/2}^1$ = 363 mV, $E_{1/2}^2$ = 216 mV, E_{Ox}^1 = 738 mV, E_{Ox}^2 = 956 mV, E_{Red}^1 = 618 mV, E_{Red}^2 = 901 mV.

UV/Vis Spectrum (DCM)**:** λ(ε) = 581 (2859), 324 (31578), 276 (20418), 227 (60248) nm ($M^{-1}cm^{-1}$).

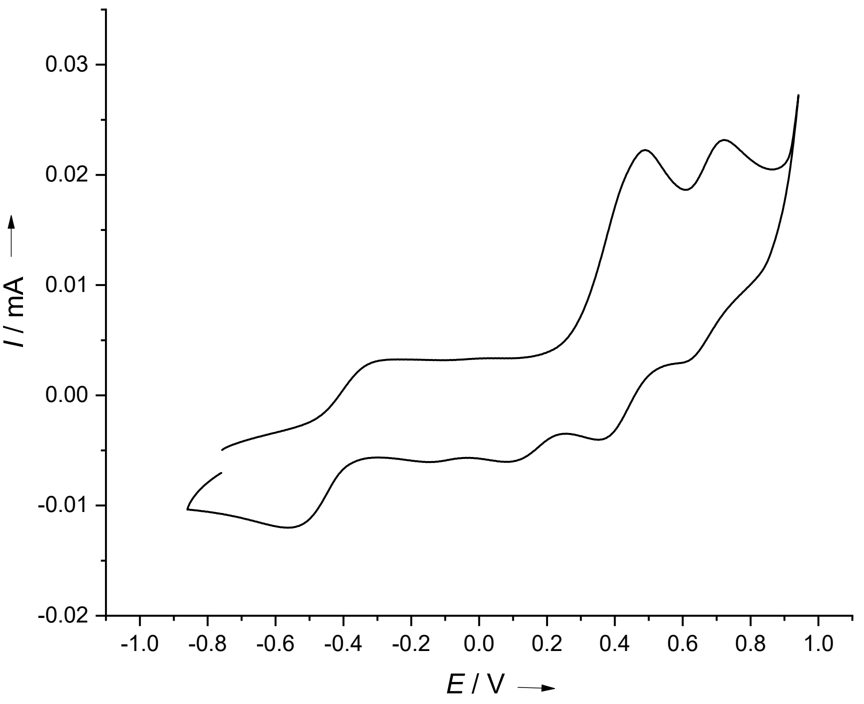

Figure 75: CV curve of $[Cu(BF_4)_2(L4)_2]$ (Fc/Fc^+ reference, 100 mV/s, $n$$Bu_4NPF_6$, Ag/AgCl electrode, DCM)

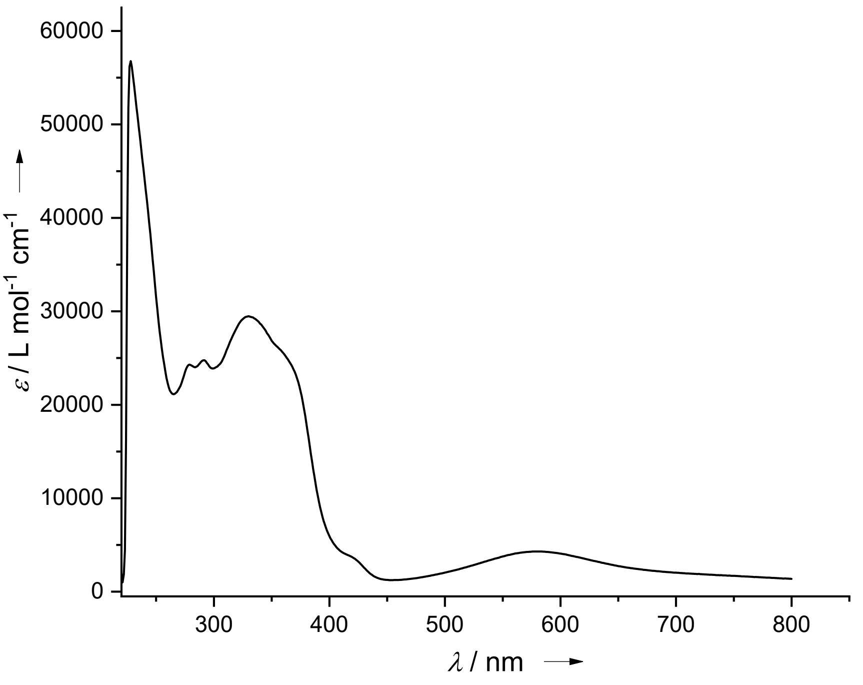

Figure 76: UV/Vis spectrum of $[Cu(BF_4)_2(L4)_2]$ in DCM.

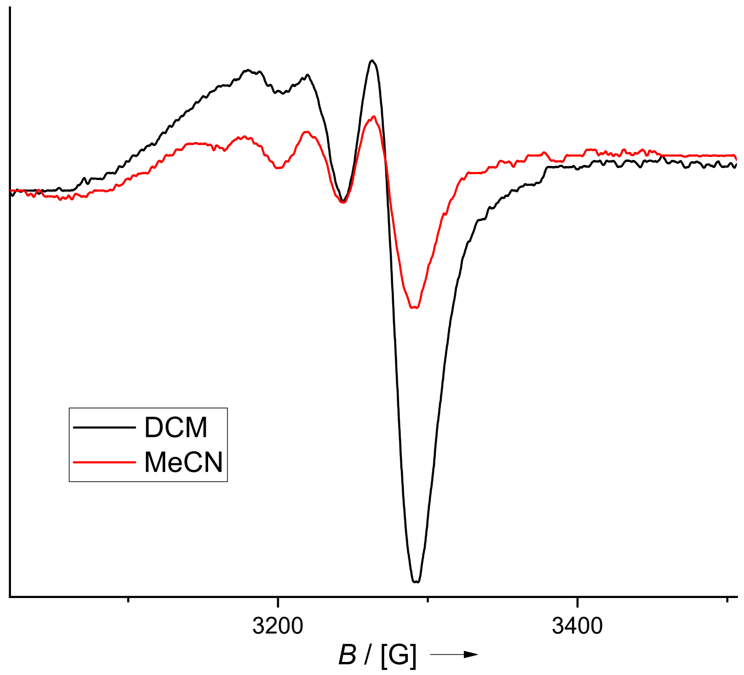

Figure 77: ESR of $[Cu(BF_4)_2(L4)_2]$ at r.t. (295 K) in DCM (9.448103 GHz, g=2.060) and MeCN (9.443915 GHz, g=2.060).

[$CoBr_2$(L5)]

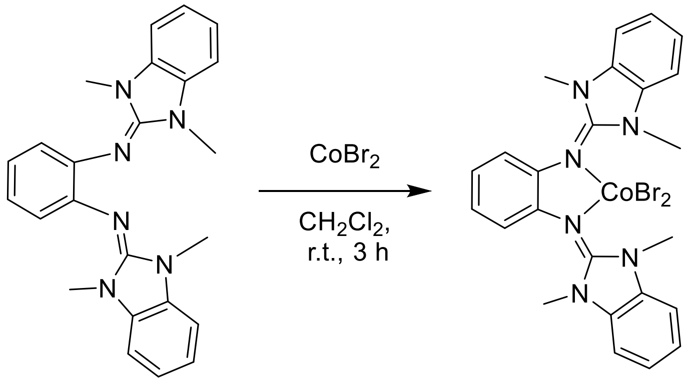

L5 (15.0 mg, 37.8 µmol, 1.0 eq.) and $CoBr_2$ (9.1 mg, 41.6 µmol, 1.1 eq.) were suspended in DCM (3 mL). After 3 h the suspension was filtered, and the solvent removed *in vacuo*. After washing with ether (3x3 mL) the product was yielded as a green solid (22.4 mg, 34.8 µmol, 92%). Crystals for structural analysis were obtained through slow diffusion with diethylether into a saturated DCM solution.

MS (ESI^+ in DCM): *m/z* = 534.0570 $[CoBrL2]^+$ (calc: 534.0578).

EA (C, H, N in %) (x0,8 DCM) $C_{25}H_{24}N_6CoBr_2$: calc: C: 43.66, H 3.78, N 12.30
found: C 43.75, H 4.11, N 12.55.

CV (DCM, [*n*Bu_4N][PF_6] 100 mV/s, vs. Fc/Fc^+): $E_{1/2}$ = 0.2988, E_{ox} = 0.8167 V.

UV/Vis-Spectrum (DCM) λ (ε) = 229 (35291), 339 (31458), 568 (614, dd), 688 (470, dd) nm (M^{-1} cm^{-1}).

Table 8: Selected bondlengths of [$CoBr_2$(L5)].

Bond	Distance [Å]	
Co-(N11,C14,C13,N12) plane	0.398	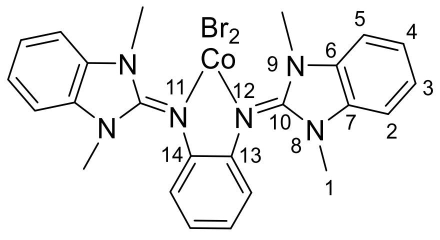
Co-Br	2.4007(6)/	
	2.3821(6)	
C10-N12	1.332(4)	
C10-N9/C10-N8	1.356(4)/1.367(4)	
Co-N12/Co-N11	2.003(3)/2.006(3)	

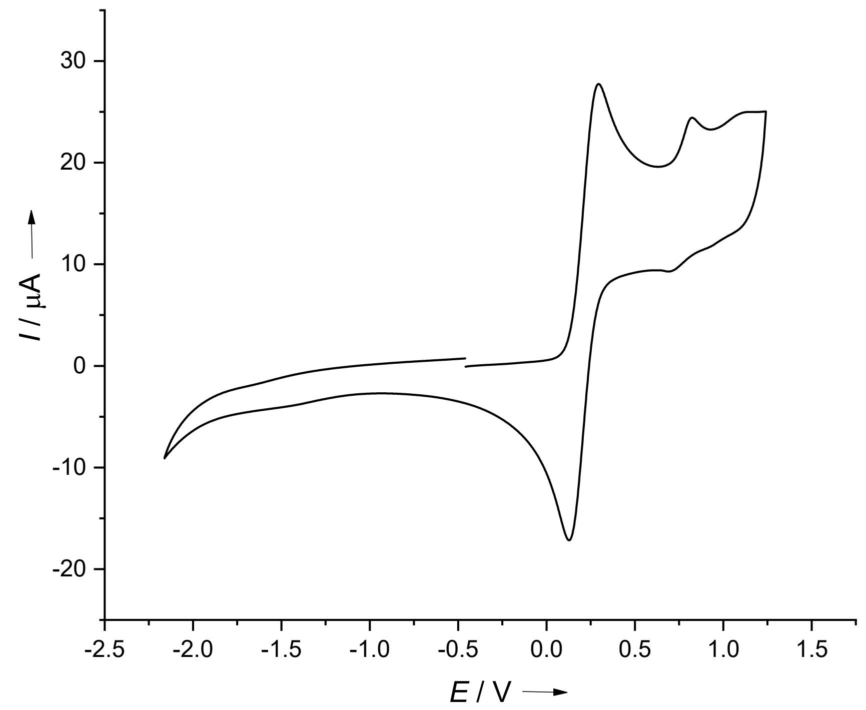

Figure 78: CV curve of $[CoBr_2(L5)]$ (Fc/Fc^+ reference, 100 mV/s, $n$$Bu_4NPF_6$, Ag/AgCl electrode, DCM).

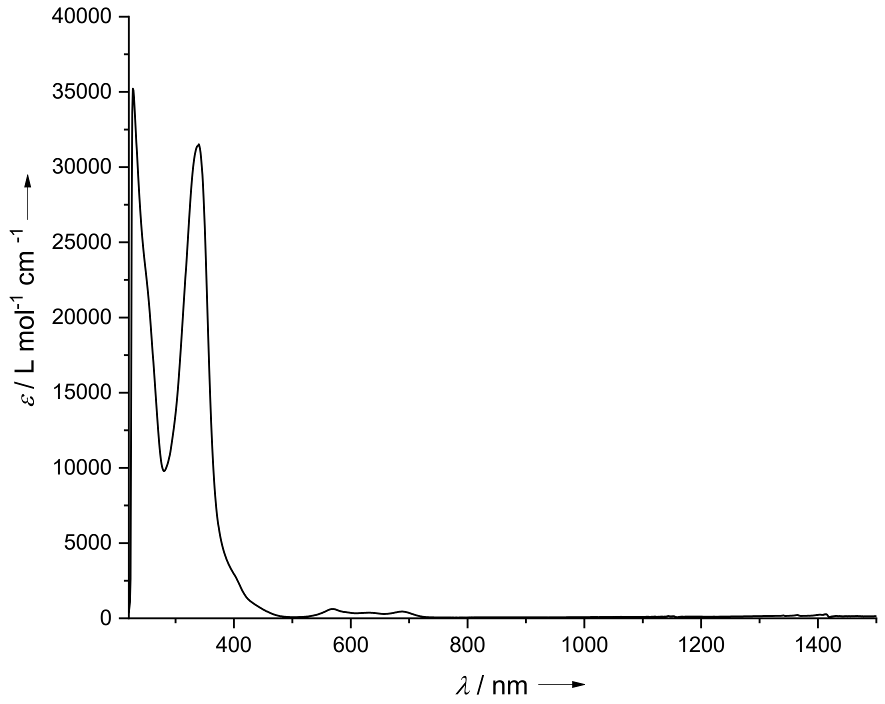

Figure 79: UV/Vis spectrum of $[CoBr_2(L5)]$ in DCM.

[Cu(II)(BF$_4$)$_2$(L5)$_2$]

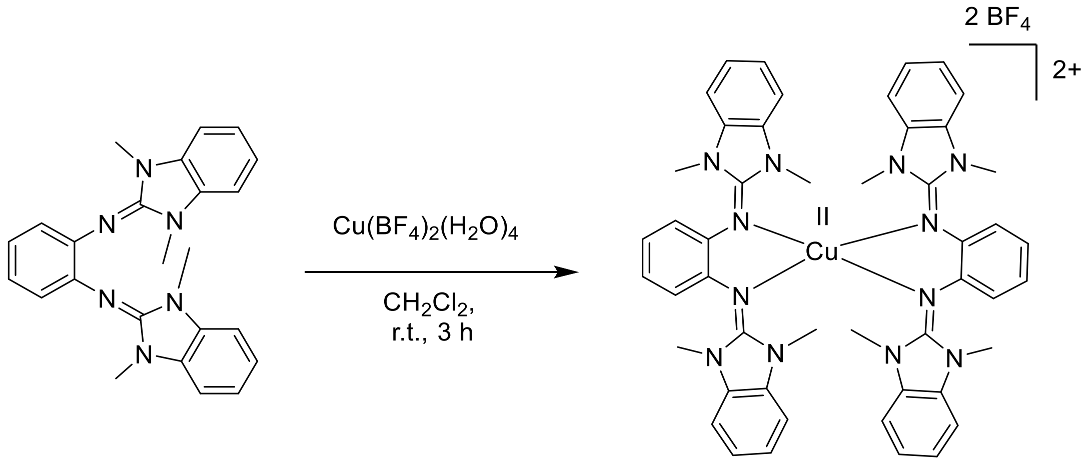

L5 (20.0 mg, 50.4 µmol, 2.0 eq.) and Cu(BF$_4$)$_2$(H$_2$O)$_4$ (7.8 mg, 25.2 µmol, 1.0 eq.) were suspended in DCM (3 mL). After 3 h the solvent was removed *in vacuo*. After washing with ether (3x3 mL) the product was yielded as a dark blue solid (22 mg, 21.4 µmol, 85%). Crystals for structural analysis were obtained through diffusion of Et$_2$O into a saturated CH$_3$CN solution.

MS (ESI$^+$ in DCM) *m/z*= 855.3420 [Cu(L2)$_2$]$^{2+}$ (calc: 855.3421).

EA (C, H, N in %) (x1.5 DCM) C$_{48}$H$_{48}$B$_2$CuF$_8$N$_{12}$: calc: C: 52.78, H: 4.52, N: 15.07
found: C: 52.48, H: 4.79, N: 15.19.

CV (DCM, [*n*Bu$_4$N][PF$_6$] 50 mV/s, ref. Fc/Fc$^+$): $E_{1/2}^1$ = -372 mV, $E_{1/2}^2$ = -41 mV, $E_{1/2}^3$ = 219 mV, $E_{1/2}^4$ = 429 mV, E_{Ox}^1 = 647 mV, E_{Ox}^2 = 832 mV, E_{Red}^1 = 580 mV, E_{Red}^1 = 777 mV.

UV/Vis spectrum (DCM) λ (ε) = 582 (3326), 329 (28165), 278 (Shoulder, 18807), 227 (52395) nm (M^{-1}cm^{-1}).

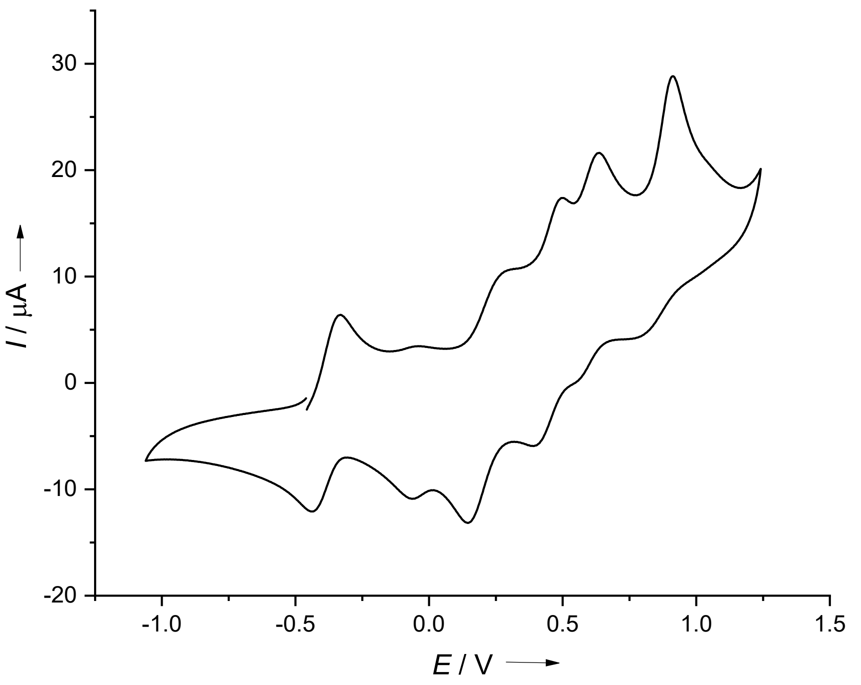

Figure 80: CV curve of $[Cu(BF_4)_2(L5)_2]$ (Fc/Fc^+ reference, 100 mV/s, nBu_4NPF_6, Ag/AgCl electrode, DCM).

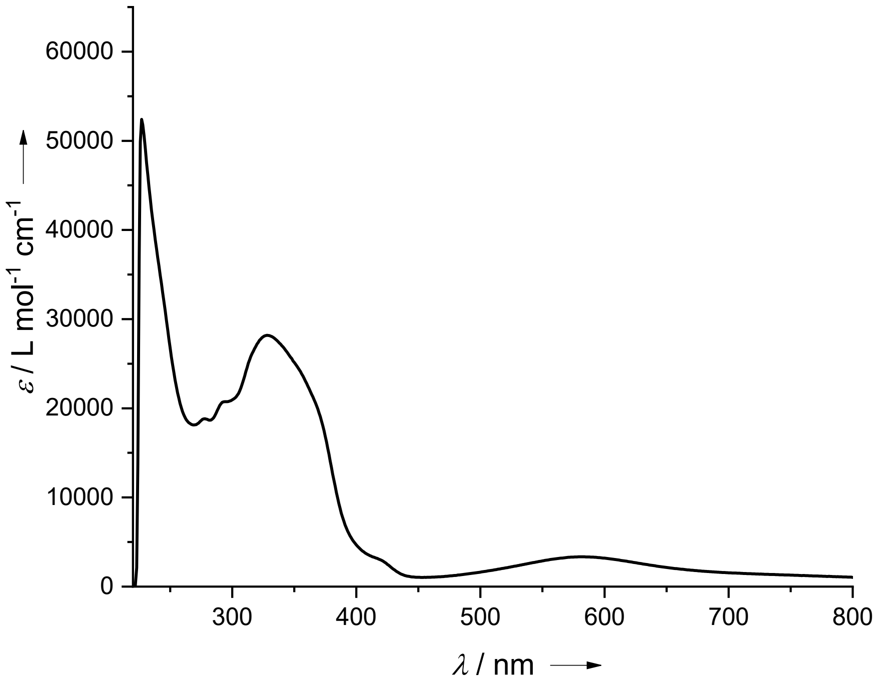

Figure 81: UV/Vis spectrum of $[Cu(BF_4)_2(L5)_2]$ in DCM.

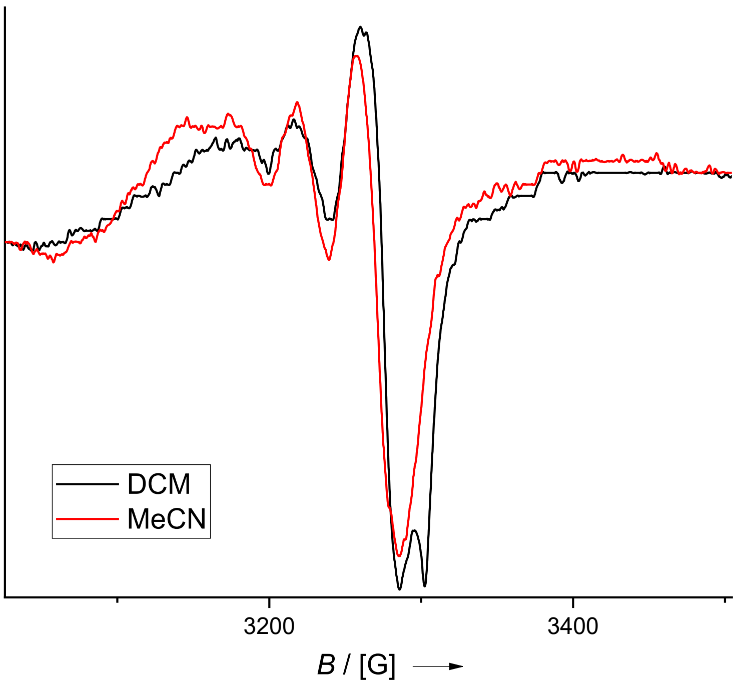

Figure 82: ESR of $[Cu(BF_4)_2(L5)_2]$ at r.t. (295 K) in DCM (9.446820 GHz, g=2.061) and MeCN (9.445350 GHz, g=2.063).

$[Co_2Br_4(L6_{Macro})]$

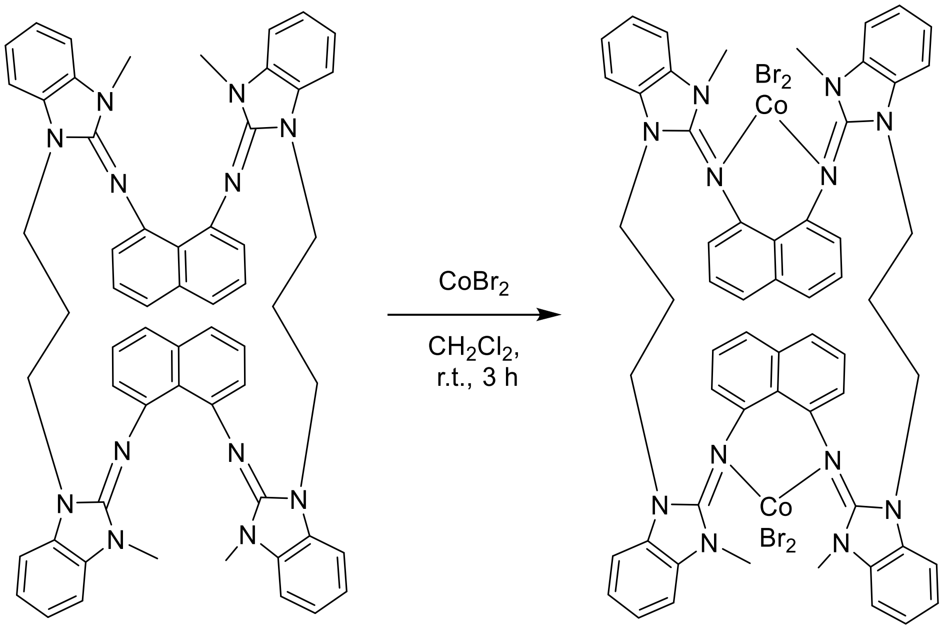

$L6_{Macro}$

$L6_{Macro}$ (20 mg, 43.6 µmol, 1.00 eq.) and $CoBr_2$ (11.4 mg, 52.3 µmol, 1.2 eq.) were suspended in DCM (5 mL). After 3 h the suspension was filtered, and the solvent removed *in vacuo*. After washing with ether (3x3 mL) the product was yielded as a green solid (15 mg, 30.3 µmol, 51%). Crystals for structural analysis were obtained through slow evaporation of the solvent out of a saturated DCM solution.

MS (ESI^+ in DCM): *m/z* = $[L5CoBr_2H^+]$ 1334.2227 (calc: 1334.2209).

EA (C, H, N in %) (*1.5 DCM*1.5 Et_2O) $C_{58}H_{52}Br_4Co_2N_{12}$: calc: C: 49.38, H: 4.43, N: 10.55
found: C: 50.00, H: 4.94, N: 9.59.

CV (DCM, $[nBu_4N][PF_6]$ 100 mV/s, vs. Fc/Fc^+): $E_{ox.}$= 0.24 V.

UV/Vis-Spectrum (DCM): λ(ε) = 206 (71194), 234 (30902, shoulder), 329 (10571), 353 (10764), 630 (1127), 688 (1215) nm ($M^{-1}cm^{-1}$).

Table 9: Selected bondlengths of $[Co_2Br_4(L6_{Macro})]$.

Bond	Distance [Å]	
Co-(N14,C2,C1,C3,N4) plane	0.621	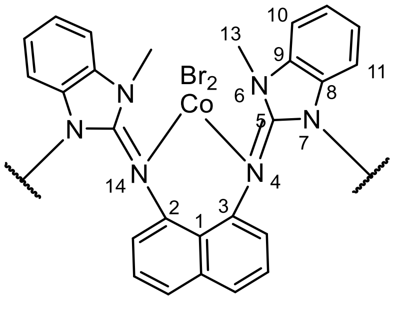
Co-Br	2.382(6)/2.372(6)	
C5-N4	1.342(3)	
C5-N7/C5-N6	1.359(3)/1.372(3)	
Co-N14/Co-N4	1.974(2)/1.972(2)	

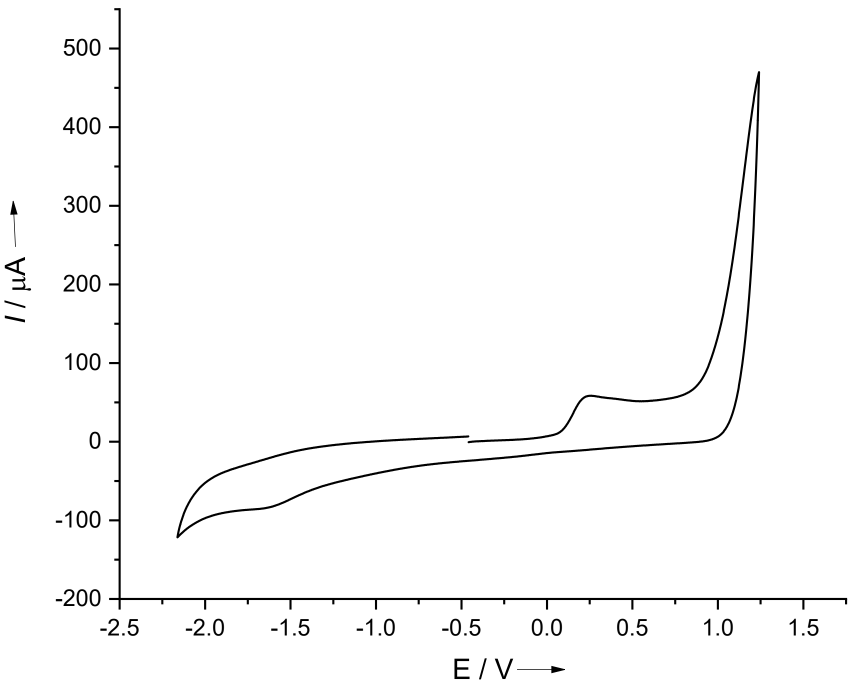

Figure 83: CV curve of [Co_2Br_4(L6$_{Macro}$)] (Fc/Fc^+ reference, 100 mV/s, $n$$Bu_4NPF_6$, Ag/AgCl electrode, DCM).

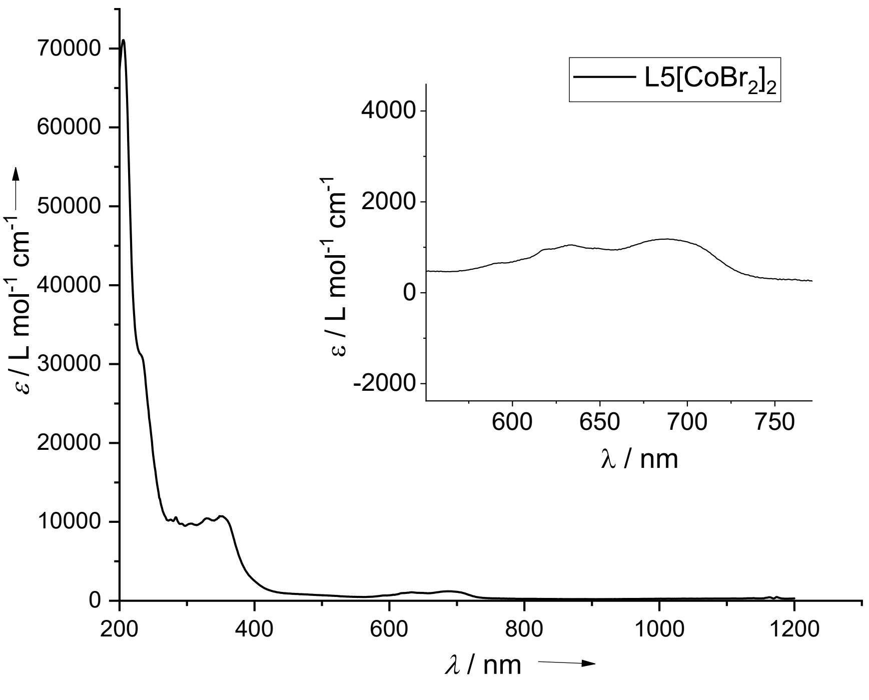

Figure 84: UV/Vis spectrum of [Co_2Br_4(L6$_{Macro}$)] in MeCN.

Calculated structures of [$CoBr_2$(L1, L2, L3, L5)] and cartesian coordinates [Å] after optimization

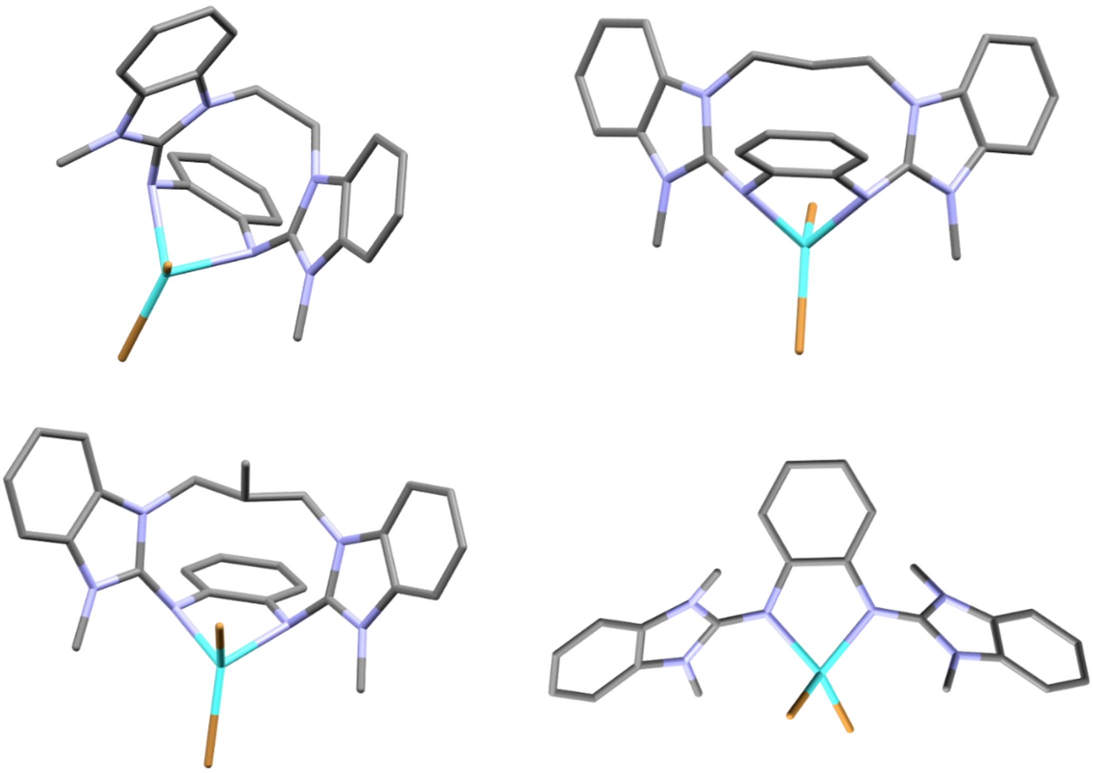

Bondlengths and distances of the calculated complexes.

Complex	Parameter	Distance [Å] or Angle [°]
[$CoBr_2$(L1)]	Co-N	2.123/2.158
	Co-Br	2.346/ 2.417
	d(N-C-C-N) plane-Co	1.072
	α	56.71
[$CoBr_2$(L2)]	Co-N	2.073/2.075
	Co-Br	2.359/ 2.423
	d(N-C-C-N) plane-Co	0.667
	α	85.34
[$CoBr_2$(L3)]	Co-N	2.075/2.076
	Co-Br	2.426/ 2.358
	d(N-C-C-N) plane-Co	0.663
	α	86.29

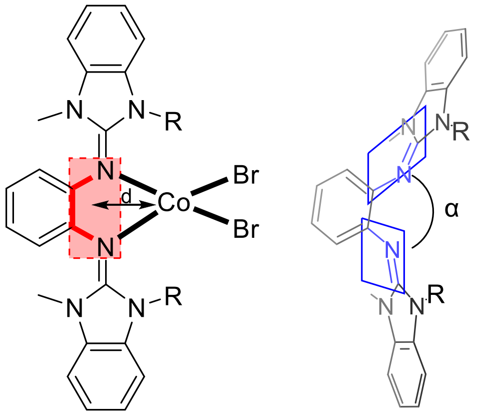

$[CoBr_2(L5)]$	Co-N	2.048/2.052
	Co-Br	2.399/2.397
	d(N-C-C-N) plane-Co	0.128
	α	149.04

$[CoBr_2(L1)]$

Br	6.52701570570903	0.34176335823088	20.68669323615265
Br	6.34781592730321	2.91974005061053	23.89672663393910
Co	6.51645372497372	2.58709511415111	21.58046953682657
N	7.87910221621130	3.78369606272748	20.41148089091030
N	9.04867590964719	2.09013310205594	19.28067832353501
N	7.77213952484427	3.36446264667182	17.99910883808104
N	5.15827478183761	3.77285745347906	20.45998846241005
N	3.80217632870552	2.07100087787130	19.59337620977829
N	4.85610121635881	3.29808787432866	18.08228984642930
C	7.20761378311655	5.03132118800265	20.36989239840096
C	5.80237932476636	5.02380716153446	20.35338619615158
C	5.10857162248990	6.23081666751109	20.33781202625356
H	4.02612753730777	6.21344750636369	20.34986893079663
C	5.80130686604991	7.43575297761032	20.32728703258041
H	5.25413014525656	8.36927647041745	20.31502821295987
C	7.19271812319528	7.44165933982582	20.36815863337691
H	7.73204058169318	8.37956576196870	20.38777773616863
C	7.89278540793276	6.24164997954974	20.40292950010779
H	8.97426915297573	6.22884218772463	20.44445706036034
C	8.20860182076190	3.16766939345953	19.30277343740356
C	9.07604569301614	1.53354659502608	18.01159856866013
C	9.71846132927794	0.41523260782249	17.51175393588268
H	10.31415276058670	-0.22361386315211	18.14809903328909
C	9.54987706529765	0.13331269318432	16.15627530964635
H	10.03274252559830	-0.73776176174883	15.73436783100009
C	8.76325497654600	0.94330820611597	15.33949283808265
H	8.64372635773398	0.69204560867558	14.29428567807177

C	8.11285417081273	2.06791777353984	15.84892432269974
H	7.49041246439074	2.68304400973585	15.21370793314854
C	8.28185684558399	2.34417147134435	17.19263654909325
C	9.78372042449275	1.59478317624990	20.42818033192308
H	9.75766219049263	2.35548722911948	21.20352996532774
H	10.81624206732960	1.40736330557879	20.13155111163750
H	9.32475318193531	0.68284595598980	20.80761382163902
C	4.63664687344892	3.14165424035928	19.44159652353861
C	3.56500357720142	1.47765136672022	18.36439650201874
C	2.84965355152539	0.34543306042167	18.01893363309489
H	2.37005791172088	-0.26681305059367	18.76931605740311
C	2.78973728988999	0.01560031139250	16.66582485188986
H	2.24395046505113	-0.86689857823007	16.36056324673153
C	3.42932232645822	0.79409472336155	15.70325719394632
H	3.37295877793232	0.50789445443001	14.66173942999099
C	4.15464212901067	1.93243773735426	16.05717068459613
H	4.66136649477134	2.51798955351787	15.30283746851097
C	4.21410696180207	2.25758804602332	17.39976884203679
C	3.27235703731915	1.60650622905325	20.86198162712917
H	3.76694812731614	0.68389402278123	21.16197031604912
H	2.19832198403289	1.44760420726183	20.75924615850991
H	3.45969253331434	2.36786868068618	21.61425551320523
C	7.05253581903271	4.50228869478600	17.46529585940719
C	5.49785951814146	4.40982189989511	17.40976299668911
H	7.42172787381988	4.65134982739032	16.45034975067954
H	7.32798757931758	5.38164561444979	18.03359035908779
H	5.09542155960570	5.34160020933448	17.80036800113080
H	5.18578985505600	4.34040056802814	16.37027461162756

[$CoBr_2$(L2)]

Br	13.81975255671866	1.29629932832410	21.36615652825267
Br	14.22991500866383	2.88924806976612	25.17771715332181
Co	14.09319633340034	0.98236983697494	23.68858726080715

N	12.86239204675247	-0.40366623410057	24.61714840596461
N	11.10493566390652	0.94044650582415	25.42096573765361
N	12.10165590505664	-0.34753380151004	26.92489377388445
N	15.53879851920711	-0.36375875979031	24.32507514232508
N	17.36999410479511	1.05074649706336	24.75099129514773
N	16.75046291028475	-0.25627923141679	26.43062631249469
C	13.53100752262182	-1.63938827124913	24.65343082795239
C	14.93011044593126	-1.61804944053679	24.50190100054629
C	15.63804575808611	-2.81849720949468	24.48193004743345
H	16.71207086466687	-2.79111888439226	24.34688394770090
C	14.97423589664935	-4.02928601808846	24.63389119877152
H	15.53407288762805	-4.95510537348306	24.61576745702747
C	13.59048057441634	-4.05052909558878	24.78439670499790
H	13.06834683948346	-4.99298698256238	24.88448315547579
C	12.87260754349773	-2.86110177233896	24.78193660537672
H	11.79431941523053	-2.86800611063225	24.88109660671577
C	12.07449095715869	-0.00537595866515	25.58684121771559
C	10.54677665882156	1.25765092752714	26.65109913290307
C	9.56708844499610	2.16851385998996	27.00529355607892
H	9.09976524955939	2.80877642750865	26.27045390118012
C	9.22035352224153	2.24198102959570	28.35321142527546
H	8.46111641844371	2.94602135917121	28.66588772210435
C	9.84001595744212	1.43327915146139	29.30494083905022
H	9.55485139482503	1.51966665017444	30.34475598219817
C	10.82689757518797	0.51624924796169	28.94398290381660
H	11.30655241515043	-0.10252012861083	29.68969760302620
C	11.16831588030648	0.44112122714785	27.60545035117724
C	10.77620819801113	1.57284817791520	24.15717724679036
H	11.25630415973817	2.54958712254830	24.08807210257856
H	9.69457682936405	1.68034120222721	24.08230780135327
H	11.13718857968073	0.94716130678848	23.34508528244483
C	16.49130228179310	0.07184017057166	25.11420259794384
C	18.15028465514767	1.40610158762418	25.84175995481699

C	19.14075439627062	2.36145558067306	25.98765849146890
H	19.42197422662237	3.01205086163827	25.17156165563417
C	19.74821193892164	2.46597394436209	27.23776665172778
H	20.52309581652437	3.20538044801282	27.38862364830100
C	19.36840419764178	1.64382324374222	28.29768806877656
H	19.85260770012220	1.75491296445968	29.25851003233903
C	18.36873368728252	0.68290874764876	28.14706221585028
H	18.07549756896784	0.05499866754271	28.97692682093356
C	17.76832611914816	0.57712843943648	26.90525415164962
C	17.41009038743735	1.67816965184844	23.44356882754711
H	16.92080055003030	1.02592754824342	22.72485930212660
H	18.44892719136802	1.82850225913530	23.15156637781045
H	16.88452442370210	2.63353685565869	23.46624299703885
C	13.23732052475167	-1.00024875154265	27.57566451671767
H	13.40219818298656	-1.96315819603868	27.09931154176083
H	12.93380928394789	-1.20181870280622	28.60074684232820
C	14.51217852826984	-0.13859408138339	27.51511623750301
H	14.41244244664515	0.56692074726539	26.69236236955175
H	14.59804803687640	0.47633759276552	28.41137412697915
C	15.80186697331501	-0.95246364504305	27.29933258499021
H	16.32085997366070	-1.14620745392381	28.23576997811971
H	15.57675787064209	-1.91944313540032	26.85690377854074

[CoBr$_2$(L3)]

Br	13.83145145277044	1.30396651016384	21.36230255427002
Br	14.21226747287230	2.88617641035434	25.18395085621098
Co	14.09224083426612	0.98253546026237	23.68411929873261
N	12.85851111462303	-0.41199632291024	24.60197063813029
N	11.08296770324100	0.91105050216823	25.40189092222723
N	12.09668319832000	-0.35920332444583	26.91048138152545
N	15.54064516789748	-0.35956263188181	24.32262909722752
N	17.39489561492226	1.02790815415584	24.74841416814865
N	16.76512825191467	-0.28421022933578	26.42145787208240

C	13.53491271361849	-1.64371744743600	24.64168919413538
C	14.93418685129857	-1.61636592437692	24.49473466628203
C	15.64768280390888	-2.81351134125759	24.47353840634095
H	16.72180689145738	-2.78112960368643	24.34071167159325
C	14.98906676403784	-4.02731917724087	24.62300307163788
H	15.55322944268065	-4.95051562484572	24.60413978395905
C	13.60544149966999	-4.05473116982936	24.77254104203834
H	13.08704019361246	-4.99935078865535	24.87182931415140
C	12.88218737429318	-2.86855780745086	24.77000872946644
H	11.80380516383075	-2.88058120370278	24.86744159466255
C	12.06527415913309	-0.02086462916628	25.57100389442395
C	10.51278264789530	1.21624676341014	26.62932477308192
C	9.51628408612236	2.11085402994837	26.97878730787529
H	9.04398682913519	2.74509178955366	26.24187191203812
C	9.15791319041305	2.17446156262491	28.32392598335334
H	8.38530434227020	2.86541902551274	28.63303241065728
C	9.78257165663115	1.37148751015686	29.27761055412127
H	9.48727564004662	1.44900001037887	30.31530062958613
C	10.78685053462022	0.47193391440894	28.92171563453148
H	11.27156048980606	-0.14062358247167	29.66895776309191
C	11.14193399447604	0.40781583780575	27.58591905404073
C	10.75526404290542	1.54392394333677	24.13802646423880
H	11.21900414841707	2.52921117965048	24.07863222119951
H	9.67271539074483	1.63282892807807	24.05235311385111
H	11.13530414631403	0.92853859084762	23.32672655765212
C	16.50412333158468	0.05922088030893	25.10856396210088
C	18.19789098190081	1.35052066260448	25.83328175229427
C	19.21281863153803	2.28057129170833	25.97792776484120
H	19.49742945512659	2.93598895397116	25.16685795481450
C	19.84194089735493	2.35184683395362	27.21949461910953
H	20.63669323891548	3.07012867014097	27.36897948097981
C	19.45967385211277	1.52189060818758	28.27282104864195
H	19.96195138159926	1.60602577261063	29.22710236637425

C	18.43542942117250	0.58757568395364	28.12397971339223
H	18.13912819332022	-0.04507688673439	28.94886309474862
C	17.81186929700779	0.51549354270047	26.89081791099976
C	17.43107206370516	1.66982709425856	23.44773711643431
H	16.92937542886338	1.03057724640610	22.72574564011730
H	18.46895529921112	1.81304985929152	23.14907306286112
H	16.91529668020873	2.63009301447158	23.48514713744915
C	13.24765999771133	-0.99209901852733	27.55918439895294
H	13.40499173168329	-1.96883962070938	27.10943674161041
H	12.97189840241272	-1.15572240234860	28.60001607114632
C	14.50998148416868	-0.11630996573326	27.44222088518700
C	15.79421600145529	-0.95433778687471	27.28989108256561
H	15.57727589678688	-1.93496228076579	26.87442115602088
H	16.28460182735373	-1.10991715600299	28.24981460804675
C	14.61448166339841	0.91197993918967	28.56422473707994
H	14.40440828582702	0.44593532780523	26.51749763864834
H	14.72199743327366	0.42456223440044	29.53795379007537
H	13.72791031093841	1.54599330168470	28.59016741556096
H	15.47224300320113	1.56630488592449	28.40810631337746

[$CoBr_2$(L5)]

Br	3.12159980822052	2.55240918174354	13.86881482597361
Br	-0.78061893905011	2.77969719145770	12.48946577347426
Co	1.28597884212373	3.85978239506721	13.04512953443940
N	1.54001279532614	5.26927222790192	11.58166399495676
N	1.27296636972202	3.97841779388534	9.62356073332658
N	-0.07260689398278	5.71923166090979	9.82593822331448
N	1.33607725822623	5.52431386087633	14.24345816265398
N	1.06442341323141	4.58662253327756	16.39053003031487
N	2.95143734668087	5.68312307932985	16.04828814345835
C	1.69009007328360	6.55899275757710	12.11154796369049
C	1.63497024431615	6.68367921321584	13.51853711450487
C	1.79036726775682	7.94620919343807	14.09358634985503

H	1.69631060082609	8.04989884541228	15.16731718104502
C	2.04517215155286	9.06374368631007	13.30797762456334
H	2.16234363389262	10.03274318690240	13.77517329711727
C	2.14700244400147	8.93201120480936	11.92809082325011
H	2.36588761221617	9.79306053634646	11.31045275320480
C	1.97054877723857	7.68593753746549	11.33847947080976
H	2.06318261356141	7.57602651340969	10.26504030356167
C	0.96307479434634	5.04195888571385	10.42115440956825
C	0.41747753388978	3.95093840163460	8.53177560778285
C	0.30835655216471	3.06996794379134	7.46979751335914
H	0.94283482609218	2.19850740209991	7.39074292632340
C	-0.66651710639899	3.34316125684494	6.51199551607305
H	-0.78111682561650	2.67491717153727	5.66933583970675
C	-1.50006985359130	4.45625906563330	6.62232456227119
H	-2.24904034561669	4.63802866117844	5.86348230879516
C	-1.39037426559788	5.34033317377276	7.69469731047056
H	-2.03767748073352	6.20241889859703	7.77561134427030
C	-0.42228153861757	5.06715509847018	8.64528079156001
C	2.27867345679828	2.97956673282363	9.93002445407065
H	1.81288222576234	2.09649508772450	10.36926239335470
H	2.98415477841935	3.39377232368033	10.64499378905220
H	2.80269402186248	2.70778631366925	9.01453898164225
C	-0.91272111399255	6.69497766793099	10.49826296195112
H	-0.57385671352319	7.71445371602936	10.31574056938983
H	-0.89795432704928	6.50198782080444	11.56782049241720
H	-1.93113751119596	6.57263721775968	10.13613645569508
C	1.76061982948726	5.32003296828018	15.47408752697794
C	1.81818184603479	4.45366873554300	17.54751138064987
C	1.56693761367833	3.79475649053231	18.73862334221560
H	0.65629212921849	3.23317306981614	18.89235104006418
C	2.54486253135690	3.87306356149958	19.72800318351474
H	2.38322565555378	3.36924219435485	20.67129363048503
C	3.72905219478216	4.58187516477032	19.52305368487060

H	4.46986446772890	4.61860180536205	20.31023762517658
C	3.98028062480777	5.24250113487547	18.32181793263609
H	4.89893325806915	5.79104723963491	18.16754734718176
C	3.00774112264684	5.16491109151358	17.34024299147418
C	-0.23426288497702	3.98535840869599	16.15472898941390
H	-0.12917931928196	2.92412810779527	15.92756007831022
H	-0.69721613102202	4.47360583801145	15.30249523891330
H	-0.85613453384242	4.11611081508705	17.03978410013942
C	4.10586549787163	6.16088840715640	15.30597484872080
H	4.04603549967108	5.79368855722631	14.28486407163697
H	5.00023360243756	5.74857370708159	15.76726722598313
H	4.15737946923286	7.24926126373145	15.29832523036664

Calculation of *ΔG* and *ΔH* for Ligand exchange in the complexes

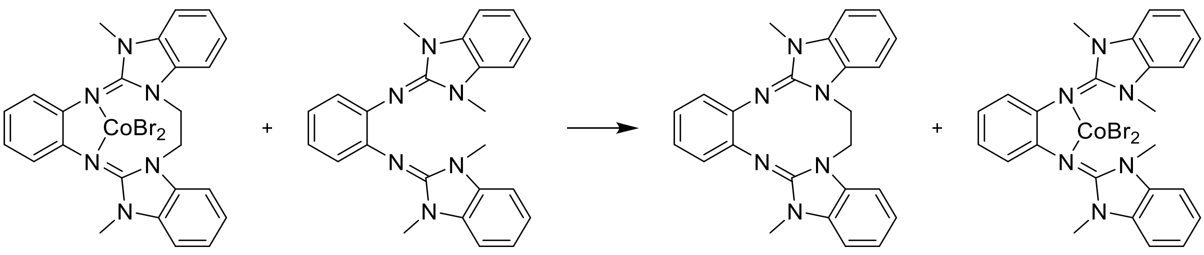

Figure 85: Exemplary calculated reaction in the ligand exchange for L1 and L5.

Table 10: Thermodynamic data of the reaction after figure 85.

Ligands	*ΔH* [kJ/mol]	*ΔG* [kJ/mol]
L1/L5	-46,644	-51,038
L2/L5	-3,631	-8,347
L3/L5	3,478	-2,678

5. Crystal data

L1

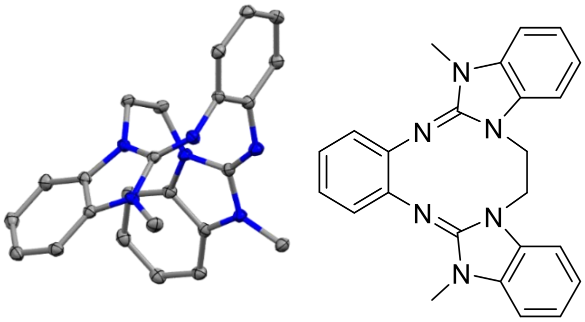

Table 11: Crystal and structural data of **L1**.

Identification code	mo_2023_eehk10_8_0m
Deposition number	2477545
Empirical formula	$C_{24}H_{22}N_6$
Formula weight	394.47
Temperature/K	100.00
Crystal system	monoclinic
Space group	$P2_1/c$
a/Å	9.090(2)
b/Å	13.047(3)
c/Å	15.826(3)
a/°	90
b/°	93.343(8)
g/°	90
Volume/Å^3	1873.8(7)
Z	4
r_{calc}g/cm^3	1.398
m/mm^{-1}	0.087
F(000)	832.0
Crystal size/mm^3	0.305 × 0.297 × 0.122
Radiation	MoKα (*l* = 0.71073)
2*Θ* range for data collection/°	4.048 to 54.988
Index ranges	-11 ≤ h ≤ 11, -16 ≤ k ≤ 16, -20 ≤ l ≤ 20
Reflections collected	123571
Independent reflections	4306 [R_{int} = 0.0751, R_{sigma} = 0.0368]
Data/restraints/parameters	4306/0/273
Goodness-of-fit on F^2	1.031
Final *R* indexes [I≥2*s* (I)]	R_1 = 0.0425, wR_2 = 0.1106
Final *R* indexes [all data]	R_1 = 0.0449, wR_2 = 0.1146
Largest diff. peak/hole / e Å^{-3}	0.27/-0.31

(L1+H)OTf

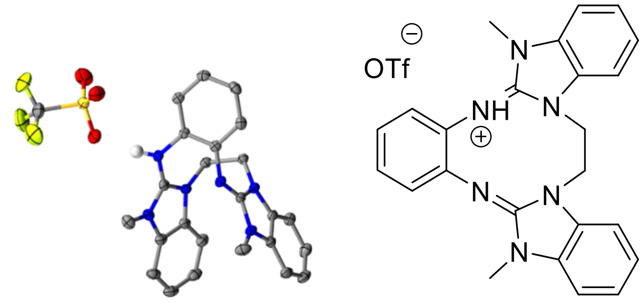

Table 12: Crystal and structural data of **(L1+H)OTf**.

Identification code	mo_2023_ee100_0a
Deposition number	2477536
Empirical formula	$C_{25}H_{23}F_3N_6O_3S$
Formula weight	544.55
Temperature/K	120.00
Crystal system	orthorhombic
Space group	$Pna2_1$
a/Å	10.4925(6)
b/Å	17.1109(9)
c/Å	13.9826(8)
a/°	90
b/°	90
g/°	90
Volume/Å^3	2510.4(2)
Z	4
r_{calc}g/cm^3	1.441
m/mm^{-1}	0.192
F(000)	1128.0
Crystal size/mm^3	0.24 × 0.23 × 0.18
Radiation	MoKα (λ = 0.71073)
2Θ range for data collection/°	3.762 to 56.116
Index ranges	$-13 \le h \le 13$, $-22 \le k \le 22$, $-18 \le l \le 18$
Reflections collected	106023
Independent reflections	6056 [R_{int} = 0.0842, R_{sigma} = 0.0412]
Data/restraints/parameters	6056/1/349
Goodness-of-fit on F^2	1.022
Final R indexes [I≥2s (I)]	R_1 = 0.0319, wR_2 = 0.0809
Final R indexes [all data]	R_1 = 0.0378, wR_2 = 0.0833
Largest diff. peak/hole / e Å^{-3}	0.24/-0.30

(L1+H)PF_6

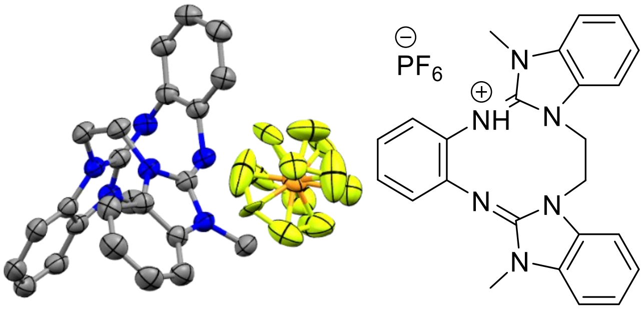

Table 13: Crystal and structural data of **(L1+H)PF_6.**

Identification code	mo_2023_eehk26_0ma
Deposition number	2477548
Empirical formula	$C_{24}H_{23}F_6N_6P$
Formula weight	540.45
Temperature/K	100.00
Crystal system	monoclinic
Space group	$P2_1/n$
a/Å	10.959(4)
b/Å	12.521(5)
c/Å	17.321(7)
a/°	90
b/°	102.229(15)
g/°	90
Volume/Å^3	2322.9(16)
Z	4
r_{calc}g/cm^3	1.545
m/mm^{-1}	0.194
F(000)	1112.0
Crystal size/mm^3	0.178 × 0.152 × 0.054
Radiation	MoKα (l = 0.71073)
2Θ range for data collection/°	4.046 to 52.946
Index ranges	$-13 \leq h \leq 13$, $-15 \leq k \leq 15$, $-21 \leq l \leq 21$
Reflections collected	58957
Independent reflections	4766 [R_{int} = 0.1109, R_{sigma} = 0.0604]
Data/restraints/parameters	4766/151/395
Goodness-of-fit on F^2	1.028
Final R indexes [I≥2s (I)]	R_1 = 0.0629, wR_2 = 0.1550
Final R indexes [all data]	R_1 = 0.1057, wR_2 = 0.1903
Largest diff. peak/hole / e Å^{-3}	0.54/-0.45

(L1+2H)Cl_2

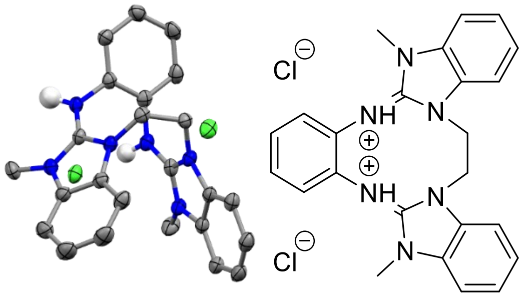

Table 14: Crystal and structural data of **(L1+2H)Cl_2**.

Identification code	mo_2023_eehk10_10b_0ma
Deposition number	2477546
Empirical formula	$C_{26}H_{30}Cl_2N_6$
Formula weight	497.46
Temperature/K	100.00
Crystal system	monoclinic
Space group	$P2_1/c$
a/Å	11.618(5)
b/Å	14.715(6)
c/Å	15.198(7)
a/°	90
b/°	111.498(15)
g/°	90
Volume/Å^3	2417.5(18)
Z	4
r_{calc}g/cm^3	1.367
m/mm^{-1}	0.296
F(000)	1048.0
Crystal size/mm^3	0.167 × 0.143 × 0.075
Radiation	MoKα (l = 0.71073)
2Θ range for data collection/°	3.768 to 52
Index ranges	-14 ≤ h ≤ 14, -18 ≤ k ≤ 15, -18 ≤ l ≤ 18
Reflections collected	79768
Independent reflections	4735 [R_{int} = 0.2032, R_{sigma} = 0.0914]
Data/restraints/parameters	4735/0/320
Goodness-of-fit on F^2	1.031
Final R indexes [I≥2s (I)]	R_1 = 0.0650, wR_2 = 0.1693
Final R indexes [all data]	R_1 = 0.0811, wR_2 = 0.1868
Largest diff. peak/hole / e Å^{-3}	0.79/-0.61

L2

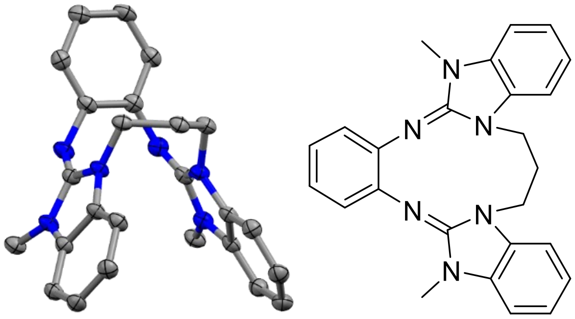

Table 15: Crystal and structural data of **L2**.

Identification code	mo_2025_ee230_4_0m
Deposition number	2477544
Empirical formula	$C_{25}H_{24}N_6$
Formula weight	408.50
Temperature/K	100.00
Crystal system	triclinic
Space group	P-1
a/Å	8.9238(13)
b/Å	9.3438(13)
c/Å	12.7315(19)
α/°	94.275(5)
β/°	108.202(5)
γ/°	94.576(5)
Volume/Å^3	999.7(3)
Z	2
ρ_{calc}g/cm^3	1.357
μ/mm^{-1}	0.084
F(000)	432.0
Crystal size/mm^3	0.168 × 0.163 × 0.059
Radiation	MoKα (λ = 0.71073)
2Θ range for data collection/°	4.398 to 52.94
Index ranges	-11 ≤ h ≤ 11, -11 ≤ k ≤ 11, -15 ≤ l ≤ 15
Reflections collected	51200
Independent reflections	4103 [R_{int} = 0.1140, R_{sigma} = 0.0498]
Data/restraints/parameters	4103/0/282
Goodness-of-fit on F^2	1.099
Final R indexes [I>=2σ (I)]	R_1 = 0.0601, wR_2 = 0.1545
Final R indexes [all data]	R_1 = 0.0839, wR_2 = 0.1756
Largest diff. peak/hole / e Å^{-3}	0.33/-0.41

(L2+H)BF$_4$

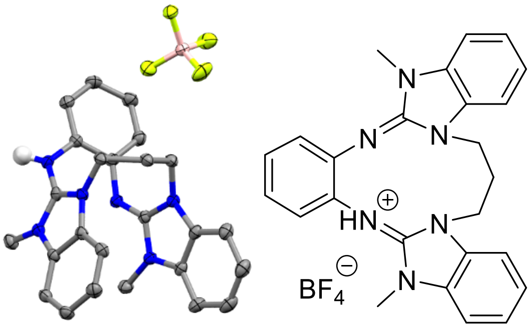

Table 16: Crystal and structural data of (**L2+H)BF$_4$**.

Identification code	mo_2024_ee120_2_0m
Deposition number	2477537
Empirical formula	$C_{26}H_{27}BCl_2F_4N_6$
Formula weight	581.24
Temperature/K	100
Crystal system	triclinic
Space group	P-1
a/Å	8.7680(4)
b/Å	11.8733(5)
c/Å	14.3746(7)
a/°	104.711(2)
b/°	99.215(2)
g/°	106.342(2)
Volume/Å^3	1345.00(11)
Z	2
r_{calc}g/cm^3	1.435
m/mm^{-1}	0.298
F(000)	600
Crystal size/mm^3	0.15 × 0.07 × 0.06
Radiation	MoKα (λ = 0.71073)
2Θ range for data collection/°	3.768 to 53.998
Index ranges	-11 ≤ h ≤ 11, -15 ≤ k ≤ 15, -18 ≤ l ≤ 18
Reflections collected	82917
Independent reflections	5877 [R_{int} = 0.0682, R_{sigma} = 0.0254]
Data/restraints/parameters	5877/16/349
Goodness-of-fit on F^2	1.052
Final R indexes [I≥2s (I)]	R_1 = 0.0397, wR_2 = 0.0990
Final R indexes [all data]	R_1 = 0.0507, wR_2 = 0.1059
Largest diff. peak/hole / e Å^{-3}	0.28/-0.29

(L2+2H)Cl$_2$

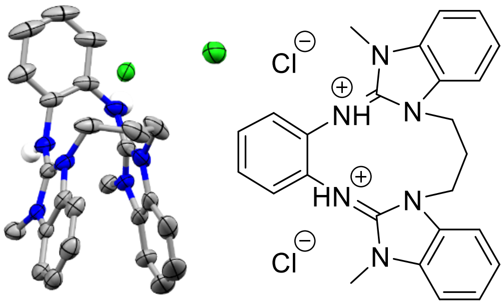

Table 17: Crystal and structural data of **(L2+2H)Cl$_2$**.

Identification code	mo_2023_eehk20_7_1ma
Deposition number	2477547
Empirical formula	$C_{25}H_{26}Cl_2N_6$
Formula weight	481.42
Temperature/K	100.00
Crystal system	monoclinic
Space group	$P2_1/c$
a/Å	15.6417(15)
b/Å	8.6278(11)
c/Å	22.278(3)
a/°	90
b/°	98.004(5)
g/°	90
Volume/Å^3	2977.2(6)
Z	4
r_{calc}g/cm^3	1.074
m/mm^{-1}	0.239
F(000)	1008.0
Crystal size/mm^3	0.253 × 0.155 × 0.153
Radiation	MoKα (/ = 0.71073)
2Θ range for data collection/°	4.224 to 52.996
Index ranges	$-18 \leq h \leq 19$, $-10 \leq k \leq 10$, $-27 \leq l \leq 27$
Reflections collected	58666
Independent reflections	6167 [R_{int} = 0.0972, R_{sigma} = 0.0514]
Data/restraints/parameters	6167/38/317
Goodness-of-fit on F^2	1.044
Final R indexes [I≥2s (I)]	R_1 = 0.0664, wR_2 = 0.1733
Final R indexes [all data]	R_1 = 0.0819, wR_2 = 0.1879
Largest diff. peak/hole / e Å^{-3}	0.75/-0.58

(L3+2H)Cl$_2$

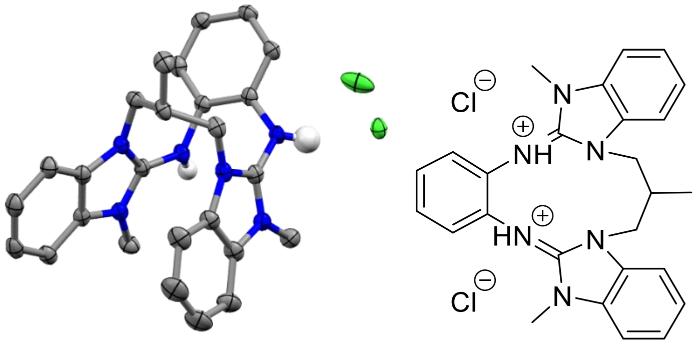

Table 18: Crystal and structural data of **(L3+2H)Cl$_2$**.

Identification code	mo_2024_eeml42_0
Deposition number	2477549
Empirical formula	$C_{26}H_{28}Cl_2N6\cdot1.2\ CH_3CN$
Formula weight	544.71
Temperature/K	100.00
Crystal system	monoclinic
Space group	$P2_1/c$
a/Å	15.5775(12)
b/Å	8.6923(7)
c/Å	20.4737(17)
α/°	90
β/°	91.013(3)
γ/°	90
Volume/Å^3	2771.8(4)
Z	4
ρ_{calc}g/cm^3	1.305
μ/mm^{-1}	0.266
F(000)	1146.0
Crystal size/mm^3	0.149 × 0.103 × 0.1
Radiation	MoKα (λ = 0.71073)
2Θ range for data collection/°	3.98 to 52.998
Index ranges	-19 ≤ h ≤ 19, -10 ≤ k ≤ 10, -25 ≤ l ≤ 25
Reflections collected	65693
Independent reflections	5739 [R_{int} = 0.1193, R_{sigma} = 0.0512]
Data/restraints/parameters	5739/2/328
Goodness-of-fit on F^2	1.028
Final R indexes [I>=2σ (I)]	R_1 = 0.0485, wR_2 = 0.1137
Final R indexes [all data]	R_1 = 0.0742, wR_2 = 0.1314
Largest diff. peak/hole / e Å^{-3}	0.33/-0.37

L4

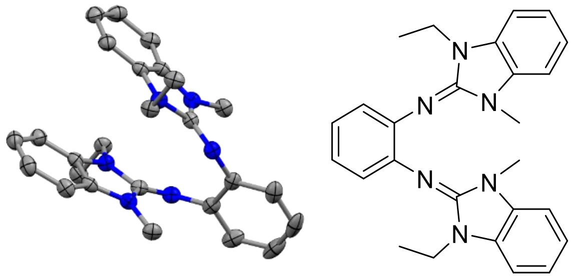

Table 19: Crystal and structural data of **L4**.

Identification code	mo_2023_ee75_0ma
Deposition number	2477529
Empirical formula	$C_{26}H_{28}N_6$
Formula weight	424.54
Temperature/K	100.00
Crystal system	monoclinic
Space group	P2/c
a/Å	16.115(3)
b/Å	11.967(2)
c/Å	12.403(3)
α/°	90
β/°	112.326(7)
γ/°	90
Volume/Å^3	2212.7(8)
Z	4
ρ_{calc}g/cm^3	1.274
μ/mm^{-1}	0.078
F(000)	904.0
Crystal size/mm^3	0.394 × 0.176 × 0.104
Radiation	MoKα (λ = 0.71073)
2Θ range for data collection/°	2.732 to 51.998
Index ranges	-19 ≤ h ≤ 19, -14 ≤ k ≤ 14, -15 ≤ l ≤ 15
Reflections collected	53244
Independent reflections	4343 [R_{int} = 0.0916, R_{sigma} = 0.0399]
Data/restraints/parameters	4343/0/294
Goodness-of-fit on F^2	1.165
Final R indexes [I>=2σ (I)]	R_1 = 0.0899, wR_2 = 0.2273
Final R indexes [all data]	R_1 = 0.0939, wR_2 = 0.2292
Largest diff. peak/hole / e Å^{-3}	1.11/-0.38

(L4H)PF$_6$

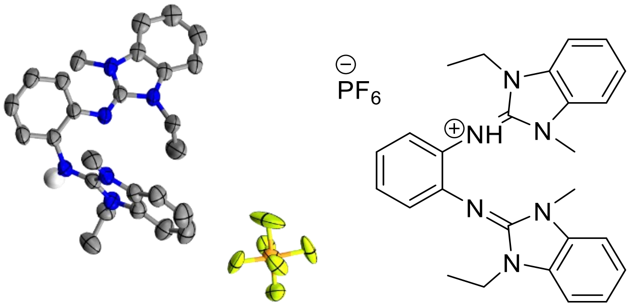

Table 20: Crystal and structural data of (**L4+H)PF$_6$**.

Identification code	mo_2023_ee81_2_0ma
Deposition number	2477531
Empirical formula	$C_{26}H_{29}F_6N_6P \cdot 0.2\ H_2O$
Formula weight	574.12
Temperature/K	120.0
Crystal system	monoclinic
Space group	$P2_1/c$
a/Å	24.113(3)
b/Å	13.1508(14)
c/Å	17.202(2)
α/°	90
β/°	99.827(4)
γ/°	90
Volume/Å^3	5374.9(10)
Z	8
ρ_{calc}g/cm^3	1.419
μ/mm^{-1}	0.173
F(000)	2384.0
Crystal size/mm^3	0.23 × 0.15 × 0.133
Radiation	MoKα (λ = 0.71073)
2Θ range for data collection/°	3.92 to 53
Index ranges	-30 ≤ h ≤ 30, -16 ≤ k ≤ 16, -21 ≤ l ≤ 21
Reflections collected	79805
Independent reflections	11142 [R_{int} = 0.0933, R_{sigma} = 0.0589]
Data/restraints/parameters	11142/560/900
Goodness-of-fit on F^2	1.053
Final R indexes [I>=2σ (I)]	R_1 = 0.0916, wR_2 = 0.2129
Final R indexes [all data]	R_1 = 0.1152, wR_2 = 0.2313
Largest diff. peak/hole / e Å^{-3}	0.73/-0.46

(L4+2H)Cl₂

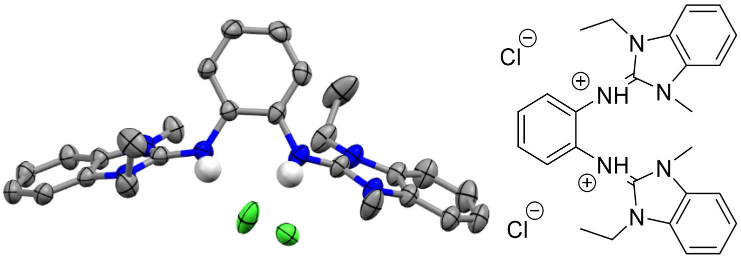

Table 21: Crystal and structural data of (**L4+2H)Cl₂**.

Identification code	mo_2023_ee79_01a
Deposition number	2477530
Empirical formula	$C_{26}H_{38}Cl_2N_6O_4$
Formula weight	497.46
Temperature/K	100.00
Crystal system	monoclinic
Space group	C2/c
a/Å	32.120(3)
b/Å	12.7754(9)
c/Å	16.9043(11)
α/°	90
β/°	121.726(4)
γ/°	90
Volume/Å^3	5900.0(8)
Z	8
ρ_{calc}g/cm^3	1.120
μ/mm^{-1}	0.243
F(000)	2096.0
Crystal size/mm^3	0.51 × 0.32 × 0.27
Radiation	MoKα (λ = 0.71073)
2Θ range for data collection/°	4.82 to 52.998
Index ranges	-40 ≤ h ≤ 40, -16 ≤ k ≤ 16, -21 ≤ l ≤ 21
Reflections collected	131376
Independent reflections	6122 [R_{int} = 0.0963, R_{sigma} = 0.0348]
Data/restraints/parameters	6122/26/341
Goodness-of-fit on F^2	1.041
Final R indexes [I>=2σ (I)]	R_1 = 0.0610, wR_2 = 0.1737
Final R indexes [all data]	R_1 = 0.0686, wR_2 = 0.1824
Largest diff. peak/hole / e Å^{-3}	1.02/-0.66

o-Diguanidinophenazine

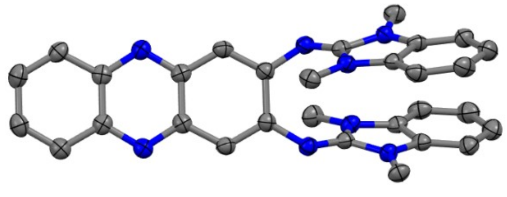

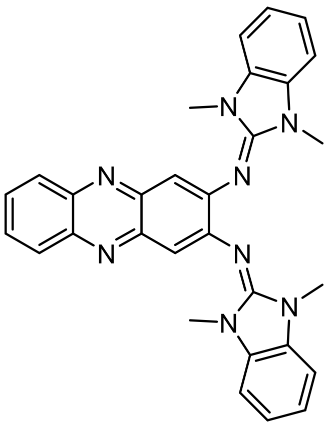

Table 22: Crystal and structural data of *o*-diguanidinophenazine.

Identification code	mo_2024_ee183_0m
Deposition number	2477541
Empirical formula	$C_{30}H_{28}N_8O$
Formula weight	516.60
Temperature/K	100.00
Crystal system	monoclinic
Space group	$P2_1/n$
a/Å	11.7598(11)
b/Å	13.0255(14)
c/Å	16.7382(18)
α/°	90
β/°	93.536(4)
γ/°	90
Volume/Å^3	2559.0(5)
Z	4
ρ_{calc}g/cm^3	1.341
μ/mm^{-1}	0.086
F(000)	1088.0
Crystal size/mm^3	0.17 × 0.13 × 0.06
Radiation	MoKα (λ = 0.71073)
2Θ range for data collection/°	3.966 to 51.996
Index ranges	-14 ≤ h ≤ 14, -16 ≤ k ≤ 16, -20 ≤ l ≤ 20
Reflections collected	48796
Independent reflections	5036 [R_{int} = 0.1244, R_{sigma} = 0.0620]
Data/restraints/parameters	5036/0/359
Goodness-of-fit on F^2	1.020
Final R indexes [I>=2σ (I)]	R_1 = 0.0478, wR_2 = 0.1106
Final R indexes [all data]	R_1 = 0.0919, wR_2 = 0.1379
Largest diff. peak/hole / e Å^{-3}	0.18/-0.30

[$CoBr_2$(L2)]

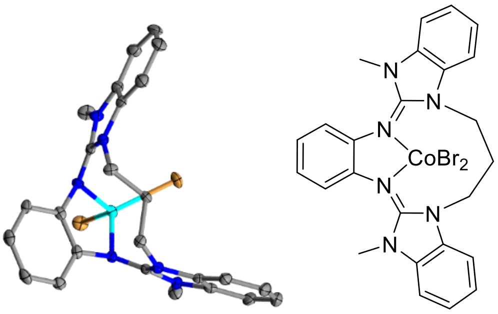

Table 23: Crystal and structural data of **[$CoBr_2$(L2)]**.

Identification code	mo_2023_ee90_2_0ma
Deposition number	2477533
Empirical formula	$C_{25}H_{24}Br_2CoN_6 \cdot 1.5\ CH_2Cl_2$
Formula weight	754.64
Temperature/K	120.00
Crystal system	monoclinic
Space group	$P2_1/c$
a/Å	24.5373(13)
b/Å	10.6239(7)
c/Å	23.6441(15)
α/°	90
β/°	109.682(2)
γ/°	90
Volume/Å^3	5803.5(6)
Z	8
ρ_{calc}g/cm^3	1.727
μ/mm^{-1}	3.651
F(000)	3008.0
Crystal size/mm^3	0.24 × 0.169 × 0.126
Radiation	MoKα (λ = 0.71073)
2Θ range for data collection/°	3.486 to 54
Index ranges	-31 ≤ h ≤ 31, -13 ≤ k ≤ 13, -30 ≤ l ≤ 30
Reflections collected	129509
Independent reflections	12667 [R_{int} = 0.0968, R_{sigma} = 0.0512]
Data/restraints/parameters	12667/18/716
Goodness-of-fit on F^2	1.170
Final R indexes [I>=2σ (I)]	R_1 = 0.0507, wR_2 = 0.1159
Final R indexes [all data]	R_1 = 0.0639, wR_2 = 0.1216
Largest diff. peak/hole / e Å^{-3}	1.63/-0.97

[$NiCl_2$(L2)]

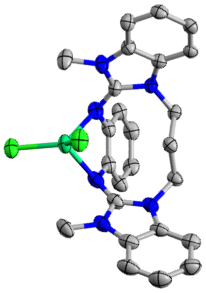

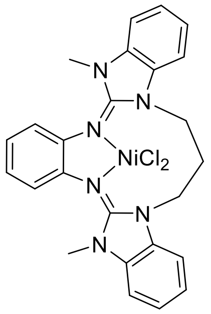

Table 24: Crystal and structural data of **[$NiCl_2$(L2)]**.

Identification code	mo_2025_ee202_0m
Deposition number	2477542
Empirical formula	$C_{25}H_{24}Cl_2N_6Ni$·0.5 CH_2Cl_2, 0.45 $C_4H_{10}O$
Formula weight	635.16
Temperature/K	100.00
Crystal system	monoclinic
Space group	$P2_1/n$
a/Å	10.3851(8)
b/Å	20.8242(17)
c/Å	26.228(2)
α/°	90
β/°	96.594(3)
γ/°	90
Volume/Å^3	5634.5(8)
Z	8
ρ_{calc}g/cm^3	1.497
μ/mm^{-1}	1.052
F(000)	2627.0
Crystal size/mm^3	0.18 × 0.14 × 0.08
Radiation	MoKα (λ = 0.71073)
2Θ range for data collection/°	3.912 to 52
Index ranges	-12 ≤ h ≤ 12, -25 ≤ k ≤ 25, -32 ≤ l ≤ 32
Reflections collected	192223
Independent reflections	11040 [R_{int} = 0.2324, R_{sigma} = 0.0926]
Data/restraints/parameters	11040/0/644
Goodness-of-fit on F^2	1.027
Final R indexes [I>=2σ (I)]	R_1 = 0.0759, wR_2 = 0.1883
Final R indexes [all data]	R_1 = 0.1207, wR_2 = 0.2217
Largest diff. peak/hole / e Å^{-3}	1.93/-0.90

[Co_2(hfac)$_4$(L2$_{Macro}$)]

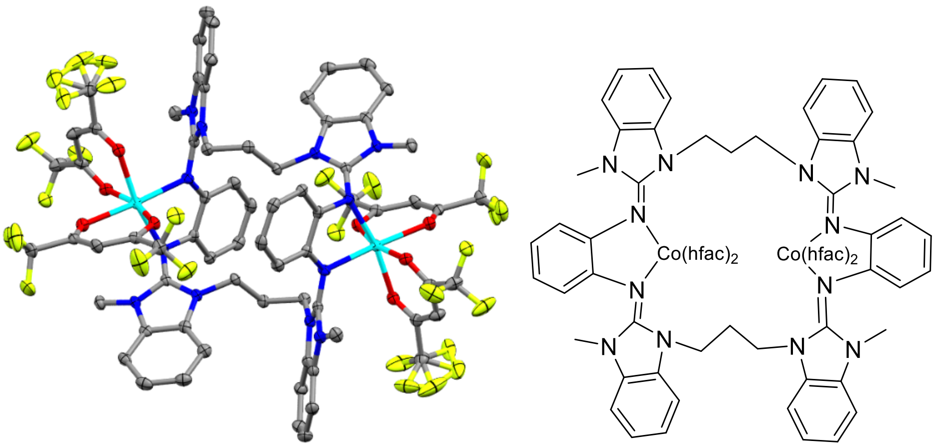

Table 25: Crystal and structural data of **[Co_2(hfac)$_4$(L2$_{Macro}$)]**.

Identification code	mo_2024_ee97_1_0ma
Deposition number	2477535
Empirical formula	$C_{70}H_{52}N_{12}O_8F_{24}Co_2$
Formula weight	1763.09
Temperature/K	100.00
Crystal system	monoclinic
Space group	$P2_1/c$
a/Å	12.6181(14)
b/Å	12.8586(13)
c/Å	22.741(2)
α/°	90
β/°	105.286(4)
γ/°	90
Volume/Å^3	3559.3(7)
Z	2
ρ_{calc}g/cm^3	1.645
μ/mm^{-1}	0.595
F(000)	1780.0
Crystal size/mm^3	0.18 × 0.17 × 0.1
Radiation	MoKα (λ = 0.71073)
2Θ range for data collection/°	3.672 to 52.998
Index ranges	-15 ≤ h ≤ 15, -16 ≤ k ≤ 16, -28 ≤ l ≤ 28
Reflections collected	119705
Independent reflections	7376 [R_{int} = 0.1252, R_{sigma} = 0.0463]
Data/restraints/parameters	7376/10/543
Goodness-of-fit on F^2	1.021
Final R indexes [I>=2σ (I)]	R_1 = 0.0412, wR_2 = 0.0897
Final R indexes [all data]	R_1 = 0.0617, wR_2 = 0.1020
Largest diff. peak/hole / e Å^{-3}	0.51/-0.44

[NiCl$_2$(L3)]

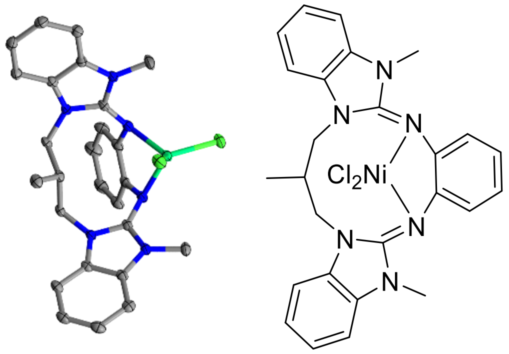

Table 26: Crystal and structural data of **[NiCl$_2$(L3)].**

Identification code	mo_2025_ee203_0m
Deposition number	2477543
Empirical formula	$C_{26}H6Cl_2N_6Ni\cdot 1.5\ CH_2Cl_2$
Formula weight	679.53
Temperature/K	101.00
Crystal system	triclinic
Space group	P-1
a/Å	12.3922(13)
b/Å	16.5375(16)
c/Å	16.8789(16)
α/°	73.022(4)
β/°	70.960(4)
γ/°	69.198(4)
Volume/Å^3	2995.4(5)
Z	4
ρ_{calc}g/cm^3	1.507
μ/mm^{-1}	1.123
F(000)	1396.0
Crystal size/mm^3	0.2 × 0.09 × 0.08
Radiation	MoKα (λ = 0.71073)
2Θ range for data collection/°	3.846 to 53.13
Index ranges	-15 ≤ h ≤ 15, -20 ≤ k ≤ 20, -21 ≤ l ≤ 21
Reflections collected	169785
Independent reflections	12395 [R_{int} = 0.0786, R_{sigma} = 0.0292]
Data/restraints/parameters	12395/0/664
Goodness-of-fit on F^2	1.079
Final R indexes [I>=2σ (I)]	R_1 = 0.0412, wR_2 = 0.1145
Final R indexes [all data]	R_1 = 0.0514, wR_2 = 0.1217
Largest diff. peak/hole / e Å^{-3}	0.39/-1.48

[$CoBr_2$(L4)]

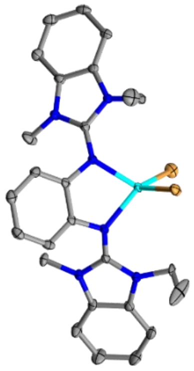

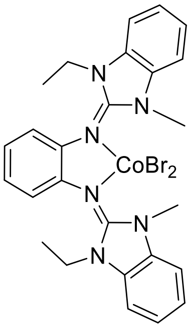

Table 27: Crystal and structural data of **[$CoBr_2$(L4)]**.

Identification code	mo_2024_ee132_0m
Deposition number	2477540
Empirical formula	$C_{27}H_{30}Br_2CoN_6$
Formula weight	728.22
Temperature/K	100.00
Crystal system	monoclinic
Space group	$P2_1/n$
a/Å	9.9722(7)
b/Å	12.7531(8)
c/Å	23.6314(12)
α/°	90
β/°	100.359(2)
γ/°	90
Volume/Å^3	2956.4(3)
Z	4
ρ_{calc}g/cm^3	1.636
μ/mm^{-1}	3.493
F(000)	1460.0
Crystal size/mm^3	0.39 × 0.37 × 0.27
Radiation	MoKα (λ = 0.71073)
2Θ range for data collection/°	4.206 to 53.992
Index ranges	-12 ≤ h ≤ 12, -16 ≤ k ≤ 16, -30 ≤ l ≤ 30
Reflections collected	120959
Independent reflections	6458 [R_{int} = 0.0908, R_{sigma} = 0.0438]
Data/restraints/parameters	6458/7/350
Goodness-of-fit on F^2	1.046
Final R indexes [I>=2σ (I)]	R_1 = 0.0344, wR_2 = 0.0835
Final R indexes [all data]	R_1 = 0.0365, wR_2 = 0.0849
Largest diff. peak/hole / e Å^{-3}	1.05/-0.64

[$Ni_3Cl_6(L4)_3$]

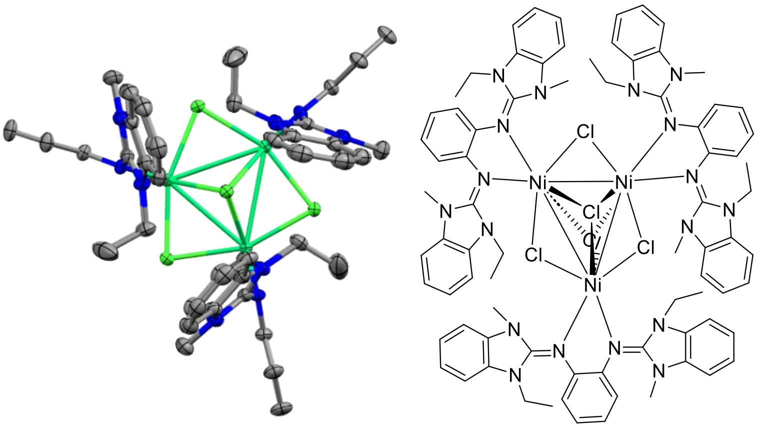

Table 28: Crystal and structural data of **[$Ni_3Cl_6(L4)_3$].**

Identification code	mo_2023_ee96_0
Deposition number	2477534
Empirical formula	$C_{78}H_{84}Cl_5N_{18}Ni_3$
Formula weight	1627.01
Temperature/K	120.00
Crystal system	hexagonal
Space group	P-62c
a/Å	13.6824(9)
b/Å	13.6824(9)
c/Å	24.615(2)
α/°	90
β/°	90
γ/°	120
Volume/Å^3	3990.8(6)
Z	2
ρ_{calc}g/cm^3	1.354
μ/mm^{-1}	0.922
F(000)	1694.0
Crystal size/mm^3	0.25 × 0.176 × 0.07
Radiation	MoKα (λ = 0.71073)
2Θ range for data collection/°	4.772 to 55.998
Index ranges	-18 ≤ h ≤ 18, -18 ≤ k ≤ 18, -32 ≤ l ≤ 32
Reflections collected	150239
Independent reflections	3294 [R_{int} = 0.0748, R_{sigma} = 0.0160]
Data/restraints/parameters	3294/0/163
Goodness-of-fit on F^2	1.070
Final R indexes [I>=2σ (I)]	R_1 = 0.0249, wR_2 = 0.0642
Final R indexes [all data]	R_1 = 0.0258, wR_2 = 0.0647
Largest diff. peak/hole / e Å^{-3}	0.39/-0.28

[Cu(BF$_4$)$_2$(L4)$_2$]

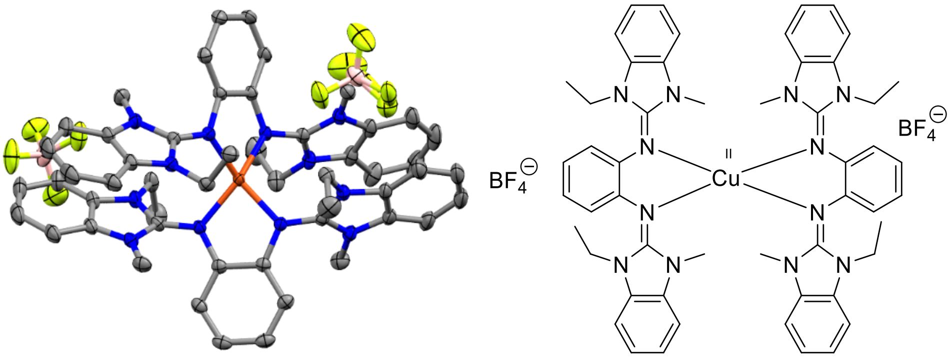

Table 29: Crystal and structural data of **[Cu(BF$_4$)$_2$(L4)$_2$]**.

Identification code	mo_2023_eepr5_2_0ma
Deposition number	2477550
Empirical formula	$C_{56}H_{62}B_2CuF_8N_{14}$
Formula weight	1168.35
Temperature/K	120.00
Crystal system	monoclinic
Space group	$P2_1/n$
a/Å	17.966(3)
b/Å	10.3908(17)
c/Å	30.400(4)
α/°	90
β/°	97.362(5)
γ/°	90
Volume/Å^3	5628.3(15)
Z	4
ρ_{calc}g/cm^3	1.379
μ/mm^{-1}	0.466
F(000)	2428.0
Crystal size/mm^3	0.32 × 0.32 × 0.292
Radiation	MoKα (λ = 0.71073)
2Θ range for data collection/°	4.146 to 55.87
Index ranges	$-23 \le h \le 23$, $-13 \le k \le 13$, $-40 \le l \le 39$
Reflections collected	281459
Independent reflections	13458 [R_{int} = 0.0867, R_{sigma} = 0.0384]
Data/restraints/parameters	13458/0/776
Goodness-of-fit on F^2	1.044
Final R indexes [I>=2σ (I)]	R_1 = 0.0423, wR_2 = 0.1112
Final R indexes [all data]	R_1 = 0.0469, wR_2 = 0.1154
Largest diff. peak/hole / e Å^{-3}	0.53/-0.57

$[CoBr_2(L5)]$

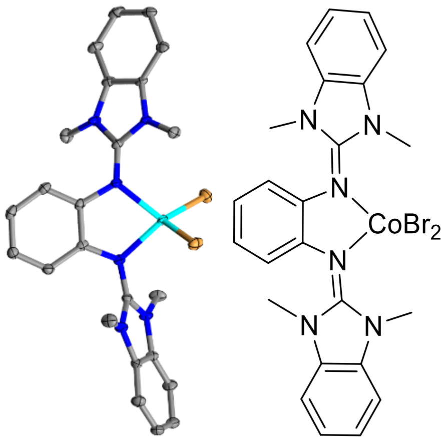

Table 30: Crystal and structural data of **$[CoBr_2(L5)]$**.

Identification code	mo_EE131_1_0ma
Deposition number	2477539
Empirical formula	$C_{24}H_{24}N_6CoBr_2$
Formula weight	615.24
Temperature/K	100.00
Crystal system	monoclinic
Space group	$P2_1/n$
a/Å	11.9916(18)
b/Å	9.7801(15)
c/Å	21.420(3)
α/°	90
β/°	103.108(5)
γ/°	90
Volume/Å^3	2446.7(6)
Z	4
ρ_{calc}g/cm^3	1.670
μ/mm^{-1}	3.992
F(000)	1228.0
Crystal size/mm^3	0.12 × 0.1 × 0.09
Radiation	MoKα (λ = 0.71073)
2Θ range for data collection/°	3.904 to 51.998
Index ranges	-14 ≤ h ≤ 14, -12 ≤ k ≤ 12, -26 ≤ l ≤ 26
Reflections collected	91401
Independent reflections	4792 [R_{int} = 0.1199, R_{sigma} = 0.0430]
Data/restraints/parameters	4792/0/302
Goodness-of-fit on F^2	1.077
Final R indexes [I>=2σ (I)]	R_1 = 0.0340, wR_2 = 0.0713
Final R indexes [all data]	R_1 = 0.0457, wR_2 = 0.0778
Largest diff. peak/hole / e Å^{-3}	0.46/-0.67

[Cu(BF$_4$)$_2$(L5)$_2$]

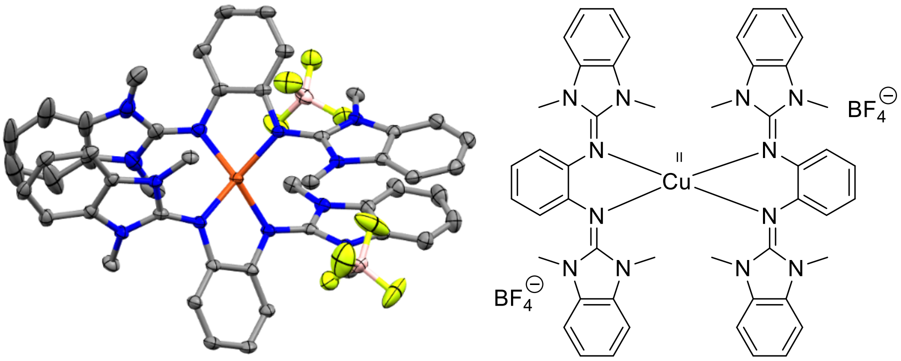

Table 31: Crystal and structural data of **[Cu(BF$_4$)$_2$(L5)$_2$]**.

Identification code	mo_2023_ee84_0ma
Deposition number	2477532
Empirical formula	$C_{48}H_{48}B_2CuF_8N_{12}\cdot 0.5\ CH_3CN$
Formula weight	1091.72
Temperature/K	120.00
Crystal system	monoclinic
Space group	$P2_1/c$
a/Å	17.3272(16)
b/Å	31.536(3)
c/Å	18.7767(15)
α/°	90
β/°	103.830(3)
γ/°	90
Volume/Å^3	9962.7(15)
Z	8
ρ_{calc}g/cm^3	1.456
μ/mm^{-1}	0.521
F(000)	4512.0
Crystal size/mm^3	0.31 × 0.18 × 0.15
Radiation	MoKα (λ = 0.71073)
2Θ range for data collection/°	3.886 to 55
Index ranges	-22 ≤ h ≤ 22, -40 ≤ k ≤ 40, -24 ≤ l ≤ 24
Reflections collected	579932
Independent reflections	22883 [R_{int} = 0.0838, R_{sigma} = 0.0343]
Data/restraints/parameters	22883/133/1398
Goodness-of-fit on F^2	1.034
Final R indexes [I>=2σ (I)]	R_1 = 0.0570, wR_2 = 0.1529
Final R indexes [all data]	R_1 = 0.0647, wR_2 = 0.1615
Largest diff. peak/hole / e Å^{-3}	1.88/-1.05

[$Co_2Br_4(L6_{Macro})$]

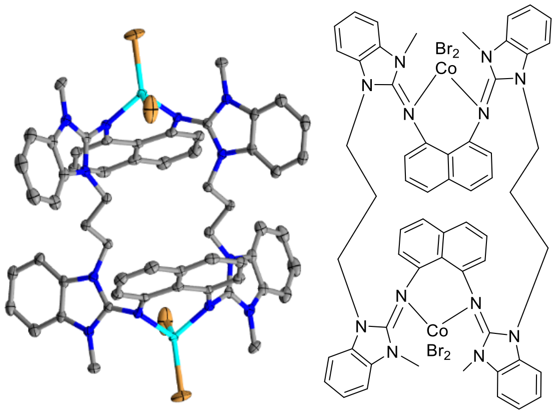

Table 32: Crystal and structural data of **[$Co_2Br_4(L6_{Macro})$]**.

Identification code	mo_2024_ee126_2_0ma
Deposition number	2477538
Empirical formula	$C_{60}H_{56}Br_4Cl_4Co_2N_{12}$
Formula weight	1524.46
Temperature/K	100.00
Crystal system	monoclinic
Space group	$P2_1/n$
a/Å	14.9339(7)
b/Å	11.5559(5)
c/Å	17.8057(9)
α/°	90
β/°	93.936(2)
γ/°	90
Volume/Å^3	3065.6(2)
Z	2
ρ_{calc}g/cm^3	1.652
μ/mm^{-1}	3.372
F(000)	1524.0
Crystal size/mm^3	0.121 × 0.079 × 0.057
Radiation	MoKα (λ = 0.71073)
2Θ range for data collection/°	4.204 to 54.842
Index ranges	-19 ≤ h ≤ 19, -14 ≤ k ≤ 14, -23 ≤ l ≤ 23
Reflections collected	129834
Independent reflections	6981 [R_{int} = 0.0880, R_{sigma} = 0.0285]
Data/restraints/parameters	6981/6/391
Goodness-of-fit on F^2	1.052
Final R indexes [I>=2σ (I)]	R_1 = 0.0351, wR_2 = 0.0846
Final R indexes [all data]	R_1 = 0.0460, wR_2 = 0.0911
Largest diff. peak/hole / e Å^{-3}	0.98/-0.87

Literature

[1] U. Jahn, P. Hartmann, I. Dix, P.G. Jones *J. Org. Chem.* **2001**, *17*, 3333–3355.

[2] SAINT (APEX III/IV) Bruker AXS GmbH, Karlsruhe, Germany **2016/2021**.

[3] a) G. M. Sheldrick, SADABS, Bruker AXS GmbH, Karlsruhe, Germany **2004-2014**; b) L. Krause, R. Herbst-Irmer, G. M. Sheldrick, D. Stalke, *J. Appl. Cryst*. **2015**, 48, 3–10.

[4] a) G. M. Sheldrick, SHELXT, Program for Crystal Structure Solution, University of Göttingen, Germany **2014-2018**; b) G. M. Sheldrick, *Acta Cryst*. **2015**, C71, 3–8.

[5] a) G. M. Sheldrick, SHELXL-20xx, University of Göttingen and Bruker AXS GmbH, Karlsruhe, Germany **2012-2018**; b) W. Robinson, G. M. Sheldrick in: N. W. Isaaks, M. R. Taylor (eds.) „*Crystallographic Computing 4*“, Ch. 22, IUCr and Oxford University Press, Oxford, UK, **1988**; c) G. M. Sheldrick*, Acta Cryst.* **2008**, A64, 112–122.

[6] O. V. Dolomanov, L. J. Bourhis, R. J. Gildea, J. A. K. Howard, H. Puschmann, *J. Appl. Cryst.* **2009**, 42, 339–341.

[7] A. Thorn, B. Dittrich, G. M. Sheldrick, *Acta Cryst.* **2012**, *A68*, 448–451.

[8] a) P. v. d. Sluis, A. L. Spek*, Acta Cryst.* **1990**, *A46*, 194–201; b) A. L. Spek, Acta Cryst. **2015**, *C71*, 9–18.

[9] A. L. Spek, PLATON, Utrecht University, The Netherlands; b) A. L. Spek, J. *Appl. Cryst.* **2003**, 36, 7–13.

[10] M. Werr, Dissertation, Ruprecht-Karls-Universität Heidelberg, Heidelberg, **2022**.

[11] S. L. Buchwald, E. J. Hennesy, *Org. Lett.* **2002**, *4*, 269–272.

[12] J. L. Moore; S. M. Taylor, V. A. Soloshonok, *Arkivoc*. **2005** *(part vi): 287–292*.

[13] U. Wild, E. Engels, O. Hübner, E. Kaifer, H.-J. Himmel, *Chem. Eur. J.* **2024**, *30*, e202403080 (1-14).

[14] V. P. W. Böhm, W. A. Herrmann, *Angew. Chem. Int. Ed.* **2000**, *39*, 4036-4038.

[15] B. Kovačević, Z. B. Maksić, *Org. Lett.* **2001**, *3*, 1523–1526.

[16] B. Kovačević, Z. B. Maksić, R. Vianello, M. Primorac. *New J. Chem.* **2002**, 26, 1329–1334.

[17] S. D. Lepore.; A. Khoram; D.C. Bromfield; P. Cohn; V. Jairaj; M.A. Silvestri, *J. Org. Chem*. **2005**, *70*, 7443–7446.
