# Supplementary material for: Steric Restraints in Redox‐Active Guanidine Ligands and Their Impact on Coordination Chemistry
Source: Chemistry. 2025 Oct 25;31(66):e02457. doi: 10.1002/chem.202502457 (PMC12648461; doi:10.1002/chem.202502457)

checkCIF/PLATON report

Structure factors have been supplied for datablock(s) mo_2023_eepr5_2_0ma

THIS REPORT IS FOR GUIDANCE ONLY. IF USED AS PART OF A REVIEW PROCEDURE FOR PUBLICATION, IT SHOULD NOT REPLACE THE EXPERTISE OF AN EXPERIENCED CRYSTALLOGRAPHIC REFEREE.

No syntax errors found. CIF dictionary Interpreting this report

Datablock: mo_2023_eepr5_2_0ma

Bond precision:	C-C = 0.0027 A		Wavelength=0.71073
Cell:	a=17.966(3)	b=10.3908(17)	c=30.400(4)
	alpha=90	beta=97.362(5)	gamma=90
Temperature:	120 K		

	Calculated	Reported
Volume	5628.3(15)	5628.3(15)
Space group	P 21/n	P 1 21/n 1
Hall group	-P 2yn	-P 2yn
Moiety formula	C52 H56 Cu N12, 2(B F4), 2(C2 H3 N)	C52 H56 Cu N12, 2(B F4), 2(C2 H3 N)
Sum formula	C56 H62 B2 Cu F8 N14	C56 H62 B2 Cu F8 N14
Mr	1168.37	1168.35
Dx,g cm-3	1.379	1.379
Z	4	4
Mu (mm-1)	0.466	0.466
F000	2428.0	2428.0
F000’	2430.34	
h,k,lmax	23,13,40	23,13,40
Nref	13507	13458
Tmin,Tmax	0.861,0.873	0.666,0.734
Tmin’	0.861	

Correction method= # Reported T Limits: Tmin=0.666 Tmax=0.734
AbsCorr = MULTI-SCAN

Data completeness= 0.996 Theta(max)= 27.935

R(reflections)= 0.0423(12176) wR2(reflections)= 0.1154(13458)

S = 1.044 Npar= 776

The following ALERTS were generated. Each ALERT has the format
test-name_ALERT_alert-type_alert-level.
Click on the hyperlinks for more details of the test.

Alert level C

```
PLAT243_ALERT_4_C High   'Solvent' Ueq as Compared to Neighbors of        C56 Check
PLAT244_ALERT_4_C Low    'Solvent' Ueq as Compared to Neighbors of         B2 Check
PLAT244_ALERT_4_C Low    'Solvent' Ueq as Compared to Neighbors of        C53 Check
PLAT911_ALERT_3_C Missing FCF Refl Between Thmin & STh/L=    0.600          7 Report
```

Alert level G

```
PLAT244_ALERT_4_G Low    'Solvent' Ueq as Compared to Neighbors of         B1 Check
PLAT300_ALERT_4_G Atom Site Occupancy of F6         Constrained at        0.7 Check
PLAT300_ALERT_4_G Atom Site Occupancy of F8         Constrained at        0.7 Check
PLAT300_ALERT_4_G Atom Site Occupancy of F9         Constrained at        0.3 Check
PLAT300_ALERT_4_G Atom Site Occupancy of F10        Constrained at        0.3 Check
PLAT300_ALERT_4_G Atom Site Occupancy of N14        Constrained at        0.9 Check
PLAT300_ALERT_4_G Atom Site Occupancy of C55        Constrained at        0.9 Check
PLAT300_ALERT_4_G Atom Site Occupancy of N15        Constrained at        0.1 Check
PLAT300_ALERT_4_G Atom Site Occupancy of C57        Constrained at        0.1 Check
PLAT300_ALERT_4_G Atom Site Occupancy of H56A       Constrained at        0.9 Check
PLAT300_ALERT_4_G Atom Site Occupancy of H56B       Constrained at        0.9 Check
PLAT300_ALERT_4_G Atom Site Occupancy of H56C       Constrained at        0.9 Check
PLAT300_ALERT_4_G Atom Site Occupancy of H56D       Constrained at        0.1 Check
PLAT300_ALERT_4_G Atom Site Occupancy of H56E       Constrained at        0.1 Check
PLAT300_ALERT_4_G Atom Site Occupancy of H56F       Constrained at        0.1 Check
PLAT302_ALERT_4_G Anion/Solvent/Minor-Residue Disorder (Resd  2  )        40% Note
PLAT302_ALERT_4_G Anion/Solvent/Minor-Residue Disorder (Resd  4  )        67% Note
PLAT432_ALERT_2_G Short Inter X...Y Contact  F8       ..C40      .       2.96 Ang.
                                                       x,y,z  =      1_555 Check
PLAT432_ALERT_2_G Short Inter X...Y Contact  F10      ..C40      .       2.96 Ang.
                                                       x,y,z  =      1_555 Check
PLAT720_ALERT_4_G Number of Unusual/Non-Standard Labels .........          1 Note
PLAT910_ALERT_3_G Missing # of FCF Reflection(s) Below Theta(Min).          3 Note
PLAT912_ALERT_4_G Missing # of FCF Reflections Above STh/L=  0.600         38 Note
PLAT913_ALERT_3_G Missing # of Very Strong Reflections in FCF ....          1 Note
PLAT933_ALERT_2_G Number of HKL-OMIT Records in Embedded .res File          1 Note
PLAT955_ALERT_1_G Reported (CIF) and Actual (FCF) Lmax Differ by .          1 Units
PLAT978_ALERT_2_G Number C-C Bonds with Positive Residual Density.          6 Info
```

0 **ALERT level A** = Most likely a serious problem - resolve or explain
0 **ALERT level B** = A potentially serious problem, consider carefully
4 **ALERT level C** = Check. Ensure it is not caused by an omission or oversight
26 **ALERT level G** = General information/check it is not something unexpected

1 ALERT type 1 CIF construction/syntax error, inconsistent or missing data
4 ALERT type 2 Indicator that the structure model may be wrong or deficient
3 ALERT type 3 Indicator that the structure quality may be low
22 ALERT type 4 Improvement, methodology, query or suggestion
0 ALERT type 5 Informative message, check

It is advisable to attempt to resolve as many as possible of the alerts in all categories. Often the minor alerts point to easily fixed oversights, errors and omissions in your CIF or refinement strategy, so attention to these fine details can be worthwhile. In order to resolve some of the more serious problems it may be necessary to carry out additional measurements or structure refinements. However, the purpose of your study may justify the reported deviations and the more serious of these should normally be commented upon in the discussion or experimental section of a paper or in the "special_details" fields of the CIF. checkCIF was carefully designed to identify outliers and unusual parameters, but every test has its limitations and alerts that are not important in a particular case may appear. Conversely, the absence of alerts does not guarantee there are no aspects of the results needing attention. It is up to the individual to critically assess their own results and, if necessary, seek expert advice.

Publication of your CIF in IUCr journals

A basic structural check has been run on your CIF. These basic checks will be run on all CIFs submitted for publication in IUCr journals (*Acta Crystallographica*, *Journal of Applied Crystallography*, *Journal of Synchrotron Radiation*); however, if you intend to submit to *Acta Crystallographica Section C* or *E* or *IUCrData*, you should make sure that full publication checks are run on the final version of your CIF prior to submission.

Publication of your CIF in other journals

Please refer to the *Notes for Authors* of the relevant journal for any special instructions relating to CIF submission.

PLATON version of 06/07/2023; check.def file version of 30/06/2023

Datablock mo_2023_eepr5_2_0ma - ellipsoid plot

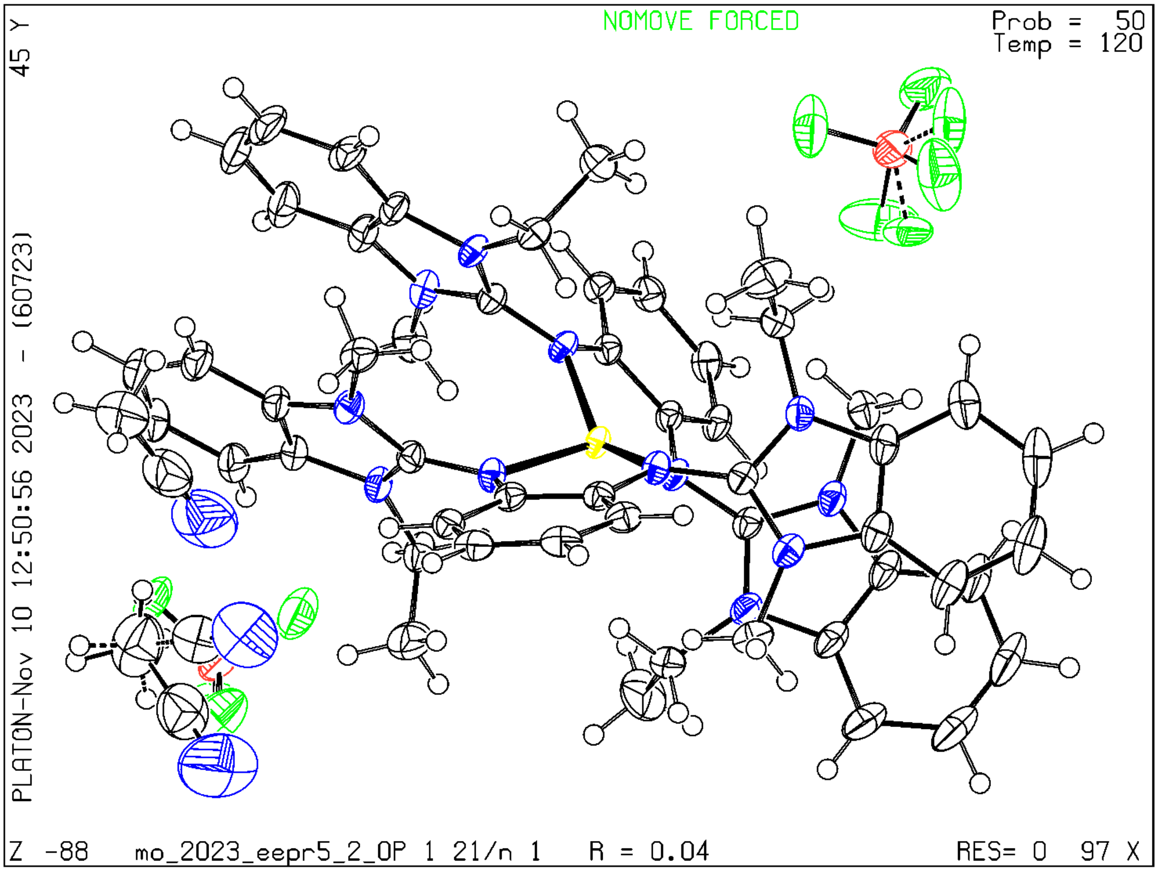

Supplement: Supplementary file 2 — Supporting Information [file CHEM-31-e02457-s002.zip › mo_2023_eepr5_2_0ma_cifreport.pdf]
