# Supplementary material for: Steric Restraints in Redox‐Active Guanidine Ligands and Their Impact on Coordination Chemistry
Source: Chemistry. 2025 Oct 25;31(66):e02457. doi: 10.1002/chem.202502457 (PMC12648461; doi:10.1002/chem.202502457)

checkCIF/PLATON report

Structure factors have been supplied for datablock(s) mo_2023_ee75_0ma

THIS REPORT IS FOR GUIDANCE ONLY. IF USED AS PART OF A REVIEW PROCEDURE FOR PUBLICATION, IT SHOULD NOT REPLACE THE EXPERTISE OF AN EXPERIENCED CRYSTALLOGRAPHIC REFEREE.

No syntax errors found. CIF dictionary Interpreting this report

Datablock: mo_2023_ee75_0ma

Bond precision: C-C = 0.0051 A Wavelength=0.71073

Cell:	a=16.115(3)	b=11.967(2)	c=12.403(3)
	alpha=90	beta=112.326(7)	gamma=90
Temperature:	100 K		

	Calculated	Reported
Volume	2212.6(8)	2212.7(8)
Space group	P 2/c	P 1 2/c 1
Hall group	-P 2yc	-P 2yc
Moiety formula	C26 H28 N6	2(C13 H14 N3)
Sum formula	C26 H28 N6	C26 H28 N6
Mr	424.54	424.54
Dx,g cm-3	1.275	1.274
Z	4	4
Mu (mm-1)	0.078	0.078
F000	904.0	904.0
F000'	904.27	
h,k,lmax	19,14,15	19,14,15
Nref	4356	4343
Tmin,Tmax	0.984,0.992	0.627,0.746
Tmin'	0.970	

Correction method= # Reported T Limits: Tmin=0.627 Tmax=0.746
AbsCorr = MULTI-SCAN

Data completeness= 0.997 Theta(max)= 25.999

R(reflections)= 0.0899(4065) wR2(reflections)= 0.2292(4343)

S = 1.165 Npar= 294

The following ALERTS were generated. Each ALERT has the format
test-name_ALERT_alert-type_alert-level.
Click on the hyperlinks for more details of the test.

Alert level B

```
PLAT097_ALERT_2_B Large Reported Max.  (Positive) Residual Density       1.11 eA-3
```

Alert level C

```
DIFMX02_ALERT_1_C  The maximum difference density is > 0.1*ZMAX*0.75
            The relevant atom site should be identified.
PLAT042_ALERT_1_C Calc. and Reported MoietyFormula Strings  Differ     Please Check
PLAT094_ALERT_2_C Ratio of Maximum / Minimum Residual Density ....       2.91 Report
PLAT230_ALERT_2_C Hirshfeld Test Diff for    N4       --C24      .        6.0 s.u.
PLAT340_ALERT_3_C Low Bond Precision on  C-C Bonds ...............    0.00514 Ang.
PLAT906_ALERT_3_C Large K Value in the Analysis of Variance ......      7.620 Check
PLAT906_ALERT_3_C Large K Value in the Analysis of Variance ......      2.262 Check
PLAT911_ALERT_3_C Missing FCF Refl Between Thmin & STh/L=    0.600         14 Report
PLAT913_ALERT_3_C Missing # of Very Strong Reflections in FCF ....          7 Note
```

Alert level G

```
PLAT083_ALERT_2_G SHELXL Second Parameter in WGHT  Unusually Large       5.46 Why ?
PLAT883_ALERT_1_G No Info/Value for _atom_sites_solution_primary .     Please Do !
PLAT933_ALERT_2_G Number of HKL-OMIT Records in Embedded .res File          5 Note
PLAT967_ALERT_5_G Note: Two-Theta Cutoff Value in Embedded .res ..       52.0 Degree
PLAT978_ALERT_2_G Number C-C Bonds with Positive Residual Density.          2 Info
```

0 **ALERT level A** = Most likely a serious problem - resolve or explain
1 **ALERT level B** = A potentially serious problem, consider carefully
9 **ALERT level C** = Check. Ensure it is not caused by an omission or oversight
5 **ALERT level G** = General information/check it is not something unexpected

3 ALERT type 1 CIF construction/syntax error, inconsistent or missing data
6 ALERT type 2 Indicator that the structure model may be wrong or deficient
5 ALERT type 3 Indicator that the structure quality may be low
0 ALERT type 4 Improvement, methodology, query or suggestion
1 ALERT type 5 Informative message, check

It is advisable to attempt to resolve as many as possible of the alerts in all categories. Often the minor alerts point to easily fixed oversights, errors and omissions in your CIF or refinement strategy, so attention to these fine details can be worthwhile. In order to resolve some of the more serious problems it may be necessary to carry out additional measurements or structure refinements. However, the purpose of your study may justify the reported deviations and the more serious of these should normally be commented upon in the discussion or experimental section of a paper or in the "special_details" fields of the CIF. checkCIF was carefully designed to identify outliers and unusual parameters, but every test has its limitations and alerts that are not important in a particular case may appear. Conversely, the absence of alerts does not guarantee there are no aspects of the results needing attention. It is up to the individual to critically assess their own results and, if necessary, seek expert advice.

Publication of your CIF in IUCr journals

A basic structural check has been run on your CIF. These basic checks will be run on all CIFs submitted for publication in IUCr journals (*Acta Crystallographica*, *Journal of Applied Crystallography*, *Journal of Synchrotron Radiation*); however, if you intend to submit to *Acta Crystallographica Section C* or *E* or *IUCrData*, you should make sure that full publication checks are run on the final version of your CIF prior to submission.

Publication of your CIF in other journals

Please refer to the *Notes for Authors* of the relevant journal for any special instructions relating to CIF submission.

PLATON version of 06/07/2023; check.def file version of 30/06/2023

Datablock mo_2023_ee75_0ma - ellipsoid plot

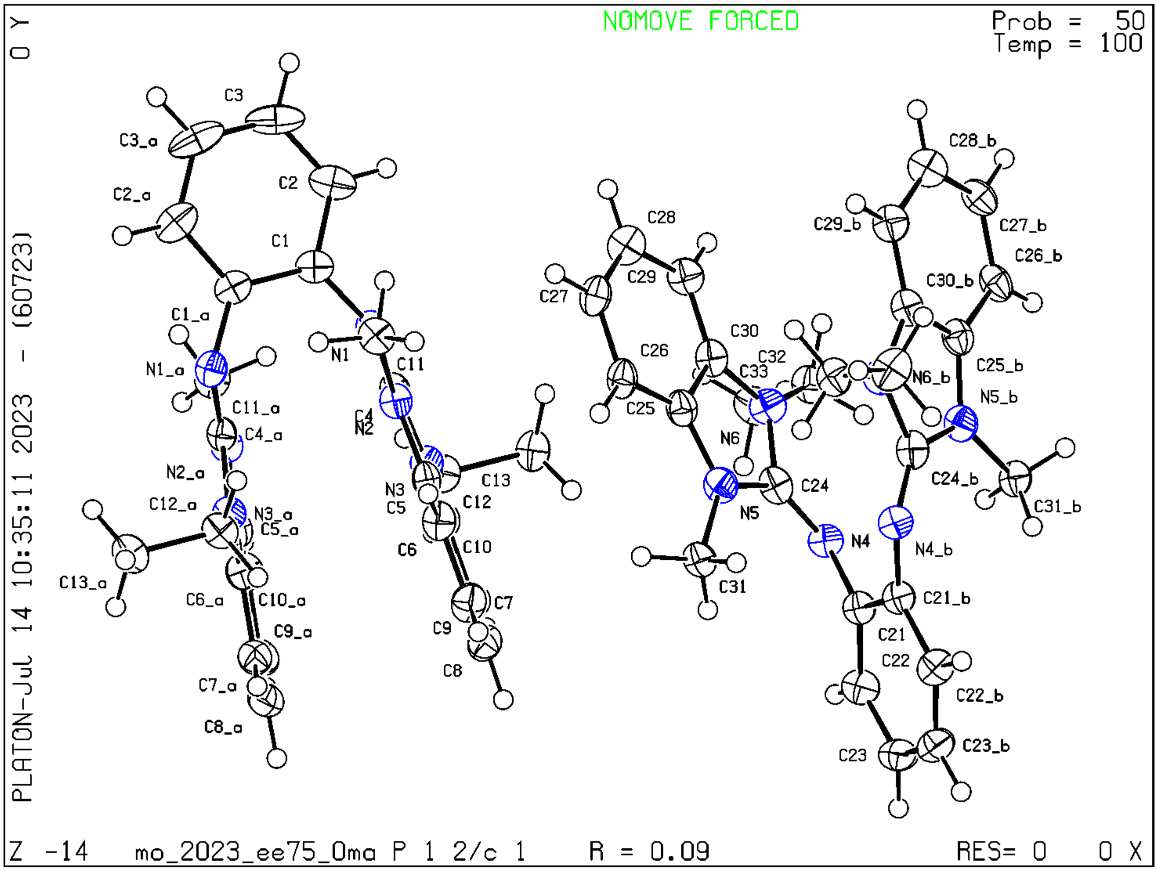

Supplement: Supplementary file 2 — Supporting Information [file CHEM-31-e02457-s002.zip › mo_2023_ee75_0ma_cifreport.pdf]
