# Supplementary material for: Steric Restraints in Redox‐Active Guanidine Ligands and Their Impact on Coordination Chemistry
Source: Chemistry. 2025 Oct 25;31(66):e02457. doi: 10.1002/chem.202502457 (PMC12648461; doi:10.1002/chem.202502457)

checkCIF/PLATON report

Structure factors have been supplied for datablock(s) mo_2023_ee79_01a

THIS REPORT IS FOR GUIDANCE ONLY. IF USED AS PART OF A REVIEW PROCEDURE FOR PUBLICATION, IT SHOULD NOT REPLACE THE EXPERTISE OF AN EXPERIENCED CRYSTALLOGRAPHIC REFEREE.

No syntax errors found. CIF dictionary Interpreting this report

Datablock: mo_2023_ee79_01a

Bond precision:	C-C = 0.0042 A		Wavelength=0.71073
Cell:	a=32.120(3)	b=12.7754(9)	c=16.9043(11)
	alpha=90	beta=121.726(4)	gamma=90
Temperature:	100 K		

	Calculated	Reported
Volume	5900.1(8)	5900.0(8)
Space group	C 2/c	C 1 2/c 1
Hall group	-C 2yc	-C 2yc
Moiety formula	C26 H30 N6, 2(Cl) [+ solvent]	2(Cl), C26 H30 N6
Sum formula	C26 H30 Cl2 N6 [+ solvent]	C26 H30 Cl2 N6
Mr	497.46	497.46
Dx,g cm-3	1.120	1.120
Z	8	8
Mu (mm-1)	0.243	0.243
F000	2096.0	2096.0
F000'	2098.93	
h,k,lmax	40,16,21	40,16,21
Nref	6129	6122
Tmin,Tmax	0.911,0.936	0.669,0.746
Tmin'	0.883	

Correction method= # Reported T Limits: Tmin=0.669 Tmax=0.746
AbsCorr = MULTI-SCAN

Data completeness= 0.999 Theta(max)= 26.499

R(reflections)= 0.0610(5228) wR2(reflections)= 0.1824(6122)

S = 1.041 Npar= 341

The following ALERTS were generated. Each ALERT has the format
test-name_ALERT_alert-type_alert-level.
Click on the hyperlinks for more details of the test.

Alert level C

PLAT340_ALERT_3_C	Low Bond Precision on C-C Bonds	0.00422	Ang.
PLAT906_ALERT_3_C	Large K Value in the Analysis of Variance	2.607	Check
PLAT911_ALERT_3_C	Missing FCF Refl Between Thmin & STh/L= 0.600	4	Report
PLAT918_ALERT_3_C	Reflection(s) with I(obs) much Smaller I(calc) .	1	Check

Alert level G

PLAT002_ALERT_2_G	Number of Distance or Angle Restraints on AtSite	4	Note
PLAT003_ALERT_2_G	Number of Uiso or Uij Restrained non-H Atoms ...	8	Report
PLAT042_ALERT_1_G	Calc. and Reported MoietyFormula Strings Differ	Please	Check
PLAT083_ALERT_2_G	SHELXL Second Parameter in WGHT Unusually Large	11.13	Why ?
PLAT128_ALERT_4_G	Alternate Setting for Input Space Group C2/c	I2/a	Note
PLAT168_ALERT_4_G	The CIF-Embedded .res File Contains EXYZ Records	4	Report
PLAT171_ALERT_4_G	The CIF-Embedded .res File Contains EADP Records	4	Report
PLAT172_ALERT_4_G	The CIF-Embedded .res File Contains DFIX Records	2	Report
PLAT178_ALERT_4_G	The CIF-Embedded .res File Contains SIMU Records	4	Report
PLAT230_ALERT_2_G	Hirshfeld Test Diff for C15 --C16 .	8.8	s.u.
PLAT300_ALERT_4_G	Atom Site Occupancy of C14 Constrained at	0.7	Check
PLAT300_ALERT_4_G	Atom Site Occupancy of C15 Constrained at	0.7	Check
PLAT300_ALERT_4_G	Atom Site Occupancy of C16 Constrained at	0.7	Check
PLAT300_ALERT_4_G	Atom Site Occupancy of C24 Constrained at	0.7	Check
PLAT300_ALERT_4_G	Atom Site Occupancy of C25 Constrained at	0.7	Check
PLAT300_ALERT_4_G	Atom Site Occupancy of C26 Constrained at	0.7	Check
PLAT300_ALERT_4_G	Atom Site Occupancy of C14B Constrained at	0.3	Check
PLAT300_ALERT_4_G	Atom Site Occupancy of C15B Constrained at	0.3	Check
PLAT300_ALERT_4_G	Atom Site Occupancy of C16B Constrained at	0.3	Check
PLAT300_ALERT_4_G	Atom Site Occupancy of C24B Constrained at	0.3	Check
PLAT300_ALERT_4_G	Atom Site Occupancy of C25B Constrained at	0.3	Check
PLAT300_ALERT_4_G	Atom Site Occupancy of C26B Constrained at	0.3	Check
PLAT300_ALERT_4_G	Atom Site Occupancy of H14A Constrained at	0.7	Check
PLAT300_ALERT_4_G	Atom Site Occupancy of H14B Constrained at	0.7	Check
PLAT300_ALERT_4_G	Atom Site Occupancy of H14C Constrained at	0.7	Check
PLAT300_ALERT_4_G	Atom Site Occupancy of H15A Constrained at	0.7	Check
PLAT300_ALERT_4_G	Atom Site Occupancy of H15B Constrained at	0.7	Check
PLAT300_ALERT_4_G	Atom Site Occupancy of H16A Constrained at	0.7	Check
PLAT300_ALERT_4_G	Atom Site Occupancy of H16B Constrained at	0.7	Check
PLAT300_ALERT_4_G	Atom Site Occupancy of H16C Constrained at	0.7	Check
PLAT300_ALERT_4_G	Atom Site Occupancy of H24A Constrained at	0.7	Check
PLAT300_ALERT_4_G	Atom Site Occupancy of H24B Constrained at	0.7	Check
PLAT300_ALERT_4_G	Atom Site Occupancy of H24C Constrained at	0.7	Check
PLAT300_ALERT_4_G	Atom Site Occupancy of H25A Constrained at	0.7	Check
PLAT300_ALERT_4_G	Atom Site Occupancy of H25B Constrained at	0.7	Check
PLAT300_ALERT_4_G	Atom Site Occupancy of H26A Constrained at	0.7	Check
PLAT300_ALERT_4_G	Atom Site Occupancy of H26B Constrained at	0.7	Check
PLAT300_ALERT_4_G	Atom Site Occupancy of H26C Constrained at	0.7	Check
PLAT300_ALERT_4_G	Atom Site Occupancy of H14D Constrained at	0.3	Check
PLAT300_ALERT_4_G	Atom Site Occupancy of H14E Constrained at	0.3	Check
PLAT300_ALERT_4_G	Atom Site Occupancy of H15C Constrained at	0.3	Check
PLAT300_ALERT_4_G	Atom Site Occupancy of H15D Constrained at	0.3	Check
PLAT300_ALERT_4_G	Atom Site Occupancy of H15E Constrained at	0.3	Check

```
PLAT300_ALERT_4_G Atom Site Occupancy of H16D       Constrained at        0.3 Check
PLAT300_ALERT_4_G Atom Site Occupancy of H16E       Constrained at        0.3 Check
PLAT300_ALERT_4_G Atom Site Occupancy of H16F       Constrained at        0.3 Check
PLAT300_ALERT_4_G Atom Site Occupancy of H24D       Constrained at        0.3 Check
PLAT300_ALERT_4_G Atom Site Occupancy of H24E       Constrained at        0.3 Check
PLAT300_ALERT_4_G Atom Site Occupancy of H25C       Constrained at        0.3 Check
PLAT300_ALERT_4_G Atom Site Occupancy of H25D       Constrained at        0.3 Check
PLAT300_ALERT_4_G Atom Site Occupancy of H25E       Constrained at        0.3 Check
PLAT300_ALERT_4_G Atom Site Occupancy of H26D       Constrained at        0.3 Check
PLAT300_ALERT_4_G Atom Site Occupancy of H26E       Constrained at        0.3 Check
PLAT300_ALERT_4_G Atom Site Occupancy of H26F       Constrained at        0.3 Check
PLAT301_ALERT_3_G Main Residue  Disorder .............(Resd  1  )        19% Note
PLAT414_ALERT_2_G Short Intra D-H..H-X       H4       ..H25C     .       2.00 Ang.
                                                      x,y,z  =      1_555 Check
PLAT605_ALERT_4_G Largest Solvent Accessible VOID in the Structure        99 A**3
PLAT860_ALERT_3_G Number of Least-Squares Restraints ............         26 Note
PLAT868_ALERT_4_G ALERTS Due to the Use of _smtbx_masks Suppressed         ! Info
PLAT883_ALERT_1_G No Info/Value for _atom_sites_solution_primary .     Please Do !
PLAT910_ALERT_3_G Missing # of FCF Reflection(s) Below Theta(Min).         3 Note
PLAT933_ALERT_2_G Number of HKL-OMIT Records in Embedded .res File         3 Note
PLAT967_ALERT_5_G Note: Two-Theta Cutoff Value in Embedded .res ..      53.0 Degree
PLAT978_ALERT_2_G Number C-C Bonds with Positive Residual Density.         7 Info
```

```
  0 ALERT level A = Most likely a serious problem - resolve or explain
  0 ALERT level B = A potentially serious problem, consider carefully
  4 ALERT level C = Check. Ensure it is not caused by an omission or oversight
 64 ALERT level G = General information/check it is not something unexpected

  2 ALERT type 1 CIF construction/syntax error, inconsistent or missing data
  7 ALERT type 2 Indicator that the structure model may be wrong or deficient
  7 ALERT type 3 Indicator that the structure quality may be low
 51 ALERT type 4 Improvement, methodology, query or suggestion
  1 ALERT type 5 Informative message, check
```

It is advisable to attempt to resolve as many as possible of the alerts in all categories. Often the minor alerts point to easily fixed oversights, errors and omissions in your CIF or refinement strategy, so attention to these fine details can be worthwhile. In order to resolve some of the more serious problems it may be necessary to carry out additional measurements or structure refinements. However, the purpose of your study may justify the reported deviations and the more serious of these should normally be commented upon in the discussion or experimental section of a paper or in the "special_details" fields of the CIF. checkCIF was carefully designed to identify outliers and unusual parameters, but every test has its limitations and alerts that are not important in a particular case may appear. Conversely, the absence of alerts does not guarantee there are no aspects of the results needing attention. It is up to the individual to critically assess their own results and, if necessary, seek expert advice.

Publication of your CIF in IUCr journals

A basic structural check has been run on your CIF. These basic checks will be run on all CIFs submitted for publication in IUCr journals (*Acta Crystallographica*, *Journal of Applied Crystallography*, *Journal of Synchrotron Radiation*); however, if you intend to submit to *Acta Crystallographica Section C* or *E* or *IUCrData*, you should make sure that full publication checks are run on the final version of your CIF prior to submission.

Publication of your CIF in other journals

Please refer to the *Notes for Authors* of the relevant journal for any special instructions relating to CIF submission.

PLATON version of 10/05/2023; check.def file version of 10/05/2023

Datablock mo_2023_ee79_01a - ellipsoid plot

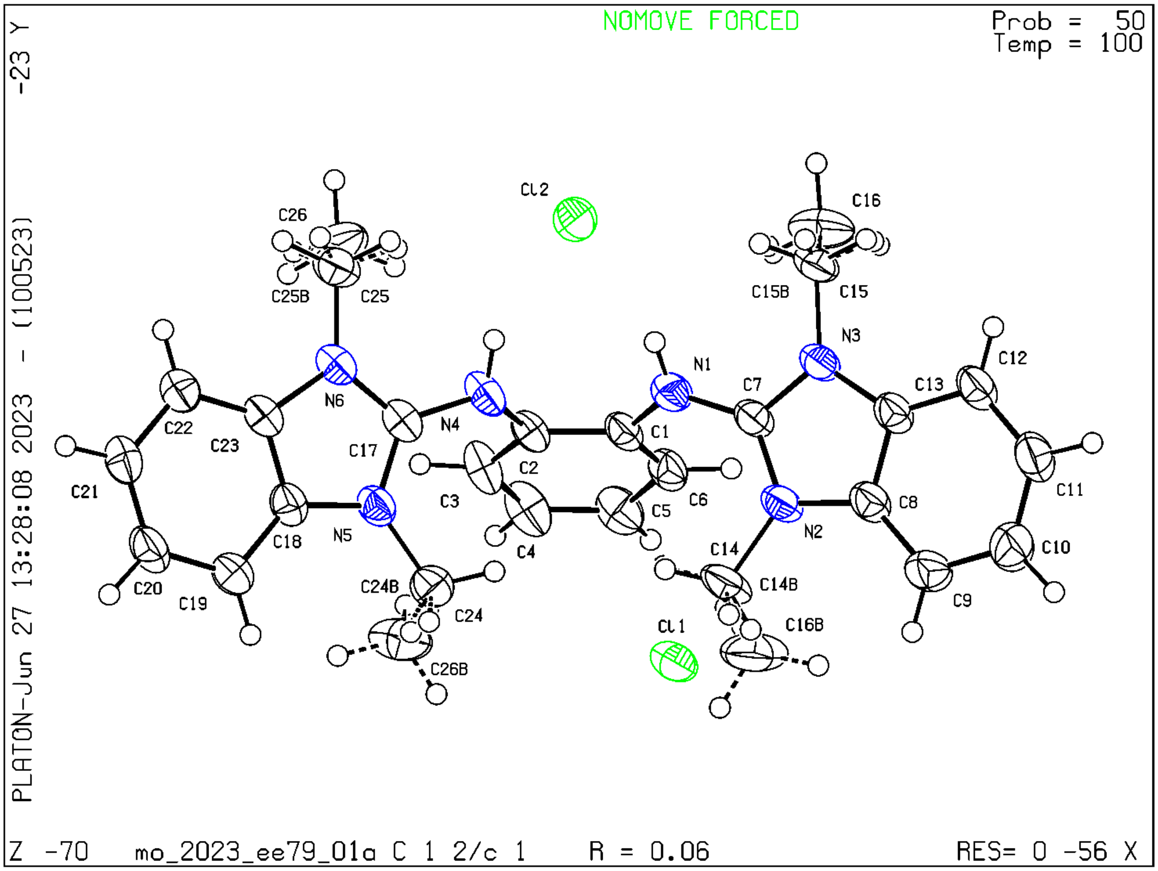

Supplement: Supplementary file 2 — Supporting Information [file CHEM-31-e02457-s002.zip › mo_2023_ee79_01a_cifreport.pdf]
