# Supplementary material for: Steric Restraints in Redox‐Active Guanidine Ligands and Their Impact on Coordination Chemistry
Source: Chemistry. 2025 Oct 25;31(66):e02457. doi: 10.1002/chem.202502457 (PMC12648461; doi:10.1002/chem.202502457)

checkCIF/PLATON report

Structure factors have been supplied for datablock(s) mo_2023_ee81_2_0ma

THIS REPORT IS FOR GUIDANCE ONLY. IF USED AS PART OF A REVIEW PROCEDURE FOR PUBLICATION, IT SHOULD NOT REPLACE THE EXPERTISE OF AN EXPERIENCED CRYSTALLOGRAPHIC REFEREE.

No syntax errors found. CIF dictionary Interpreting this report

Datablock: mo_2023_ee81_2_0ma

Bond precision:	C-C = 0.0066 A	Wavelength=0.71073	
Cell:	a=24.113(3)	b=13.1508(14)	c=17.202(2)
	alpha=90	beta=99.827(4)	gamma=90
Temperature:	120 K		

	Calculated	Reported
Volume	5374.8(11)	5374.9(10)
Space group	P 21/c	P 1 21/c 1
Hall group	-P 2ybc	-P 2ybc
Moiety formula	C26 H29 N6, F6 P, 0.2(H2 O)	F6 P, C26 H29 N6, 0.2(H2 O)
Sum formula	C26 H29.40 F6 N6 O0.20 P	C26 H29.40 F6 N6 O0.20 P
Mr	574.13	574.12
Dx,g cm-3	1.419	1.419
Z	8	8
Mu (mm-1)	0.173	0.173
F000	2384.0	2384.0
F000'	2386.16	
h,k,lmax	30,16,21	30,16,21
Nref	11144	11142
Tmin,Tmax	0.969,0.977	0.686,0.739
Tmin'	0.961	

Correction method= # Reported T Limits: Tmin=0.686 Tmax=0.739
AbsCorr = MULTI-SCAN

Data completeness= 1.000 Theta(max)= 26.500

R(reflections)= 0.0916(8197) wR2(reflections)= 0.2313(11142)

S = 1.053 Npar= 900

The following ALERTS were generated. Each ALERT has the format
test-name_ALERT_alert-type_alert-level.
Click on the hyperlinks for more details of the test.

Alert level C

```
PLAT042_ALERT_1_C Calc. and Reported MoietyFormula Strings  Differ     Please Check
PLAT077_ALERT_4_C Unitcell Contains Non-integer Number of Atoms ..     Please Check
PLAT234_ALERT_4_C Large Hirshfeld Difference N1       --C1       .       0.21 Ang.
PLAT234_ALERT_4_C Large Hirshfeld Difference C20      --C21      .       0.17 Ang.
PLAT234_ALERT_4_C Large Hirshfeld Difference P1       --F1       .       0.16 Ang.
PLAT241_ALERT_2_C High   'MainMol' Ueq as Compared to Neighbors of        C15 Check
PLAT250_ALERT_2_C Large U3/U1 Ratio for Average U(i,j) Tensor ....        2.3 Note
PLAT250_ALERT_2_C Large U3/U1 Ratio for Average U(i,j) Tensor ....        2.8 Note
PLAT260_ALERT_2_C Large Average Ueq of Residue Including        P1      0.107 Check
PLAT260_ALERT_2_C Large Average Ueq of Residue Including        P4      0.101 Check
PLAT340_ALERT_3_C Low Bond Precision on  C-C Bonds ...............    0.00662 Ang.
PLAT360_ALERT_2_C Short  C(sp3)-C(sp3) Bond  C25      - C26      .       1.40 Ang.
PLAT906_ALERT_3_C Large K Value in the Analysis of Variance ......     11.298 Check
PLAT906_ALERT_3_C Large K Value in the Analysis of Variance ......      2.269 Check
```

Alert level G

```
PLAT002_ALERT_2_G Number of Distance or Angle Restraints on AtSite         33 Note
PLAT003_ALERT_2_G Number of Uiso or Uij Restrained non-H Atoms ...         21 Report
PLAT007_ALERT_5_G Number of Unrefined Donor-H Atoms .............           2 Report
PLAT083_ALERT_2_G SHELXL Second Parameter in WGHT  Unusually Large       9.77 Why ?
PLAT172_ALERT_4_G The CIF-Embedded .res File Contains DFIX Records          5 Report
PLAT176_ALERT_4_G The CIF-Embedded .res File Contains SADI Records         12 Report
PLAT178_ALERT_4_G The CIF-Embedded .res File Contains SIMU Records          4 Report
PLAT187_ALERT_4_G The CIF-Embedded .res File Contains RIGU Records          6 Report
PLAT191_ALERT_3_G A Non-default SADI Restraint Value has been used     0.0500 Report
PLAT191_ALERT_3_G A Non-default SADI Restraint Value has been used     0.0400 Report
PLAT191_ALERT_3_G A Non-default SADI Restraint Value has been used     0.0400 Report
PLAT191_ALERT_3_G A Non-default SADI Restraint Value has been used     0.0500 Report
PLAT191_ALERT_3_G A Non-default SADI Restraint Value has been used     0.0500 Report
PLAT191_ALERT_3_G A Non-default SADI Restraint Value has been used     0.0400 Report
PLAT191_ALERT_3_G A Non-default SADI Restraint Value has been used     0.0400 Report
PLAT191_ALERT_3_G A Non-default SADI Restraint Value has been used     0.0500 Report
PLAT300_ALERT_4_G Atom Site Occupancy of C1         Constrained at        0.5 Check
PLAT300_ALERT_4_G Atom Site Occupancy of C1B        Constrained at        0.5 Check
PLAT300_ALERT_4_G Atom Site Occupancy of C2         Constrained at        0.5 Check
PLAT300_ALERT_4_G Atom Site Occupancy of C2B        Constrained at        0.5 Check
PLAT300_ALERT_4_G Atom Site Occupancy of C3         Constrained at        0.5 Check
PLAT300_ALERT_4_G Atom Site Occupancy of C3B        Constrained at        0.5 Check
PLAT300_ALERT_4_G Atom Site Occupancy of C4         Constrained at        0.5 Check
PLAT300_ALERT_4_G Atom Site Occupancy of C4B        Constrained at        0.5 Check
PLAT300_ALERT_4_G Atom Site Occupancy of C5         Constrained at        0.5 Check
PLAT300_ALERT_4_G Atom Site Occupancy of C5B        Constrained at        0.5 Check
PLAT300_ALERT_4_G Atom Site Occupancy of C6         Constrained at        0.5 Check
PLAT300_ALERT_4_G Atom Site Occupancy of C6B        Constrained at        0.5 Check
PLAT300_ALERT_4_G Atom Site Occupancy of H3         Constrained at        0.5 Check
PLAT300_ALERT_4_G Atom Site Occupancy of H3B        Constrained at        0.5 Check
PLAT300_ALERT_4_G Atom Site Occupancy of H4         Constrained at        0.5 Check
PLAT300_ALERT_4_G Atom Site Occupancy of H4B        Constrained at        0.5 Check
PLAT300_ALERT_4_G Atom Site Occupancy of H5         Constrained at        0.5 Check
```

```
PLAT300_ALERT_4_G Atom Site Occupancy of H5B       Constrained at        0.5 Check
PLAT300_ALERT_4_G Atom Site Occupancy of H6        Constrained at        0.5 Check
PLAT300_ALERT_4_G Atom Site Occupancy of H6B       Constrained at        0.5 Check
PLAT300_ALERT_4_G Atom Site Occupancy of P1        Constrained at        0.6 Check
PLAT300_ALERT_4_G Atom Site Occupancy of F1        Constrained at        0.6 Check
PLAT300_ALERT_4_G Atom Site Occupancy of F2        Constrained at        0.6 Check
PLAT300_ALERT_4_G Atom Site Occupancy of F3        Constrained at        0.6 Check
PLAT300_ALERT_4_G Atom Site Occupancy of F4        Constrained at        0.6 Check
PLAT300_ALERT_4_G Atom Site Occupancy of F5        Constrained at        0.6 Check
PLAT300_ALERT_4_G Atom Site Occupancy of F6        Constrained at        0.6 Check
PLAT300_ALERT_4_G Atom Site Occupancy of P3        Constrained at        0.6 Check
PLAT300_ALERT_4_G Atom Site Occupancy of F13       Constrained at        0.6 Check
PLAT300_ALERT_4_G Atom Site Occupancy of F14       Constrained at        0.6 Check
PLAT300_ALERT_4_G Atom Site Occupancy of F15       Constrained at        0.6 Check
PLAT300_ALERT_4_G Atom Site Occupancy of F16       Constrained at        0.6 Check
PLAT300_ALERT_4_G Atom Site Occupancy of F17       Constrained at        0.6 Check
PLAT300_ALERT_4_G Atom Site Occupancy of F18       Constrained at        0.6 Check
PLAT300_ALERT_4_G Atom Site Occupancy of P2        Constrained at        0.4 Check
PLAT300_ALERT_4_G Atom Site Occupancy of F7        Constrained at        0.4 Check
PLAT300_ALERT_4_G Atom Site Occupancy of F8        Constrained at        0.4 Check
PLAT300_ALERT_4_G Atom Site Occupancy of F9        Constrained at        0.4 Check
PLAT300_ALERT_4_G Atom Site Occupancy of F10       Constrained at        0.4 Check
PLAT300_ALERT_4_G Atom Site Occupancy of F11       Constrained at        0.4 Check
PLAT300_ALERT_4_G Atom Site Occupancy of F12       Constrained at        0.4 Check
PLAT300_ALERT_4_G Atom Site Occupancy of P4        Constrained at        0.4 Check
PLAT300_ALERT_4_G Atom Site Occupancy of F19       Constrained at        0.4 Check
PLAT300_ALERT_4_G Atom Site Occupancy of F20       Constrained at        0.4 Check
PLAT300_ALERT_4_G Atom Site Occupancy of F21       Constrained at        0.4 Check
PLAT300_ALERT_4_G Atom Site Occupancy of F22       Constrained at        0.4 Check
PLAT300_ALERT_4_G Atom Site Occupancy of F23       Constrained at        0.4 Check
PLAT300_ALERT_4_G Atom Site Occupancy of F24       Constrained at        0.4 Check
PLAT300_ALERT_4_G Atom Site Occupancy of O1        Constrained at        0.4 Check
PLAT300_ALERT_4_G Atom Site Occupancy of H1A       Constrained at        0.4 Check
PLAT300_ALERT_4_G Atom Site Occupancy of H1B       Constrained at        0.4 Check
PLAT301_ALERT_3_G Main Residue  Disorder .............(Resd  1  )        19% Note
PLAT302_ALERT_4_G Anion/Solvent/Minor-Residue Disorder (Resd  3  )       100% Note
PLAT302_ALERT_4_G Anion/Solvent/Minor-Residue Disorder (Resd  4  )       100% Note
PLAT302_ALERT_4_G Anion/Solvent/Minor-Residue Disorder (Resd  5  )       100% Note
PLAT302_ALERT_4_G Anion/Solvent/Minor-Residue Disorder (Resd  6  )       100% Note
PLAT302_ALERT_4_G Anion/Solvent/Minor-Residue Disorder (Resd  7  )       100% Note
PLAT304_ALERT_4_G Non-Integer Number of Atoms in ..... (Resd  3  )       4.20 Check
PLAT304_ALERT_4_G Non-Integer Number of Atoms in ..... (Resd  4  )       4.20 Check
PLAT304_ALERT_4_G Non-Integer Number of Atoms in ..... (Resd  5  )       2.80 Check
PLAT304_ALERT_4_G Non-Integer Number of Atoms in ..... (Resd  6  )       2.80 Check
PLAT304_ALERT_4_G Non-Integer Number of Atoms in ..... (Resd  7  )       1.20 Check
PLAT432_ALERT_2_G Short Inter X...Y Contact  F9       ..C37      .       2.89 Ang.
                                         1-x,1-y,-z  =      3_665 Check
PLAT432_ALERT_2_G Short Inter X...Y Contact  F24      ..C14      .       2.90 Ang.
                                      x,1/2-y,1/2+z  =      4_566 Check
PLAT432_ALERT_2_G Short Inter X...Y Contact  O1       ..C6B      .       2.75 Ang.
                                              x,y,z  =      1_555 Check
PLAT720_ALERT_4_G Number of Unusual/Non-Standard Labels .........          1 Note
PLAT790_ALERT_4_G Centre of Gravity not Within Unit Cell: Resd.  #         5 Note
              F6 P
PLAT811_ALERT_5_G No ADDSYM Analysis: Too Many Excluded Atoms ....         ! Info
PLAT860_ALERT_3_G Number of Least-Squares Restraints ............        560 Note
PLAT910_ALERT_3_G Missing # of FCF Reflection(s) Below Theta(Min).         3 Note
```

```
PLAT967_ALERT_5_G Note: Two-Theta Cutoff Value in Embedded .res ..       53.0 Degree
PLAT978_ALERT_2_G Number C-C Bonds with Positive Residual Density.          1 Info
```

```
   0 ALERT level A = Most likely a serious problem - resolve or explain
   0 ALERT level B = A potentially serious problem, consider carefully
  14 ALERT level C = Check. Ensure it is not caused by an omission or oversight
  88 ALERT level G = General information/check it is not something unexpected

   1 ALERT type 1 CIF construction/syntax error, inconsistent or missing data
  13 ALERT type 2 Indicator that the structure model may be wrong or deficient
  14 ALERT type 3 Indicator that the structure quality may be low
  71 ALERT type 4 Improvement, methodology, query or suggestion
   3 ALERT type 5 Informative message, check
```

It is advisable to attempt to resolve as many as possible of the alerts in all categories. Often the minor alerts point to easily fixed oversights, errors and omissions in your CIF or refinement strategy, so attention to these fine details can be worthwhile. In order to resolve some of the more serious problems it may be necessary to carry out additional measurements or structure refinements. However, the purpose of your study may justify the reported deviations and the more serious of these should normally be commented upon in the discussion or experimental section of a paper or in the "special_details" fields of the CIF. checkCIF was carefully designed to identify outliers and unusual parameters, but every test has its limitations and alerts that are not important in a particular case may appear. Conversely, the absence of alerts does not guarantee there are no aspects of the results needing attention. It is up to the individual to critically assess their own results and, if necessary, seek expert advice.

Publication of your CIF in IUCr journals

A basic structural check has been run on your CIF. These basic checks will be run on all CIFs submitted for publication in IUCr journals (*Acta Crystallographica*, *Journal of Applied Crystallography*, *Journal of Synchrotron Radiation*); however, if you intend to submit to *Acta Crystallographica Section C* or *E* or *IUCrData*, you should make sure that full publication checks are run on the final version of your CIF prior to submission.

Publication of your CIF in other journals

Please refer to the *Notes for Authors* of the relevant journal for any special instructions relating to CIF submission.

PLATON version of 06/07/2023; check.def file version of 30/06/2023

Datablock mo_2023_ee81_2_0ma - ellipsoid plot

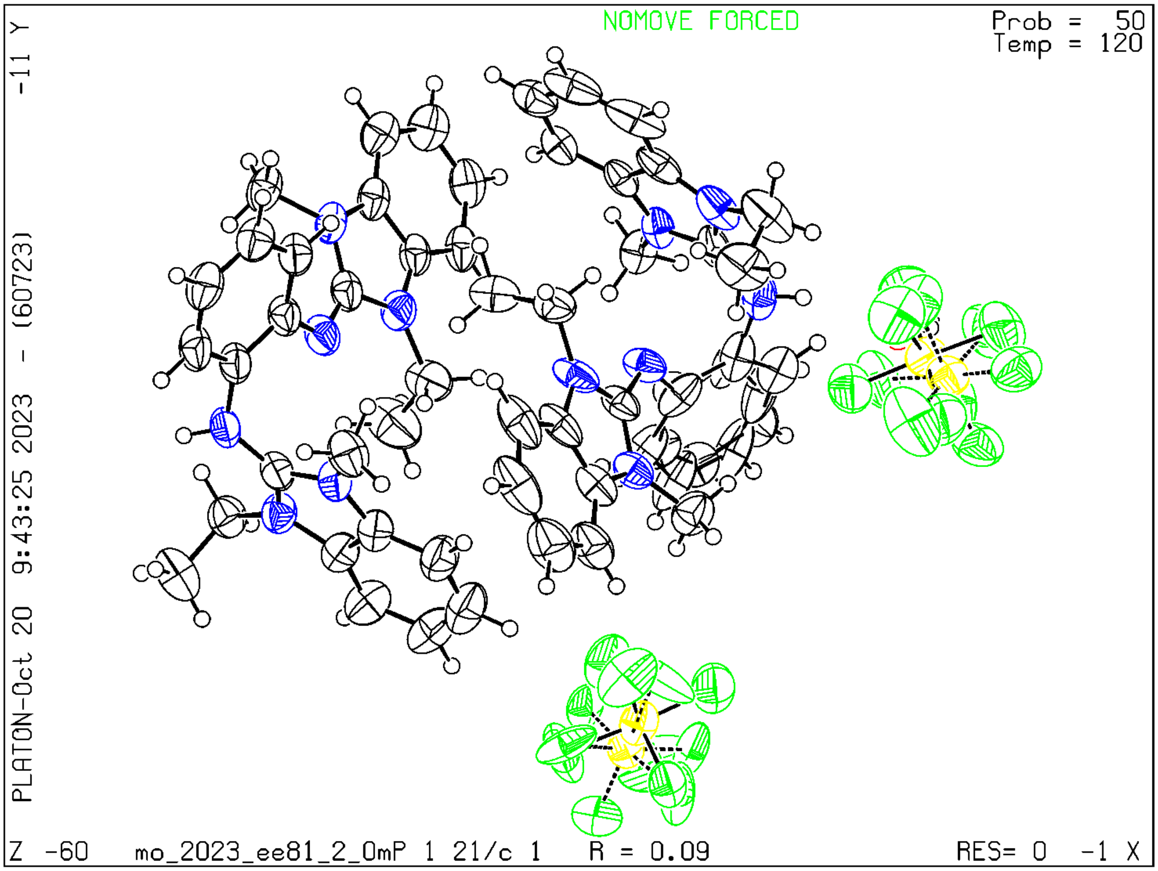

Supplement: Supplementary file 2 — Supporting Information [file CHEM-31-e02457-s002.zip › mo_2023_ee81_2_0ma_cifreport.pdf]
