# Supplementary material for: Steric Restraints in Redox‐Active Guanidine Ligands and Their Impact on Coordination Chemistry
Source: Chemistry. 2025 Oct 25;31(66):e02457. doi: 10.1002/chem.202502457 (PMC12648461; doi:10.1002/chem.202502457)
Supplement: Supplementary file 2 — Supporting Information [file CHEM-31-e02457-s002.zip › checkCIF_ee84.pdf]

checkCIF (basic structural check) running

Checking for embedded fcf data in CIF ...
Found embedded fcf data in CIF. Extracting fcf data from uploaded CIF, please wait . . . .

checkCIF/PLATON (basic structural check)

Structure factors have been supplied for datablock(s) mo_2023_ee84_0ma

THIS REPORT IS FOR GUIDANCE ONLY. IF USED AS PART OF A REVIEW PROCEDURE FOR PUBLICATION, IT SHOULD NOT REPLACE THE EXPERTISE OF AN EXPERIENCED CRYSTALLOGRAPHIC REFEREE.

No syntax errors found. CIF dictionary
Please wait while processing .... Interpreting this report

Structure factor report

Datablock: mo_2023_ee84_0ma

Bond precision:	C-C = 0.0043 A		Wavelength=0.71073
Cell:	a=17.3272(16)	b=31.536(3)	c=18.7767(15)
	alpha=90	beta=103.830(3)	gamma=90
Temperature:	120 K		

	Calculated	Reported
Volume	9962.7(15)	9962.7(15)
Space group	P 21/c	P 1 21/c 1
Hall group	-P 2ybc	-P 2ybc
Moiety formula	C48 H48 Cu N12, 2(B F4), C2 H3 N [+ solvent]	C48 H48 Cu N12, 2(B F4), C2 H3 N, [+ solventS]0.5[CH3CN]
Sum formula	C50 H51 B2 Cu F8 N13 [+ solvent]	C51 H52.50 B2 Cu F8 N13.50
Mr	1071.21	1091.72
Dx,g cm-3	1.428	1.456
Z	8	8
Mu (mm-1)	0.519	0.521
F000	4424.0	4512.0
F000'	4428.56	
h,k,lmax	22,40,24	22,40,24
Nref	22891	22883
Tmin,Tmax	0.894,0.925	0.688,0.746
Tmin'	0.851	

Correction method= # Reported T Limits: Tmin=0.688 Tmax=0.746 AbsCorr = MULTI-SCAN

Data completeness= 1.000 Theta(max)= 27.500

R(reflections)= 0.0570(20026) wR2(reflections)= 0.1615(22883)

S = 1.034 Npar= 1398

The following ALERTS were generated. Each ALERT has the format
test-name_ALERT_alert-type_alert-level.
Click on the hyperlinks for more details of the test.

Alert level B

PLAT434_ALERT_2_B Short Inter HL..HL Contact F10 ..F12 . 2.46 Ang.
-x,1-y,-z = 3_565 Check

Author Response: Due to unresolvable BF4 disorder

PLAT434_ALERT_2_B Short Inter HL..HL Contact F12 ..F12 . 2.39 Ang.
-x,1-y,-z = 3_565 Check

Alert level C

PLAT213_ALERT_2_C Atom C69 has ADP max/min Ratio ..... 4.0 prolat
And 2 other PLAT213 Alerts
More ...

PLAT220_ALERT_2_C NonSolvent Resd 2 C Ueq(max)/Ueq(min) Range 4.9 Ratio
PLAT243_ALERT_4_C High 'Solvent' Ueq as Compared to Neighbors of N63 Check
PLAT244_ALERT_4_C Low 'Solvent' Ueq as Compared to Neighbors of B4 Check
PLAT244_ALERT_4_C Low 'Solvent' Ueq as Compared to Neighbors of C49 Check
PLAT250_ALERT_2_C Large U3/U1 Ratio for Average U(i,j) Tensor .... 2.6 Note
PLAT260_ALERT_2_C Large Average Ueq of Residue Including F13 0.105 Check
PLAT260_ALERT_2_C Large Average Ueq of Residue Including F9 0.179 Check
PLAT910_ALERT_3_C Missing # of FCF Reflection(s) Below Theta(Min). 8 Note
PLAT918_ALERT_3_C Reflection(s) with I(obs) much Smaller I(calc) . 5 Check
PLAT971_ALERT_2_C Check Calcd Resid. Dens. 1.13Ang From F9 1.81 eA-3
PLAT971_ALERT_2_C Check Calcd Resid. Dens. 1.76Ang From F9 1.59 eA-3
PLAT977_ALERT_2_C Check Negative Difference Density on H71 . -0.39 eA-3

Alert level G

FORMU01_ALERT_1_G There is a discrepancy between the atom counts in the
_chemical_formula_sum and _chemical_formula_moiety. This is
usually due to the moiety formula being in the wrong format.
Atom count from _chemical_formula_sum: C51 H52.5 B2 Cu1 F8 N13.5
Atom count from _chemical_formula_moiety:C50 H51 B2 Cu1 F8 N13
FORMU01_ALERT_2_G There is a discrepancy between the atom counts in the
_chemical_formula_sum and the formula from the _atom_site* data.
Atom count from _chemical_formula_sum:C51 H52.5 B2 Cu1 F8 N13.5
Atom count from the _atom_site data: C50 H51 B2 Cu1 F8 N13
CELLZ01_ALERT_1_G Difference between formula and atom_site contents detected.
CELLZ01_ALERT_1_G ALERT: Large difference may be due to a
symmetry error - see SYMMG tests
From the CIF: _cell_formula_units_Z 8
From the CIF: _chemical_formula_sum C51 H52.50 B2 Cu F8 N13.50
TEST: Compare cell contents of formula and atom_site data

atom	Z*formula	cif sites	diff
C	408.00	400.00	8.00
H	420.00	408.00	12.00
B	16.00	16.00	0.00
Cu	8.00	8.00	0.00
F	64.00	64.00	0.00
N	108.00	104.00	4.00

PLAT002_ALERT_2_G Number of Distance or Angle Restraints on AtSite 12 Note
PLAT003_ALERT_2_G Number of Uiso or Uij Restrained non-H Atoms ... 9 Report
PLAT041_ALERT_1_G Calc. and Reported SumFormula Strings Differ Please Check
PLAT083_ALERT_2_G SHELXL Second Parameter in WGHT Unusually Large 20.75 Why ?
PLAT172_ALERT_4_G The CIF-Embedded .res File Contains DFIX Records 2 Report
PLAT176_ALERT_4_G The CIF-Embedded .res File Contains SADI Records 5 Report
PLAT178_ALERT_4_G The CIF-Embedded .res File Contains SIMU Records 4 Report
PLAT187_ALERT_4_G The CIF-Embedded .res File Contains RIGU Records 4 Report
PLAT191_ALERT_3_G A Non-default SADI Restraint Value has been used 0.0400 Report
And 2 other PLAT191 Alerts
More ...
PLAT231_ALERT_4_G Hirshfeld Test (Solvent) F16 --B4 . 6.4 s.u.
And 2 other PLAT231 Alerts
More ...
PLAT244_ALERT_4_G Low 'Solvent' Ueq as Compared to Neighbors of B1 Check
And 2 other PLAT244 Alerts
More ...
PLAT300_ALERT_4_G Atom Site Occupancy of C1A Constrained at 0.5 Check
And 9 other PLAT300 Alerts
More ...
PLAT302_ALERT_4_G Anion/Solvent/Minor-Residue Disorder (Resd 3) 60% Note
PLAT302_ALERT_4_G Anion/Solvent/Minor-Residue Disorder (Resd 7) 67% Note
PLAT432_ALERT_2_G Short Inter X...Y Contact F3 ..C13 . 2.94 Ang.
x,3/2-y,-1/2+z = 4_575 Check
PLAT605_ALERT_4_G Largest Solvent Accessible VOID in the Structure 76 A**3
PLAT720_ALERT_4_G Number of Unusual/Non-Standard Labels .... 3 Note
PLAT790_ALERT_4_G Centre of Gravity not Within Unit Cell: Resd. # 3 Note
B F4
PLAT790_ALERT_4_G Centre of Gravity not Within Unit Cell: Resd. # 7 Note
C2 H3 N
PLAT860_ALERT_3_G Number of Least-Squares Restraints .... 133 Note
PLAT868_ALERT_4_G ALERTS Due to the Use of _smtbx_masks Suppressed ! Info
PLAT967_ALERT_5_G Note: Two-Theta Cutoff Value in Embedded .res .. 55.0 Degree
PLAT978_ALERT_2_G Number C-C Bonds with Positive Residual Density. 1 Info

0 **ALERT level A** = Most likely a serious problem - resolve or explain
2 **ALERT level B** = A potentially serious problem, consider carefully
15 **ALERT level C** = Check. Ensure it is not caused by an omission or oversight

42 **ALERT level G** = General information/check it is not something unexpected

4 ALERT type 1 CIF construction/syntax error, inconsistent or missing data
18 ALERT type 2 Indicator that the structure model may be wrong or deficient
6 ALERT type 3 Indicator that the structure quality may be low
30 ALERT type 4 Improvement, methodology, query or suggestion
1 ALERT type 5 Informative message, check

It is advisable to attempt to resolve as many as possible of the alerts in all categories. Often the minor alerts point to easily fixed oversights, errors and omissions in your CIF or refinement strategy, so attention to these fine details can be worthwhile. In order to resolve some of the more serious problems it may be necessary to carry out additional measurements or structure refinements. However, the purpose of your study may justify the reported deviations and the more serious of these should normally be commented upon in the discussion or experimental section of a paper or in the "special_details" fields of the CIF. checkCIF was carefully designed to identify outliers and unusual parameters, but every test has its limitations and alerts that are not important in a particular case may appear. Conversely, the absence of alerts does not guarantee there are no aspects of the results needing attention. It is up to the individual to critically assess their own results and, if necessary, seek expert advice.

Publication of your CIF in IUCr journals

A basic structural check has been run on your CIF. These basic checks will be run on all CIFs submitted for publication in IUCr journals (*Acta Crystallographica*, *Journal of Applied Crystallography*, *Journal of Synchrotron Radiation*); however, if you intend to submit to *Acta Crystallographica Section C* or *E* or *IUCrData*, you should make sure that full publication checks are run on the final version of your CIF prior to submission.

Publication of your CIF in other journals

Please refer to the *Notes for Authors* of the relevant journal for any special instructions relating to CIF submission.

PLATON version of 06/07/2023; check.def file version of 30/06/2023

Datablock mo_2023_ee84_0ma - ellipsoid plot

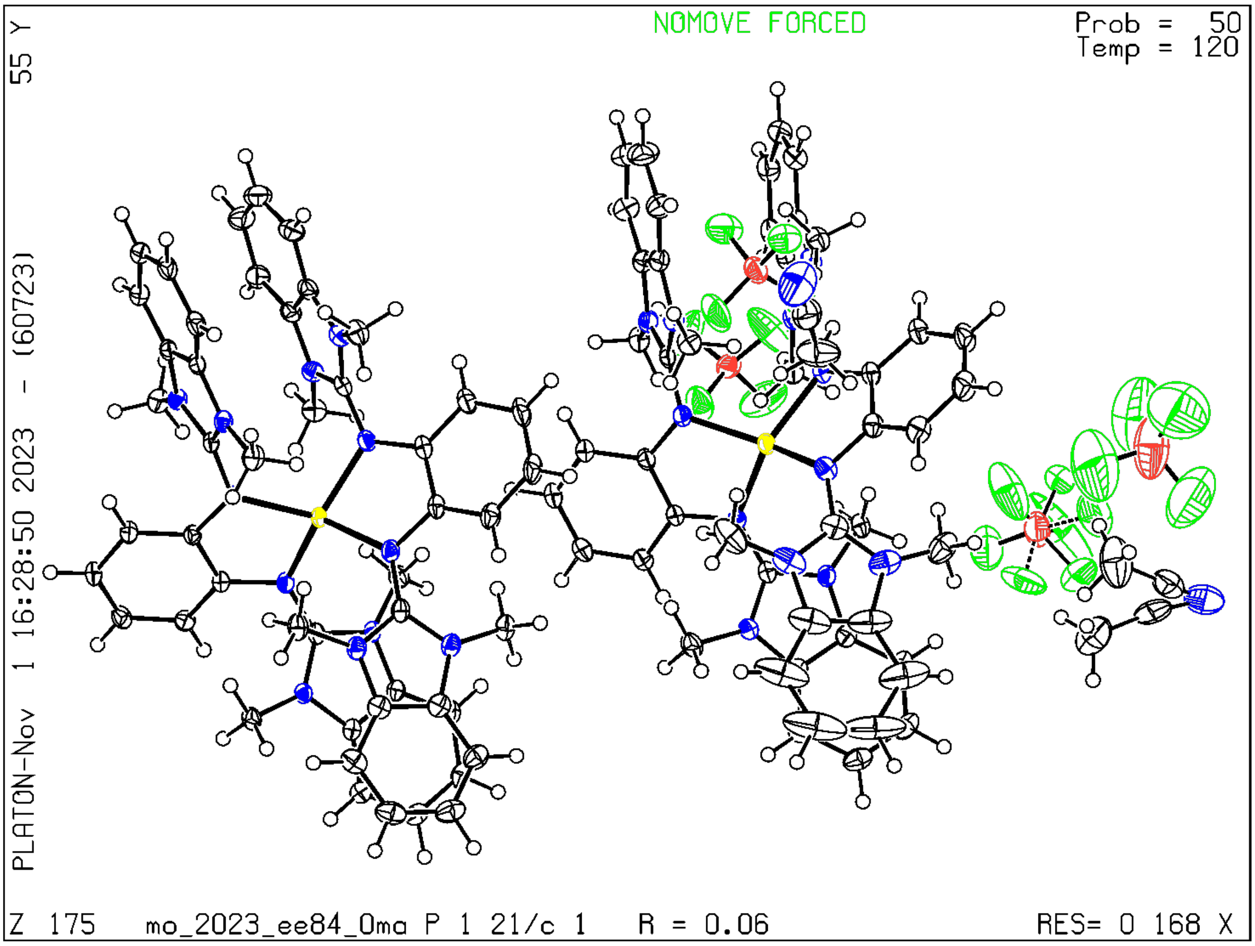

Download CIF editor (publCIF) from the IUCr
Download CIF editor (enCIFer) from the CCDC
Test a new CIF entry
