# Supplementary material for: Steric Restraints in Redox‐Active Guanidine Ligands and Their Impact on Coordination Chemistry
Source: Chemistry. 2025 Oct 25;31(66):e02457. doi: 10.1002/chem.202502457 (PMC12648461; doi:10.1002/chem.202502457)

checkCIF/PLATON report

Structure factors have been supplied for datablock(s) mo_2023_ee90_2_0ma

THIS REPORT IS FOR GUIDANCE ONLY. IF USED AS PART OF A REVIEW PROCEDURE FOR PUBLICATION, IT SHOULD NOT REPLACE THE EXPERTISE OF AN EXPERIENCED CRYSTALLOGRAPHIC REFEREE.

No syntax errors found. CIF dictionary Interpreting this report

Datablock: mo_2023_ee90_2_0ma

Bond precision:	C-C = 0.0067 A		Wavelength=0.71073
Cell:	a=24.5373(13)	b=10.6239(7)	c=23.6441(15)
	alpha=90	beta=109.682(2)	gamma=90
Temperature:	120 K		

	Calculated	Reported
Volume	5803.5(6)	5803.5(6)
Space group	P 21/c	P 1 21/c 1
Hall group	-P 2ybc	-P 2ybc
Moiety formula	2(C25 H24 Br2 Co N6), 3(C H2 Cl2)	C25 H24 Br2 Co N6, 1.5(C H2 Cl2)
Sum formula	C53 H54 Br4 Cl6 Co2 N12	C26.50 H27 Br2 Cl3 Co N6
Mr	1509.24	754.64
Dx,g cm-3	1.727	1.727
Z	4	8
Mu (mm-1)	3.651	3.651
F000	3008.0	3008.0
F000'	3010.52	
h,k,lmax	31,13,30	31,13,30
Nref	12682	12667
Tmin,Tmax	0.482,0.631	0.609,0.746
Tmin'	0.412	

Correction method= # Reported T Limits: Tmin=0.609 Tmax=0.746
AbsCorr = MULTI-SCAN

Data completeness= 0.999 Theta(max)= 27.000

R(reflections)= 0.0507(10559) wR2(reflections)= 0.1216(12667)

S = 1.170 Npar= 716

The following ALERTS were generated. Each ALERT has the format
test-name_ALERT_alert-type_alert-level.
Click on the hyperlinks for more details of the test.

Alert level C

```
PLAT042_ALERT_1_C Calc. and Reported MoietyFormula Strings  Differ     Please Check
PLAT244_ALERT_4_C Low    'Solvent' Ueq as Compared to Neighbors of        C27 Check
PLAT244_ALERT_4_C Low    'Solvent' Ueq as Compared to Neighbors of        C26 Check
PLAT244_ALERT_4_C Low    'Solvent' Ueq as Compared to Neighbors of        C28 Check
PLAT341_ALERT_3_C Low Bond Precision on  C-C Bonds ...............     0.0067 Ang.
PLAT906_ALERT_3_C Large K Value in the Analysis of Variance ......      3.283 Check
PLAT911_ALERT_3_C Missing FCF Refl Between Thmin & STh/L=    0.600         14 Report
PLAT971_ALERT_2_C Check Calcd Resid. Dens.  1.20Ang From Cl5             1.68 eA-3
PLAT977_ALERT_2_C Check Negative Difference Density on H28B      .      -0.33 eA-3
```

Alert level G

```
PLAT002_ALERT_2_G Number of Distance or Angle Restraints on AtSite          5 Note
PLAT003_ALERT_2_G Number of Uiso or Uij Restrained non-H Atoms ...          4 Report
PLAT045_ALERT_1_G Calculated and Reported Z Differ by a Factor ...      0.500 Check
PLAT083_ALERT_2_G SHELXL Second Parameter in WGHT  Unusually Large      34.38 Why ?
PLAT176_ALERT_4_G The CIF-Embedded .res File Contains SADI Records          1 Report
PLAT178_ALERT_4_G The CIF-Embedded .res File Contains SIMU Records          1 Report
PLAT300_ALERT_4_G Atom Site Occupancy of Cl3        Constrained at        0.8 Check
PLAT300_ALERT_4_G Atom Site Occupancy of Cl4B       Constrained at        0.8 Check
PLAT300_ALERT_4_G Atom Site Occupancy of Cl4        Constrained at        0.2 Check
PLAT300_ALERT_4_G Atom Site Occupancy of Cl7        Constrained at        0.2 Check
PLAT300_ALERT_4_G Atom Site Occupancy of H27C       Constrained at        0.8 Check
PLAT300_ALERT_4_G Atom Site Occupancy of H27D       Constrained at        0.8 Check
PLAT300_ALERT_4_G Atom Site Occupancy of H27A       Constrained at        0.2 Check
PLAT300_ALERT_4_G Atom Site Occupancy of H27B       Constrained at        0.2 Check
PLAT302_ALERT_4_G Anion/Solvent/Minor-Residue Disorder (Resd  3  )        67% Note
PLAT434_ALERT_2_G Short Inter HL..HL Contact Cl5      ..Cl7      .       3.28 Ang.
                                                 x,-1+y,z  =      1_545 Check
PLAT790_ALERT_4_G Centre of Gravity not Within Unit Cell: Resd.  #          2 Note
              C25 H24 Br2 Co N6
PLAT790_ALERT_4_G Centre of Gravity not Within Unit Cell: Resd.  #          5 Note
              C H2 Cl2
PLAT860_ALERT_3_G Number of Least-Squares Restraints ............          18 Note
PLAT883_ALERT_1_G No Info/Value for _atom_sites_solution_primary .     Please Do !
PLAT910_ALERT_3_G Missing # of FCF Reflection(s) Below Theta(Min).          1 Note
PLAT913_ALERT_3_G Missing # of Very Strong Reflections in FCF ....          3 Note
PLAT933_ALERT_2_G Number of HKL-OMIT Records in Embedded .res File          4 Note
PLAT967_ALERT_5_G Note: Two-Theta Cutoff Value in Embedded .res ..       54.0 Degree
PLAT978_ALERT_2_G Number C-C Bonds with Positive Residual Density.          2 Info
```

0 **ALERT level A** = Most likely a serious problem - resolve or explain
0 **ALERT level B** = A potentially serious problem, consider carefully
9 **ALERT level C** = Check. Ensure it is not caused by an omission or oversight
25 **ALERT level G** = General information/check it is not something unexpected

3 ALERT type 1 CIF construction/syntax error, inconsistent or missing data

```
 8 ALERT type 2 Indicator that the structure model may be wrong or deficient
 6 ALERT type 3 Indicator that the structure quality may be low
16 ALERT type 4 Improvement, methodology, query or suggestion
 1 ALERT type 5 Informative message, check
```

It is advisable to attempt to resolve as many as possible of the alerts in all categories. Often the minor alerts point to easily fixed oversights, errors and omissions in your CIF or refinement strategy, so attention to these fine details can be worthwhile. In order to resolve some of the more serious problems it may be necessary to carry out additional measurements or structure refinements. However, the purpose of your study may justify the reported deviations and the more serious of these should normally be commented upon in the discussion or experimental section of a paper or in the "special_details" fields of the CIF. checkCIF was carefully designed to identify outliers and unusual parameters, but every test has its limitations and alerts that are not important in a particular case may appear. Conversely, the absence of alerts does not guarantee there are no aspects of the results needing attention. It is up to the individual to critically assess their own results and, if necessary, seek expert advice.

Publication of your CIF in IUCr journals

A basic structural check has been run on your CIF. These basic checks will be run on all CIFs submitted for publication in IUCr journals (*Acta Crystallographica*, *Journal of Applied Crystallography*, *Journal of Synchrotron Radiation*); however, if you intend to submit to *Acta Crystallographica Section C* or *E* or *IUCrData*, you should make sure that full publication checks are run on the final version of your CIF prior to submission.

Publication of your CIF in other journals

Please refer to the *Notes for Authors* of the relevant journal for any special instructions relating to CIF submission.

PLATON version of 06/07/2023; check.def file version of 30/06/2023

Datablock mo_2023_ee90_2_0ma - ellipsoid plot

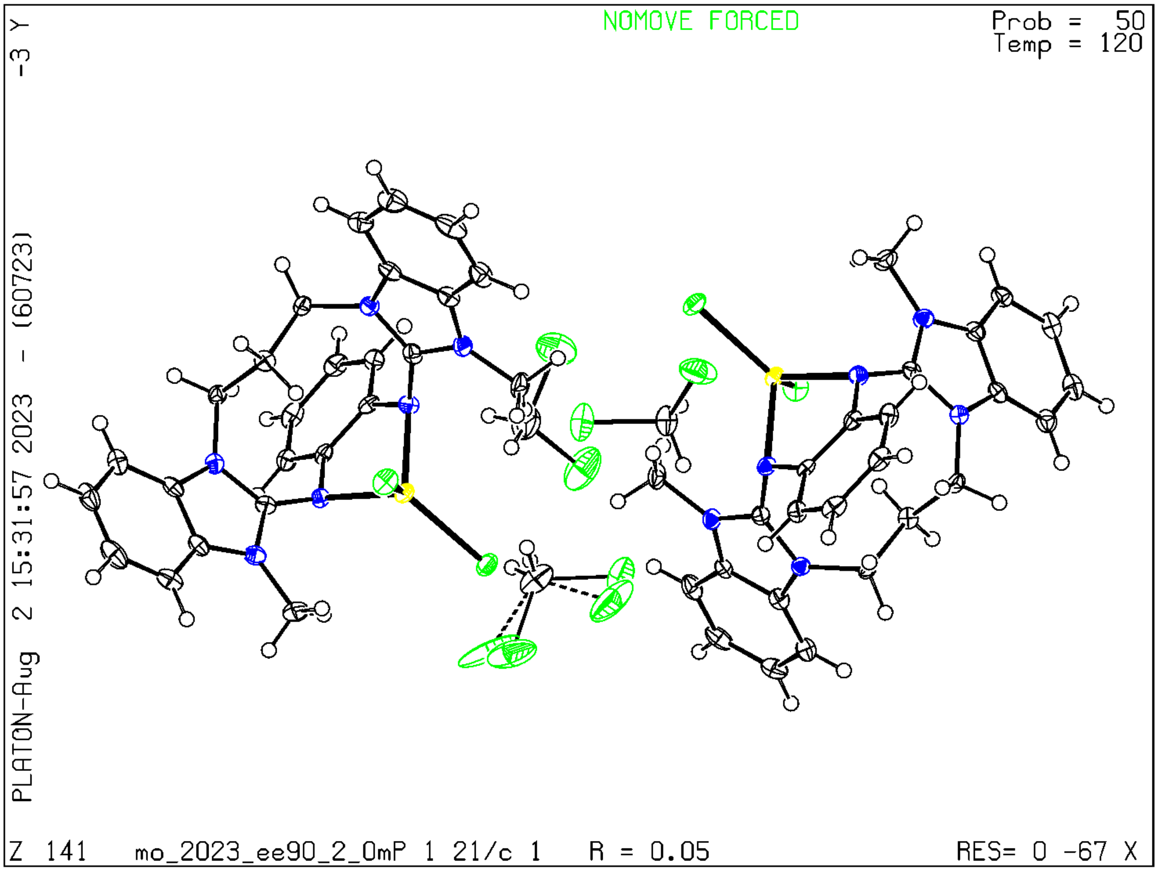

Supplement: Supplementary file 2 — Supporting Information [file CHEM-31-e02457-s002.zip › mo_2023_ee90_2_0ma_cifreport.pdf]
