# Supplementary material for: Steric Restraints in Redox‐Active Guanidine Ligands and Their Impact on Coordination Chemistry
Source: Chemistry. 2025 Oct 25;31(66):e02457. doi: 10.1002/chem.202502457 (PMC12648461; doi:10.1002/chem.202502457)

checkCIF/PLATON report

Structure factors have been supplied for datablock(s) mo_2023_ee96_0

THIS REPORT IS FOR GUIDANCE ONLY. IF USED AS PART OF A REVIEW PROCEDURE FOR PUBLICATION, IT SHOULD NOT REPLACE THE EXPERTISE OF AN EXPERIENCED CRYSTALLOGRAPHIC REFEREE.

No syntax errors found. CIF dictionary Interpreting this report

Datablock: mo_2023_ee96_0

Bond precision:	C-C = 0.0041 A	Wavelength=0.71073	
Cell:	a=13.6824(9)	b=13.6824(9)	c=24.615(2)
	alpha=90	beta=90	gamma=120
Temperature:	120 K		

	Calculated	Reported
Volume	3990.8(6)	3990.8(6)
Space group	P -6 2 c	P -6 2 c
Hall group	P -6c -2c	P -6c -2c
Moiety formula	C78 H84 Cl5 N18 Ni3 [+ solvent]	C78 H84 Cl5 N18 Ni3, [+ solventS]0.9[C4H10O]
Sum formula	C78 H84 Cl5 N18 Ni3 [+ solvent]	C78 H84 Cl5 N18 Ni3
Mr	1626.95	1627.01
Dx,g cm-3	1.354	1.354
Z	2	2
Mu (mm-1)	0.922	0.922
F000	1694.0	1694.0
F000'	1697.88	
h,k,lmax	18,18,32	18,18,32
Nref	3296[1852]	3294
Tmin,Tmax	0.823,0.937	0.689,0.746
Tmin'	0.794	

Correction method= # Reported T Limits: Tmin=0.689 Tmax=0.746
AbsCorr = MULTI-SCAN

Data completeness= 1.78/1.00 Theta(max)= 27.999

R(reflections)= 0.0249(3203)

wR2(reflections)= 0.0647(3294)

S = 1.070 Npar= 163

The following ALERTS were generated. Each ALERT has the format
test-name_ALERT_alert-type_alert-level.
Click on the hyperlinks for more details of the test.

Alert level C

```
PLAT220_ALERT_2_C NonSolvent   Resd 1  C   Ueq(max)/Ueq(min) Range        3.2 Ratio
PLAT242_ALERT_2_C Low    'MainMol' Ueq as Compared to Neighbors of        C12 Check
PLAT911_ALERT_3_C Missing FCF Refl Between Thmin & STh/L=    0.600          2 Report
```

Alert level G

```
PLAT232_ALERT_2_G Hirshfeld Test Diff (M-X)  Ni1      --Cl2      .        5.5 s.u.
PLAT605_ALERT_4_G Largest Solvent Accessible VOID in the Structure        137 A**3
PLAT794_ALERT_5_G Tentative Bond Valency for Ni1       (II)      .       1.92 Info
PLAT868_ALERT_4_G ALERTS Due to the Use of _smtbx_masks Suppressed          ! Info
PLAT910_ALERT_3_G Missing # of FCF Reflection(s) Below Theta(Min).          3 Note
PLAT913_ALERT_3_G Missing # of Very Strong Reflections in FCF ....          2 Note
PLAT967_ALERT_5_G Note: Two-Theta Cutoff Value in Embedded .res ..       56.0 Degree
PLAT978_ALERT_2_G Number C-C Bonds with Positive Residual Density.          3 Info
```

---

0 **ALERT level A** = Most likely a serious problem - resolve or explain
0 **ALERT level B** = A potentially serious problem, consider carefully
3 **ALERT level C** = Check. Ensure it is not caused by an omission or oversight
8 **ALERT level G** = General information/check it is not something unexpected

0 ALERT type 1 CIF construction/syntax error, inconsistent or missing data
4 ALERT type 2 Indicator that the structure model may be wrong or deficient
3 ALERT type 3 Indicator that the structure quality may be low
2 ALERT type 4 Improvement, methodology, query or suggestion
2 ALERT type 5 Informative message, check

It is advisable to attempt to resolve as many as possible of the alerts in all categories. Often the minor alerts point to easily fixed oversights, errors and omissions in your CIF or refinement strategy, so attention to these fine details can be worthwhile. In order to resolve some of the more serious problems it may be necessary to carry out additional measurements or structure refinements. However, the purpose of your study may justify the reported deviations and the more serious of these should normally be commented upon in the discussion or experimental section of a paper or in the "special_details" fields of the CIF. checkCIF was carefully designed to identify outliers and unusual parameters, but every test has its limitations and alerts that are not important in a particular case may appear. Conversely, the absence of alerts does not guarantee there are no aspects of the results needing attention. It is up to the individual to critically assess their own results and, if necessary, seek expert advice.

Publication of your CIF in IUCr journals

A basic structural check has been run on your CIF. These basic checks will be run on all CIFs submitted for publication in IUCr journals (*Acta Crystallographica*, *Journal of Applied Crystallography*, *Journal of Synchrotron Radiation*); however, if you intend to submit to *Acta Crystallographica Section C* or *E* or *IUCrData*, you should make sure that full publication checks are run on the final version of your CIF prior to submission.

Publication of your CIF in other journals

Please refer to the *Notes for Authors* of the relevant journal for any special instructions relating to CIF submission.

PLATON version of 06/07/2023; check.def file version of 30/06/2023

Datablock mo_2023_ee96_0 - ellipsoid plot

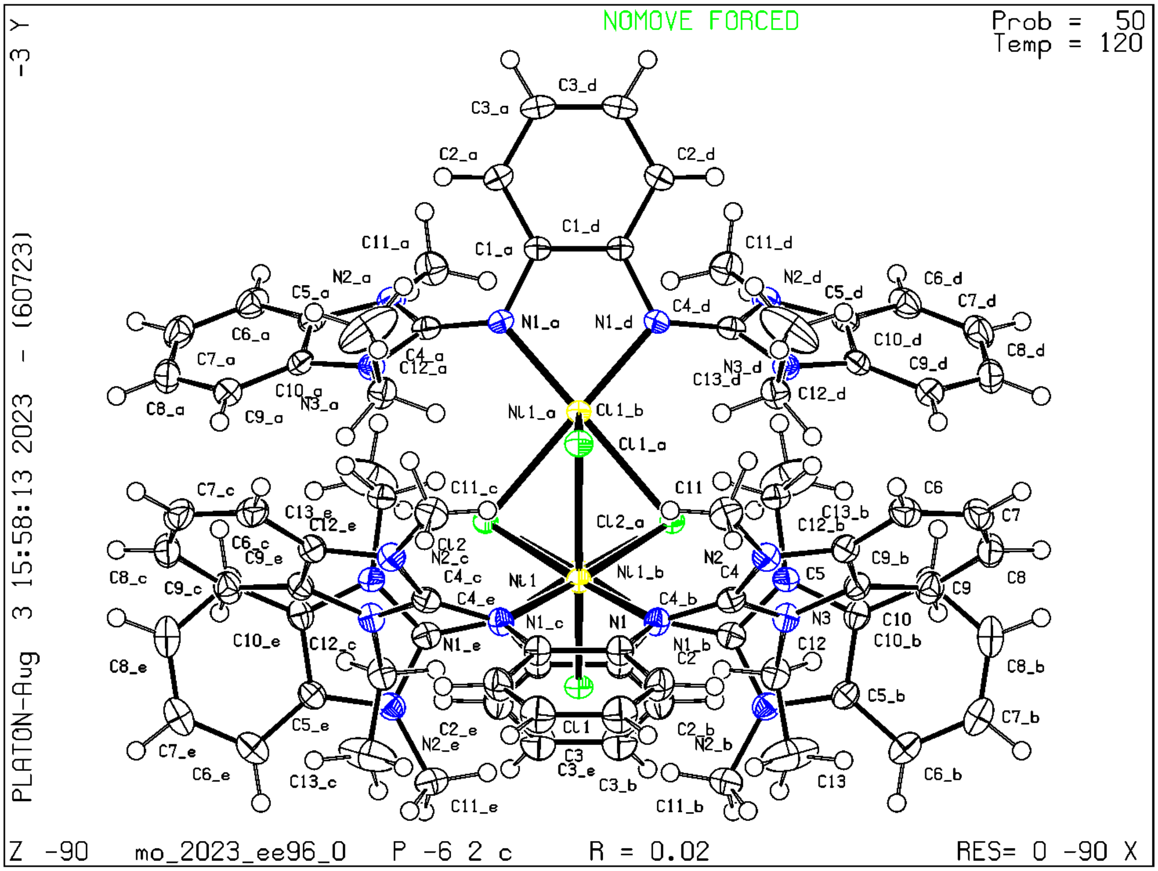

Supplement: Supplementary file 2 — Supporting Information [file CHEM-31-e02457-s002.zip › mo_2023_ee96_0_cifreport.pdf]
