# Supplementary material for: Steric Restraints in Redox‐Active Guanidine Ligands and Their Impact on Coordination Chemistry
Source: Chemistry. 2025 Oct 25;31(66):e02457. doi: 10.1002/chem.202502457 (PMC12648461; doi:10.1002/chem.202502457)

checkCIF/PLATON report

Structure factors have been supplied for datablock(s) mo_2024_ee97_1_0ma

THIS REPORT IS FOR GUIDANCE ONLY. IF USED AS PART OF A REVIEW PROCEDURE FOR PUBLICATION, IT SHOULD NOT REPLACE THE EXPERTISE OF AN EXPERIENCED CRYSTALLOGRAPHIC REFEREE.

No syntax errors found. CIF dictionary Interpreting this report

Datablock: mo_2024_ee97_1_0ma

Bond precision:	C-C = 0.0039 A		Wavelength=0.71073
Cell:	a=12.6181(14)	b=12.8586(13)	c=22.741(2)
	alpha=90	beta=105.286(4)	gamma=90
Temperature:	100 K		

	Calculated	Reported
Volume	3559.2(6)	3559.3(7)
Space group	P 21/c	P 1 21/c 1
Hall group	-P 2ybc	-P 2ybc
Moiety formula	C70 H52 Co2 F24 N12 O8	C70 H52 Co2 F24 N12 O8
Sum formula	C70 H52 Co2 F24 N12 O8	C70 H52 Co2 F24 N12 O8
Mr	1763.10	1763.09
Dx,g cm-3	1.645	1.645
Z	2	2
Mu (mm-1)	0.595	0.595
F000	1780.0	1780.0
F000'	1782.67	
h,k,lmax	15,16,28	15,16,28
Nref	7377	7376
Tmin,Tmax	0.898,0.942	0.657,0.746
Tmin'	0.898	

Correction method= # Reported T Limits: Tmin=0.657 Tmax=0.746
AbsCorr = MULTI-SCAN

Data completeness= 1.000 Theta(max)= 26.499

R(reflections)= 0.0412(5695) wR2(reflections)= 0.1020(7376)

S = 1.021 Npar= 543

The following ALERTS were generated. Each ALERT has the format
test-name_ALERT_alert-type_alert-level.
Click on the hyperlinks for more details of the test.

Alert level C

```
RINTA01_ALERT_3_C  The value of Rint is greater than 0.12
            Rint given   0.125
PLAT213_ALERT_2_C Atom F3B             has ADP max/min Ratio .....        4.0 prolat
PLAT242_ALERT_2_C Low    'MainMol' Ueq as Compared to Neighbors of        C34 Check
```

Alert level G

```
PLAT002_ALERT_2_G Number of Distance or Angle Restraints on AtSite          6 Note
PLAT020_ALERT_3_G The Value of Rint is Greater Than 0.12 .........      0.125 Report
PLAT083_ALERT_2_G SHELXL Second Parameter in WGHT  Unusually Large       6.30 Why ?
PLAT176_ALERT_4_G The CIF-Embedded .res File Contains SADI Records          1 Report
PLAT178_ALERT_4_G The CIF-Embedded .res File Contains SIMU Records          1 Report
PLAT187_ALERT_4_G The CIF-Embedded .res File Contains RIGU Records          1 Report
PLAT232_ALERT_2_G Hirshfeld Test Diff (M-X)  Co1      --O2       .        5.6 s.u.
PLAT232_ALERT_2_G Hirshfeld Test Diff (M-X)  Co1      --O3       .        5.4 s.u.
PLAT242_ALERT_2_G Low    'MainMol' Ueq as Compared to Neighbors of        C29 Check
PLAT242_ALERT_2_G Low    'MainMol' Ueq as Compared to Neighbors of        C30 Check
PLAT242_ALERT_2_G Low    'MainMol' Ueq as Compared to Neighbors of        C35 Check
PLAT300_ALERT_4_G Atom Site Occupancy of F2         Constrained at        0.8 Check
PLAT300_ALERT_4_G Atom Site Occupancy of F3         Constrained at        0.8 Check
PLAT300_ALERT_4_G Atom Site Occupancy of F2B        Constrained at        0.2 Check
PLAT300_ALERT_4_G Atom Site Occupancy of F3B        Constrained at        0.2 Check
PLAT301_ALERT_3_G Main Residue  Disorder ..............(Resd    1)         3% Note
PLAT434_ALERT_2_G Short Inter HL..HL Contact F5       ..F3B      .       2.66 Ang.
                                              -x,1-y,1-z  =     3_566 Check
PLAT434_ALERT_2_G Short Inter HL..HL Contact F6       ..F3B      .       2.82 Ang.
                                              -x,1-y,1-z  =     3_566 Check
PLAT434_ALERT_2_G Short Inter HL..HL Contact F7       ..F2B      .       2.77 Ang.
                                              -x,1-y,1-z  =     3_566 Check
PLAT794_ALERT_5_G Tentative Bond Valency for Co1       (II)      .       2.06 Info
PLAT860_ALERT_3_G Number of Least-Squares Restraints ............          10 Note
PLAT883_ALERT_1_G No Info/Value for _atom_sites_solution_primary .     Please Do !
PLAT910_ALERT_3_G Missing # of FCF Reflection(s) Below Theta(Min).          1 Note
                1  0  0,
PLAT967_ALERT_5_G Note: Two-Theta Cutoff Value in Embedded .res ..       53.0 Degree
PLAT969_ALERT_5_G The 'Henn et al.' R-Factor-gap value ...........      2.016 Note
              Predicted wR2: Based on SigI**2  5.06 or SHELX Weight  9.99
PLAT978_ALERT_2_G Number C-C Bonds with Positive Residual Density.          4 Info
```

0 **ALERT level A** = Most likely a serious problem - resolve or explain
0 **ALERT level B** = A potentially serious problem, consider carefully
3 **ALERT level C** = Check. Ensure it is not caused by an omission or oversight
26 **ALERT level G** = General information/check it is not something unexpected

1 ALERT type 1 CIF construction/syntax error, inconsistent or missing data
13 ALERT type 2 Indicator that the structure model may be wrong or deficient
5 ALERT type 3 Indicator that the structure quality may be low
7 ALERT type 4 Improvement, methodology, query or suggestion
3 ALERT type 5 Informative message, check

It is advisable to attempt to resolve as many as possible of the alerts in all categories. Often the minor alerts point to easily fixed oversights, errors and omissions in your CIF or refinement strategy, so attention to these fine details can be worthwhile. In order to resolve some of the more serious problems it may be necessary to carry out additional measurements or structure refinements. However, the purpose of your study may justify the reported deviations and the more serious of these should normally be commented upon in the discussion or experimental section of a paper or in the "special_details" fields of the CIF. checkCIF was carefully designed to identify outliers and unusual parameters, but every test has its limitations and alerts that are not important in a particular case may appear. Conversely, the absence of alerts does not guarantee there are no aspects of the results needing attention. It is up to the individual to critically assess their own results and, if necessary, seek expert advice.

Publication of your CIF in IUCr journals

A basic structural check has been run on your CIF. These basic checks will be run on all CIFs submitted for publication in IUCr journals (*Acta Crystallographica*, *Journal of Applied Crystallography*, *Journal of Synchrotron Radiation*); however, if you intend to submit to *Acta Crystallographica Section C* or *E* or *IUCrData*, you should make sure that full publication checks are run on the final version of your CIF prior to submission.

Publication of your CIF in other journals

Please refer to the *Notes for Authors* of the relevant journal for any special instructions relating to CIF submission.

PLATON version of 15/07/2024; check.def file version of 15/07/2024

Datablock mo_2024_ee97_1_0ma - ellipsoid plot

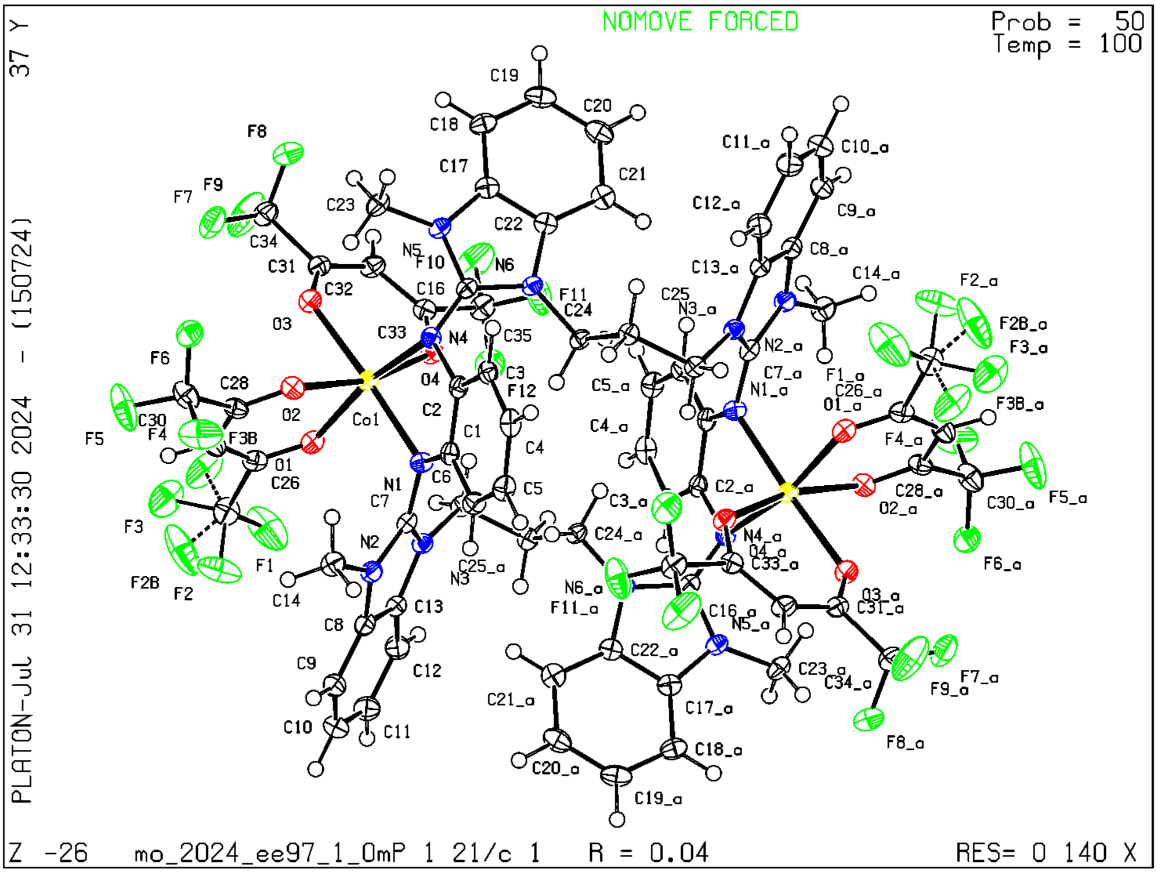

Supplement: Supplementary file 2 — Supporting Information [file CHEM-31-e02457-s002.zip › mo_2024_ee97_1_0ma_cifreport.pdf]
