# Supplementary material for: Steric Restraints in Redox‐Active Guanidine Ligands and Their Impact on Coordination Chemistry
Source: Chemistry. 2025 Oct 25;31(66):e02457. doi: 10.1002/chem.202502457 (PMC12648461; doi:10.1002/chem.202502457)

checkCIF/PLATON report

Structure factors have been supplied for datablock(s) mo_2023_ee100_0a

THIS REPORT IS FOR GUIDANCE ONLY. IF USED AS PART OF A REVIEW PROCEDURE FOR PUBLICATION, IT SHOULD NOT REPLACE THE EXPERTISE OF AN EXPERIENCED CRYSTALLOGRAPHIC REFEREE.

No syntax errors found. CIF dictionary Interpreting this report

Datablock: mo_2023_ee100_0a

```
Bond precision:   C-C = 0.0032 A          Wavelength=0.71073

Cell:              a=10.4925(6)      b=17.1109(9)      c=13.9826(8)
                   alpha=90          beta=90           gamma=90
Temperature:       120 K

                Calculated                      Reported
Volume          2510.4(2)                       2510.4(2)
Space group     P n a 21                        P n a 21
Hall group      P 2c -2n                        P 2c -2n
Moiety formula  C24 H23 N6, C F3 O3 S           C F3 O3 S, C24 H23 N6
Sum formula     C25 H23 F3 N6 O3 S              C25 H23 F3 N6 O3 S
Mr              544.55                          544.55
Dx,g cm-3       1.441                           1.441
Z               4                               4
Mu (mm-1)       0.192                           0.192
F000            1128.0                          1128.0
F000'           1129.08
h,k,lmax        13,22,18                        13,22,18
Nref            6091[ 3168]                     6056
Tmin,Tmax       0.955,0.966                     0.692,0.739
Tmin'           0.955

Correction method= # Reported T Limits: Tmin=0.692 Tmax=0.739
AbsCorr = MULTI-SCAN

Data completeness= 1.91/0.99        Theta(max)= 28.058

R(reflections)= 0.0319( 5806)                          wR2(reflections)=
                                                       0.0833( 6056)
S = 1.022                    Npar= 349
```

The following ALERTS were generated. Each ALERT has the format
test-name_ALERT_alert-type_alert-level.
Click on the hyperlinks for more details of the test.

Alert level C

```
PLAT042_ALERT_1_C Calc. and Reported MoietyFormula Strings  Differ     Please Check
PLAT244_ALERT_4_C Low    'Solvent' Ueq as Compared to Neighbors of         S1 Check
PLAT601_ALERT_2_C Unit Cell Contains Solvent Accessible VOIDS of .         36 Ang**3
```

Alert level G

```
PLAT244_ALERT_4_G Low    'Solvent' Ueq as Compared to Neighbors of        C25 Check
PLAT912_ALERT_4_G Missing # of FCF Reflections Above STh/L=  0.600         11 Note
PLAT978_ALERT_2_G Number C-C Bonds with Positive Residual Density.          8 Info
```

0 **ALERT level A** = Most likely a serious problem - resolve or explain
0 **ALERT level B** = A potentially serious problem, consider carefully
3 **ALERT level C** = Check. Ensure it is not caused by an omission or oversight
3 **ALERT level G** = General information/check it is not something unexpected

1 ALERT type 1 CIF construction/syntax error, inconsistent or missing data
2 ALERT type 2 Indicator that the structure model may be wrong or deficient
0 ALERT type 3 Indicator that the structure quality may be low
3 ALERT type 4 Improvement, methodology, query or suggestion
0 ALERT type 5 Informative message, check

It is advisable to attempt to resolve as many as possible of the alerts in all categories. Often the minor alerts point to easily fixed oversights, errors and omissions in your CIF or refinement strategy, so attention to these fine details can be worthwhile. In order to resolve some of the more serious problems it may be necessary to carry out additional measurements or structure refinements. However, the purpose of your study may justify the reported deviations and the more serious of these should normally be commented upon in the discussion or experimental section of a paper or in the "special_details" fields of the CIF. checkCIF was carefully designed to identify outliers and unusual parameters, but every test has its limitations and alerts that are not important in a particular case may appear. Conversely, the absence of alerts does not guarantee there are no aspects of the results needing attention. It is up to the individual to critically assess their own results and, if necessary, seek expert advice.

Publication of your CIF in IUCr journals

A basic structural check has been run on your CIF. These basic checks will be run on all CIFs submitted for publication in IUCr journals (*Acta Crystallographica*, *Journal of Applied Crystallography*, *Journal of Synchrotron Radiation*); however, if you intend to submit to *Acta Crystallographica Section C* or *E* or *IUCrData*, you should make sure that full publication checks are run on the final version of your CIF prior to submission.

Publication of your CIF in other journals

Please refer to the *Notes for Authors* of the relevant journal for any special instructions relating to CIF submission.

PLATON version of 06/07/2023; check.def file version of 30/06/2023

Datablock mo_2023_ee100_0a - ellipsoid plot

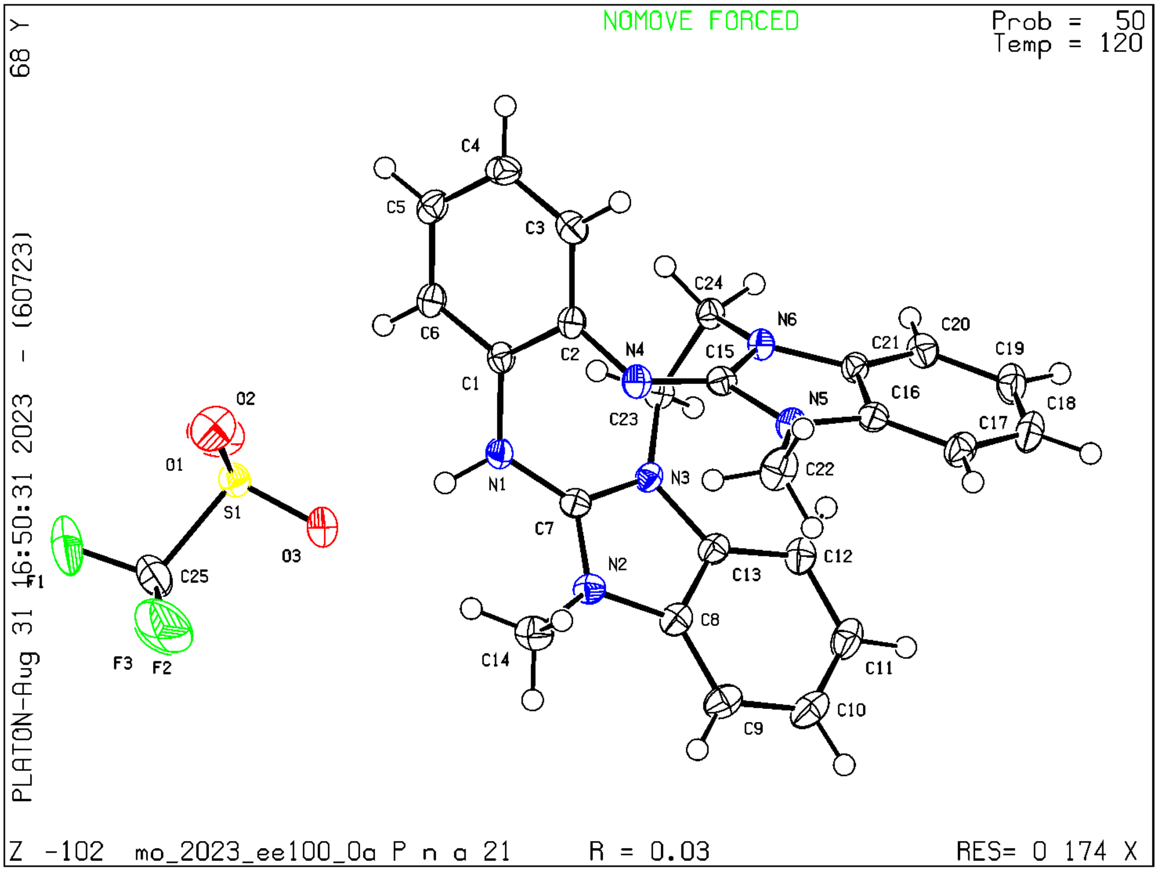

Supplement: Supplementary file 2 — Supporting Information [file CHEM-31-e02457-s002.zip › mo_2023_ee100_0a_cifreport.pdf]
