# Supplementary material for: Steric Restraints in Redox‐Active Guanidine Ligands and Their Impact on Coordination Chemistry
Source: Chemistry. 2025 Oct 25;31(66):e02457. doi: 10.1002/chem.202502457 (PMC12648461; doi:10.1002/chem.202502457)

checkCIF/PLATON report

Structure factors have been supplied for datablock(s) mo_2024_ee126_2_0ma

THIS REPORT IS FOR GUIDANCE ONLY. IF USED AS PART OF A REVIEW PROCEDURE FOR PUBLICATION, IT SHOULD NOT REPLACE THE EXPERTISE OF AN EXPERIENCED CRYSTALLOGRAPHIC REFEREE.

No syntax errors found. CIF dictionary Interpreting this report

Datablock: mo_2024_ee126_2_0ma

Bond precision:	C-C = 0.0040 A		Wavelength=0.71073
Cell:	a=14.9339(7)	b=11.5559(5)	c=17.8057(9)
	alpha=90	beta=93.936(2)	gamma=90
Temperature:	100 K		

	Calculated	Reported
Volume	3065.6(2)	3065.6(2)
Space group	P 21/n	P 1 21/n 1
Hall group	-P 2yn	-P 2yn
Moiety formula	C58 H52 Br4 Co2 N12, 2(C H2 Cl2)	C58 H52 Br4 Co2 N12, 2(C H2 Cl2)
Sum formula	C60 H56 Br4 Cl4 Co2 N12	C60 H56 Br4 Cl4 Co2 N12
Mr	1524.43	1524.46
Dx,g cm-3	1.651	1.652
Z	2	2
Mu (mm-1)	3.372	3.372
F000	1524.0	1524.0
F000'	1524.69	
h,k,lmax	19,14,23	19,14,23
Nref	6990	6981
Tmin,Tmax	0.734,0.825	0.679,0.746
Tmin'	0.658	

Correction method= # Reported T Limits: Tmin=0.679 Tmax=0.746
AbsCorr = MULTI-SCAN

Data completeness= 0.999 Theta(max)= 27.421

R(reflections)= 0.0351(5832) wR2(reflections)= 0.0911(6981)

S = 1.052 Npar= 391

The following ALERTS were generated. Each ALERT has the format
test-name_ALERT_alert-type_alert-level.
Click on the hyperlinks for more details of the test.

Alert level C

```
PLAT213_ALERT_2_C Atom Br3              has ADP max/min Ratio .....        3.2 prolat
PLAT220_ALERT_2_C NonSolvent   Resd 1 Br   Ueq(max)/Ueq(min) Range         4.0 Ratio
PLAT242_ALERT_2_C Low    'MainMol' Ueq as Compared to Neighbors of         Co1 Check
```

Alert level G

```
PLAT002_ALERT_2_G Number of Distance or Angle Restraints on AtSite           5 Note
PLAT176_ALERT_4_G The CIF-Embedded .res File Contains SADI Records           2 Report
PLAT178_ALERT_4_G The CIF-Embedded .res File Contains SIMU Records           1 Report
PLAT232_ALERT_2_G Hirshfeld Test Diff (M-X)  Br2      --Co1      .         7.8 s.u.
PLAT301_ALERT_3_G Main Residue  Disorder ..............(Resd    1)          5% Note
PLAT860_ALERT_3_G Number of Least-Squares Restraints .............           6 Note
PLAT910_ALERT_3_G Missing # of FCF Reflection(s) Below Theta(Min).           2 Note
               -1  0  1,   1  0  1,
PLAT912_ALERT_4_G Missing # of FCF Reflections Above STh/L=  0.600           6 Note
PLAT969_ALERT_5_G The 'Henn et al.' R-Factor-gap value ...........        2.97 Note
              Predicted wR2: Based on SigI**2  3.07 or SHELX Weight  8.92
PLAT978_ALERT_2_G Number C-C Bonds with Positive Residual Density.           4 Info
```

```
   0 ALERT level A = Most likely a serious problem - resolve or explain
   0 ALERT level B = A potentially serious problem, consider carefully
   3 ALERT level C = Check. Ensure it is not caused by an omission or oversight
  10 ALERT level G = General information/check it is not something unexpected

   0 ALERT type 1 CIF construction/syntax error, inconsistent or missing data
   6 ALERT type 2 Indicator that the structure model may be wrong or deficient
   3 ALERT type 3 Indicator that the structure quality may be low
   3 ALERT type 4 Improvement, methodology, query or suggestion
   1 ALERT type 5 Informative message, check
```

It is advisable to attempt to resolve as many as possible of the alerts in all categories. Often the minor alerts point to easily fixed oversights, errors and omissions in your CIF or refinement strategy, so attention to these fine details can be worthwhile. In order to resolve some of the more serious problems it may be necessary to carry out additional measurements or structure refinements. However, the purpose of your study may justify the reported deviations and the more serious of these should normally be commented upon in the discussion or experimental section of a paper or in the "special_details" fields of the CIF. checkCIF was carefully designed to identify outliers and unusual parameters, but every test has its limitations and alerts that are not important in a particular case may appear. Conversely, the absence of alerts does not guarantee there are no aspects of the results needing attention. It is up to the individual to critically assess their own results and, if necessary, seek expert advice.

Publication of your CIF in IUCr journals

A basic structural check has been run on your CIF. These basic checks will be run on all CIFs submitted for publication in IUCr journals (*Acta Crystallographica*, *Journal of Applied Crystallography*, *Journal of Synchrotron Radiation*); however, if you intend to submit to *Acta Crystallographica Section C* or *E* or *IUCrData*, you should make sure that full publication checks are run on the final version of your CIF prior to submission.

Publication of your CIF in other journals

Please refer to the *Notes for Authors* of the relevant journal for any special instructions relating to CIF submission.

PLATON version of 06/01/2024; check.def file version of 05/01/2024

Datablock mo_2024_ee126_2_0ma - ellipsoid plot

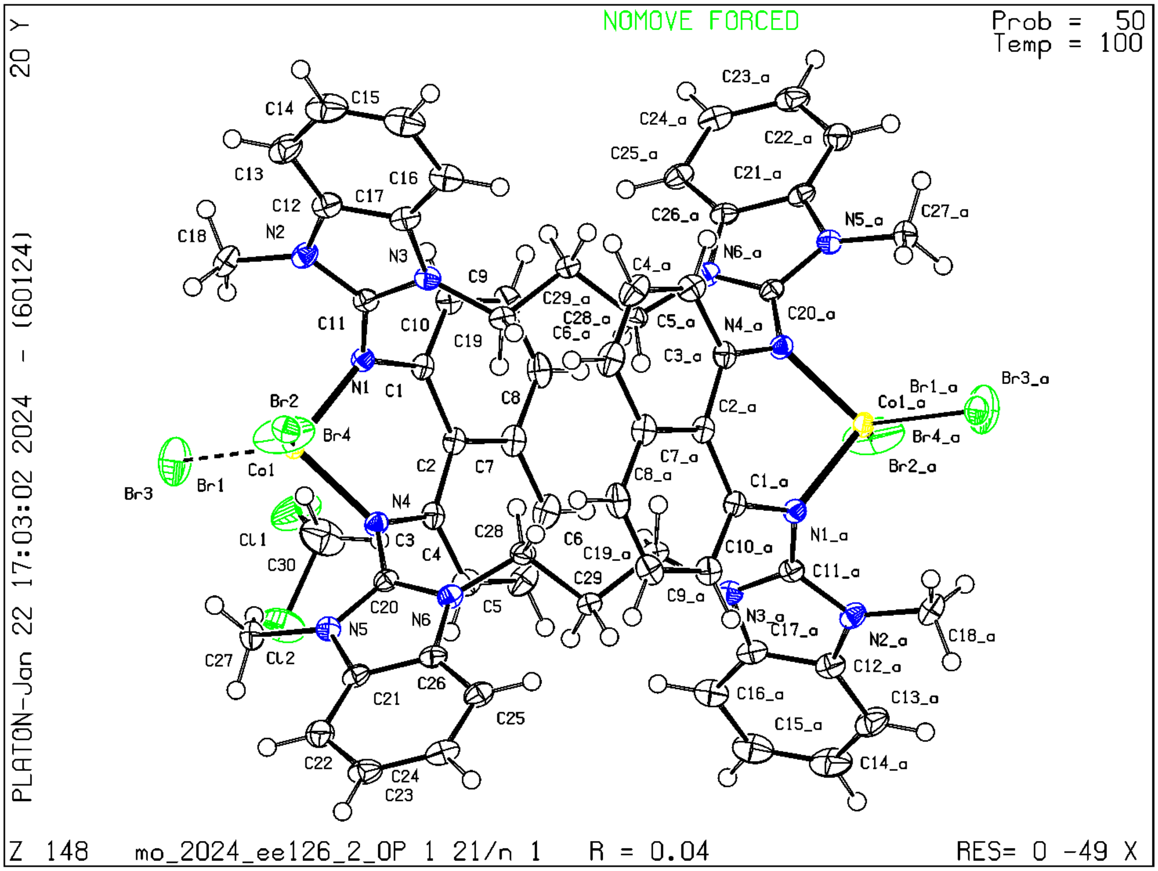

Supplement: Supplementary file 2 — Supporting Information [file CHEM-31-e02457-s002.zip › mo_2024_ee126_2_0ma_cifreport.pdf]
