# Supplementary material for: Steric Restraints in Redox‐Active Guanidine Ligands and Their Impact on Coordination Chemistry
Source: Chemistry. 2025 Oct 25;31(66):e02457. doi: 10.1002/chem.202502457 (PMC12648461; doi:10.1002/chem.202502457)

checkCIF/PLATON report

Structure factors have been supplied for datablock(s) mo_2024_ee132_0m

THIS REPORT IS FOR GUIDANCE ONLY. IF USED AS PART OF A REVIEW PROCEDURE FOR PUBLICATION, IT SHOULD NOT REPLACE THE EXPERTISE OF AN EXPERIENCED CRYSTALLOGRAPHIC REFEREE.

No syntax errors found. CIF dictionary Interpreting this report

Datablock: mo_2024_ee132_0m

Bond precision:	C-C = 0.0038 A		Wavelength=0.71073
Cell:	a=9.9722(7)	b=12.7531(8)	c=23.6314(12)
	alpha=90	beta=100.359(2)	gamma=90
Temperature:	100 K		

	Calculated	Reported
Volume	2956.4(3)	2956.4(3)
Space group	P 21/n	P 1 21/n 1
Hall group	-P 2yn	-P 2yn
Moiety formula	C26 H28 Br2 Co N6 [+ solvent]	C26 H28 Br2 Co N6, 1[CH2CL2]
Sum formula	C26 H28 Br2 Co N6 [+ solvent]	C27 H30 Br2 Cl2 Co N6
Mr	643.27	728.22
Dx,g cm-3	1.445	1.636
Z	4	4
Mu (mm-1)	3.307	3.493
F000	1292.0	1460.0
F000'	1291.47	
h,k,lmax	12,16,30	12,16,30
Nref	6462	6458
Tmin,Tmax	0.279,0.389	0.526,0.746
Tmin'	0.235	

Correction method= # Reported T Limits: Tmin=0.526 Tmax=0.746
AbsCorr = MULTI-SCAN

Data completeness= 0.999 Theta(max)= 26.996

R(reflections)= 0.0344(6094)

wR2(reflections)= 0.0849(6458)

S = 1.046

Npar= 350

The following ALERTS were generated. Each ALERT has the format
test-name_ALERT_alert-type_alert-level.
Click on the hyperlinks for more details of the test.

Alert level C

CRYSC01_ALERT_1_C The word below has not been recognised as a standard identifier.
bluish
PLAT213_ALERT_2_C Atom C15 has ADP max/min Ratio ..... 3.8 prolat
PLAT213_ALERT_2_C Atom C16 has ADP max/min Ratio ..... 3.7 prolat
PLAT220_ALERT_2_C NonSolvent Resd 1 C Ueq(max)/Ueq(min) Range 5.4 Ratio
PLAT222_ALERT_3_C NonSolvent Resd 1 H Uiso(max)/Uiso(min) Range 5.8 Ratio
PLAT911_ALERT_3_C Missing FCF Refl Between Thmin & STh/L= 0.600 2 Report
6 0 8, -7 0 11,

Alert level G

FORMU01_ALERT_2_G There is a discrepancy between the atom counts in the _chemical_formula_sum and the formula from the _atom_site* data.
Atom count from _chemical_formula_sum:C27 H30 Br2 Cl2 Co1 N6
Atom count from the _atom_site data: C26 H28 Br2 Co1 N6
CELLZ01_ALERT_1_G Difference between formula and atom_site contents detected.
CELLZ01_ALERT_1_G ALERT: Large difference may be due to a symmetry error - see SYMMG tests
From the CIF: _cell_formula_units_Z 4
From the CIF: _chemical_formula_sum C27 H30 Br2 Cl2 Co N6
TEST: Compare cell contents of formula and atom_site data

atom	Z*formula	cif sites	diff
C	108.00	104.00	4.00
H	120.00	112.00	8.00
Br	8.00	8.00	0.00
Cl	8.00	0.00	8.00
Co	4.00	4.00	0.00
N	24.00	24.00	0.00

PLAT002_ALERT_2_G Number of Distance or Angle Restraints on AtSite 4 Note
PLAT003_ALERT_2_G Number of Uiso or Uij Restrained non-H Atoms ... 2 Report
PLAT041_ALERT_1_G Calc. and Reported SumFormula Strings Differ Please Check
Calc: C26 H28 Br2 Co N6
Rep.: C27 H30 Br2 Cl2 Co N6
PLAT042_ALERT_1_G Calc. and Reported MoietyFormula Strings Differ Please Check
Calc: C26 H28 Br2 Co N6
Rep.: C26 H28 Br2 Co N6, 1[CH2CL2]
PLAT051_ALERT_1_G Mu(calc) and Mu(CIF) Ratio Differs from 1.0 by . 5.32 %
PLAT083_ALERT_2_G SHELXL Second Parameter in WGHT Unusually Large 6.73 Why ?
PLAT168_ALERT_4_G The CIF-Embedded .res File Contains EXYZ Records 2 Report
PLAT171_ALERT_4_G The CIF-Embedded .res File Contains EADP Records 2 Report
PLAT176_ALERT_4_G The CIF-Embedded .res File Contains SADI Records 1 Report
PLAT178_ALERT_4_G The CIF-Embedded .res File Contains SIMU Records 2 Report
PLAT300_ALERT_4_G Atom Site Occupancy of C15 Constrained at 0.75 Check

```
PLAT300_ALERT_4_G Atom Site Occupancy of C16        Constrained at       0.75 Check
PLAT300_ALERT_4_G Atom Site Occupancy of C24        Constrained at        0.5 Check
PLAT300_ALERT_4_G Atom Site Occupancy of C24B       Constrained at        0.5 Check
PLAT300_ALERT_4_G Atom Site Occupancy of C25        Constrained at        0.5 Check
PLAT300_ALERT_4_G Atom Site Occupancy of C25B       Constrained at        0.5 Check
PLAT300_ALERT_4_G Atom Site Occupancy of C26        Constrained at        0.5 Check
PLAT300_ALERT_4_G Atom Site Occupancy of C26B       Constrained at        0.5 Check
PLAT300_ALERT_4_G Atom Site Occupancy of C15B       Constrained at       0.25 Check
PLAT300_ALERT_4_G Atom Site Occupancy of C16B       Constrained at       0.25 Check
PLAT300_ALERT_4_G Atom Site Occupancy of H15A       Constrained at       0.75 Check
PLAT300_ALERT_4_G Atom Site Occupancy of H15B       Constrained at       0.75 Check
PLAT300_ALERT_4_G Atom Site Occupancy of H16A       Constrained at       0.75 Check
PLAT300_ALERT_4_G Atom Site Occupancy of H16B       Constrained at       0.75 Check
PLAT300_ALERT_4_G Atom Site Occupancy of H16C       Constrained at       0.75 Check
PLAT300_ALERT_4_G Atom Site Occupancy of H24A       Constrained at        0.5 Check
PLAT300_ALERT_4_G Atom Site Occupancy of H24B       Constrained at        0.5 Check
PLAT300_ALERT_4_G Atom Site Occupancy of H24C       Constrained at        0.5 Check
PLAT300_ALERT_4_G Atom Site Occupancy of H24D       Constrained at        0.5 Check
PLAT300_ALERT_4_G Atom Site Occupancy of H24E       Constrained at        0.5 Check
PLAT300_ALERT_4_G Atom Site Occupancy of H25A       Constrained at        0.5 Check
PLAT300_ALERT_4_G Atom Site Occupancy of H25B       Constrained at        0.5 Check
PLAT300_ALERT_4_G Atom Site Occupancy of H25C       Constrained at        0.5 Check
PLAT300_ALERT_4_G Atom Site Occupancy of H25D       Constrained at        0.5 Check
PLAT300_ALERT_4_G Atom Site Occupancy of H25E       Constrained at        0.5 Check
PLAT300_ALERT_4_G Atom Site Occupancy of H26A       Constrained at        0.5 Check
PLAT300_ALERT_4_G Atom Site Occupancy of H26B       Constrained at        0.5 Check
PLAT300_ALERT_4_G Atom Site Occupancy of H26C       Constrained at        0.5 Check
PLAT300_ALERT_4_G Atom Site Occupancy of H26D       Constrained at        0.5 Check
PLAT300_ALERT_4_G Atom Site Occupancy of H26E       Constrained at        0.5 Check
PLAT300_ALERT_4_G Atom Site Occupancy of H26F       Constrained at        0.5 Check
PLAT300_ALERT_4_G Atom Site Occupancy of H15C       Constrained at       0.25 Check
PLAT300_ALERT_4_G Atom Site Occupancy of H15D       Constrained at       0.25 Check
PLAT300_ALERT_4_G Atom Site Occupancy of H16D       Constrained at       0.25 Check
PLAT300_ALERT_4_G Atom Site Occupancy of H16E       Constrained at       0.25 Check
PLAT300_ALERT_4_G Atom Site Occupancy of H16F       Constrained at       0.25 Check
PLAT301_ALERT_3_G Main Residue  Disorder .............(Resd    1)        14% Note
PLAT432_ALERT_2_G Short Inter X...Y Contact  C10      ..C16B     .       3.00 Ang.
                                5/2-x,1/2+y,1/2-z  =      2_755 Check
PLAT605_ALERT_4_G Largest Solvent Accessible VOID in the Structure        218 A**3
PLAT860_ALERT_3_G Number of Least-Squares Restraints ............           7 Note
PLAT868_ALERT_4_G ALERTS Due to the Use of _smtbx_masks Suppressed          ! Info
PLAT910_ALERT_3_G Missing # of FCF Reflection(s) Below Theta(Min).          2 Note
                0  1  1,   0  0  2,
PLAT967_ALERT_5_G Note: Two-Theta Cutoff Value in Embedded .res ..       54.0 Degree
PLAT969_ALERT_5_G The 'Henn et al.' R-Factor-gap value ..........       2.03 Note
              Predicted wR2: Based on SigI**2  4.18 or SHELX Weight  8.34
PLAT978_ALERT_2_G Number C-C Bonds with Positive Residual Density.          6 Info
```

---

0 **ALERT level A** = Most likely a serious problem - resolve or explain

0 **ALERT level B** = A potentially serious problem, consider carefully

6 **ALERT level C** = Check. Ensure it is not caused by an omission or oversight

58 **ALERT level G** = General information/check it is not something unexpected

6 ALERT type 1 CIF construction/syntax error, inconsistent or missing data

```
 9 ALERT type 2 Indicator that the structure model may be wrong or deficient
 5 ALERT type 3 Indicator that the structure quality may be low
42 ALERT type 4 Improvement, methodology, query or suggestion
 2 ALERT type 5 Informative message, check
```

It is advisable to attempt to resolve as many as possible of the alerts in all categories. Often the minor alerts point to easily fixed oversights, errors and omissions in your CIF or refinement strategy, so attention to these fine details can be worthwhile. In order to resolve some of the more serious problems it may be necessary to carry out additional measurements or structure refinements. However, the purpose of your study may justify the reported deviations and the more serious of these should normally be commented upon in the discussion or experimental section of a paper or in the "special_details" fields of the CIF. checkCIF was carefully designed to identify outliers and unusual parameters, but every test has its limitations and alerts that are not important in a particular case may appear. Conversely, the absence of alerts does not guarantee there are no aspects of the results needing attention. It is up to the individual to critically assess their own results and, if necessary, seek expert advice.

Publication of your CIF in IUCr journals

A basic structural check has been run on your CIF. These basic checks will be run on all CIFs submitted for publication in IUCr journals (*Acta Crystallographica*, *Journal of Applied Crystallography*, *Journal of Synchrotron Radiation*); however, if you intend to submit to *Acta Crystallographica Section C* or *E* or *IUCrData*, you should make sure that full publication checks are run on the final version of your CIF prior to submission.

Publication of your CIF in other journals

Please refer to the *Notes for Authors* of the relevant journal for any special instructions relating to CIF submission.

PLATON version of 06/01/2024; check.def file version of 05/01/2024

Datablock mo_2024_ee132_0m - ellipsoid plot

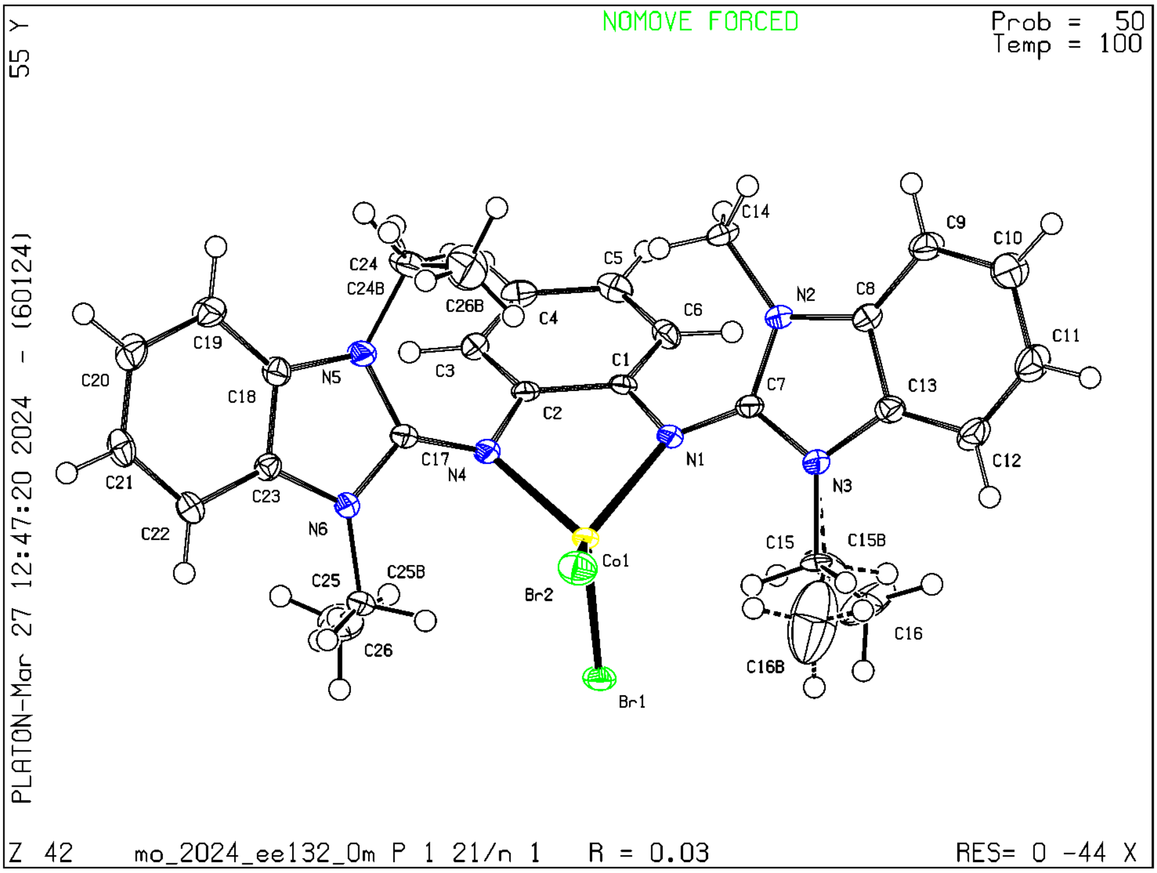

Supplement: Supplementary file 2 — Supporting Information [file CHEM-31-e02457-s002.zip › mo_2024_ee132_0m_cifreport.pdf]
