# Supplementary material for: Steric Restraints in Redox‐Active Guanidine Ligands and Their Impact on Coordination Chemistry
Source: Chemistry. 2025 Oct 25;31(66):e02457. doi: 10.1002/chem.202502457 (PMC12648461; doi:10.1002/chem.202502457)

checkCIF/PLATON report

Structure factors have been supplied for datablock(s) mo_2024_ee183_0m

THIS REPORT IS FOR GUIDANCE ONLY. IF USED AS PART OF A REVIEW PROCEDURE FOR PUBLICATION, IT SHOULD NOT REPLACE THE EXPERTISE OF AN EXPERIENCED CRYSTALLOGRAPHIC REFEREE.

No syntax errors found. CIF dictionary Interpreting this report

Datablock: mo_2024_ee183_0m

```
Bond precision:   C-C = 0.0031 A          Wavelength=0.71073

Cell:             a=11.7598(11)    b=13.0255(14)     c=16.7382(18)
                  alpha=90         beta=93.536(4)    gamma=90
Temperature:      100 K

                 Calculated                  Reported
Volume           2559.0(5)                   2559.0(5)
Space group      P 21/n                      P 1 21/n 1
Hall group       -P 2yn                      -P 2yn
Moiety formula   C30 H26 N8, H2 O            H2 O, C30 H26 N8
Sum formula      C30 H28 N8 O                C30 H28 N8 O
Mr               516.60                      516.60
Dx,g cm-3        1.341                       1.341
Z                4                           4
Mu (mm-1)        0.086                       0.086
F000             1088.0                      1088.0
F000'            1088.36
h,k,lmax         14,16,20                    14,16,20
Nref             5037                        5036
Tmin,Tmax        0.987,0.995                 0.589,0.745
Tmin'            0.985

Correction method= # Reported T Limits: Tmin=0.589 Tmax=0.745
AbsCorr = MULTI-SCAN

Data completeness= 1.000           Theta(max)= 25.998

R(reflections)= 0.0478( 3176)                        wR2(reflections)=
                                                     0.1379( 5036)
S = 1.020                    Npar= 359
```

The following ALERTS were generated. Each ALERT has the format
test-name_ALERT_alert-type_alert-level.
Click on the hyperlinks for more details of the test.

Alert level C

```
RINTA01_ALERT_3_C  The value of Rint is greater than 0.12
            Rint given   0.124
PLAT042_ALERT_1_C Calc. and Reported MoietyFormula Strings  Differ     Please Check
              Calc: C30 H26 N8, H2 O
              Rep.: H2 O, C30 H26 N8
PLAT905_ALERT_3_C Negative K value in the Analysis of Variance ...     -4.470 Report
```

Alert level G

```
PLAT007_ALERT_5_G Number of Unrefined Donor-H Atoms .............          2 Report
              H1A   H1B
PLAT020_ALERT_3_G The Value of Rint is Greater Than 0.12 .........      0.124 Report
PLAT913_ALERT_3_G Missing # of Very Strong Reflections in FCF ....          1 Note
                0  4  0,
PLAT967_ALERT_5_G Note: Two-Theta Cutoff Value in Embedded .res ..       52.0 Degree
PLAT969_ALERT_5_G The 'Henn et al.' R-Factor-gap value ..........      1.957 Note
              Predicted wR2: Based on SigI**2  7.05 or SHELX Weight 13.53
PLAT978_ALERT_2_G Number C-C Bonds with Positive Residual Density.          1 Info
```

0 **ALERT level A** = Most likely a serious problem - resolve or explain
0 **ALERT level B** = A potentially serious problem, consider carefully
3 **ALERT level C** = Check. Ensure it is not caused by an omission or oversight
6 **ALERT level G** = General information/check it is not something unexpected

1 ALERT type 1 CIF construction/syntax error, inconsistent or missing data
1 ALERT type 2 Indicator that the structure model may be wrong or deficient
4 ALERT type 3 Indicator that the structure quality may be low
0 ALERT type 4 Improvement, methodology, query or suggestion
3 ALERT type 5 Informative message, check

It is advisable to attempt to resolve as many as possible of the alerts in all categories. Often the minor alerts point to easily fixed oversights, errors and omissions in your CIF or refinement strategy, so attention to these fine details can be worthwhile. In order to resolve some of the more serious problems it may be necessary to carry out additional measurements or structure refinements. However, the purpose of your study may justify the reported deviations and the more serious of these should normally be commented upon in the discussion or experimental section of a paper or in the "special_details" fields of the CIF. checkCIF was carefully designed to identify outliers and unusual parameters, but every test has its limitations and alerts that are not important in a particular case may appear. Conversely, the absence of alerts does not guarantee there are no aspects of the results needing attention. It is up to the individual to critically assess their own results and, if necessary, seek expert advice.

Publication of your CIF in IUCr journals

A basic structural check has been run on your CIF. These basic checks will be run on all CIFs submitted for publication in IUCr journals (*Acta Crystallographica*, *Journal of Applied Crystallography*, *Journal of Synchrotron Radiation*); however, if you intend to submit to *Acta Crystallographica Section C* or *E* or *IUCrData*, you should make sure that full publication checks are run on the final version of your CIF prior to submission.

Publication of your CIF in other journals

Please refer to the *Notes for Authors* of the relevant journal for any special instructions relating to CIF submission.

PLATON version of 22/08/2024; check.def file version of 21/08/2024

Datablock mo_2024_ee183_0m - ellipsoid plot

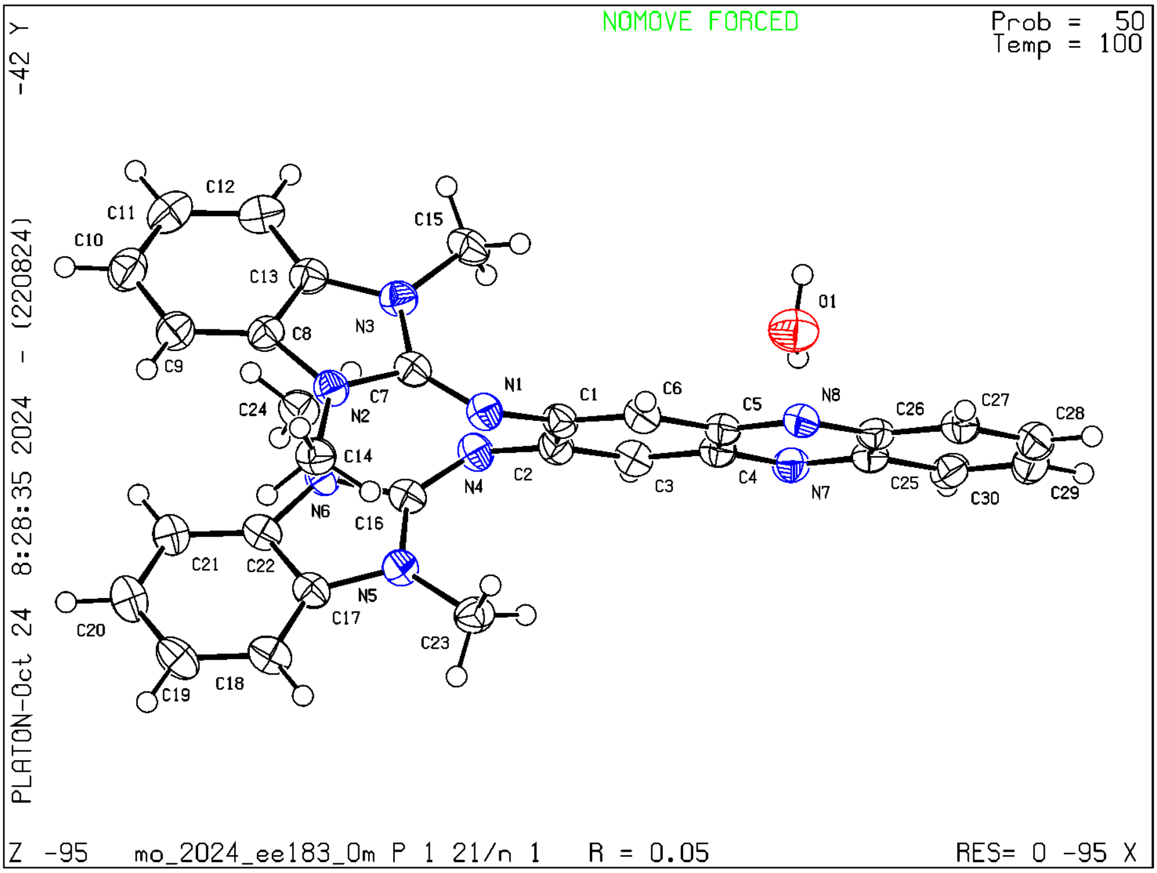

Supplement: Supplementary file 2 — Supporting Information [file CHEM-31-e02457-s002.zip › mo_2024_ee183_0m_cifreport.pdf]
