# Supplementary material for: Steric Restraints in Redox‐Active Guanidine Ligands and Their Impact on Coordination Chemistry
Source: Chemistry. 2025 Oct 25;31(66):e02457. doi: 10.1002/chem.202502457 (PMC12648461; doi:10.1002/chem.202502457)

checkCIF/PLATON report

Structure factors have been supplied for datablock(s) mo_2025_ee202_0m

THIS REPORT IS FOR GUIDANCE ONLY. IF USED AS PART OF A REVIEW PROCEDURE FOR PUBLICATION, IT SHOULD NOT REPLACE THE EXPERTISE OF AN EXPERIENCED CRYSTALLOGRAPHIC REFEREE.

No syntax errors found. CIF dictionary Interpreting this report

Datablock: mo_2025_ee202_0m

Bond precision: C-C = 0.0099 A Wavelength=0.71073

Cell:	a=10.3851(8)	b=20.8242(17)	c=26.228(2)
	alpha=90	beta=96.594(3)	gamma=90
Temperature:	100 K		

	Calculated	Reported
Volume	5634.6(8)	5634.5(8)
Space group	P 21/n	P 1 21/n 1
Hall group	-P 2yn	-P 2yn
Moiety formula	2(C25 H24 Cl2 N6 Ni), C H2 Cl2 [+ solvent]	C25 H24 Cl2 N6 Ni, 0.5(C H2 Cl2), 0.45[C4H10O], 0.25[CH2CL2]
Sum formula	C51 H50 Cl6 N12 Ni2 [+ solvent]	C27.55 H30 Cl3.50 N6 Ni O0.45
Mr	1161.11	635.16
Dx,g cm-3	1.369	1.497
Z	4	8
Mu (mm-1)	0.998	1.052
F000	2392.0	2627.0
F000'	2398.76	
h,k,lmax	12,25,32	12,25,32
Nref	11079	11040
Tmin,Tmax	0.838,0.919	0.565,0.745
Tmin'	0.827	

Correction method= # Reported T Limits: Tmin=0.565 Tmax=0.745
AbsCorr = MULTI-SCAN

Data completeness= 0.996 Theta(max)= 26.000

R(reflections)= 0.0759(6985) wR2(reflections)= 0.2217(11040)

S = 1.027 Npar= 644

The following ALERTS were generated. Each ALERT has the format
test-name_ALERT_alert-type_alert-level.
Click on the hyperlinks for more details of the test.

Alert level B

```
RINTA01_ALERT_3_B  The value of Rint is greater than 0.18
            Rint given   0.232
```

Author Response: The crystal was very small and weakly diffracting even after prolonged exposure time.

```
PLAT020_ALERT_3_B The Value of Rint is Greater Than 0.12 .........      0.232 Report
```

Author Response: The crystal was very small and weakly diffracting even after prolonged exposure time.

Alert level C

```
PLAT094_ALERT_2_C Ratio of Maximum / Minimum Residual Density ....       2.15 Report
PLAT341_ALERT_3_C Low Bond Precision on  C-C Bonds ..............    0.00995 Ang.
PLAT905_ALERT_3_C Negative K value in the Analysis of Variance ...     -3.575 Report
PLAT911_ALERT_3_C Missing FCF Refl Between Thmin & STh/L=    0.600         36 Report
                6  0  8,   6  1  8,   7  0  9,   6  1  9,   7  1  9,   6  2  9,
                6  0 10,   6  1 10,   7  1 10,   6  2 10,   7  2 10,   7  0 11,
                6  1 11,   7  1 11,   7  2 11,   7  3 11,   8  0 12,   7  1 12,
                7  2 12,   7  3 12,   7  0 13,   7  1 13,   8  1 13,   8  2 13,
                8  0 14,   7  1 14,   8  1 14,   8  2 14,   7  0 15,   9  0 15,
                   (    6 More Missing: see the .ckf listing file)
PLAT971_ALERT_2_C Check Calcd Resid. Dens.  0.91Ang From Ni2           1.65 eA-3
PLAT971_ALERT_2_C Check Calcd Resid. Dens.  0.86Ang From Ni1           1.52 eA-3
```

Alert level G

```
FORMU01_ALERT_1_G  There is a discrepancy between the atom counts in the
            _chemical_formula_sum and _chemical_formula_moiety. This is
            usually due to the moiety formula being in the wrong format.
            Atom count from _chemical_formula_sum:   C27.55 H30 Cl3.5 N6 Ni1 O0.45
            Atom count from _chemical_formula_moiety:C27.3 H29.5 Cl3 N6 Ni1 O0.45
FORMU01_ALERT_2_G  There is a discrepancy between the atom counts in the
            _chemical_formula_sum and the formula from the _atom_site* data.
            Atom count from _chemical_formula_sum:C27.55 H30 Cl3.5 N6 Ni1 O0.45
            Atom count from the _atom_site data:  C25.5 H25 Cl3 N6 Ni1
CELLZ01_ALERT_1_G Difference between formula and atom_site contents detected.
CELLZ01_ALERT_1_G ALERT: Large difference may be due to a
            symmetry error - see SYMMG tests
```

```
          From the CIF: _cell_formula_units_Z    8
          From the CIF: _chemical_formula_sum  C27.55 H30 Cl3.50 N6 Ni O0.45
          TEST: Compare cell contents of formula and atom_site data

          atom    Z*formula  cif sites diff
          C        220.40    204.00   16.40
          H        240.00    200.00   40.00
          Cl        28.00     24.00    4.00
          N         48.00     48.00    0.00
          Ni         8.00      8.00    0.00
          O          3.60      0.00    3.60
PLAT041_ALERT_1_G Calc. and Reported SumFormula    Strings  Differ     Please Check
             Calc: C51 H50 Cl6 N12 Ni2
             Rep.: C27.55 H30 Cl3.50 N6 Ni O0.45
PLAT042_ALERT_1_G Calc. and Reported MoietyFormula Strings  Differ     Please Check
             Calc: 2(C25 H24 Cl2 N6 Ni), C H2 Cl2
             Rep.: C25 H24 Cl2 N6 Ni, 0.5(C H2 Cl2), 0.45[C4H10O]
                   , 0.25[CH2CL2]
PLAT045_ALERT_1_G Calculated and Reported Z Differ by a Factor ...      0.500 Check
PLAT051_ALERT_1_G Mu(calc) and Mu(cif) Ratio Differs from 1.0 by .       5.12 %
PLAT083_ALERT_2_G SHELXL Second Parameter in WGHT  Unusually Large      28.38 Why ?
PLAT605_ALERT_4_G Largest Solvent Accessible VOID in the Structure        275 A**3
PLAT794_ALERT_5_G Tentative Bond Valency for Ni1       (II)      .       2.01 Info
PLAT794_ALERT_5_G Tentative Bond Valency for Ni2       (II)      .       1.99 Info
PLAT868_ALERT_4_G ALERTS Due to the Use of _smtbx_masks Suppressed          ! Info
PLAT910_ALERT_3_G Missing FCF Reflection(s) Below Theta(Min)[Deg]=       1.96 Note
               0  1  1,   0  0  2,   0  1  2,
PLAT967_ALERT_5_G Note: Two-Theta Cutoff Value in Embedded .res ..       52.0 Degree
PLAT969_ALERT_5_G The 'Henn et al.' R-Factor-gap value ..........      2.248 Note
             Predicted wR2: Based on SigI**2  9.87 or SHELX Weight 21.60
PLAT978_ALERT_2_G Number C-C Bonds with Positive Residual Density.          0 Info
```

---

   0 **ALERT level A** = Most likely a serious problem - resolve or explain
   2 **ALERT level B** = A potentially serious problem, consider carefully
   6 **ALERT level C** = Check. Ensure it is not caused by an omission or oversight
  17 **ALERT level G** = General information/check it is not something unexpected

```
   7 ALERT type 1 CIF construction/syntax error, inconsistent or missing data
   6 ALERT type 2 Indicator that the structure model may be wrong or deficient
   6 ALERT type 3 Indicator that the structure quality may be low
   2 ALERT type 4 Improvement, methodology, query or suggestion
   4 ALERT type 5 Informative message, check
```

It is advisable to attempt to resolve as many as possible of the alerts in all categories. Often the minor alerts point to easily fixed oversights, errors and omissions in your CIF or refinement strategy, so attention to these fine details can be worthwhile. In order to resolve some of the more serious problems it may be necessary to carry out additional measurements or structure refinements. However, the purpose of your study may justify the reported deviations and the more serious of these should normally be commented upon in the discussion or experimental section of a paper or in the "special_details" fields of the CIF. checkCIF was carefully designed to identify outliers and unusual parameters, but every test has its limitations and alerts that are not important in a particular case may appear. Conversely, the absence of alerts does not guarantee there are no aspects of the results needing attention. It is up to the individual to critically assess their own results and, if necessary, seek expert advice.

Publication of your CIF in IUCr journals

A basic structural check has been run on your CIF. These basic checks will be run on all CIFs submitted for publication in IUCr journals (*Acta Crystallographica*, *Journal of Applied Crystallography*, *Journal of Synchrotron Radiation*); however, if you intend to submit to *Acta Crystallographica Section C* or *E* or *IUCrData*, you should make sure that full publication checks are run on the final version of your CIF prior to submission.

Publication of your CIF in other journals

Please refer to the *Notes for Authors* of the relevant journal for any special instructions relating to CIF submission.

PLATON version of 04/06/2025; check.def file version of 30/05/2025

Datablock mo_2025_ee202_0m - ellipsoid plot

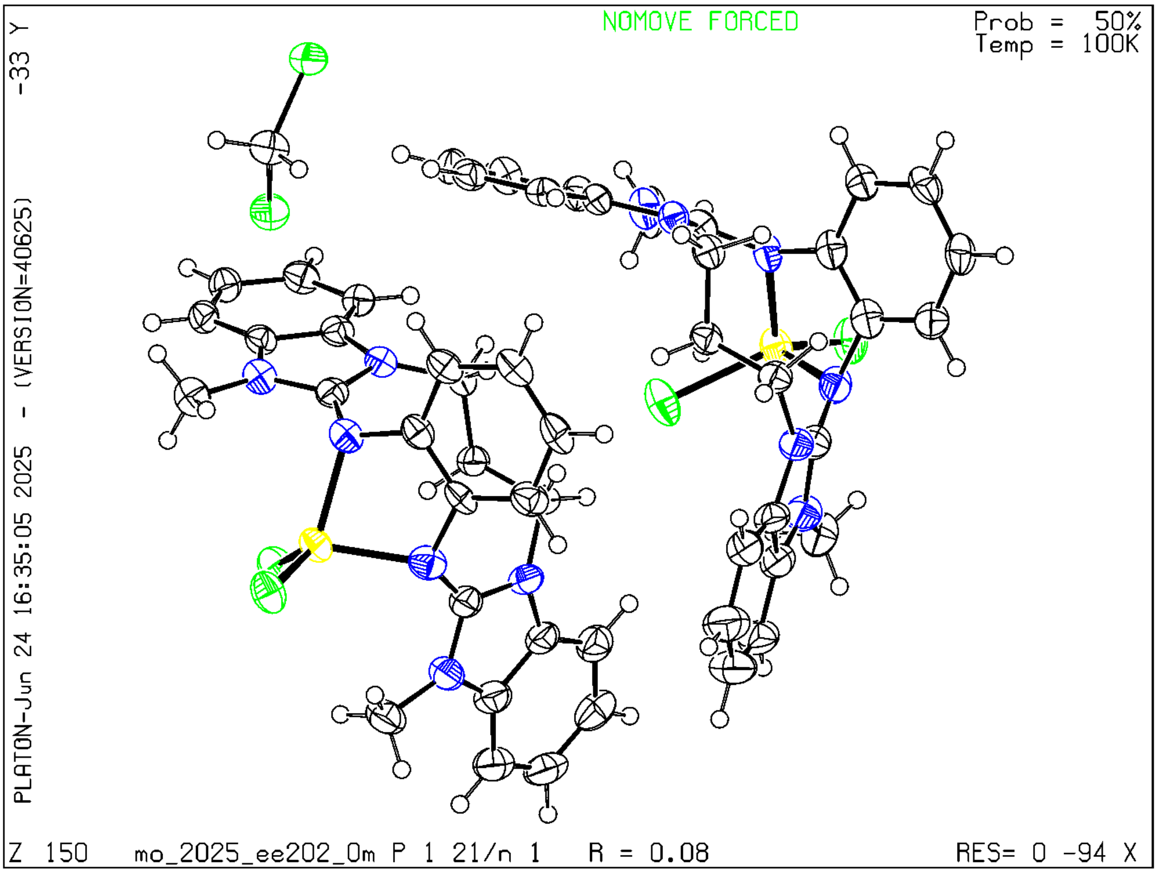

Supplement: Supplementary file 2 — Supporting Information [file CHEM-31-e02457-s002.zip › mo_2025_ee202_0m_cifreport.pdf]
