# Supplementary material for: Steric Restraints in Redox‐Active Guanidine Ligands and Their Impact on Coordination Chemistry
Source: Chemistry. 2025 Oct 25;31(66):e02457. doi: 10.1002/chem.202502457 (PMC12648461; doi:10.1002/chem.202502457)

# checkCIF/PLATON report

Structure factors have been supplied for datablock(s) mo_2025_ee203_0m

THIS REPORT IS FOR GUIDANCE ONLY. IF USED AS PART OF A REVIEW PROCEDURE FOR PUBLICATION, IT SHOULD NOT REPLACE THE EXPERTISE OF AN EXPERIENCED CRYSTALLOGRAPHIC REFEREE.

No syntax errors found. CIF dictionary Interpreting this report

## Datablock: mo_2025_ee203_0m

Bond precision: C-C = 0.0042 A Wavelength=0.71073

| | | | |
|---|---|---|---|
| Cell: | a=12.3922(13) | b=16.5375(16) | c=16.8789(16) |
| | alpha=73.022(4) | beta=70.960(4) | gamma=69.198(4) |
| Temperature: | 100 K | | |

| | Calculated | Reported |
|---|---|---|
| Volume | 2995.4(5) | 2995.4(5) |
| Space group | P -1 | P -1 |
| Hall group | -P 1 | -P 1 |
| Moiety formula | 2(C26 H26 Cl2 N6 Ni), C H2 Cl2 [+ solvent] | C26 H26 Cl2 N6 Ni, 0.5(C H2 Cl2), 1[CH2CL2] |
| Sum formula | C53 H54 Cl6 N12 Ni2 [+ solvent] | C27.50 H29 Cl5 N6 Ni |
| Mr | 1189.16 | 679.53 |
| Dx,g cm-3 | 1.319 | 1.507 |
| Z | 2 | 4 |
| Mu (mm-1) | 0.941 | 1.123 |
| F000 | 1228.0 | 1396.0 |
| F000' | 1231.39 | |
| h,k,lmax | 15,20,21 | 15,20,21 |
| Nref | 12503 | 12395 |
| Tmin,Tmax | 0.886,0.914 | 0.670,0.745 |
| Tmin' | 0.799 | |

Correction method= # Reported T Limits: Tmin=0.670 Tmax=0.745
AbsCorr = MULTI-SCAN

Data completeness= 0.991 Theta(max)= 26.565

R(reflections)= 0.0412( 10248) wR2(reflections)= 0.1217( 12395)

S = 1.079 Npar= 664

---

The following ALERTS were generated. Each ALERT has the format
**test-name_ALERT_alert-type_alert-level.**
Click on the hyperlinks for more details of the test.

---

### Alert level C

```
CRYSC01_ALERT_1_C The word below has not been recognised as a standard
            identifier.
            yellowish
PLAT910_ALERT_3_C Missing # of FCF Reflection(s) Below Theta(Min).          5 Note
                1  0  0,   0  1  0,   0  0  1,   0  1  1,   1  1  1,
PLAT911_ALERT_3_C Missing FCF Refl Between Thmin & STh/L=    0.600          2 Report
                1  0  1,   3  0  3,
```

---

### Alert level G

```
FORMU01_ALERT_2_G  There is a discrepancy between the atom counts in the
            _chemical_formula_sum and the formula from the _atom_site* data.
            Atom count from _chemical_formula_sum:C27.5 H29 Cl5 N6 Ni1
            Atom count from the _atom_site data:  C26.5 H27 Cl3 N6 Ni1
CELLZ01_ALERT_1_G Difference between formula and atom_site contents detected.
CELLZ01_ALERT_1_G ALERT: Large difference may be due to a
            symmetry error - see SYMMG tests
           From the CIF: _cell_formula_units_Z    4
           From the CIF: _chemical_formula_sum  C27.50 H29 Cl5 N6 Ni
           TEST: Compare cell contents of formula and atom_site data

           atom    Z*formula  cif sites diff
           C        110.00    106.00   4.00
           H        116.00    108.00   8.00
           Cl        20.00     12.00   8.00
           N         24.00     24.00   0.00
           Ni         4.00      4.00   0.00
PLAT041_ALERT_1_G Calc. and Reported SumFormula    Strings  Differ     Please Check
              Calc: C53 H54 Cl6 N12 Ni2
              Rep.: C27.50 H29 Cl5 N6 Ni
PLAT042_ALERT_1_G Calc. and Reported MoietyFormula Strings  Differ     Please Check
              Calc: 2(C26 H26 Cl2 N6 Ni), C H2 Cl2
              Rep.: C26 H26 Cl2 N6 Ni, 0.5(C H2 Cl2), 1[CH2CL2]
PLAT045_ALERT_1_G Calculated and Reported Z Differ by a Factor ...      0.500 Check
PLAT051_ALERT_1_G Mu(calc) and Mu(cif) Ratio Differs from 1.0 by .      16.25 %
PLAT154_ALERT_1_G The s.u.'s on the Cell Angles are Equal ..(Note)      0.004 Degree
PLAT434_ALERT_2_G Short Inter HL..HL Contact Cl7      ..Cl7      .       3.39 Ang.
                                                 3-x,-y,2-z  =     2_857 Check
PLAT606_ALERT_4_G Solvent Accessible VOID(S) in Structure .......           ! Info
PLAT790_ALERT_4_G Centre of Gravity not Within Unit Cell: Resd.  #          2 Note
              C26 H26 Cl2 N6 Ni
PLAT790_ALERT_4_G Centre of Gravity not Within Unit Cell: Resd.  #          3 Note
              C H2 Cl2
PLAT794_ALERT_5_G Tentative Bond Valency for Ni1       (II)      .       1.98 Info
PLAT794_ALERT_5_G Tentative Bond Valency for Ni2       (II)      .       2.00 Info
```

```
PLAT868_ALERT_4_G ALERTS Due to the Use of _smtbx_masks Suppressed          ! Info
PLAT912_ALERT_4_G Missing # of FCF Reflections Above STh/L=  0.600        101 Note
PLAT913_ALERT_3_G Missing # of Very Strong Reflections in FCF ....          1 Note
                1  0  1,
PLAT969_ALERT_5_G The 'Henn et al.' R-Factor-gap value ..........       3.746 Note
              Predicted wR2: Based on SigI**2  3.25 or SHELX Weight 11.28
PLAT978_ALERT_2_G Number C-C Bonds with Positive Residual Density.          6 Info
PLAT992_ALERT_5_G Repd & Actual _reflns_number_gt Values Differ by          3 Check
```

---

```
   0 ALERT level A = Most likely a serious problem - resolve or explain
   0 ALERT level B = A potentially serious problem, consider carefully
   3 ALERT level C = Check. Ensure it is not caused by an omission or oversight
  20 ALERT level G = General information/check it is not something unexpected

   8 ALERT type 1 CIF construction/syntax error, inconsistent or missing data
   3 ALERT type 2 Indicator that the structure model may be wrong or deficient
   3 ALERT type 3 Indicator that the structure quality may be low
   5 ALERT type 4 Improvement, methodology, query or suggestion
   4 ALERT type 5 Informative message, check
```

---

---

It is advisable to attempt to resolve as many as possible of the alerts in all categories. Often the minor alerts point to easily fixed oversights, errors and omissions in your CIF or refinement strategy, so attention to these fine details can be worthwhile. In order to resolve some of the more serious problems it may be necessary to carry out additional measurements or structure refinements. However, the purpose of your study may justify the reported deviations and the more serious of these should normally be commented upon in the discussion or experimental section of a paper or in the "special_details" fields of the CIF. checkCIF was carefully designed to identify outliers and unusual parameters, but every test has its limitations and alerts that are not important in a particular case may appear. Conversely, the absence of alerts does not guarantee there are no aspects of the results needing attention. It is up to the individual to critically assess their own results and, if necessary, seek expert advice.

**Publication of your CIF in IUCr journals**

A basic structural check has been run on your CIF. These basic checks will be run on all CIFs submitted for publication in IUCr journals (*Acta Crystallographica*, *Journal of Applied Crystallography*, *Journal of Synchrotron Radiation*); however, if you intend to submit to *Acta Crystallographica Section C* or *E* or *IUCrData*, you should make sure that full publication checks are run on the final version of your CIF prior to submission.

**Publication of your CIF in other journals**

Please refer to the *Notes for Authors* of the relevant journal for any special instructions relating to CIF submission.

---

**PLATON version of 02/02/2025; check.def file version of 02/02/2025**

**Datablock mo_2025_ee203_0m** - ellipsoid plot

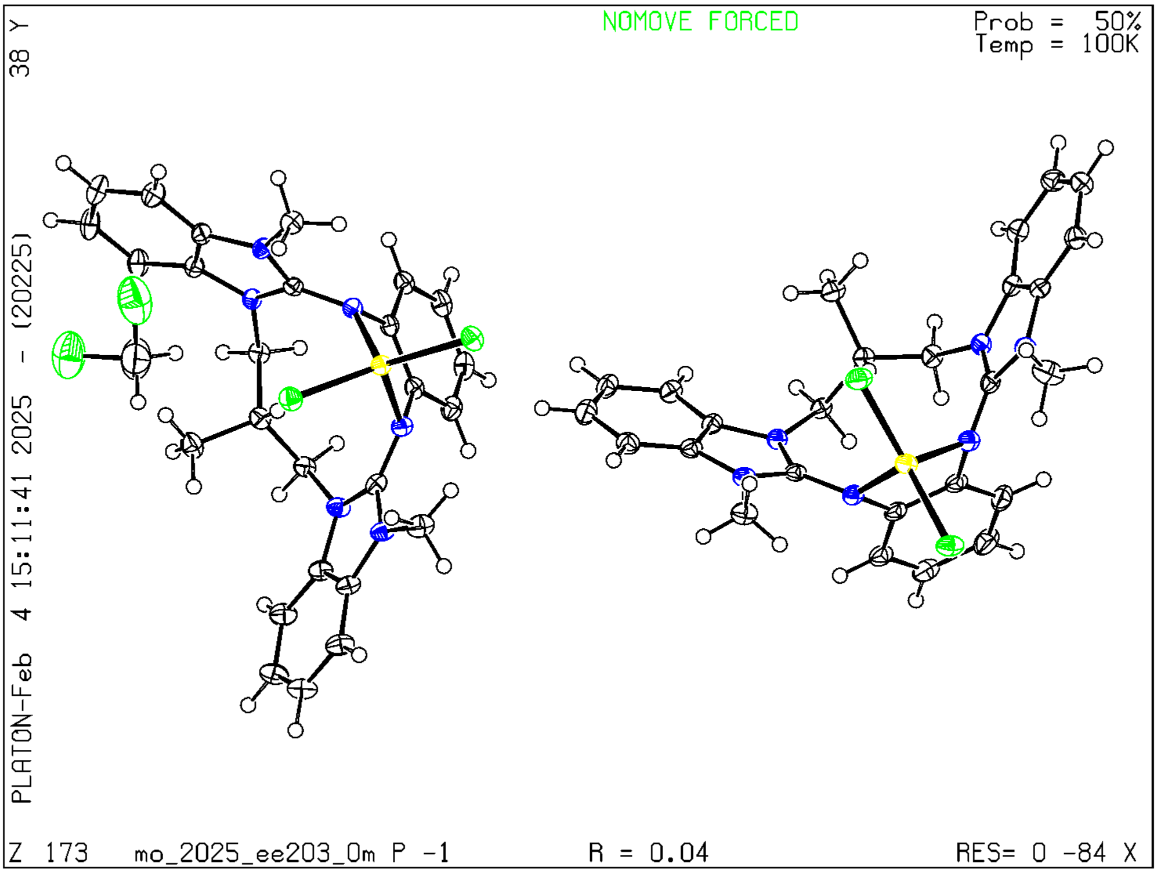

Supplement: Supplementary file 2 — Supporting Information [file CHEM-31-e02457-s002.zip › mo_2025_ee203_0m_cifreport.pdf]
