# Supplementary material for: Steric Restraints in Redox‐Active Guanidine Ligands and Their Impact on Coordination Chemistry
Source: Chemistry. 2025 Oct 25;31(66):e02457. doi: 10.1002/chem.202502457 (PMC12648461; doi:10.1002/chem.202502457)

checkCIF/PLATON report

Structure factors have been supplied for datablock(s) mo_2025_ee230_4_0m

THIS REPORT IS FOR GUIDANCE ONLY. IF USED AS PART OF A REVIEW PROCEDURE FOR PUBLICATION, IT SHOULD NOT REPLACE THE EXPERTISE OF AN EXPERIENCED CRYSTALLOGRAPHIC REFEREE.

No syntax errors found.   CIF dictionary   Interpreting this report

Datablock: mo_2025_ee230_4_0m

```
Bond precision:   C-C = 0.0035 A          Wavelength=0.71073

Cell:           a=8.9238(13)      b=9.3438(13)       c=12.7315(19)
                alpha=94.275(5)   beta=108.202(5)    gamma=94.576(5)
Temperature:    100 K
```

	Calculated	Reported
Volume	999.7(3)	999.7(3)
Space group	P -1	P-1
Hall group	-P 1	-P 1
Moiety formula	C25 H24 N6	C25 H24 N6
Sum formula	C25 H24 N6	C25 H24 N6
Mr	408.50	408.50
Dx,g cm-3	1.357	1.357
Z	2	2
Mu (mm-1)	0.084	0.084
F000	432.0	432.0
F000’	432.13	
h,k,lmax	11,11,15	11,11,15
Nref	4137	4103
Tmin,Tmax	0.986,0.995	0.626,0.745
Tmin’	0.986	

```
Correction method= # Reported T Limits: Tmin=0.626 Tmax=0.745
AbsCorr = MULTI-SCAN

Data completeness= 0.992          Theta(max)= 26.470

R(reflections)= 0.0601( 3029)         wR2(reflections)= 0.1756( 4103)

S = 1.099                  Npar= 282
```

The following ALERTS were generated. Each ALERT has the format
test-name_ALERT_alert-type_alert-level.
Click on the hyperlinks for more details of the test.

Alert level C

```
PLAT906_ALERT_3_C Large K Value in the Analysis of Variance ......      3.022 Check
PLAT911_ALERT_3_C Missing FCF Refl Between Thmin & STh/L=    0.600          7 Report
                7  6  0,   7  7  0,  -7 -7  1,  -7 -6  1,  -7 -7  2,  -6  1  3,
               -3 -9  5,
```

Alert level G

```
PLAT154_ALERT_1_G The s.u.'s on the Cell Angles are Equal ..(Note)      0.005 Degree
PLAT910_ALERT_3_G Missing FCF Reflection(s) Below Theta(Min)[Deg]=       2.20 Note
                0  0  1,
PLAT912_ALERT_4_G Missing # of FCF Reflections Above STh/L=  0.600         26 Note
PLAT933_ALERT_2_G Number of HKL-OMIT Records in Embedded .res File          1 Note
               -3 -9  5,
PLAT969_ALERT_5_G The 'Henn et al.' R-Factor-gap value ..........      2.982 Note
              Predicted wR2: Based on SigI**2  5.89 or SHELX Weight 15.97
PLAT978_ALERT_2_G Number C-C Bonds with Positive Residual Density.          2 Info
```

0 **ALERT level A** = Most likely a serious problem - resolve or explain
0 **ALERT level B** = A potentially serious problem, consider carefully
2 **ALERT level C** = Check. Ensure it is not caused by an omission or oversight
6 **ALERT level G** = General information/check it is not something unexpected

1 ALERT type 1 CIF construction/syntax error, inconsistent or missing data
2 ALERT type 2 Indicator that the structure model may be wrong or deficient
3 ALERT type 3 Indicator that the structure quality may be low
1 ALERT type 4 Improvement, methodology, query or suggestion
1 ALERT type 5 Informative message, check

It is advisable to attempt to resolve as many as possible of the alerts in all categories. Often the minor alerts point to easily fixed oversights, errors and omissions in your CIF or refinement strategy, so attention to these fine details can be worthwhile. In order to resolve some of the more serious problems it may be necessary to carry out additional measurements or structure refinements. However, the purpose of your study may justify the reported deviations and the more serious of these should normally be commented upon in the discussion or experimental section of a paper or in the "special_details" fields of the CIF. checkCIF was carefully designed to identify outliers and unusual parameters, but every test has its limitations and alerts that are not important in a particular case may appear. Conversely, the absence of alerts does not guarantee there are no aspects of the results needing attention. It is up to the individual to critically assess their own results and, if necessary, seek expert advice.

Publication of your CIF in IUCr journals

A basic structural check has been run on your CIF. These basic checks will be run on all CIFs submitted for publication in IUCr journals (*Acta Crystallographica*, *Journal of Applied Crystallography*, *Journal of Synchrotron Radiation*); however, if you intend to submit to *Acta Crystallographica Section C* or *E* or *IUCrData*, you should make sure that full publication checks are run on the final version of your CIF prior to submission.

Publication of your CIF in other journals

Please refer to the *Notes for Authors* of the relevant journal for any special instructions relating to CIF submission.

PLATON version of 04/06/2025; check.def file version of 30/05/2025

Datablock mo_2025_ee230_4_0m - ellipsoid plot

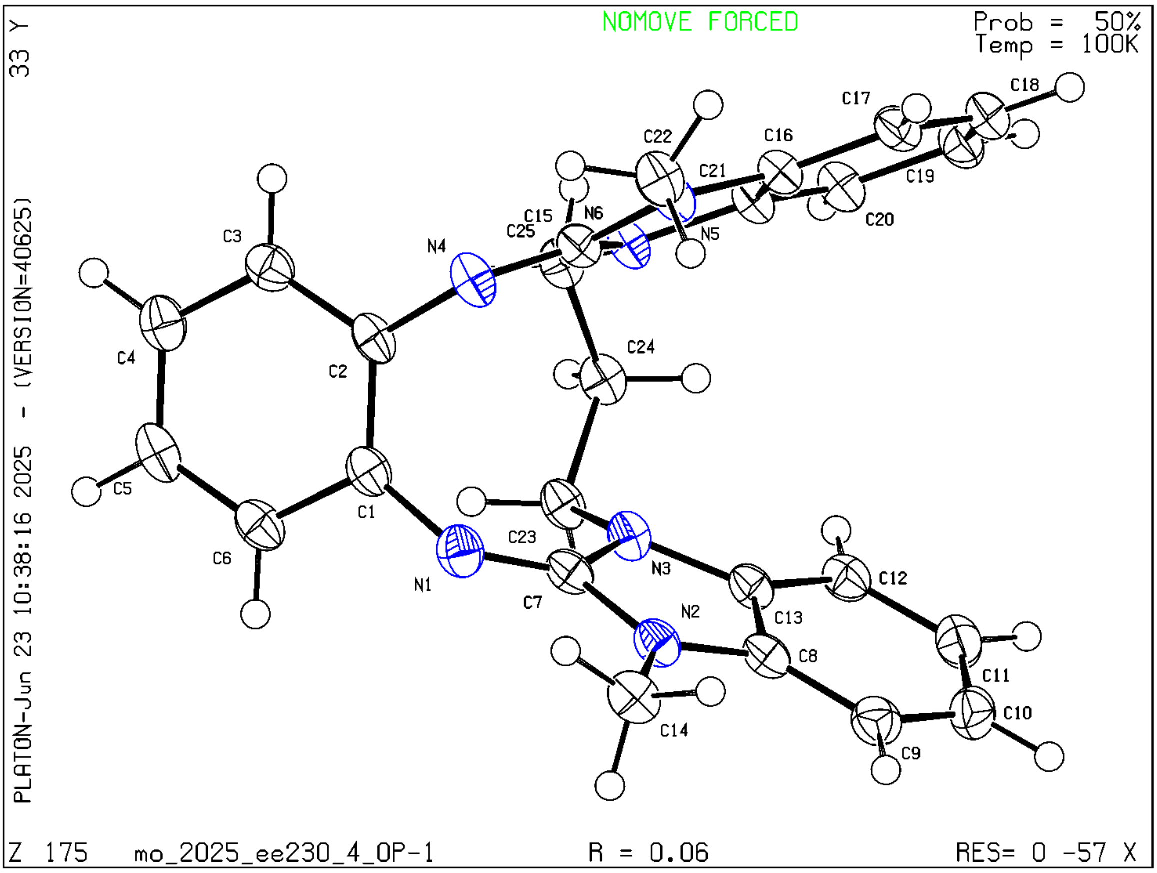

Supplement: Supplementary file 2 — Supporting Information [file CHEM-31-e02457-s002.zip › mo_2025_ee230_4_0m_cifreport.pdf]
