# Supplementary material for: Steric Restraints in Redox‐Active Guanidine Ligands and Their Impact on Coordination Chemistry
Source: Chemistry. 2025 Oct 25;31(66):e02457. doi: 10.1002/chem.202502457 (PMC12648461; doi:10.1002/chem.202502457)

checkCIF/PLATON report

Structure factors have been supplied for datablock(s) mo_2023_eehk10_8_0m

THIS REPORT IS FOR GUIDANCE ONLY. IF USED AS PART OF A REVIEW PROCEDURE FOR PUBLICATION, IT SHOULD NOT REPLACE THE EXPERTISE OF AN EXPERIENCED CRYSTALLOGRAPHIC REFEREE.

No syntax errors found.   CIF dictionary   Interpreting this report

Datablock: mo_2023_eehk10_8_0m

```
Bond precision:   C-C = 0.0015 A          Wavelength=0.71073

Cell:               a=9.090(2)      b=13.047(3)         c=15.826(3)
                    alpha=90        beta=93.343(8)      gamma=90
Temperature:        100 K

                  Calculated                  Reported
Volume            1873.7(7)                   1873.8(7)
Space group       P 21/c                      P 1 21/c 1
Hall group        -P 2ybc                     -P 2ybc
Moiety formula    C24 H22 N6                  C24 H22 N6
Sum formula       C24 H22 N6                  C24 H22 N6
Mr                394.48                      394.47
Dx,g cm-3         1.398                       1.398
Z                 4                           4
Mu (mm-1)         0.087                       0.087
F000              832.0                       832.0
F000'             832.25
h,k,lmax          11,16,20                    11,16,20
Nref              4309                        4306
Tmin,Tmax         0.974,0.989                 0.701,0.732
Tmin'             0.974

Correction method= # Reported T Limits: Tmin=0.701 Tmax=0.732
AbsCorr = MULTI-SCAN

Data completeness= 0.999            Theta(max)= 27.494

                                                      wR2(reflections)=
R(reflections)= 0.0425( 3964)                         0.1146( 4306)

S = 1.031                     Npar= 273
```

The following ALERTS were generated. Each ALERT has the format
test-name_ALERT_alert-type_alert-level.
Click on the hyperlinks for more details of the test.

Alert level C

```
PLAT911_ALERT_3_C Missing FCF Refl Between Thmin & STh/L=    0.600           4 Report
```

Alert level G

```
PLAT933_ALERT_2_G Number of HKL-OMIT Records in Embedded .res File           2 Note
PLAT967_ALERT_5_G Note: Two-Theta Cutoff Value in Embedded .res ..        55.0 Degree
PLAT978_ALERT_2_G Number C-C Bonds with Positive Residual Density.          11 Info
```

0 **ALERT level A** = Most likely a serious problem - resolve or explain
0 **ALERT level B** = A potentially serious problem, consider carefully
1 **ALERT level C** = Check. Ensure it is not caused by an omission or oversight
3 **ALERT level G** = General information/check it is not something unexpected

0 ALERT type 1 CIF construction/syntax error, inconsistent or missing data
2 ALERT type 2 Indicator that the structure model may be wrong or deficient
1 ALERT type 3 Indicator that the structure quality may be low
0 ALERT type 4 Improvement, methodology, query or suggestion
1 ALERT type 5 Informative message, check

It is advisable to attempt to resolve as many as possible of the alerts in all categories. Often the minor alerts point to easily fixed oversights, errors and omissions in your CIF or refinement strategy, so attention to these fine details can be worthwhile. In order to resolve some of the more serious problems it may be necessary to carry out additional measurements or structure refinements. However, the purpose of your study may justify the reported deviations and the more serious of these should normally be commented upon in the discussion or experimental section of a paper or in the "special_details" fields of the CIF. checkCIF was carefully designed to identify outliers and unusual parameters, but every test has its limitations and alerts that are not important in a particular case may appear. Conversely, the absence of alerts does not guarantee there are no aspects of the results needing attention. It is up to the individual to critically assess their own results and, if necessary, seek expert advice.

Publication of your CIF in IUCr journals

A basic structural check has been run on your CIF. These basic checks will be run on all CIFs submitted for publication in IUCr journals (*Acta Crystallographica*, *Journal of Applied Crystallography*, *Journal of Synchrotron Radiation*); however, if you intend to submit to *Acta Crystallographica Section C* or *E* or *IUCrData*, you should make sure that full publication checks are run on the final version of your CIF prior to submission.

Publication of your CIF in other journals

Please refer to the *Notes for Authors* of the relevant journal for any special instructions relating to CIF submission.

PLATON version of 10/05/2023; check.def file version of 10/05/2023

Datablock mo_2023_eehk10_8_0m - ellipsoid plot

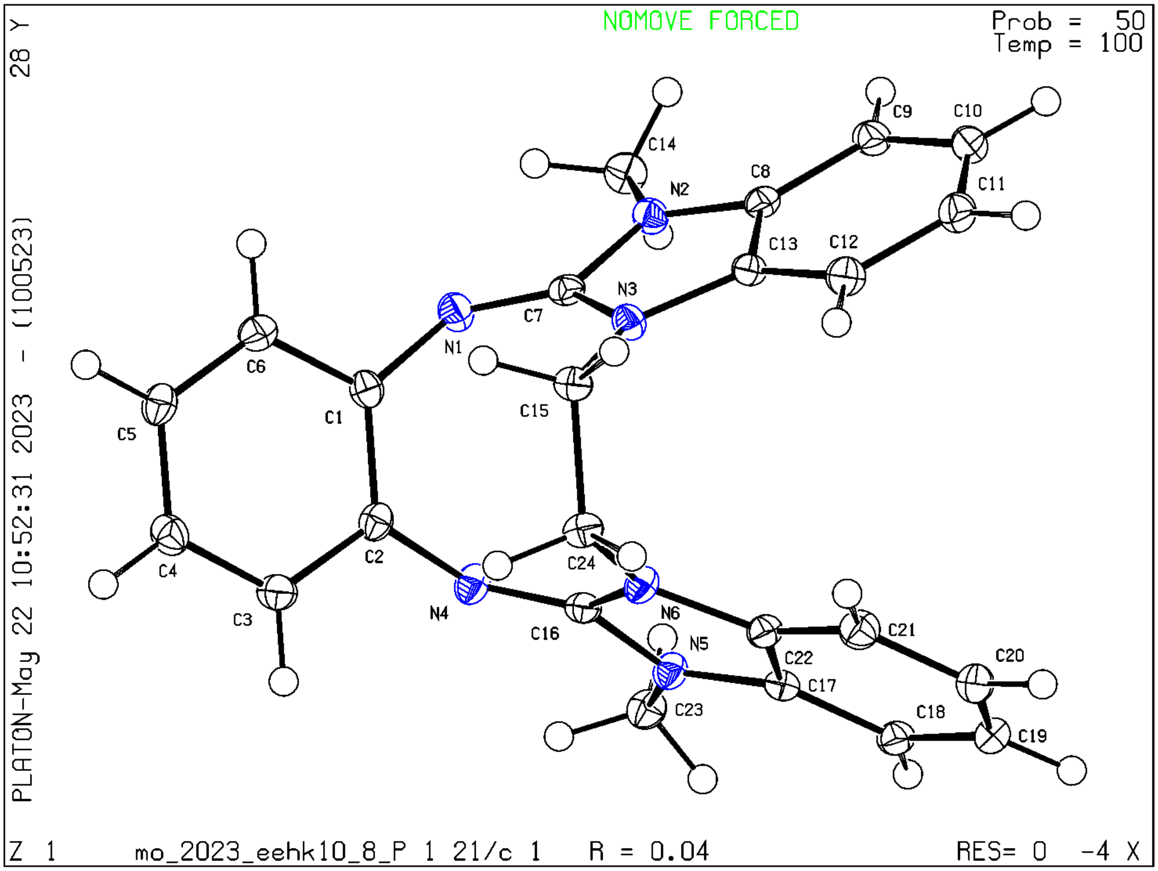

Supplement: Supplementary file 2 — Supporting Information [file CHEM-31-e02457-s002.zip › mo_2023_eehk10_8_0m_cifreport.pdf]
