# Supplementary material for: Steric Restraints in Redox‐Active Guanidine Ligands and Their Impact on Coordination Chemistry
Source: Chemistry. 2025 Oct 25;31(66):e02457. doi: 10.1002/chem.202502457 (PMC12648461; doi:10.1002/chem.202502457)

checkCIF/PLATON report

Structure factors have been supplied for datablock(s) mo_2023_eehk26_0ma

THIS REPORT IS FOR GUIDANCE ONLY. IF USED AS PART OF A REVIEW PROCEDURE FOR PUBLICATION, IT SHOULD NOT REPLACE THE EXPERTISE OF AN EXPERIENCED CRYSTALLOGRAPHIC REFEREE.

No syntax errors found. CIF dictionary Interpreting this report

Datablock: mo_2023_eehk26_0ma

```
Bond precision:   C-C = 0.0054 A          Wavelength=0.71073

Cell:             a=10.959(4)    b=12.521(5)          c=17.321(7)
                  alpha=90       beta=102.229(15)     gamma=90
Temperature:      100 K

                 Calculated                   Reported
Volume           2322.8(16)                   2322.9(16)
Space group      P 21/n                       P 1 21/n 1
Hall group       -P 2yn                       -P 2yn
Moiety formula   C24 H23 N6, F6 P             F6 P, C24 H23 N6
Sum formula      C24 H23 F6 N6 P              C24 H23 F6 N6 P
Mr               540.45                       540.45
Dx,g cm-3        1.546                        1.545
Z                4                            4
Mu (mm-1)        0.194                        0.194
F000             1112.0                       1112.0
F000'            1113.05
h,k,lmax         13,15,21                     13,15,21
Nref             4797                         4766
Tmin,Tmax        0.966,0.990                  0.604,0.745
Tmin'            0.966

Correction method= # Reported T Limits: Tmin=0.604 Tmax=0.745
AbsCorr = MULTI-SCAN

Data completeness= 0.994          Theta(max)= 26.473

                                                     wR2(reflections)=
R(reflections)= 0.0629( 3053)                        0.1903( 4766)

S = 1.028                    Npar= 395
```

The following ALERTS were generated. Each ALERT has the format
test-name_ALERT_alert-type_alert-level.
Click on the hyperlinks for more details of the test.

Alert level C

```
PLAT042_ALERT_1_C Calc. and Reported MoietyFormula Strings  Differ     Please Check
PLAT340_ALERT_3_C Low Bond Precision on  C-C Bonds ...............    0.00537 Ang.
PLAT767_ALERT_4_C INS Embedded LIST 6 Instruction Should be LIST 4     Please Check
PLAT905_ALERT_3_C Negative K value in the Analysis of Variance ...     -2.050 Report
PLAT905_ALERT_3_C Negative K value in the Analysis of Variance ...     -0.132 Report
```

Alert level G

```
PLAT002_ALERT_2_G Number of Distance or Angle Restraints on AtSite          7 Note
PLAT003_ALERT_2_G Number of Uiso or Uij Restrained non-H Atoms ...          7 Report
PLAT172_ALERT_4_G The CIF-Embedded .res File Contains DFIX Records          1 Report
PLAT176_ALERT_4_G The CIF-Embedded .res File Contains SADI Records          3 Report
PLAT178_ALERT_4_G The CIF-Embedded .res File Contains SIMU Records          2 Report
PLAT187_ALERT_4_G The CIF-Embedded .res File Contains RIGU Records          1 Report
PLAT191_ALERT_3_G A Non-default SADI Restraint Value has been used     0.0400 Report
PLAT191_ALERT_3_G A Non-default SADI Restraint Value has been used     0.0500 Report
PLAT244_ALERT_4_G Low    'Solvent' Ueq as Compared to Neighbors of         P2 Check
PLAT300_ALERT_4_G Atom Site Occupancy of F1         Constrained at        0.9 Check
PLAT300_ALERT_4_G Atom Site Occupancy of F2         Constrained at        0.9 Check
PLAT300_ALERT_4_G Atom Site Occupancy of F3         Constrained at        0.9 Check
PLAT300_ALERT_4_G Atom Site Occupancy of F4         Constrained at        0.9 Check
PLAT300_ALERT_4_G Atom Site Occupancy of F5         Constrained at        0.9 Check
PLAT300_ALERT_4_G Atom Site Occupancy of F6         Constrained at        0.9 Check
PLAT300_ALERT_4_G Atom Site Occupancy of F7         Constrained at        0.1 Check
PLAT300_ALERT_4_G Atom Site Occupancy of F8         Constrained at        0.1 Check
PLAT300_ALERT_4_G Atom Site Occupancy of F9         Constrained at        0.1 Check
PLAT300_ALERT_4_G Atom Site Occupancy of F10        Constrained at        0.1 Check
PLAT300_ALERT_4_G Atom Site Occupancy of F11        Constrained at        0.1 Check
PLAT300_ALERT_4_G Atom Site Occupancy of F12        Constrained at        0.1 Check
PLAT302_ALERT_4_G Anion/Solvent/Minor-Residue Disorder (Resd  2  )        86% Note
PLAT432_ALERT_2_G Short Inter X...Y Contact  F9       ..C22      .       2.90 Ang.
                                  1/2-x,-1/2+y,3/2-z  =      2_546 Check
PLAT432_ALERT_2_G Short Inter X...Y Contact  F12      ..C7       .       2.67 Ang.
                                          -x,1-y,1-z  =      3_566 Check
PLAT860_ALERT_3_G Number of Least-Squares Restraints .............        151 Note
PLAT912_ALERT_4_G Missing # of FCF Reflections Above STh/L=  0.600         32 Note
PLAT978_ALERT_2_G Number C-C Bonds with Positive Residual Density.          0 Info
```

0 **ALERT level A** = Most likely a serious problem - resolve or explain
0 **ALERT level B** = A potentially serious problem, consider carefully
5 **ALERT level C** = Check. Ensure it is not caused by an omission or oversight
27 **ALERT level G** = General information/check it is not something unexpected

1 ALERT type 1 CIF construction/syntax error, inconsistent or missing data
5 ALERT type 2 Indicator that the structure model may be wrong or deficient
6 ALERT type 3 Indicator that the structure quality may be low
20 ALERT type 4 Improvement, methodology, query or suggestion
0 ALERT type 5 Informative message, check

It is advisable to attempt to resolve as many as possible of the alerts in all categories. Often the minor alerts point to easily fixed oversights, errors and omissions in your CIF or refinement strategy, so attention to these fine details can be worthwhile. In order to resolve some of the more serious problems it may be necessary to carry out additional measurements or structure refinements. However, the purpose of your study may justify the reported deviations and the more serious of these should normally be commented upon in the discussion or experimental section of a paper or in the "special_details" fields of the CIF. checkCIF was carefully designed to identify outliers and unusual parameters, but every test has its limitations and alerts that are not important in a particular case may appear. Conversely, the absence of alerts does not guarantee there are no aspects of the results needing attention. It is up to the individual to critically assess their own results and, if necessary, seek expert advice.

Publication of your CIF in IUCr journals

A basic structural check has been run on your CIF. These basic checks will be run on all CIFs submitted for publication in IUCr journals (*Acta Crystallographica*, *Journal of Applied Crystallography*, *Journal of Synchrotron Radiation*); however, if you intend to submit to *Acta Crystallographica Section C* or *E* or *IUCrData*, you should make sure that full publication checks are run on the final version of your CIF prior to submission.

Publication of your CIF in other journals

Please refer to the *Notes for Authors* of the relevant journal for any special instructions relating to CIF submission.

PLATON version of 10/05/2023; check.def file version of 10/05/2023

Datablock mo_2023_eehk26_0ma - ellipsoid plot

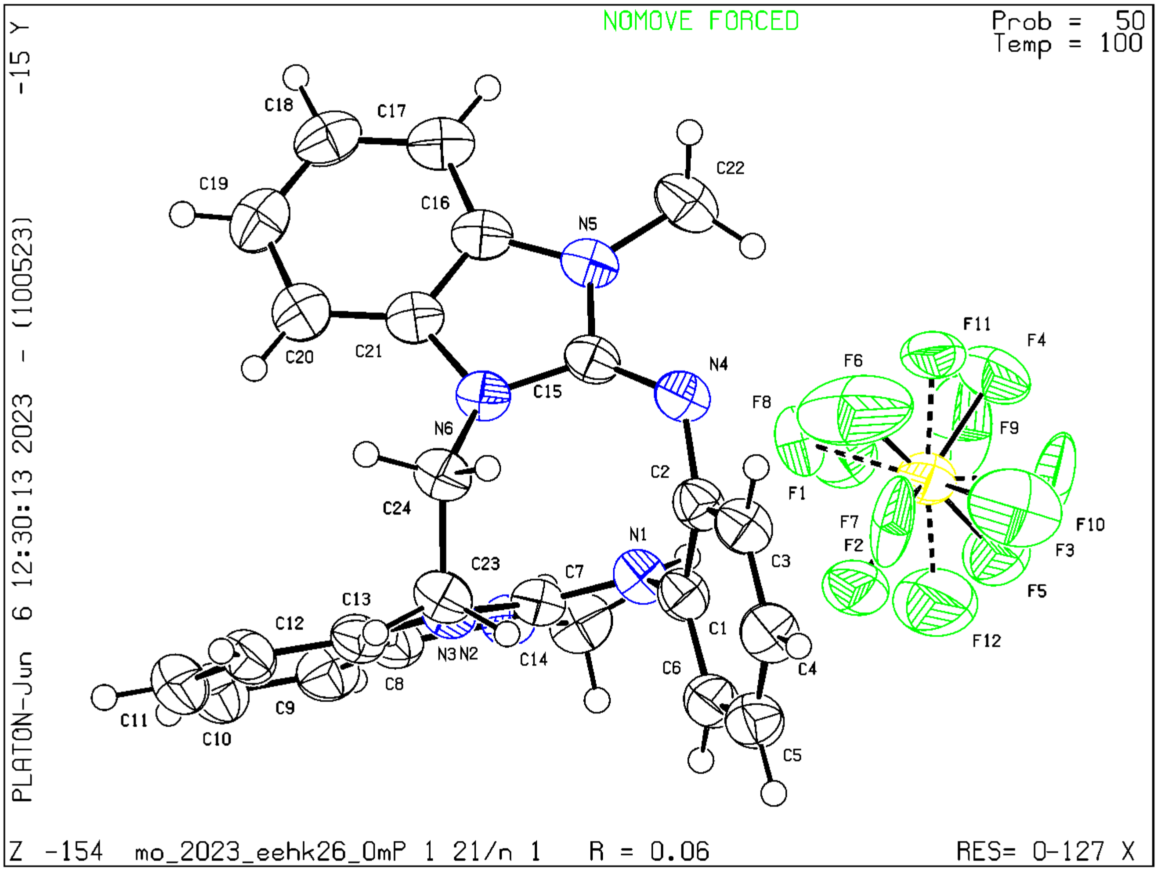

Supplement: Supplementary file 2 — Supporting Information [file CHEM-31-e02457-s002.zip › mo_2023_eehk26_0ma_cifreport.pdf]
