# Supplementary material for: Steric Restraints in Redox‐Active Guanidine Ligands and Their Impact on Coordination Chemistry
Source: Chemistry. 2025 Oct 25;31(66):e02457. doi: 10.1002/chem.202502457 (PMC12648461; doi:10.1002/chem.202502457)

checkCIF/PLATON report

Structure factors have been supplied for datablock(s) mo_2024_eeml42_0

THIS REPORT IS FOR GUIDANCE ONLY. IF USED AS PART OF A REVIEW PROCEDURE FOR PUBLICATION, IT SHOULD NOT REPLACE THE EXPERTISE OF AN EXPERIENCED CRYSTALLOGRAPHIC REFEREE.

No syntax errors found. CIF dictionary Interpreting this report

Datablock: mo_2024_eeml42_0

Bond precision: C-C = 0.0036 A Wavelength=0.71073

Cell:	a=15.5775(12)	b=8.6923(7)	c=20.4737(17)
	alpha=90	beta=91.013(3)	gamma=90
Temperature:	100 K		

	Calculated	Reported
Volume	2771.8(4)	2771.8(4)
Space group	P 21/c	P 1 21/c 1
Hall group	-P 2ybc	-P 2ybc
Moiety formula	C26 H28 N6, 2(Cl) [+ solvent]	C26 H28 N6, 2(Cl), 1.2[CH3CN]
Sum formula	C26 H28 Cl2 N6 [+ solvent]	C28.40 H31.60 Cl2 N7.20
Mr	495.44	544.71
Dx,g cm-3	1.187	1.305
Z	4	4
Mu (mm-1)	0.258	0.266
F000	1040.0	1146.0
F000’	1041.46	
h,k,lmax	19,10,25	19,10,25
Nref	5741	5739
Tmin,Tmax	0.968,0.974	0.650,0.746
Tmin’	0.961	

Correction method= # Reported T Limits: Tmin=0.650 Tmax=0.746
AbsCorr = MULTI-SCAN

Data completeness= 1.000 Theta(max)= 26.499

R(reflections)= 0.0485(4120) wR2(reflections)= 0.1314(5739)

S = 1.028 Npar= 328

The following ALERTS were generated. Each ALERT has the format
test-name_ALERT_alert-type_alert-level.
Click on the hyperlinks for more details of the test.

Alert level C

```
PLAT905_ALERT_3_C Negative K value in the Analysis of Variance ...     -1.545 Report
```

Alert level G

```
FORMU01_ALERT_2_G  There is a discrepancy between the atom counts in the
            _chemical_formula_sum and the formula from the _atom_site* data.
            Atom count from _chemical_formula_sum:C28.4 H31.6 Cl2 N7.2
            Atom count from the _atom_site data:  C26 H28 Cl2 N6
CELLZ01_ALERT_1_G Difference between formula and atom_site contents detected.
CELLZ01_ALERT_1_G ALERT: Large difference may be due to a
            symmetry error - see SYMMG tests
           From the CIF: _cell_formula_units_Z    4
           From the CIF: _chemical_formula_sum  C28.40 H31.60 Cl2 N7.20
           TEST: Compare cell contents of formula and atom_site data

           atom    Z*formula  cif sites diff
           C        113.60    104.00    9.60
           H        126.40    112.00   14.40
           Cl         8.00      8.00    0.00
           N         28.80     24.00    4.80
PLAT002_ALERT_2_G Number of Distance or Angle Restraints on AtSite          4 Note
PLAT041_ALERT_1_G Calc. and Reported SumFormula    Strings  Differ     Please Check
              Calc: C26 H28 Cl2 N6
              Rep.: C28.40 H31.60 Cl2 N7.20
PLAT042_ALERT_1_G Calc. and Reported MoietyFormula Strings  Differ     Please Check
              Calc: C26 H28 N6, 2(Cl)
              Rep.: C26 H28 N6, 2(Cl), 1.2[CH3CN]
PLAT172_ALERT_4_G The CIF-Embedded .res File Contains DFIX Records          1 Report
PLAT300_ALERT_4_G Atom Site Occupancy of Cl2        Constrained at       0.95 Check
PLAT300_ALERT_4_G Atom Site Occupancy of Cl3        Constrained at       0.05 Check
PLAT302_ALERT_4_G Anion/Solvent/Minor-Residue Disorder (Resd    3)       100% Note
PLAT302_ALERT_4_G Anion/Solvent/Minor-Residue Disorder (Resd    4)       100% Note
PLAT304_ALERT_4_G Non-Integer Number of Atoms in ..... (Resd    3)       0.95 Check
PLAT304_ALERT_4_G Non-Integer Number of Atoms in ..... (Resd    4)       0.05 Check
PLAT605_ALERT_4_G Largest Solvent Accessible VOID in the Structure        197 A**3
PLAT860_ALERT_3_G Number of Least-Squares Restraints ............           2 Note
PLAT868_ALERT_4_G ALERTS Due to the Use of _smtbx_masks Suppressed          ! Info
PLAT910_ALERT_3_G Missing # of FCF Reflection(s) Below Theta(Min).          1 Note
                1  0  0,
PLAT967_ALERT_5_G Note: Two-Theta Cutoff Value in Embedded .res ..       53.0 Degree
PLAT969_ALERT_5_G The 'Henn et al.' R-Factor-gap value ..........        2.22 Note
              Predicted wR2: Based on SigI**2  5.92 or SHELX Weight 13.16
PLAT978_ALERT_2_G Number C-C Bonds with Positive Residual Density.          4 Info
```

0 **ALERT level A** = Most likely a serious problem - resolve or explain
0 **ALERT level B** = A potentially serious problem, consider carefully
1 **ALERT level C** = Check. Ensure it is not caused by an omission or oversight
20 **ALERT level G** = General information/check it is not something unexpected

```
4 ALERT type 1 CIF construction/syntax error, inconsistent or missing data
3 ALERT type 2 Indicator that the structure model may be wrong or deficient
3 ALERT type 3 Indicator that the structure quality may be low
9 ALERT type 4 Improvement, methodology, query or suggestion
2 ALERT type 5 Informative message, check
```

It is advisable to attempt to resolve as many as possible of the alerts in all categories. Often the minor alerts point to easily fixed oversights, errors and omissions in your CIF or refinement strategy, so attention to these fine details can be worthwhile. In order to resolve some of the more serious problems it may be necessary to carry out additional measurements or structure refinements. However, the purpose of your study may justify the reported deviations and the more serious of these should normally be commented upon in the discussion or experimental section of a paper or in the "special_details" fields of the CIF. checkCIF was carefully designed to identify outliers and unusual parameters, but every test has its limitations and alerts that are not important in a particular case may appear. Conversely, the absence of alerts does not guarantee there are no aspects of the results needing attention. It is up to the individual to critically assess their own results and, if necessary, seek expert advice.

Publication of your CIF in IUCr journals

A basic structural check has been run on your CIF. These basic checks will be run on all CIFs submitted for publication in IUCr journals (*Acta Crystallographica*, *Journal of Applied Crystallography*, *Journal of Synchrotron Radiation*); however, if you intend to submit to *Acta Crystallographica Section C* or *E* or *IUCrData*, you should make sure that full publication checks are run on the final version of your CIF prior to submission.

Publication of your CIF in other journals

Please refer to the *Notes for Authors* of the relevant journal for any special instructions relating to CIF submission.

PLATON version of 06/01/2024; check.def file version of 05/01/2024

Datablock mo_2024_eeml42_0 - ellipsoid plot

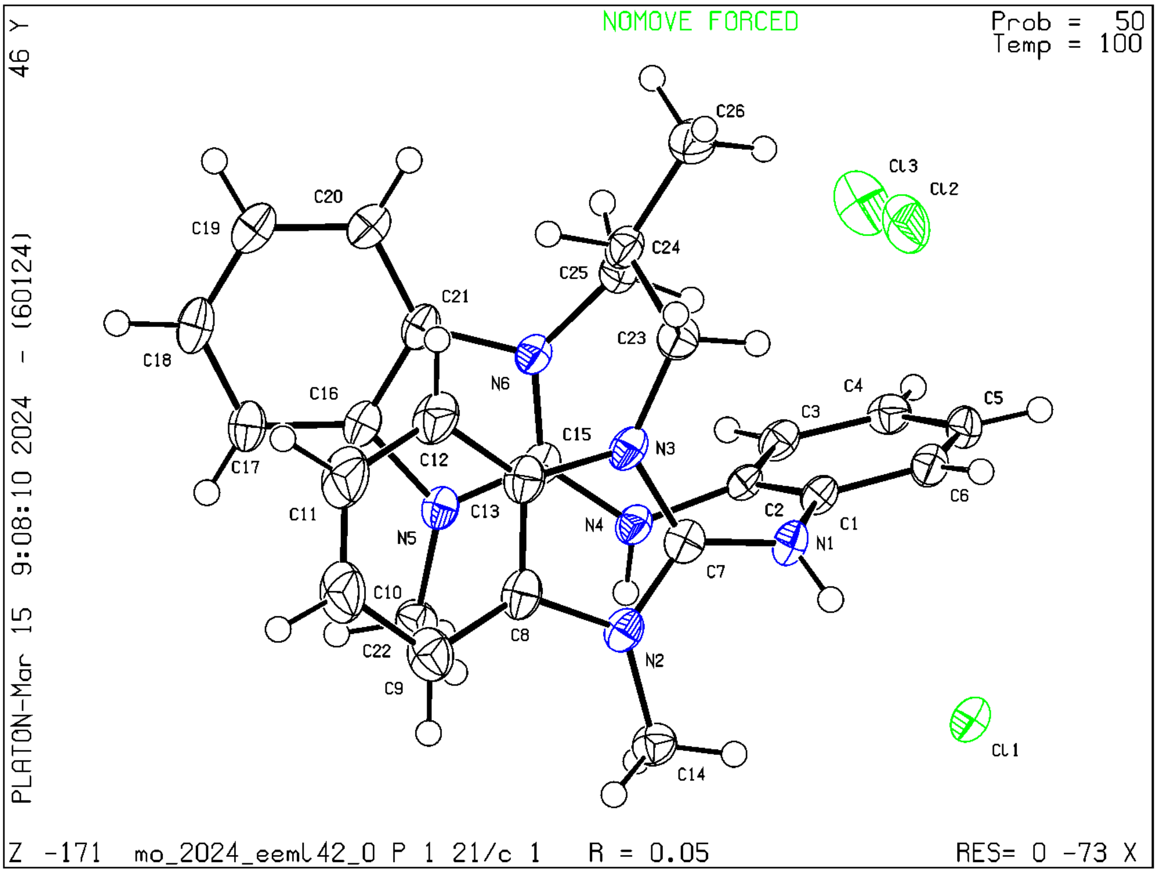

Supplement: Supplementary file 2 — Supporting Information [file CHEM-31-e02457-s002.zip › mo_2024_eeml42_0_cifreport.pdf]
