# Supplementary material for: Steric Restraints in Redox‐Active Guanidine Ligands and Their Impact on Coordination Chemistry
Source: Chemistry. 2025 Oct 25;31(66):e02457. doi: 10.1002/chem.202502457 (PMC12648461; doi:10.1002/chem.202502457)

checkCIF/PLATON report

Structure factors have been supplied for datablock(s) mo_2023_eehk10_10b_0ma

THIS REPORT IS FOR GUIDANCE ONLY. IF USED AS PART OF A REVIEW PROCEDURE FOR PUBLICATION, IT SHOULD NOT REPLACE THE EXPERTISE OF AN EXPERIENCED CRYSTALLOGRAPHIC REFEREE.

No syntax errors found. CIF dictionary Interpreting this report

Datablock: mo_2023_eehk10_10b_0ma

Bond precision: C-C = 0.0050 A Wavelength=0.71073

Cell:	a=11.618(5)	b=14.715(6)	c=15.198(7)
	alpha=90	beta=111.498(15)	gamma=90
Temperature:	100 K		

	Calculated	Reported
Volume	2417.5(18)	2417.5(18)
Space group	P 21/c	P 1 21/c 1
Hall group	-P 2ybc	-P 2ybc
Moiety formula	C24 H24 N6, C2 H6, 2(Cl)	2(Cl), C24 H24 N6, C2 H6
Sum formula	C26 H30 Cl2 N6	C26 H30 Cl2 N6
Mr	497.46	497.46
Dx,g cm-3	1.367	1.367
Z	4	4
Mu (mm-1)	0.296	0.296
F000	1048.0	1048.0
F000’	1049.46	
h,k,lmax	14,18,18	14,18,18
Nref	4740	4735
Tmin,Tmax	0.952,0.978	0.541,0.663
Tmin’	0.952	

Correction method= # Reported T Limits: Tmin=0.541 Tmax=0.663
AbsCorr = MULTI-SCAN

Data completeness= 0.999 Theta(max)= 26.000

R(reflections)= 0.0650(3810) wR2(reflections)= 0.1868(4735)

S = 1.031 Npar= 320

The following ALERTS were generated. Each ALERT has the format
test-name_ALERT_alert-type_alert-level.
Click on the hyperlinks for more details of the test.

Alert level B

```
RINTA01_ALERT_3_B  The value of Rint is greater than 0.18
            Rint given   0.203
```

Author Response: Due to poor quality of crystal.

Alert level C

```
PLAT042_ALERT_1_C Calc. and Reported MoietyFormula Strings  Differ     Please Check
PLAT340_ALERT_3_C Low Bond Precision on  C-C Bonds ...............    0.00505 Ang.
PLAT360_ALERT_2_C Short  C(sp3)-C(sp3) Bond  C25      - C26      .       1.40 Ang.
PLAT413_ALERT_2_C Short Inter XH3 .. XHn     H3       ..H26A     .       2.14 Ang.
                                                 2-x,1-y,-z  =      3_765 Check
PLAT413_ALERT_2_C Short Inter XH3 .. XHn     H10      ..H26A     .       2.13 Ang.
                                           -1+x,3/2-y,1/2+z  =      4_476 Check
PLAT413_ALERT_2_C Short Inter XH3 .. XHn     H12      ..H26B     .       2.05 Ang.
                                                   -1+x,y,z  =      1_455 Check
PLAT906_ALERT_3_C Large K Value in the Analysis of Variance ......      3.378 Check
PLAT911_ALERT_3_C Missing FCF Refl Between Thmin & STh/L=    0.600          5 Report
PLAT977_ALERT_2_C Check Negative Difference Density on H26A      .      -0.41 eA-3
PLAT977_ALERT_2_C Check Negative Difference Density on H26B      .      -0.43 eA-3
PLAT977_ALERT_2_C Check Negative Difference Density on H26C      .      -0.48 eA-3
```

Alert level G

```
PLAT020_ALERT_3_G The Value of Rint is Greater Than 0.12 .........      0.203 Report
PLAT380_ALERT_4_G Incorrectly? Oriented X(sp2)-Methyl Moiety .....        C14 Check
PLAT432_ALERT_2_G Short Inter X...Y Contact  Cl1      ..C26      .       3.06 Ang.
                                                   -1+x,y,z  =      1_455 Check
PLAT913_ALERT_3_G Missing # of Very Strong Reflections in FCF ....          3 Note
PLAT933_ALERT_2_G Number of HKL-OMIT Records in Embedded .res File          2 Note
PLAT967_ALERT_5_G Note: Two-Theta Cutoff Value in Embedded .res ..       52.0 Degree
PLAT978_ALERT_2_G Number C-C Bonds with Positive Residual Density.          0 Info
```

0 **ALERT level A** = Most likely a serious problem - resolve or explain
1 **ALERT level B** = A potentially serious problem, consider carefully
11 **ALERT level C** = Check. Ensure it is not caused by an omission or oversight
7 **ALERT level G** = General information/check it is not something unexpected

1 ALERT type 1 CIF construction/syntax error, inconsistent or missing data
10 ALERT type 2 Indicator that the structure model may be wrong or deficient
6 ALERT type 3 Indicator that the structure quality may be low
1 ALERT type 4 Improvement, methodology, query or suggestion
1 ALERT type 5 Informative message, check

It is advisable to attempt to resolve as many as possible of the alerts in all categories. Often the minor alerts point to easily fixed oversights, errors and omissions in your CIF or refinement strategy, so attention to these fine details can be worthwhile. In order to resolve some of the more serious problems it may be necessary to carry out additional measurements or structure refinements. However, the purpose of your study may justify the reported deviations and the more serious of these should normally be commented upon in the discussion or experimental section of a paper or in the "special_details" fields of the CIF. checkCIF was carefully designed to identify outliers and unusual parameters, but every test has its limitations and alerts that are not important in a particular case may appear. Conversely, the absence of alerts does not guarantee there are no aspects of the results needing attention. It is up to the individual to critically assess their own results and, if necessary, seek expert advice.

Publication of your CIF in IUCr journals

A basic structural check has been run on your CIF. These basic checks will be run on all CIFs submitted for publication in IUCr journals (*Acta Crystallographica*, *Journal of Applied Crystallography*, *Journal of Synchrotron Radiation*); however, if you intend to submit to *Acta Crystallographica Section C* or *E* or *IUCrData*, you should make sure that full publication checks are run on the final version of your CIF prior to submission.

Publication of your CIF in other journals

Please refer to the *Notes for Authors* of the relevant journal for any special instructions relating to CIF submission.

PLATON version of 10/05/2023; check.def file version of 10/05/2023

Datablock mo_2023_eehk10_10b_0ma - ellipsoid plot

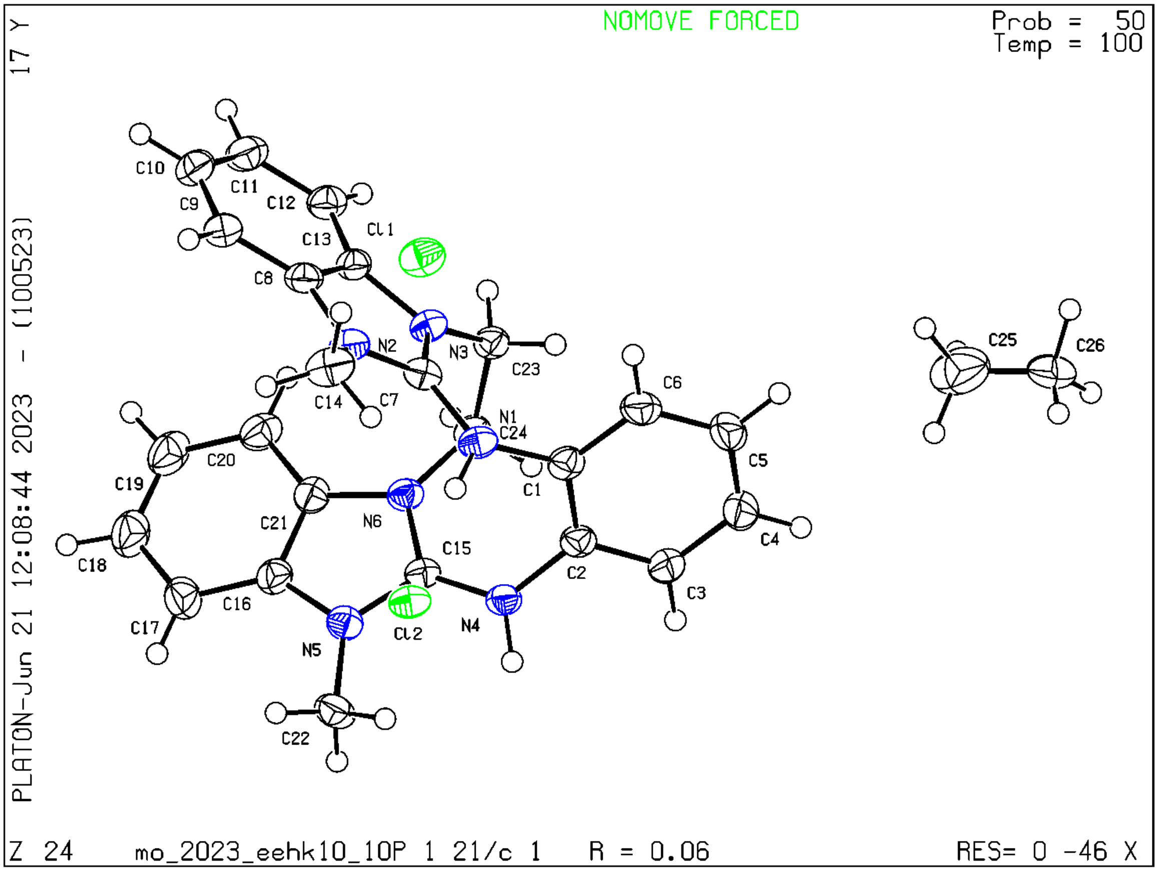

Supplement: Supplementary file 2 — Supporting Information [file mo_2023_eehk10_10b_0ma_cifreport.pdf › mo_2023_eehk10_10b_0ma_cifreport.pdf]
